# Extraordinary diversity among members of the large gene family, *185/333*, from the purple sea urchin, *Strongylocentrotus purpuratus*

**DOI:** 10.1186/1471-2199-8-68

**Published:** 2007-08-15

**Authors:** Katherine M Buckley, L Courtney Smith

**Affiliations:** 1 George Washington University, Department of Biological Sciences, Washington DC 20052, USA

## Abstract

**Background:**

Recent analysis of immune-related genes within the sea urchin genome revealed a number of large gene families with vertebrate homologues, such as the Toll-like and NOD/NALP-like receptor families and C-type lectins in addition to a rudimentary complement system. Therefore, the immune response of the purple sea urchin appears to be more complex than previously believed. Another component of the sea urchin immune response is an unusual family of mRNAs, known as *185/333*, which is strongly upregulated in response to pathogen challenge. The work presented here indicates that this family of transcripts is derived from an unexpectedly diverse gene family.

**Results:**

The *185/333 *genes are small (< 2 kb) with only two exons. Their extraordinary diversity was exemplified by 121 unique sequences identified from 171 cloned genes. Sequences from the second exons were aligned optimally by introducing large gaps, which defined blocks of sequence known as elements. Genes were defined by the presence or absence of elements. Phylogenetic analysis defined five intron types which, when combined with the exon element patterns resulted in 31 gene patterns, 14 of which were not described previously. Sequence diversity was present in all elements, and was higher in the intron than the exons. Repeats within the sequence facilitated multiple alignments, of which two were analyzed in detail. Although the two alignments differed in length, number of elements, and number of patterns, both were about equally accurate at describing the *185/333 *sequences. The genes were closely linked and flanked by short repeats. The repeats within and between the genes may promote their diversification through gene conversion, recombination, and meiotic mispairing.

**Conclusion:**

The diversity of the *185/333 *gene family represents an intriguing addition to what is known about the *S. purpuratus *immune response, and provides further evidence that invertebrate immune systems are neither simple nor static.

## Background

Diversified immune responses provide hosts with advantages in the arms race against pathogens. Diversification of immune-response proteins enables organisms to detect and combat a broad array of pathogens with great precision. Although it was long believed that invertebrate organisms possessed simple, static immune systems, recent evidence from numerous organisms suggests that this may not be correct (reviewed in [1]). In the freshwater snail, *Biomphalaria glabrata*, a family of genes encodes fibrinogen-related proteins (FREPs), which contain IgSF and fibrinogen domains [2-9]. Although it is not known how many FREP genes are present in the snail genome, at least 13 subfamilies exist, with some predicted to contain as many as eight loci. These genes are expressed in response to trematode parasites and produce a large repertoire of mRNAs that encode a diverse set of proteins. It is believed that a limited set of source sequences is used to generate FREP diversity through point mutation and somatic recombination [6]. A comparably diverse gene family in amphioxus, *Branchiostoma floridae*, encodes V region-containing chitin-binding proteins (VCBP), which contain two Ig V-type domains [10]. Five subfamilies of VCBPs are known to exist based on sequence analysis. Higher plants also have large gene families that encode resistance (R) proteins. R gene diversity results from extensive recombination, mispairing, gene duplication, and gene conversion [11-13].

Analysis of the sea urchin genome revealed a number of large gene families putatively involved in the sea urchin immune response [14], in addition to the previously identified complement system [14-21]. The *S. purpuratus *genome contains 222 predicted gene models that encode Toll-like receptors (TLRs) and 203 that encode NOD/NALP-like receptors (NLRs) [14]. Both the TLR and NLR gene families are components of vertebrate innate immune systems, although with significantly fewer versions (there are 10 TLR and approximately 20 NLR genes in humans [14]). Although little is known about the functions of these receptor genes in the sea urchin, their diversity suggests that they can recognize a broad range of pathogenic signatures collectively or combinatorially [22]. An additional 218 gene models encoding scavenger receptor cysteine-rich receptors (SRCR) were predicted from the sea urchin genome [14]. Previous studies have shown that the expressed SRCR transcript repertoire is transient in unchallenged sea urchins and changes in response to injury or pathogenic challenge [23]. SRCRs are members of a metazoan superfamily with a variety of functions, including the regulation and development of vertebrate immune responses [24-26]. Thus, large gene families appear to be a common theme in the sea urchin immune response.

Additionally, a diverse set of transcripts called *185/333 *is strongly upregulated in response to a variety of immunological challenges [27-30]. *185/333 *sequences are of particular interest due to their diversity and expression patterns. Of 81 originally characterized *185/333 *cDNA sequences, 67 had unique nucleotide sequences encoding 64 unique protein sequences [27]. Further analysis of expression patterns indicated that certain *185/333 *sequences may be preferentially upregulated in response to particular pathogen-associated molecular patterns (PAMPs) [29]. Alignment of the transcripts required the insertion of large gaps, creating 25 blocks of sequence called elements, and an identifiable leader. The sequences also contained five types of repeats, the borders of which did not correspond to the element borders. Because individual *185/333 *sequences included a variable subset of elements, their presence and absence were used to define element patterns. Specific patterns observed in numerous clones defined cDNA sets [27]. Although the mRNA structure strongly implied alternative splicing [27], two assembled *185/333 *genes from an early assembly of the *S. purpuratus *genome indicated that, with the exception of the leader, all elements were located on a single exon [30]. Due to the substantial diversity of the mRNA and the unexpectedly simple gene structure, it was of interest to characterize the *185/333 *gene family more closely.

The results presented here illustrate that the observed *185/333 *transcript diversity is at least partially the result of a highly diverse family of genes present in the sea urchin genome. Previous studies suggest that there are about 100 different *185/333 *alleles per sea urchin [30]. The diversity of *185/333* is illustrated by the fact that 71% of 171 cloned and sequenced genes were unique, and from these, 33 element patterns were identified, about half of which were newly described. From this and previous studies [27,29,30], a total of 51 element patterns are now known from 16 *S. purpuratus *individuals. We also show that the closely linked genes are flanked by short repeats, which may facilitate diversification through gene conversion, recombination, or meiotic mispairing. Overall the data show that the *185/333 *gene family represents a new example of molecular diversity among invertebrate immune systems.

## Results

### The 185/333 genes are variable in size

**Figure 1:**
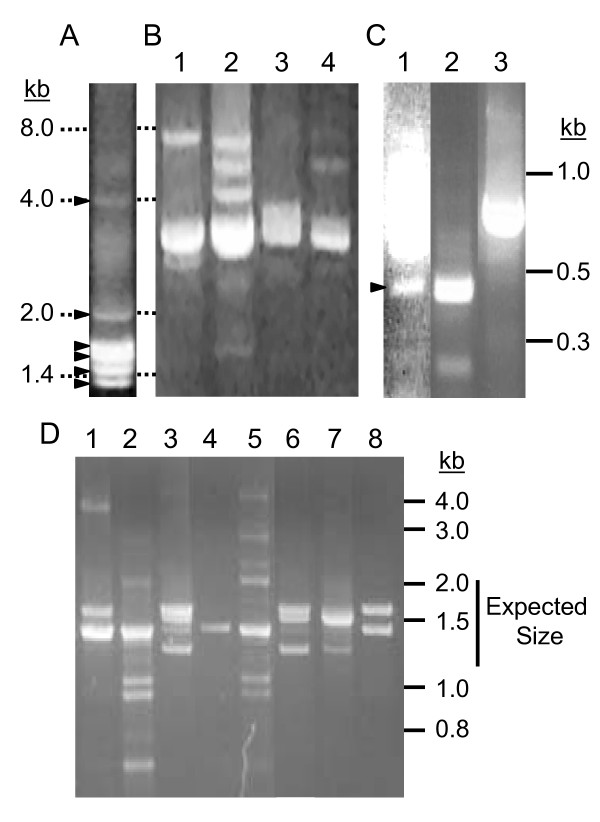
**Amplification of *185/333 *sequences**. **A**. gDNA from animal 2 was amplified by PCR using primers flanking the *185/333 *genes (185-5'UTR and 185-3'UTR; see Table 1) and showed bands of five major sizes from 1.2 to 2 kb and a minor band around 4 kb. Triangles indicate the positions of each of the bands. Similar amplification of gDNA from six additional animals had identical patterns of bands (data not shown). **B**. Amplification of intergenic regions by PCR using primers that annealed within genes but were oriented away from each other (185-LR1 and 185-F5 primers; Table 1) revealed a major band in all animals of about 3 kb (lanes 1–4, animals 10–13). **C. **gDNA from animal 2 was amplified by PCR using primers that annealed in the 5' UTR (185-5' UTR; Table 1) and the type I repeats found in elements Ex4, Ex5, and Ex6(185-R5; Table 1) and revealed the presence of genes that lacked introns. Animal 2 gDNA (lane 1) shows a band of 450 bp (indicated with a carrot). A cDNA (*Sp0313*, DQ183171; lane 2) also amplifies a band of about 450 bp. A cloned gene (2-02, Additional file 1; GenBank accession number EF607673; lane 3) with a typical intron amplifies a band within the range of bands in **A**. **D. **PCR amplification of *185/333 *from eight different BAC clones using primers (185-5'UTR and 185-3'UTR; Table 1). Each lane contains template DNA from a different BAC (1, 126J14; 2, 108H07; 3, 053M03; 4, 121A07; 5, 004D19; 6, 182N21; 7, 148J22; 8, 019L13).

Searches of the initial assembly of the *S. purpuratus *genome (assembly 09/22/03; http://hgsc.bcm.tmc.edu/projects/seaurchin) identified two *185/333 *genes, which were small (<2 kb) [30]. It was therefore feasible to analyze entire genes amplified by PCR from genomic DNA (gDNA) using primers flanking the *185/333 *coding region (185-5'UTR and 185-3'UTR; Table 1). Analysis of the *185/333 *genes using PCR revealed five major sizes of genes ranging from 1.2 to 2.0 kb. A representative result from one of six sea urchins is shown in Figure 1A. Amplicons derived from three different sea urchins were cloned and sequenced. In total, 171 genes were cloned and sequenced from gDNA: 87 genes from animal 2 coelomocytes, 34 genes from animal 4 coelomocytes, and 50 genes from animal 10 sperm. Clones with identical nucleotide sequence were assumed to be derived from the same gene and omitted from further analysis. In total, 121 unique genes were identified: 53 from animal 2, 30 from animal 4, and 38 from animal 10. The genes ranged in size from 1206 to 1894 nucleotides (nt) (Figure 1A; Additional file 1). The structure of all cloned genes was consistent with previous data [30] and had two exons separated by a small intron. The first exon was short (51 or 54 nt) and encoded the leader, whereas the much longer second exon (771–1431 nt) encoded the remainder of the open reading frame (ORF). The intron ranged in size from 380 to 413 nt (average = 407 nt). Although a minor band of ~4 kb was also observed, no genes larger than 2 kb were cloned. Therefore, the structures of these putative genes are unknown.

**Table 1:** Primers used in PCR and sequencing

**Primer**	**Sequence (5' → 3')**	Annealing site*
185-5'UTR	TAGCATCGGAGAGACCT	5'UTR
185-3'UTR	AAATTCTACACCTCGGCGAC	3'UTR
185-LR1	ATCRTYGCCATYSTGGCYG	Leader
185-F2	AAGMGATTWCAATGAACKRCGAG	Ex1/Er1
185-F5	GGAACYGARGAMGGATCTC	Ex25/Er27
185-F6	GAAGAAGAAACTGATGCTGCC	Ex7/Er6
185-R5	ACTCTGTACTGCGGAGAGCCGAC	Ex4/Er3, Ex5/Er4 and Ex6/Er5
185-R6	GCAGCATCAGTTTCTTCKTCTC	Ex7/Er6
185-R9	CTTHARGTGGTGAARATGTCG	Ex25/Er27
185-R11	ATCTCCCAGGCGTGATG	Ex25/Er27

**Figure 2:**
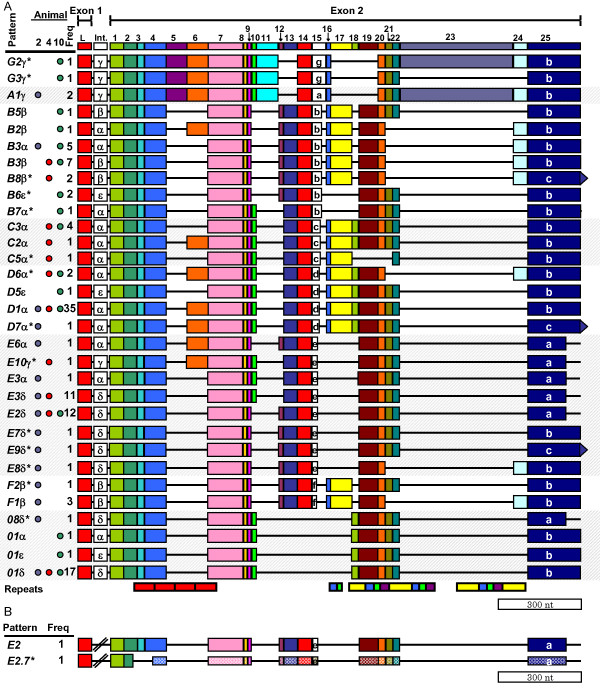
**cDNA-based Element patterns of the *185/333 *genes**. **A**. Exon element patterns. Gene sequences were manually aligned based on alignments of cDNA sequences. The insertion of gaps (black lines), which defined the 25 elements (shown as colored boxes) [30]. A consensus sequence containing all the elements and the locations of the two exons is shown at the top. The source of each gDNA is indicated by the colored dots in the columns labeled "Animal". Blue dots indicate that the pattern was isolated from animal 2, red dots from animal 4, and green dots from animal 10. The frequency with which the patterns were found is indicated in the column labeled "Freq". The element patterns are sorted into groups (grey background shading) based on the subtype of Ex15 (specified by the letter in the box). The locations of the five types of repeats [27,30] are shown at the bottom of **A **(red boxes indicate type 1 repeats; blue = type 2; green = type 3; yellow = type 4; and purple = type 5). Patterns not previously identified from cDNA analysis [27,30] are denoted with an asterisk (*). The position of the stop codon in element Ex25 is indicated by the letter present in the box and the size/shape of the box (Additional file 2, Additional file 3). There are 31 unique patterns based on both exon elements and intron type. **B: **Intronless genes with unique element patterns amplified from BAC DNA. Untranslated sequence (due to a missense deletion) is indicated by lighter shading and narrower boxes for *E2.7*. The locations of the repeats are the same as **A**.

*The annealing site indicates the elements in which the primers will hybridize in either the cDNA-based alignment (elements denoted as Ex; Figure 2) or repeat-based alignment (elements denoted as Er; Figure 6).

### Elements and patterns

#### Exon 2 elements

To align the gene sequences optimally, large gaps were inserted that created discrete sections of sequence called elements (Figure 2A, Additional file 2 and Additional file 3). The borders of the elements located within the second exon were defined based on the positions of gaps in an alignment of *185/333 *cDNAs [30]. All 25 previously identified elements were found among the gene sequences, and no new elements were found. The elements, named Ex1–25, to indicate that they are located within the exon, ranged in size from 12 to 357 nt (Additional file 4). All genes contained elements Ex1, 2, 3, 4, 7, 8, 9, and 25, whereas Ex23 was found in only three out of 171 genes. Previously, seven subelements of Ex15 were defined from the cDNA sequences based on length [30] all of which were found in the gene sequences. Subelements Ex15a, Ex15c, Ex15f, and Ex15g were identified from single sea urchins (Figure 2A), suggesting that Ex15 subtypes may not be present in all members of the population.

The terminal element in both the cDNA [30] and gene sequences, Ex25, was characterized by stop codons in three locations, which divided Ex25 into three regions, Ex25a (138 nt), 25b (57 nt) and 25c (Additional file 2 and Additional file 3). The final stop codon, which defined element Ex25c, was not found in the gene sequences because the primer used for cloning (185-3'UTR; Table 1) was located in 25c just 5' of the third stop codon. Furthermore, there were three distinct subtypes of element Ex25b in the cDNA sequences based on the presence or absence of a series of gaps in one of two positions [30], all of which were also identified among the gene sequences (Additional file 2, Additional file 3).

The frequency with which each of the elements was present within the clones was relatively constant among the three animals studied (data not shown). The exceptions were pairs of mutually exclusive elements, of which one or the other was found in all clones analyzed. One such pair of elements was Ex10 and Ex12. In animal 2, 66% of clones had element 10 and 34% had Ex12, whereas in animal 4, 87% of clones had element 10 and 16% had Ex12. It is of note that Ex10 and Ex12 were 92% similar at the nucleotide level and therefore may encode regions of similar function in the protein.

#### Patterns

##### Exon element patterns

The variable presence of individual exon elements in the gene sequences defined 27 different exon element patterns, of which fourteen had not been defined previously [29,30] (Figure 2A; asterisked patterns). As previously noted [30], subtypes of Ex15 correlated with the presence or absence of suites of other elements and were used to define groups of patterns. These associations also held for the gene sequences (Table 2; Figure 2A). Furthermore, Ex21 and Ex22 were always found together, and the pair was mutually exclusive with Ex24, unless Ex23 was present. Ex10 and Ex12 were also mutually exclusive, as noted above. These two elements were distinguished by the first amino acid of the element (Ex10 either had a proline or a leucine, whereas Ex12 had a histidine). Ex14 and Ex15 always occurred together, regardless of the Ex15 subtype (Figure 2A). Therefore, not only did suites of elements appear in association with subtypes of Ex15, but elements also commonly appeared either in association with or in the absence of other elements.

**Table 2:** Suites of elements associated with Er10/Er15 subtypes

			cDNA-based alignment		Repeat-based alignment	
			
Ex15/Er10 Subtype	Intron types	# Genes	# Exon Patterns	Elements Present*	# Exon Patterns	Elements Present^+^
a	γ	2	1	5, 6, 10, 11, 14–16, 20–24	1	3, 4, 7, 9–16, 18–25
b	α,β,ε	19	7	13–15, 19, 20	5	8–11, 14, 25
c	α	6	3	10, 13–17, 22	3	8–11, 14, 16, 17, 21, 22
d	α,ε	39	4	10, 13–20,	2	8–11, 14, 16, 17, 21–25
e	α,γ,δ	29	7	13–15, 19–20	4	8–11, 14, 25
f	β	4	2	12–17, 19–20	1	8–11, 14, 16, 17, 21, 23–25
g	γ	2	2	5, 6, 10, 11, 14–16, 20–22	2	3, 4, 7, 9–11
0	α,ε,γ	20	2	10, 18–22	1	22–25

*Elements Ex1–4, 7–9, and 25 were omitted because they were present in all genes (Figure 2A)

^+^Elements Er1, 2, 5, 6, 26, and 27 were omitted because they were present in all genes (Figure 6)

Genes with Ex15a or Ex15g (element patterns *A1, G2 *and *G3*) had nearly identical combinations of elements (Table 2; Figure 2A). Ex15g and Ex15a may therefore represent population-level differences of a single Ex15 subtype rather than two distinct subtypes. This was supported by the fact that a) each subtype was isolated from an individual animal, b) element Ex15g was only a single amino acid longer than Ex15a and was otherwise identical in sequence, and c) the element patterns of these genes were very similar. However, a single clone (10-013; Additional file 1), with element pattern *G3*, contained most of the typical group *A/G *hallmarks, but lacked elements Ex23 and Ex24 (Figure 2A). Of the more than 810 gene and transcript sequences analyzed thus far [29,30], Ex15a and Ex15g always occurred in association with Ex23, with the exception of *G3*. Furthermore, the sequence of the *G3 *clone was identical to the single *G2 *clone from the same animal, with the exception of the absence of Ex23 and Ex24 (Additional file 2). Therefore, the *G3 *type gene may have arisen as a result of a duplication of the *G2 *gene, followed by a deletion of elements Ex23 and 24.

##### Intron types

**Figure 3:**
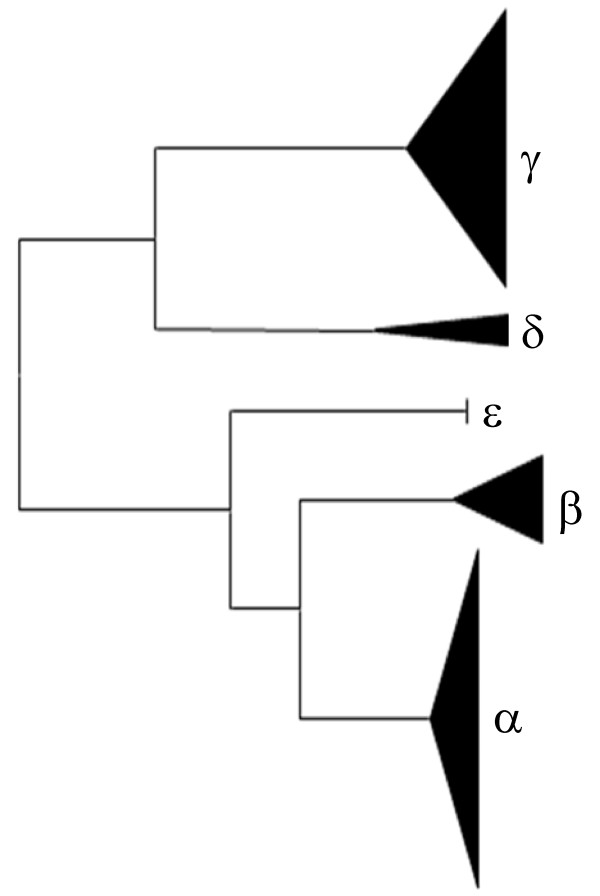
**Phylogenetic tree of intron sequences**. Intron sequences were aligned manually (Additional file 2). Maximum likelihood and maximum parsimony methods both produced the unrooted tree shown. The five major clades were used to define the five intron types, as shown.

The intron sequences were aligned and compared using phylogenetics. Intron types were found between 4 (ε) and 54 (α) times. There was strong support to separate the introns into five major clades (α-ε), however, the relationships among the intron sequences within each major clade was generally unresolved (Figure 3). The resulting tree indicated two larger clades composed of intron types δ and γ, and intron types α, β, or ε, which illustrates sequence similarities among the introns. Genes were therefore named according to exon pattern and intron type (i.e., gene pattern *D1*α had exon element pattern *D1 *and intron type α).

A total of 31 gene element patterns were defined by the combination of exon element patterns and intron types (Figure 2A; Figure 3), 12 of which formed gene sets composed of two or more unique genes with identical element patterns. Eight patterns were collected from more than one animal. Genes that formed sets were isolated from either a single animal (*D5*ε was present only in animal 2), or from all three animals (*E2*δ). The three patterns shared among all three animals, *E2*δ, *D1*α, and *01*δ display increased transcription after sea urchins are challenged with various PAMPs [29]. The most commonly observed transcript pattern, *E2*, was found 12 times among the genes, suggesting that its increased transcription may result from an increased number of genes.

Generally, exon element patterns correlated with intron type (Figure 2A). However, there were three instances in which genes with the same exon element patterns had different intron types. Exon element pattern *01 *correlated with three different intron types, α, δ, or ε. Gene pattern *01*δ was identified in the majority of the *01 *clones (17 of the 19 *01 *clones), whereas *01*α and *01*ε were isolated only once (Figure 2A). Exon element patterns *E3 *and *B3 *also had different intron types (*E3 *genes had either intron α or δ, whereas *B3 *genes had either introns α or β). Genes with identical intron element patterns also had different exon element patterns, such as *C3*α and *D1*α (Figure 2A; Figure 3). Therefore, although exon element patterns typically correlated with a single intron type, there were examples when they did not.

### Diversity

As expected from previous analyses of the cDNA sequences [27,29,30], the *185/333 *genes comprise an exceptionally diverse family. Identical gene sequences were not shared among the three sea urchins. *Taq-*induced polymorphisms and template switching were ruled out as sources of diversity (Additional file 5). To quantify the diversity, two approaches were employed: calculations of entropy-based diversity scores and ratios of synonymous to non-synonymous nucleotide substitutions [30]. Diversity scores were calculated as in [29,30] using a formula based on the method of [31], and depend upon the frequency of the nucleotides or amino acids at each alignment position, but are independent of the sequence length or size of the data set. Although the maximum possible diversity score is 1.609, a more realistic biological maximum score might be similar to that previously observed for element 11 from expressed cDNA sequences [30], or about 0.600. Diversity scores provide a means to identify variable regions, and can be used to compare the diversity of different elements or different sets of genes.

#### Nucleotide diversity of the elements

**Figure 4:**
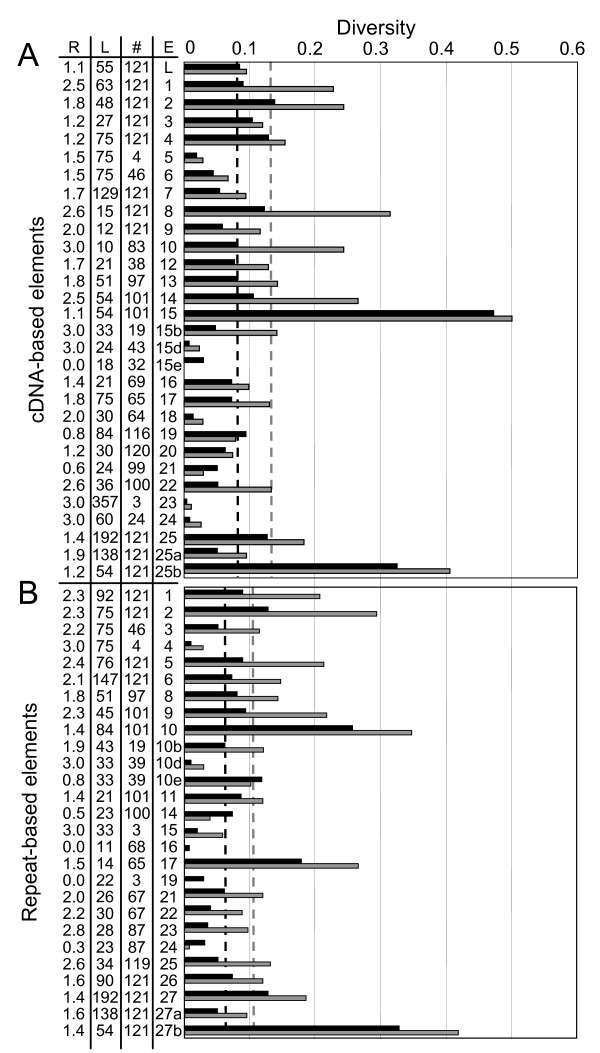
**Diversity of the *185/333 *gene elements**. Nucleotide diversity scores (black bars) and amino acid diversity scores (gray bars) of individual exon elements are shown. The average element nucleotide diversity is indicated by a dashed black line and average element amino acid diversity is shown as a dashed gray line. The absolute range of diversity scores is 0 (entirely conserved) to 1.609 for nucleotide alignments (based on an even distribution of five states: four nucleotides and a gap) or 3.044 for amino acid alignments (21 possible states) [30]. The highest element diversity score previously observed was from element 11 (0.5381) from a set of cDNAs isolated from bacterially-challenged coelomocytes pooled from five animals [30]. This score was similar to that obtained from a modeled element in which 40% of the positions contained four different states in 20% of the sequences. Elements with a diversity score of 0 were omitted (Ex11, Ex15a, Ex15c, and Ex15f-g of the cDNA-based alignment (**A**) and Er7, Er10a, Er10c, Er10f-g, Er12 -13, Er18, and Er20 of the repeat-based alignment (**B**). The number (#), length in nucleotides (L) and nucleotide to amino acid diversity ratio (R) are indicated in the table to the left of the graphs. Ex25 includes both Ex25a and Ex25b. Ex25a includes all sequences from the start of Ex25 up to and including the first stop codon. Ex25b includes only the sequence following the first stop codon to the second stop codon (Additional file 2 and Additional file 3). Element Er27 in the repeat-based alignment (**B**) is treated similarly.

Analysis of the exon element diversity scores indicated that, with the exception of elements Ex15 and Ex25b, the nucleotide diversity ranged from 0 to 0.1364 (Ex2) (average = 0.0846 ± 0.0878), and was distributed throughout all of the elements (Figure 4A). Two dissimilar versions of element 11 were identified from the cDNA sequences, which was the source of the high diversity score [30]. However, only a single, entirely conserved version of Ex11 (equivalent to Ex11b [30]), was identified in the gene sequences. Elements Ex25b and Ex15 had the highest diversity scores (0.3237 and 0.4093, respectively) as a result of the subtypes of these elements. When subtypes were analyzed separately, diversity scores for the three non-conserved Ex15 subtypes (Ex15b, Ex15d, and Ex15e) were < 0.0471 (Figure 4A). Likewise, the nucleotide diversity of Ex25a was much lower than the diversity of Ex25b (0.0495 vs. 0.3237, respectively), due to the three subtypes of Ex25b. When analyzed separately, the Ex25b variants also had considerably lower scores (nucleotide diversity scores of Ex25b1 = 0.0977; Ex25b2 = 0.0165; Ex25b3 = 0.0079). The exon element diversity varied throughout the sequence of the genes and greatly increased when element subtypes were analyzed together.

The overall sequence diversity in the dataset stemmed from the interplay between four possible sources: a) SNPs present only in individual genes (unshared variations), b) the number of variable positions in an alignment, c) the number of states in each alignment position, and d) the number of unique sequences. Because diversity scores rely on the frequency of each state in each alignment position, changes that occur in a single sequence have minimal effects on the overall diversity. However, as the total number of sequences decreases, the impact of these variations on the overall diversity scores increases. The range of percent variable positions among the elements was 0 (Ex11) to 88.9% (Ex15). Ex15 had both the greatest percent of variable positions and the highest nucleotide diversity score. The average number of states per variable nucleotide position among the elements (excluding the conserved Ex11) was 2.1.

A set of unique element sequences was generated from the combination of unique SNPs and variable position characteristics. Each exon element had a suite of distinct, but related, nucleotide sequences, with an average of 11.2 unique sequences per exon element (Table 3; Additional file 4). The number of and differences among these sequences was the primary factor in the diversity scores. Increased element diversity scores correlated with increased numbers of unique element sequences. Ex4 and Ex20 were identified 121 and 120 times, respectively, but Ex4 had 21 unique sequences and a higher nucleotide diversity score (0.1278) than element Ex20 (0.0619), which had eight distinct sequences (Additional file 4). Overall, the four diversity characteristics influenced the diversity scores by varying amounts and were useful means by which to characterize and compare the elements.

**Table 3:** Diversity of the Intron and Exon^+^

Sequence	Length	Nt Diversity	# Unshared Variations (%)^1^	% Variable Positions	# Unique Sequences
cDNA-based 2^nd ^Exon	1695	0.0522	93 (5.5)	18.2	112
Intron	446	0.1752*	25 (5.6)	40.1*	52
Repeat-based 2^nd ^Exon	1449	0.0556	102 (7.0)	21.3	112

^+ ^Gaps were omitted from all calculations.

^1 ^The percent of the total positions within the alignment that contain an unshared variation.

* Indicates that the intron values were significantly different than the values for both the cDNA-based 2^nd ^exon and the repeat-based 2^nd ^exon (p < 0.05) based on a two-tailed t-test.

#### Amino acid sequence diversity of the elements

The diversity of the amino acid sequences encoded by the exon elements was analyzed using the same approaches used to characterize nucleotide diversity. Overall, amino acid element diversity ranged from 0 to 0.4977 (Ex15; average = 0.1350 ± 0.1120; Figure 4A, gray bars), and generally correlated with nucleotide element diversity scores. The average number of unshared variations for the amino acid element sequences was 2.81, with a maximum of 24 in Ex7. On average, 38% of amino acid positions were variable, higher than the average number of variable nucleotide positions (26%), underscoring the high number of nonsynonymous substitutions within the data set. The average number of states per variable position over all elements was similar to that observed in the nucleotide alignments (2.2 for amino acids vs. 2.1 for nucleotides). The highest average number of states per variable position within an element was Ex21, which had 3. However, this number is low, given that the theoretical maximum number of states per position is 21 amino acids. Furthermore, the average number of unique amino acid sequences is lower than that in the nucleotide alignments (8.7 for amino acids vs. 11.2 for nucleotides). Therefore, although some of the diversity is masked in the encoded proteins by synonymous substitutions, if all of the genes are translated, much of the diversity would permeate through to generate a highly diverse pool of proteins.

#### Ratios of nucleotide and amino acid diversity scores

Ratios of the nucleotide vs. amino acid diversity scores (diversity ratios) provided insight into the relative levels of synonymous and non-synonymous substitutions among the elements [29,30]. This approach was implemented because the elements were generally too short to determine dn/ds ratios using PAML [32]. If each nucleotide change resulted in an amino acid change, the ratio of amino acid diversity to nucleotide diversity would equal 3 because the number of variable positions and the diversity of those positions would remain constant, but the length of the nt vs. amino acid sequence would differ by a factor of 3 (3 nucleotides encode 1 amino acid). However, if all nucleotide changes were silent, the resulting amino acid diversity and diversity ratio would equal 0. Specifically, the diversity ratio indicates how many of the three variable positions in a codon result in non-synonymous changes. A high diversity ratio indicates primarily non-synonymous changes, and a low ratio indicates mostly synonymous changes. There was no correlation between diversity ratios and diversity scores.

The two elements with diversity ratios of less than 1 (fewer than one out of three nucleotide changes resulted in an amino acid change) were Ex19 (0.83) and Ex21 (0.60), suggesting that these elements had the greatest number of synonymous nucleotide substitutions (Figure 4A). Elements with ratios greater than 2.5 were Ex1, Ex8, Ex10, Ex22, Ex23, and Ex24 (Figure 4A). Ex8, Ex10, Ex23, and Ex24 exhibited artificially high ratios due to a low number of nucleotide changes, most of which encoded non-synonymous changes. For example, Ex23 had two non-synonymous nucleotide mutations that resulted in a ratio close to 3. Overall, 15 of the 25 elements were characterized by diversity ratios of at least 1.5 (Figure 4A), indicating that, on average for these elements, half of the nucleotide substitutions resulted in a nonsynonymous amino acid change. The amino acid diversity scores therefore illustrated that not only are the *185/333 *genes diverse, but much of this diversity encodes changes to the amino acid sequence.

#### Diversity of the intron

The diversity of the intron sequences was measured using the same characteristics as those used to understand the diversity of the exon elements. To compare intron and exon diversity, diversity characteristics for the entire second exon were analyzed disregarding all gaps (including those that defined the elements). Therefore, the values for the second exon were lower than when the second exon was analyzed including gaps as a fifth state. The diversity of the intron was over 3 times higher than the second exon (Table 3). This elevated diversity resulted from a significant increase in the percent variable positions in the intron (with respect to the lengths of the entire alignments). Within the intron sequence, ~40% of the alignment positions were variable, as compared to only 18% in the second exon (Table 3).

#### Diversity of individual nucleotide positions

**Figure 5:**
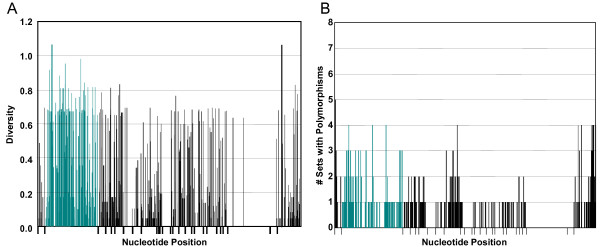
**Diversity of nucleotide positions**. A. Each bar represents the nucleotide diversity of a single nucleotide position within the alignment of all cloned *185*/333 genes. Green bars represent nucleotide positions within the intron. Gaps due to missing elements were excluded from the diversity calculations. B. Hypervariable positions within the *185/333 *genes were identified using diversity analysis on clones belonging to cDNA-based sets (Table 5) with more than two members (94 genes total). Because sets had variable numbers of members, which artificially increases diversity for positions in sets with fewer members, each diversity score greater than 0 was assigned a value of 1. Each bar in the graph represents the number of sets (out of 8 total) that contained a polymorphism at the specific nucleotide position. Green bars indicate positions within the intron. Bars below the x-axis denote the borders between elements (cDNA-based alignment).

Diversity of individual nucleotide positions across all genes was analyzed, omitting gaps, and results indicated that diversity was relatively constant throughout the alignment with no strikingly hypervariable regions (Figure 5A). The positional nucleotide diversity scores ranged from 0 to 1.0677 (position 2046, located in Ex25; Additional File 1), with an average of 0.0773. A few conserved stretches were apparent, such as Ex23, which had only two variable positions, in addition to regions within Ex5 and Ex11 were noticeably more conserved than the rest of the sequence. Because most of the variability resulted from differences among the unique element sequences, diversity was calculated for each position for each set of genes with shared element patterns (Figure 5B). To normalize the unequal number of members in a gene set, the number of sets containing polymorphisms at each position within the alignment was calculated. This approach identified positions that were commonly variable among genes with identical element patterns and disregarded inter-set differences (Figure 5B). There were 632 variable positions in the entire alignment of 2298 nucleotides. However, only 105 positions were polymorphic among the different sets. Three adjacent sites were polymorphic in five of the eight sets that composed a single codon indel located in the leader (nt positions 4–6, Additional file 2). There were 12 positions in which four of the sets were polymorphic; three were located in the intron, eight were in Ex25, and one was in Ex 7 (Additional file 2). Therefore, much of the diversity appeared to be the result of differences among sets, and was not concentrated in specific regions of the alignment, but rather, was distributed throughout the sequence.

The overall diversity for the entire *185/333 *nucleotide alignment, including gaps as a fifth state, was 0.2692. Separating the sequences by source animal resulted in diversity scores of 0.2532 for the genes from animal 2, 0.2253 for the genes from animal 4, and 0.2856 for the genes from animal 10. If the gene sets were congruous and their combination did not produce any additional diversity, the diversity score for the entire set of genes would be equal to average of the scores from individual animals, weighted by the number of genes isolated from each animal. This weighted average was 0.2565, slightly lower than the observed value. This phenomenon of higher diversity for genes from all three animals combined was not limited to the results from the analysis of the entire alignment, but also to individual elements. For example, Ex5 was conserved within individual animals, but had a diversity score of 0.0185 when all three animals were combined. The increased diversity score for genes from the three animals combined indicated that gene sequences from a single animal were more conserved than the collection of genes across animals. These differences underscored not only the diversity of members of this gene family, but also the diversity of the *185/333 *genes within the population.

### Repeat-based alignment

**Figure 6:**
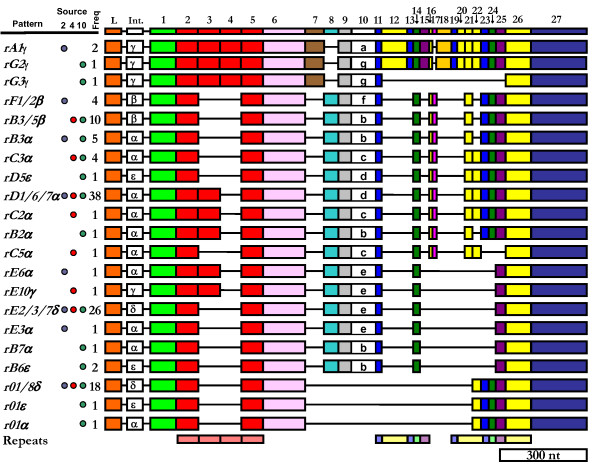
**Repeat-based alignment of the *185/333 *genes**. The alignment was optimized for repeats and 27 elements (colored boxes) were defined. Gaps due to missing elements are indicated by horizontal black lines. There are 17 different exon element patterns, which, when combined with the intron types (Figure 3), form a total of 21 unique gene patterns. The frequency (Freq) of patterns indicates how often the pattern was identified. The source animal for each pattern is indicated by the presence of a colored dot as in Figure 2. The subtype of element Er10 (which corresponds to Ex15, Figure 2A) is indicated by the letter in the box. Intron types are unaffected by this alignment because they do not have repeats; their designation corresponds to that shown in Figure 3.

In agreement with previous analysis of *185/333 *cDNA sequence alignments [27,30], five types of repeats were identified in the gene sequences (shown as colored boxes in Figure 2A). Type 1 tandem repeats (red in Figure 2A) were located in Ex2–7; repeat types 2 through 5 were located in Ex16–23 in mixed groups of interspersed repeats. There was no correlation between the edges of the elements and the edges of the repeats in previous alignments [27,30] or that shown in Figure 2. Furthermore, in the gene alignments, element borders for Ex1–4 were not supported by gaps as in previous message alignments (Figure 2A). Consequently, we explored the possibility of an alternative alignment such that the element borders were defined by the repeats. By modifying the alignment shown in Figure 2 (the cDNA-based alignment) to correlate the edges of the repeats and elements, the set of *185/333 *genes was re-aligned into the repeat-based alignment (Figure 6; Additional file 6 and 7). Elements in the repeat-based alignment were designated Er1 through Er27.

**Figure 7:**
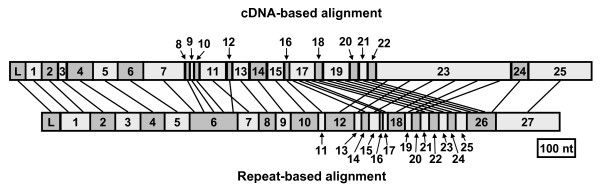
**Correlation of elements in the cDNA-based and repeat-based alignments**. The elements of the cDNA-based and repeat-based alignments are shown as blocks. Lines connecting the blocks indicate the relative positions of elements in the two alignments. In elements ExL to Ex7 and ErL to Er6, the elements correlate closely between the two alignments. Only elements Ex1 and Ex2 of the cDNA-based alignment are merged to form Er1 in the repeat-based alignment. In Ex8 to Ex25, however, the sequences are interdigitated to accommodate the alignment of the repeats, particularly those in Ex23.

The relationship between the elements of the cDNA-based and repeat-based alignments is shown in Figure 7. The edges of the elements for Er1–5 were altered according to the type 1 repeats and Ex1 and Ex2 were merged to form Er1. Ex7 through Ex10 and Ex12 were not defined by gaps in the gene sequences, and were therefore merged to form Er6. To realign the sequences to be in accordance with repeat types 2 through 5, the sequences were interdigitated. As a result Ex23 was divided into six smaller elements in the repeat-based alignment (Er12, 13, 15, 18, 19, and 20; Figure 7). Ex22 and Ex24 were very similar in sequence and were merged into part of Er26. Elements Ex15 and Ex25 were maintained including their subtypes, but were renumbered Er10 and Er27. Therefore, due to the repeats and elements that were unsupported by gaps in the gene sequences, realigning the *185/333 *genes resulted in an agreement between element and repeat borders.

Although the repeat-based alignment had 27 elements, it was shorter than the cDNA-based alignment (Figure 7; Table 4). Overall, the repeat-based elements ranged in length from 11 nt (Er16) to 192 nt (Er27), with an average of 56 nt (Table 4). As in the cDNA-based alignment, repeat-based elements were present in varying frequencies, from 2% (Er12, Er13, Er18, Er19, and Er20) to 100% (ErL, Er1, Er2, Er5, Er6, Er26, and Er27) of the clones. As a result of sequence interdigitation, there were fewer repeats of types 3, 4, and 5 (Table 4).

**Table 4:** Characteristics of the cDNA-based vs. repeat-based alignments

	cDNA-based Alignment	Repeat-based Alignment
2^nd ^exon length (nt)	1721	1524
Number of elements	25	27
Average element Size (nt)	68	56
Range of element Sizes (nt)	12 – 357	11–192
Element Patterns (2^nd ^exon)	27	14
Element Patterns (intron + exons)	31	21
Type 1 repeats (red*)	4	4
Type 2 repeats (blue)	4	4
Type 3 repeats (green)	4	2
Type 4 repeats (yellow)	4	3
Type 5 repeats (purple)	3	2
Average set nt diversity	0.0194	0.0216
Overall nt diversity	0.3011	0.2779
Average element nt diversity	0.0846	0.0629

*Colors of the repeats as shown in Figures 2 and 6.

#### Repeat-based alignment element patterns

The presence or absence of 27 elements from the second exon of the repeat-based alignment defined 14 unique patterns (Figure 6), fewer than the 27 patterns identified in the cDNA-based alignment (Figure 2A; Table 3). Element patterns from the repeat-based alignment have been designated with an *r *in front of the pattern name. Some gene sets were unaffected by the alignment changes (*e.g*., *A1 *became *rA1*). Other cDNA-based element patterns were merged to form repeat-based patterns; *E2δ, E3*δ, and *E7δ *were combined in the repeat-based alignment to form gene set *rE2/3/7*δ. The majority of repeat-based patterns were defined by the presence or absence of elements Er3, Er14, Er16, Er17, and Er21 through Er25 (Figure 6). The most common repeat-based pattern was *rD1/6/7 *(38 genes), whereas twelve patterns were defined by single genes. As in the cDNA-based alignment, genes containing either Er10a or Er10g were distinct from the rest of the dataset because they also had Er4, Er7, Er12–16, and Er18–27 (Table 2). Repeat-based element patterns largely correlated with intron type, with the same exceptions as those in the cDNA-based alignment (Figure 6).

#### Element diversity analysis

To understand how modifying the alignment affected sequence diversity, nucleotide and amino acid diversity scores were calculated for the repeat-based elements (Figure 4B) and the overall alignment. The overall alignment nucleotide sequence diversity and average element diversity for the repeat-based alignment were slightly lower than those of the cDNA-based alignment (Table 4). Due to the element subtypes, elements Er10 and Er27b had the highest nucleotide diversity (similar to Ex15 and Ex25; Figure 7). One explanation for the decreased average repeat-based element diversity score was an increased number of conserved elements: five in the repeat-based alignment, compared to one in the cDNA-based alignment (Figure 4).

#### Diversity of the gene sets

In addition to analyzing element diversity, it was of interest to understand diversity across the entire sequence. This analysis, however, was confounded by the gaps required to optimize the alignment. Therefore, we determined the diversity scores for sets of genes with the same exon element pattern and intron type, thereby circumventing the problem introduced by the gaps. Diversity scores were calculated for thirteen sets with three or more members from both alignments, which included or omitted the intron (Table 5). Results indicated that the average diversity of gene sets (0.0214) was significantly lower than the average diversity of all cDNA-based elements from all genes (0.0668), despite the fact that the genes were much longer than individual elements. Nucleotide diversity of gene sets ranged from 0.0044 (pattern *F1*β) to 0.0549 (*rE2/3/7/8/*9δ; Figure 5, Table 5). This lower diversity implied that genes with identical element patterns also tended to have elements with similar sequences. Furthermore, in eight of the 13 sets, the nucleotide diversity for entire genes (exons and intron) was lower than the diversity for the exons without the intron (Table 5). This implied that, for genes with the same exon element pattern, the intron was at least as conserved, if not more so, than the exons. This result was supported by the diversity analysis of the intron sequences. The diversity of all 55 intron type α sequences associated with a variety of different exon element patterns was 0.0261, whereas the diversity for the α intron sequences from just the genes with the *D1*α exon element pattern was about half that (0.0159). This indicated that genes with identical element patterns were more closely related to one another by nucleotide sequence than genes with different element patterns.

**Table 5:** Characteristics of the gene sets in both the cDNA-based and the repeat-based alignment^#^

**Set**	**Freq.**	**Length (nt)**	**Total nt div**	**ORF diversity**			**dn/ds**
					
				**nt**	**aa**	**nt/aa ratio**^§^	
*01δ*	17	1246	0.0151	0.0168	0.0305	1.82	**1.0699**
*r01/8δ*^	18	1246	0.0151	0.0128	0.0267	2.09	**1.0468**
B3α/rB3α*	5	1454	0.0178	0.0181	0.0198	1.09	0.6332
*B3β*	9	1458	0.0243	0.0170	0.0248	1.46	0.6859
*rB3/5β*^	10	1459	0.0267	0.0222	0.0340	1.53	0.6776
C3/rC3α*	4	1481	0.0104	0.0105	0.0161	1.53	0.5533
*D1α*	35	1555	0.0191	0.0333	0.0485	1.46	**1.1154**
*rD1/6/7α*^	38	1555	0.0217	0.0347	0.0534	1.54	**1.1558**
*E2δ*	12	1341	0.0411	0.0244	0.0420	1.72	0.8273
*rE2/3/7/8/9δ*^	26	1342	0.0466	0.0549	0.0766	1.40	0.7969
*E3δ*	11	1339	0.0237	0.0148	0.0329	2.22	**2.4132**
*F1β*	3	1440	0.0035	0.0044	0.0075	1.70	0.3826
*rF1/2β*^	4	1440	0.0129	0.0176	0.0280	1.59	0.6807

^#^Repeat-based elements are denoted by an *r *before the set name.

*Gene sets that were not different between the two alignments.

^Gene sets in the repeat-based alignment that were merged from two or more cDNA-based alignment sets.

^§^A score of 1.5 indicates that changes in half of the nucleotides are nonsynonymous.

To understand the effect of nucleotide substitutions on the encoded protein sequence, gene sets from both alignments were subjected to two parallel lines of investigation: calculations of synonymous vs. non-synonymous (dn/ds) ratios [32], and ratios of nucleotide to amino acid diversity scores (Table 5). Of the 13 sets analyzed, five (*01*δ, *r01*δ/*8, D1*α, *rD1/6/7*α, and *E3*δ) had dn/ds ratios greater than 1, indicating positive (diversifying) selection (shown in bold, Table 5). Two of these sets also had the highest diversity ratios (those with the most non-synonymous substitutions): *E3*δ, and *r01*δ/*8*. The set with least non-synonymous substitutions, *B3*α/*rB3*α, also had a low dn/ds ratio. There was not a strong correlation between the dn/ds values and the diversity ratios because PAML-based dn/ds analysis incorporates an amino acid similarity matrix, whereas the diversity ratios weight all amino acid substitutions equally. The average diversity ratio for the sets was 1.62, indicating that over half (1.62 of every 3) of the nucleotide substitutions resulted in a non-synonymous amino acid substitution. The average set diversity for the repeat-based alignment was slightly higher than that for the cDNA-based alignment (Table 4; Table 5), which may have been due to merging elements to generate the repeat-based alignment. Merging Ex10 and Ex12 increased the diversity score to 0.1197, compared to 0.0807 and 0.0766 when analyzed separately. Understanding the effects of nucleotide diversity on the encoded protein sequences provides some insight into the resulting pool of proteins.

Although it is unusual for a set of sequences to be aligned multiple ways with about equal characteristics, the repeats within the *185/333 *sequences enabled this result. Each of the alignments defined different numbers of elements based on repeats and/or gaps within the sequences, and the presence or absence of those elements defined a series of patterns into which all of the genes could be classified. It was noteworthy that neither alignment proved significantly better at describing the diversity of the gene elements or sets. Due to the high levels of diversity found among the *185/333 *sequences, it is likely that future analyses will uncover additional alignments that will refine our current understanding of these sequences.

### BAC screening and intronless genes

A small BAC library constructed from *S. purpuratus *gDNA [33] was screened to identify clones containing *185/333 *genes. In total, 48 BACs (of 92,160 in the library) were positive for *185/333 *genes. Further analysis of these BACs by PCR using *185/333*-specific primers (185-5'UTR and 185-3'UTR; Table 1) revealed that all 48 amplified at least one, and sometimes up to 10 bands (Figure 1D). We have assumed that each band represents one or more *185/333 *genes in the BAC sequence, most of which were within the typical size range. However, some bands were notably smaller than expected (< 1 kb; Figure 1D). Cloning and sequencing of these bands revealed that these genes did not have an intron (Figure 2B; Additional file 8).

In total, 12 clones were sequenced from two BACs (091G11, n = 8; and 190D12, n = 4), of which five were unique, three contained introns (patterns *D1*α, *01*δ, and *B3*β) and two were intronless (Figure 2B). Two of the sequences were isolated from both BACs, suggesting that the BAC inserts overlap. The two intronless clones had exon patterns *E2 *(named *E2x*) and previously unidentified pattern, *E2.7* (Figure 2B). *E2.7* was unique because it had the same element pattern as *E2*δ, but contained an 88 bp deletion spanning elements Ex2 through Ex3 (Er1 and Er2). This deletion did not correspond with a repeat or an intact element and introduced a frameshift that resulted in missense sequence and a stop codon 23 bp after the deletion. It was therefore possible that *E2.7 *was a pseudogene, perhaps the only one identified from 126 unique genes analyzed.

Additional evidence for the presence of intronless genes was acquired by amplifying genomic DNA using primers that annealed in the 5'UTR (185-5'UTR; Table 1) and in the type 1 repeats of elements Ex4, Ex5, and Ex6 (185-R5; Table 1). A fragment of ~450 bp was amplified from genomic DNA from animal 2, which was too short to include a typical 400 bp intron (Figure 1C, lane 1). This region was similar in size to that amplified from a cDNA clone, *Sp0313 *(DQ183171, [27]) using the same primers (Figure 1C, lane 2). The low frequency of intronless clones within the *185/333 *gene family may account for our inability to detect smaller fragments when the entire genome was used as the template.

### Gene locus structure

**Figure 8:**
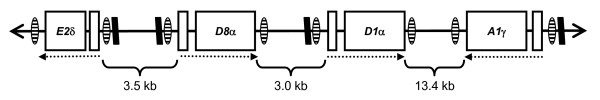
**Structure of part of the *185/333 *locus**. Scaffold_v2_79421 from the *S. purpuratus *genome assembly (Version 2, June 15, 2006) contains four linked *185/333 *genes (diagram not to scale). Each of the genes includes a single intron in the predicted location. The element patterns of the genes are based on the cDNA-based alignment are indicated. Pattern *D8 *was not isolated from the cloned genes; however, it is similar to pattern *D1*, but contains Ex12 rather than Ex10. The orientations of the genes are indicated by the arrows. Dinucleotide (GA; striped ovals) flank the genes and trinucleotide repeats (GAT; solid parallelograms) are present on the 5' side of the genes.

Because multiple *185/333 *genes were present on individual BACs, we used PCR to investigate the size of intergenic regions. Amplification of genomic DNA from four animals using primers 185-F5 and 185-LR1 (Table 1) showed that the major intergenic size was ~3 kb (Figure 1B). Bands of various sizes, ranging from about 1.6 kb to 8 kb, indicated that spacing between the genes was variable among the four animals analyzed. Given the constraints of PCR, it was possible that some genes were too far apart for successful intergenic amplification. A representative genomic scaffold from the sea urchin genome (assembly v. 2, 6/15/06) contained four genes spaced 3, 3.5 and 13.4 kb apart (Figure 8). Intergenic sequence from the contigs revealed that the genes were flanked on the both sides by a stretch of ~15 dinucleotide (GA) repeats, and also on the 5' side by trinucleotide repeats. Although the significance of these repeats is unknown, preliminary cloning and sequencing of the region upstream of 17 *185/333 *genes indicates that the dinucleotide repeats are commonly present (Kim and Smith, unpublished).

## Discussion

Previous reports described the sequences and expression patterns of the *185/333 *transcripts [27,29,30]. The data presented here detail the structure and diversity of the *185/333 *genes and suggest that they compose a large family of linked genes within the *S. purpuratus *genome. The typical gene structure has two exons separated by a single intron, although genes lacking introns are also present in the genome. Based on repeats within the genes, multiple alignments of the *185/333 *sequences are possible. The two alignments utilized in this study define elements based on large gaps employed for one of two reasons: to align the transcript sequences [27,29,30], or to optimize the repeats. The presence/absence of the elements defines element patterns (33 patterns in the cDNA-based alignment and 17 patterns in the repeat-based alignment). The high level of diversity of the genes is exemplified by the 121 distinct genes among the 171 that were cloned and sequenced. Diversity is distributed throughout the sequence, and varies among the elements. The 121 unique gene sequences encode 101 unique amino acid sequences, indicating that the genetic diversity extends to the encoded proteins. Half of the gene sets, composed of unique genes with identical element patterns, are under positive selection according to dn/ds analysis. The *185/333 *genes (100 ± 20 alleles [30]) are typically spaced about 3 kb apart, and are flanked by short repeats. The *185/333 *gene family is therefore composed of a suite of closely linked, highly diverse genes.

### Gene diversity

Although initial reports of the *185/333 *sequences suggested that their diversity may be the result of putative alternative splicing [27], the data presented here support the previous conclusion [30] that the element pattern diversity observed among the *185/333 *transcripts is not the result of alternative splicing, but resides within the second exon. With one exception (element 11a), all of the elements found in the message sequences were also identified among the genes, and no new elements were found. Other than the single intron located directly after the leader sequence, no additional introns were identified. However, larger amplicons (~4 kb) were observed when genomic DNA was amplified with primers flanking the genes (Figure 1A). Although nothing is known about the sequence of these genes, there are three possible explanations for these fragments: (1) genes with duplications of currently known elements, or additional elements that are currently unknown; (2) genes in which the primer annealing sites were mutated or missing, causing the amplification to skip over genes; (3) genes with larger or more introns. Analysis of the *185/333 *locus in which many of the genes reside will be required to understand the PCR results. However, based on the frequency of genes in which the second exon encodes the majority of the open reading frame, it is unlikely that alternative splicing is the primary mechanism to generate the diverse array of element patterns observed among the transcript sequences.

In a previous study [29], half of the expressed transcripts contained an early stop codon which, if translated, would encode missense or truncated proteins. It is curious that, given the frequency of these messages and the number of genes cloned and sequenced, with one exception (pattern *E2.7*), no early stop codons or frameshifts were found in any of the gene sequences. Element pattern *E2.1*, which has a SNP generated stop codon in Ex10 and constitutes 65% of the *185/333 *messages in coelomocytes prior to challenge and 33% of *185/333 *messages after challenge with various antigens, was not found among the gene sequences, despite the fact that animals 2 and 4, from which *E2.1 *transcripts were isolated, were also the source of gDNA in this study. It is possible that an *E2.1 *gene is present within the genome of these two animals and was simply not cloned. Alternatively, the *185/333 *messages may be post-transcriptionally modified through as yet unknown mechanisms that introduce SNPs into the transcripts, which cause frameshifts or early stop codons. This discord between gene and message sequences from individual animals is a future area of investigation.

In addition to analyzing the diversity of the *185/333 *gene sequences, it is also of interest to understand those aspects of the genes which are conserved. Element sequences are conserved in that there are very few variations found in single sequences, and each element has relatively few unique sequences. Because there are so few unshared variations, each exon element (L – Ex25) consists of, on average, 11 unique sequences. Given the 121 unique total gene sequences, this number is surprisingly low. Elements are commonly associated with one another, and element patterns are largely defined by element Ex15 (or Er10) subtypes, which are commonly associated with suites of elements [30]. Furthermore, diversity data on the sets suggests that genes with identical element patterns tend to have more similar nucleotide sequences. Perhaps genes commonly share suites of elements due to their relative proximity on the chromosome and frequent recombination. The genes may be arranged within the *185/333 *locus in smaller gene subfamilies, according to Ex15 subtype or element pattern. Further sequencing and analysis of this locus will be required to confirm this hypothesis.

On the whole, the intron sequences of the *185/333 *gene family are more diverse than the exon sequences, primarily as a result of an increased average number of variable positions within the elements. This may be due to decreased selective pressure to maintain this non-coding sequence. Although diversity is present throughout the intron sequence, small conserved regions exist that may serve as transcription factor binding sites or regulatory sequences. Preliminary evidence suggests that there may be AP-1, dorsal and GATA binding sites within the intron (unpublished data), all of which have been shown to upregulate genes involved with immune functions [34-36]. Although most of the exon element patterns are associated with a specific intron type, there are examples when the two do not correlate. If the intron serves to regulate gene expression or has some other regulatory function, altering the intron type may affect the transcription of the exon element pattern.

Using three animals in this study provided preliminary insight into the population-level diversity of the *185/333 *gene family. Among the 121 unique sequences isolated from the three animals, no identical gene sequence was shared among two or more animals. This diversity is also illustrated from the 872 sequences (both messages and genes) collected from 16 different animals that contain 477 unique open reading frames that form 51 exon element patterns and encode 323 unique protein sequences [29,30]. The 14 new exon element patterns identified in this study may represent genes that are not commonly expressed, or genes that are not expressed in response to challenge with marine bacteria, lipopolysaccharide (LPS), double-stranded RNA (dsRNA), or β-1,3-glucan; [29,30]). Although the function of the 185/333 proteins is unknown, the sequence diversity, size of the gene family, and expression patterns all suggest a role in the defense response of *S. purpuratus*. An immune-related function would help explain this high level of inter-animal diversity, because in the arms race against pathogens, it is important to maintain a polymorphic population of immune-related genes within the host to allow survival in the presence of new pathogens.

Compared to other sea urchin genes, the *185/333 *genes are atypical. Among the 23,300 predicted gene models in the sea urchin genome, the average gene has 8.3 exons and spans 7.7 kb [37]. Furthermore, although the *185/333 *intron is smaller than average (~400 bp compared to the average size of 750 bp), the second exon length is significantly longer (average exon length is 100–115 nt) [37]. It has been suggested that highly expressed genes are under selection to maintain short introns, due to the cost of transcribing and splicing long introns [38]. Although the *185/333 *introns are not much shorter than average, in *C. elegans*, the difference in average intron length between highly expressed genes and those expressed at low levels was only 2-fold. At the extreme, histone genes, which are highly expressed in replicating cells lack introns altogether [39]. The *185/333 *genes are strongly upregulated in response to bacterial challenge, and represent ~65% of the transcripts in immune activated coelomocytes [30]. This high level of expression may be the selective pressure for maintaining short intron sequences.

### Locus structure

Analysis of the *S. purpuratus *genome assembly and genomic DNA by PCR amplification indicates that the *185/333 *gene locus consists of tandem arrays of closely linked genes. Genomic DNA amplification using a variety of primers suggests that genes are oriented head to tail, head to head, and tail to tail (unpublished data). Given the high sequence similarity (>88% identical), among the genes and their close linkage, the several types of repeats located within the genes, and the small repeats located between the genes, each gene and its immediate flanking region might be considered as a large repeat. The sequences flanking the genes through the short repeat regions are moderately conserved, and this conservation is lost beyond the dinucleotide repeats (unpublished data). Therefore, it is possible that the *185/333 *genes are arranged as cassettes defined on either side by dinucleotide repeats, which may facilitate diversification events, such as meiotic mispairing, gene conversion, recombination, or duplication and/or deletion of intact genes with the cis promoter. Dinucleotide repeats have been associated with genomic instability, which increases population diversity [40] and recombination frequency [41]. Although dinucleotide repeats are also common in the *S. purpuratus *genome (preliminary estimates of about 49,000 in the genome [33]), nothing is known about the relative locations of repeats vs. open reading frames. The putative *185/333 *gene locus structure therefore appears to be optimal for numerous diversification strategies as would be predicted for genes that encode proteins with important immune functions.

## Conclusion

Analysis of the *185/333 *gene family reveals that the diversity observed within the transcript repertoire is not the result of alternative splicing, but rather, is encoded within the second exon of the genes. These genes exhibit variability in both element patterns and sequence variations, and preliminary data indicates that they encode a diverse family of proteins (unpublished). The repeats within and surrounding the genes, as well as their close linkage create a locus that is optimal to promote diversification events such as recombination and/or gene conversion. Therefore, the *185/333 *gene family represents an example of invertebrate innate immunity that is more complex than previously believed. These results add to the growing body of evidence suggesting that all organisms, including vertebrates, invertebrates, and plants maintain highly diverse innate immune systems, and that diversification may be driven by pathogen pressure.

## Methods

### Animals

Sea urchins were collected by Marinus Scientific, Inc. (Long Beach, CA) by SCUBA divers from near-shore, sub-tidal regions off the coast of southern California and were housed as previously described [17].

### Genomic DNA isolation

Genomic DNA (gDNA) was isolated from coelomocytes collected from two sea urchins or from sperm from a single individual as previously described [30,42].

### BAC library screens and BAC plasmid isolation

Filters from an arrayed BAC library were screened for *185/333 *positive clones using riboprobes. The library was constructed using the pBAC3.6 vector with insert sizes between 50 and 60 kb [33]. Three cDNA clones [*Sp0032 *(DQ183167), *Sp0164 *(DQ183149), and *Sp0313 *(DQ183171)], with element patterns *A1*, *D1*, and *C1*, respectively [27,30] were selected to synthesize a mixture of riboprobes because they collectively contained all known elements. Riboprobes were generated by incorporating ^32^P-rUTP and filters were screened according to [43]. Positive clones were received from Eric Davidson and Andrew Cameron at the California Institute of Technology.

Overnight cultures of BAC clones were grown in 2xYT containing 12.5 μg/mL chloramphenicol. Cells were pelleted by centrifugation for 2 minutes at 12,500 × *g *and resuspended in 300 μL buffer P1 (15 mM Tris, pH = 8; 10 mM EDTA; 100 μg/mL RNAseA). Buffer P2 (300 μL; 0.2 N NaOH; 1% SDS), and buffer P3 (300 μL; 3 M potassium acetate, pH = 5) were added, and the mixture was kept on ice for 5 minutes. Samples were spun at 12,500 × *g *for 10 minutes at 4°C and the supernatants were precipitated by the addition of an equal volume of isopropanol and resuspended in Tris-EDTA (TE; 10 mM Tris, pH = 8; 1 mM EDTA).

### Gene amplification, intergenic PCR, and cloning

The template for polymerase chain reaction (PCR) was either 100 ng of gDNA, 50 ng of BAC DNA, or 10 ng of a cloned gene. Genes cloned from animals 2 and 10 were amplified in PCR reactions consisting of 1 μM of each primer (Table 1), 150 μM of each deoxynucleotide, 2 mM MgCl_2_, and either 0.5 U *Optimase *DNA Polymerase (Transgenomic, Inc.) or 0.5 U *Ex Taq *DNA Polymerase (Takara Mirius Bio., Madison, WI) and 1X of the appropriate company-supplied buffer. Both amplifications used the following program: 94°C for 3 minutes followed by 25 cycles of 94°C for 30 seconds, 61°C for 30 seconds, and 72°C for 1 minute, with a final extension of 72°C for 5 minutes and a 4°C hold. Full-length genes were amplified by PCR using the primer pair 185-3'UTR and 185-5'UTR (Table 1). To analyze the 5' half of the cloned genes (leader through Ex7) and to identify those without introns, clones were amplified with primers 185-5'UTR and 185-R6 (Table 1).

To amplify intergenic regions from BAC or gDNA, reactions consisted of 400 nM each primer (LR1 and F5; Table 1), 400 μM of each deoxynucleotide, 2.5 mM MgCl_2_, 1X company supplied buffer (Takara Mirius Bio), and 0.5 U *Takara LA Taq *(Takara Mirius Bio). The amplification program was: 94°C for 2 minutes followed by 25 cycles of 94°C for 20 seconds, and 61°C for 9 minutes, with a final extension of 72°C for 10 minutes and a 4°C hold.

Amplified *185/333 *genes were cloned into the pCR4-TOPO vector using the Topo-TA Cloning Kit for Sequencing according to the manufacturer (Invitrogen, Carlsbad, CA). Plasmid DNA was isolated using the Wizard Plus Miniprep DNA Purification System (Promega, Madison, WI). Gene sequences were submitted to Genbank with accession numbers EF607618–EF607793.

### Cycle sequencing

DNA sequencing was done using the GenomeLab Dye Terminator Cycle Sequencing with Quick Start Kit according to the manufacturer (Beckman Coulter, Fullerton, CA) and analyzed on a Beckman CEQ 8000 DNA sequencer (Beckman Coulter).

### Bioinformatics

Sequences were manually aligned using Bioedit [44]. PERL scripts were written to identify identical sequences and to calculate diversity characteristics (Additional files 9, 10, 11, 12). Sequence diversity was calculated as in [30]. Nonsynonymous/synonymous (dn/ds) ratios were calculated using Phylogenetic Analysis by Maximum Likelihood (PAML) [32]. Neighbor-joining trees for PAML were created using unique sequences with Molecular Evolutionary Genetics Analysis (MEGA), v. 3.1 [45]. Phylogenetic analysis of the intron was done using Phylip [46]. Maximum likelihood and maximum parsimony trees were generated, and bootstrap values were determined based on 500 replications.

## Authors' contributions

KMB was responsible for the PCR, cloning, sequencing, sequence analysis, bioinformatics analysis of all the sequences and prepared the manuscript. LCS directed the research, assisted in data analysis, edited the manuscript and provided funding. Both authors read and approved the final manuscript.

## Supplementary Material

Additional file 1 Characteristics of the cloned *185/333 *genes. A table describing all of the genes cloned and sequences, their accession numbers, size, element pattern and intron type. Click here for file

Additional file 2 cDNA-based nucleotide alignment of the *185/333 *genes. The cDNA-based nucleotide alignment of the complete 121 unique gene sequences showing the element borders and repeats. Click here for file

Additional file 3 cDNA-based amino acid alignment of the *185/333 *genes. The cDNA-based amino acid alignment of the open reading frames of the 121 unique gene sequences showing the element borders and repeats. Click here for file

Additional file 4 Characteristics of the elements. The lengths, frequencies, and diversity characteristics of the elements from both the cDNA-based and repeat-based alignments. Click here for file

Additional file 5 Diversity controls. A description of the control experiments performed to eliminate the possibility that the observed *185/333 *gene diversity was the result of either artificial *Taq-*induced errors or template switching. Click here for file

Additional file 6 Repeat-based nucleotide alignment of the *185/333 *genes. The repeat-based alignment of the second exon of the 121 unique gene sequences showing the element borders and repeats. Click here for file

Additional file 7 Repeat-based amino acid alignment of the *185/333 *genes. The repeat-based alignment of the deduced protein sequence of the second exons of the 121 unique gene sequences showing the element borders and repeats. Click here for file

Additional file 8 Alignment of the *185/333 *genes isolated from two BACs. The nucleotide and amino acid sequence of the five unique *185/333 *genes isolated from two separate BACs, aligned with the cDNA-based alignment. Click here for file

Additional file 9 Sametool.pl: A PERL script for determining identical sequences. A PERL script that reads a directory of files of aligned nucleotide or amino acid sequences in FASTA format, determines the number of unique sequences, and identifies groups of identical sequences. Click here for file

Additional file 10 Entropy.pl: A PERL script to calculate sequence diversity. A PERL script that reads a directory of files of aligned nucleotide or amino acid sequences in FASTA format and calculates the entropy of the alignment in each file. Click here for file

Additional file 11 Variablepos.pl: A PERL script that calculates the number of variable positions. A PERL script that reads a directory of files of aligned nucleotide or amino acid files and determines the number and percent of variable positions within the alignment. Click here for file

Additional file 12 Statespervar.pl: A PERL script that calculates the number of states in each variable alignment position. A PERL script that reads a directory of files of aligned nucleotide or amino acid sequences and determines the average number of states in each variable position in the alignment. Click here for file
