# Supplementary material for: Extraordinary diversity among members of the large gene family, 185/333, from the purple sea urchin, Strongylocentrotus purpuratus
Source: BMC Mol Biol. 2007 Aug 15;8:68. doi: 10.1186/1471-2199-8-68 (PMC1988830; doi:10.1186/1471-2199-8-68)
Supplement: Additional file 1 — Characteristics of the cloned 185/333 genes. A table describing all of the genes cloned and sequences, their accession numbers, size, element pattern and intron type. [file 1471-2199-8-68-S1.pdf]

Additional File 1: Characteristics of the 171 cloned and sequenced genes. The clone number, source animal, Genbank accession number and size are shown, in addition to the element pattern for both the cDNA-based and repeat-based alignments, intron type, Ex15/Er10 element subtype, and location of the stop codon.

Animal	Sequence	Genbank Acc. #	Gene (Nt)	Intron (Nt)	ORF (Nt)	ORF (aa)	cDNA Pattern	RBA Pattern	Intron Type	10/15 Subtype	25 stop
2	2-002	EF607673	1542	411	1131	377	*D1*	*rD1/6/7*	α	d	b
2	2-005	EF607674	1544	413	1131	377	*D7*	*rD1/6/7*	α	d	c
2	2-006	EF607675	1440	414	1026	342	*F1*	*rF1/2*	β	f	b
2	2-007	EF607676	1542	411	1131	377	*D1*	*rD1/6/7*	α	d	b
2	2-008	EF607677	1542	411	1131	377	*D1*	*rD1/6/7*	α	d	b
2	2-009	EF607678	1440	414	1026	342	*F1*	*rF1/2*	β	f	b
2	2-010	EF607679	1440	414	1026	342	*F1*	*rF1/2*	β	f	b
2	2-011	EF607688	1333	406	927	309	*E3δ*	*rE2/3δ/7*	δ	e	a
2	2-012	EF607697	1318	406	912	304	*E2*	*rE2/3δ/7*	δ	e	a
2	2-013	EF607698	1544	413	1131	377	*D1*	*rD1/6/7*	α	d	b
2	2-015	EF607699	1335	405	930	310	*E8*	*rE2/3δ/7*	δ	e	b
2	2-017	EF607700	1338	411	927	309	*E3α*	*rE3α*	α	e	a
2	2-018	EF607701	1543	412	1131	377	*D1*	*rD1/6/7*	α	d	b
2	2-019	EF607702	1318	406	912	304	*E2*	*rE2/3δ/7*	δ	e	a
2	2-020	EF607703	1318	406	912	304	*E2*	*rE2/3δ/7*	δ	e	a
2	2-021	EF607704	1542	411	1131	377	*D1*	*rD1/6/7*	α	d	b
2	2-022	EF607705	1542	411	1131	377	*D1*	*rD1/6/7*	α	d	b
2	2-023	EF607706	1542	411	1131	377	*D1*	*rD1/6/7*	α	d	b
2	2-024	EF607707	1544	413	1131	377	*D1*	*rD1/6/7*	α	d	b
2	2-025	EF607708	1318	406	912	304	*E2*	*rE2/3δ/7*	δ	e	a
2	2-026	EF607709	1544	413	1131	377	*D1*	*rD1/6/7*	α	d	b
2	2-027	EF607710	1542	411	1131	377	*D1*	*rD1/6/7*	α	d	b
2	2-028	EF607711	1542	411	1131	377	*D1*	*rD1/6/7*	α	d	b
2	2-029	EF607712	1335	405	930	310	*E2*	*rE2/3δ/7*	δ	e	a
2	2-031	EF607713	1335	405	930	310	*E8*	*rE2/3δ/7*	δ	e	b
2	2-032	EF607714	1543	412	1131	377	*D1*	*rD1/6/7*	α	d	b
2	2-033	EF607715	1318	406	912	304	*E2*	*rE2/3δ/7*	δ	e	a
2	2-034	EF607716	1894	412	1482	494	*A1*	*rA1*	γ	a	b
2	2-035	EF607717	1333	406	927	309	*E3δ*	*rE2/3δ/7*	δ	e	a
2	2-036	EF607718	1894	412	1482	494	*A1*	*rA1*	γ	a	b

Animal	Sequence	Genbank Acc. #	Gene (Nt)	Intron (Nt)	ORF (Nt)	ORF (aa)	cDNA Pattern	RBA Pattern	Intron Type	10/15 Subtype	25 stop
2	2-037	EF607719	1333	406	927	309	*E3δ*	*rE2/3δ/7*	δ	e	a
2	2-038	EF607720	1542	411	1131	377	*D1*	*rD1/6/7*	α	d	b
2	2-039	EF607721	1542	411	1131	377	*D1*	*rD1/6/7*	α	d	b
2	2-040	EF607722	1440	414	1026	342	*F1*	*rF1/2*	β	f	b
2	2-041	EF607723	1231	406	825	275	*01δ*	*r01δ/2*	δ	0	b
2	2-047	EF607724	1333	406	927	309	*E3δ*	*rE2/3δ/7*	δ	e	a
2	2-048	EF607725	1542	411	1131	377	*D1*	*rD1/6/7*	α	d	b
2	2-049	EF607726	1228	406	822	274	*01δ*	*r01δ/8*	δ	0	b
2	2-050	EF607727	1228	406	822	274	*08*	*r01δ/8*	δ	0	a
2	2-052	EF607728	1544	413	1131	377	*D1*	*rD1/6/7*	α	d	b
2	2-055	EF607729	1433	407	1026	342	*B3α*	*rB3α*	α	b	b
2	2-056	EF607730	1541	410	1131	377	*D1*	*rD1/6/7*	α	d	b
2	2-057	EF607731	1543	412	1131	377	*D1*	*rD1/6/7*	α	d	b
2	2-059	EF607732	1231	406	825	275	*01δ*	*r01δ/2*	δ	0	b
2	2-061	EF607733	1398	411	987	329	*E6*	*rE6*	α	e	a
2	2-063	EF607734	1335	405	930	310	*E2*	*rE2/3δ/7*	δ	e	a
2	2-064	EF607735	1542	411	1131	377	*D1*	*rD1/6/7*	α	d	b
2	2-065	EF607736	1246	406	840	280	*01δ*	*r01δ/2*	δ	0	b
2	2-066	EF607737	1434	414	1020	340	*F2*	*rF1/2*	β	f	b
2	2-067	EF607738	1333	406	927	309	*E3δ*	*rE2/3δ/7*	δ	e	a
2	2-069	EF607739	1231	406	825	275	*01δ*	*r01δ/2*	δ	0	b
2	2-070	EF607740	1543	412	1131	377	*D1*	*rD1/6/7*	α	d	b
2	2-071	EF607741	1318	406	912	304	*E2*	*rE2/3δ/7*	δ	e	a
2	2-073	EF607742	1327	406	921	307	*E7*	*rE2/3δ/7*	δ	e	b
2	2-076	EF607743	1543	412	1131	377	*D1*	*rD1/6/7*	α	d	b
2	2-077	EF607744	1542	411	1131	377	*D1*	*rD1/6/7*	α	d	b
2	2-079	EF607745	1542	411	1131	377	*D1*	*rD1/6/7*	α	d	b
2	2-080	EF607746	1544	413	1131	377	*D1*	*rD1/6/7*	α	d	b
2	2-081	EF607747	1440	414	1026	342	*F1*	*rF1/2*	β	f	b
2	2-082	EF607748	1543	412	1131	377	*D1*	*rD1/6/7*	α	d	b
2	2-083	EF607749	1231	406	825	275	*01δ*	*r01δ/2*	δ	0	b

Animal	Sequence	Genbank Acc. #	Gene (Nt)	Intron (Nt)	ORF (Nt)	ORF (aa)	cDNA Pattern	RBA Pattern	Intron Type	10/15 Subtype	25 stop
2	2-084	EF607750	1231	406	825	275	*01δ*	*r01δ/2*	δ	0	b
2	2-089	EF607751	1318	406	912	304	*E2*	*rE2/3δ/7*	δ	e	a
2	2-090	EF607752	1335	405	930	310	*E9*	*rE2/3δ/7*	δ	e	c
2	2-091	EF607753	1329	405	924	308	*E9*	*rE2/3δ/7*	δ	e	c
2	2-093	EF607754	1440	414	1026	342	*F1*	*rF1/2*	β	f	b
2	2-094	EF607755	1335	405	930	310	*E2*	*rE2/3δ/7*	δ	e	a
2	2-095	EF607756	1321	406	915	305	*E2*	*rE2/3δ/7*	δ	e	a
2	2-096	EF607757	1440	414	1026	342	*F1*	*rF1/2*	β	f	b
2	2-098	EF607758	1544	413	1131	377	*D1*	*rD1/6/7*	α	d	b
2	2-099	EF607759	1318	406	912	304	*E2*	*rE2/3δ/7*	δ	e	a
2	2-101	EF607680	1541	410	1131	377	*D1*	*rD1/6/7*	α	d	b
2	2-102	EF607681	1542	411	1131	377	*D1*	*rD1/6/7*	α	d	b
2	2-103	EF607682	1440	414	1026	342	*F1*	*rF1/2*	β	f	b
2	2-104	EF607683	1433	407	1026	342	*B3α*	*rB3α*	α	b	b
2	2-105	EF607684	1335	405	930	310	*E2*	*rE2/3δ/7*	δ	e	a
2	2-106	EF607685	1543	412	1131	377	*D1*	*rD1/6/7*	α	d	b
2	2-107	EF607686	1334	407	927	309	*E3δ*	*rE2/3δ/7*	δ	e	a
2	2-109	EF607687	1545	411	1134	378	*D1*	*rD1/6/7*	α	d	b
2	2-110	EF607689	1440	414	1026	342	*F1*	*rF1/2*	β	f	b
2	2-111	EF607690	1542	411	1131	377	*D1*	*rD1/6/7*	α	d	b
2	2-112	EF607691	1335	405	930	310	*E2*	*rE2/3δ/7*	δ	e	a
2	2-114	EF607692	1318	406	912	304	*E2*	*rE2/3δ/7*	δ	e	a
2	2-115	EF607693	1543	412	1131	377	*D1*	*rD1/6/7*	α	d	b
2	2-116	EF607694	1231	406	825	275	*01δ*	*r01δ/8*	δ	0	b
2	2-118	EF607695	1318	406	912	304	*E2*	*rE2/3δ/7*	δ	e	a
2	2-119	EF607696	1318	406	912	304	*E2*	*rE2/3δ/7*	δ	e	a
4	4-1501	EF607760	1541	410	1131	377	*D1*	*rD1/6/7*	α	d	b
4	4-1503	EF607761	1227	405	822	274	*01δ*	*r01δ/2*	δ	0	b
4	4-1508	EF607762	1227	405	822	274	*01δ*	*r01δ/2*	δ	0	b
4	4-1511	EF607763	1550	410	1140	380	*D6*	*rD1/6/7*	α	d	b
4	4-1513	EF607764	1227	405	822	274	*01δ*	*r01δ/8*	δ	0	b

Animal	Sequence	Genbank Acc. #	Gene (Nt)	Intron (Nt)	ORF (Nt)	ORF (aa)	cDNA Pattern	RBA Pattern	Intron Type	10/15 Subtype	25 stop
4	4-1515	EF607765	1547	410	1137	379	*D1*	*rD1/6/7*	α	d	b
4	4-1516	EF607766	1481	410	1071	357	*C3*	*rC3*	α	c	b
4	4-1517	EF607767	1227	405	822	274	*01δ*	*r01δ/8*	δ	0	b
4	4-1518	EF607768	1227	405	822	274	*01δ*	*r01δ/8*	δ	0	b
4	4-1520	EF607769	1333	406	927	309	*E3δ*	*rE2/3δ/7*	δ	e	a
4	4-1521	EF607770	1458	414	1044	348	*B3β*	*rB3β*	β	b	b
4	4-1523	EF607771	1550	410	1140	380	*D1*	*rD1/6/7*	α	d	b
4	4-1527	EF607772	1227	405	822	274	*01δ*	*r01δ/8*	δ	0	b
4	4-1528	EF607773	1414	409	1005	335	*E10*	*rE10*	γ	e	a
4	4-1530	EF607774	1545	411	1134	378	*D1*	*rD1/6/7*	α	d	b
4	4-1531	EF607775	1333	406	927	309	*E3δ*	*rE2/3δ/7*	δ	e	a
4	4-1532	EF607776	1334	407	927	309	*E3δ*	*rE2/3δ/7*	δ	e	a
4	4-1533	EF607777	1333	406	927	309	*E3δ*	*rE2/3δ/7*	δ	e	a
4	4-1534	EF607778	1458	414	1044	348	*B3β*	*rB3β*	β	b	b
4	4-1535	EF607779	1227	405	822	274	*01δ*	*r01δ/2*	δ	0	b
4	4-1536	EF607780	1310	410	900	300	*C5*	*rC5*	α	c	b
4	4-1538	EF607781	1551	411	1140	380	*D1*	*rD1/6/7*	α	d	b
4	4-1541	EF607782	1541	410	1131	377	*D1*	*rD1/6/7*	α	d	b
4	4-1542	EF607783	1333	406	927	309	*E3δ*	*rE2/3δ/7*	δ	e	a
4	4-1543	EF607784	1544	410	1134	378	*D1*	*rD1/6/7*	α	d	b
4	4-1544	EF607785	1481	410	1071	357	*C3*	*rC3*	α	c	b
4	4-1545	EF607786	1556	410	1146	382	*C2*	*rC2*	α	c	b
4	4-1547	EF607787	1227	405	822	274	*01δ*	*r01δ/8*	δ	0	b
4	4-1548	EF607788	1458	414	1044	348	*B3β*	*rB3β*	β	b	b
4	4-1550	EF607789	1336	406	930	310	*E2*	*rE2/3δ/7*	δ	e	a
4	4-2410	EF607790	1542	411	1131	377	*D1*	*rD1/6/7*	α	d	b
4	4-2412	EF607791	1541	410	1131	377	*D1*	*rD1/6/7*	α	d	b
4	4-2415	EF607792	1458	414	1044	348	*B3β*	*rB3β*	β	b	b
4	4-2449	EF607793	1541	410	1131	377	*D1*	*rD1/6/7*	α	d	b
10	10-001	EF607621	1446	402	1044	348	*B3β*	*rB3β*	β	b	b
10	10-002	EF607622	1446	402	1044	348	*B3β*	*rB3β*	β	b	b

Animal	Sequence	Genbank Acc. #	Gene (Nt)	Intron (Nt)	ORF (Nt)	ORF (aa)	cDNA Pattern	RBA Pattern	Intron Type	10/15 Subtype	25 stop
10	10-003	EF607623	1228	406	822	274	*01δ*	*r01δ/2*	δ	0	b
10	10-004	EF607624	1446	387	1059	353	*D5*	*rD5*	ε	d	b
10	10-005	EF607625	1446	387	1059	353	*D5*	*rD5*	ε	d	b
10	10-006	EF607626	1541	410	1131	377	*D1*	*rD1/6/7*	α	d	b
10	10-007	EF607627	1329	387	942	314	*B6*	*rB6*	ε	b	b
10	10-008	EF607628	1337	407	930	310	*E2*	*rE2/3δ/7*	δ	e	a
10	10-010	EF607629	1891	409	1482	494	*G2*	*rG2*	γ	g	b
10	10-011	EF607630	1440	402	1038	346	*B5*	*rB3β*	β	b	b
10	10-012	EF607631	1446	402	1044	348	*B3β*	*rB3β*	β	b	b
10	10-013	EF607632	1474	409	1065	355	*G3*	*rG3*	γ	g	b
10	10-014	EF607633	1541	410	1131	377	*D1*	*rD1/6/7*	α	d	b
10	10-015	EF607634	1540	409	1131	377	*D1*	*rD1/6/7*	α	d	b
10	10-017	EF607635	1448	407	1041	347	*B3α*	*rB3α*	α	b	b
10	10-018	EF607636	1329	387	942	314	*B6*	*rB6*	ε	b	b
10	10-019	EF607637	1519	409	1110	370	*B2*	*rB2*	α	b	b
10	10-020	EF607638	1337	407	930	310	*E2*	*rE2/3δ/7*	δ	e	a
10	10-021	EF607639	1541	410	1131	377	*D1*	*rD1/6/7*	α	d	b
10	10-022	EF607640	1541	410	1131	377	*D1*	*rD1/6/7*	α	d	b
10	10-024	EF607641	1546	409	1137	379	*D6*	*rD1/6/7*	α	d	b
10	10-025	EF607642	1228	406	822	274	*01ε*	*r01ε*	δ	0	b
10	10-026	EF607643	1337	407	930	310	*E2*	*rE2/3δ/7*	δ	e	a
10	10-027	EF607644	1337	407	930	310	*E2*	*rE2/3δ/7*	δ	e	a
10	10-028	EF607645	1206	381	825	275	*01ε*	*r01ε*	ε	0	b
10	10-029	EF607646	1228	406	822	274	*01δ*	*r01δ/8*	δ	0	b
10	10-030	EF607647	1228	406	822	274	*01δ*	*r01δ/8*	δ	0	b
10	10-031	EF607648	1451	407	1044	348	*B3α*	*rB3α*	α	b	b
10	10-032	EF607649	1228	406	822	274	*01δ*	*r01δ/8*	δ	0	b
10	10-034	EF607650	1228	406	822	274	*01δ*	*r01δ/8*	δ	0	b
10	10-035	EF607651	1478	410	1068	356	*C3*	*rC3*	α	c	b
10	10-037	EF607652	1227	405	822	274	*01δ*	*r01δ/8*	δ	0	b
10	10-038	EF607653	1228	406	822	274	*01δ*	*r01δ/8*	δ	0	b

Animal	Sequence	Genbank Acc. #	Gene (Nt)	Intron (Nt)	ORF (Nt)	ORF (aa)	cDNA Pattern	RBA Pattern	Intron Type	10/15 Subtype	25 stop
10	10-039	EF607654	1446	402	1044	348	*B3β*	*rB3β*	β	b	b
10	10-040	EF607655	1433	407	1026	342	*B3α*	*rB3α*	α	b	b
10	10-042	EF607656	1446	402	1044	348	*B8*	*rB3β*	β	b	c
10	10-043	EF607657	1446	402	1044	348	*B8*	*rB3β*	β	b	c
10	10-045	EF607658	1541	410	1131	377	*D1*	*rD1/6/7*	α	d	b
10	10-046	EF607659	1228	406	822	274	*01δ*	*r01δ/8*	δ	0	b
10	10-048	EF607660	1228	406	822	274	*01δ*	*r01δ/8*	δ	0	b
10	10-049	EF607661	1541	410	1131	377	*D1*	*rD1/6/7*	α	d	b
10	10-050	EF607662	1478	410	1068	356	*C3*	*rC3*	α	c	b
10	10-051	EF607663	1451	410	1041	347	*B3α*	*rB3α*	α	b	b
10	10-052	EF607664	1232	410	822	274	*01α*	*r01α*	α	0	b
10	10-053	EF607665	1228	406	822	274	*01δ*	*r01δ/8*	δ	0	b
10	10-054	EF607666	1349	410	939	313	*B7*	*rB7*	α	b	b
10	10-055	EF607667	1547	410	1137	379	*D1*	*rD1/6/7*	α	d	b
10	10-056	EF607668	1541	410	1131	377	*D1*	*rD1/6/7*	α	d	b
10	10-057	EF607669	1541	410	1131	377	*D1*	*rD1/6/7*	α	d	b
10	10-058	EF607670	1228	406	822	274	*01δ*	*r01δ/8*	δ	0	b
