# Supplementary material for: Extraordinary diversity among members of the large gene family, 185/333, from the purple sea urchin, Strongylocentrotus purpuratus
Source: BMC Mol Biol. 2007 Aug 15;8:68. doi: 10.1186/1471-2199-8-68 (PMC1988830; doi:10.1186/1471-2199-8-68)
Supplement: Additional file 2 — cDNA-based nucleotide alignment of the 185/333 genes. The cDNA-based nucleotide alignment of the complete 121 unique gene sequences showing the element borders and repeats. [file 1471-2199-8-68-S2.pdf]

Additional File 2: cDNA-based nucleotide alignment of the gene sequences. Element borders are indicated by vertical black lines, and are labeled above the sequence. Repeats are indicated by shaded boxes, which each different color (red = type 1; blue = type 2; green = type 3; yellow = type 4; and purple = type 5). Stop codons are shown in red boxes. The conserved splice signals are indicated by blue boxes. Conserved stretches in the alignment (minimum of 8 nt long, with a maximum average entropy of 0.02 and maximum entropy per position of 0.2) are indicated by green boxes above the alignment. Sites that are polymorphic in five of the eight sets are shown by red arrows; positions polymorphic in four of the eight sets are indicated by orange arrows.

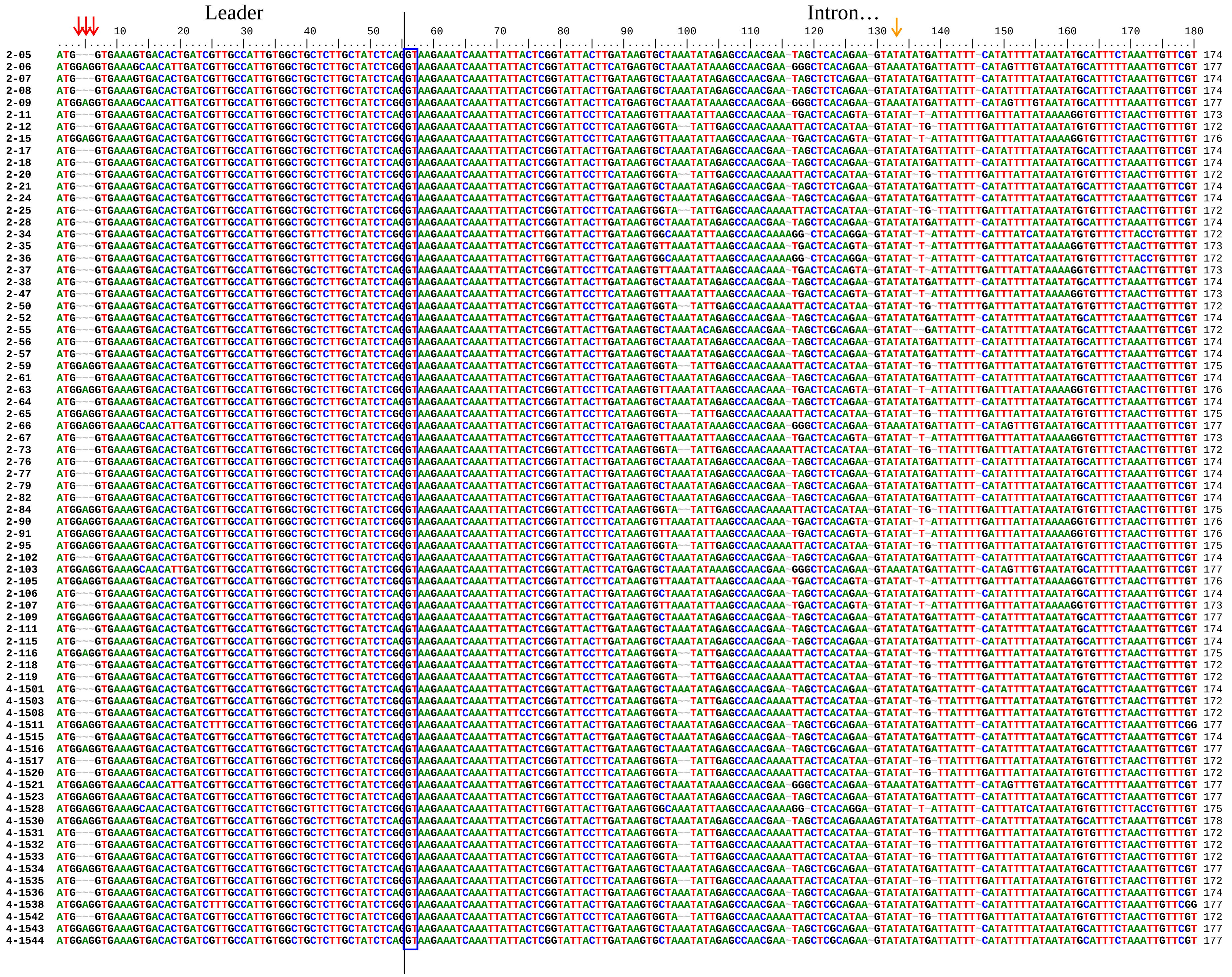

Leader
Intron…
10
20
30
40
50
60
70
80
90
100
110
120
130
140
150
160
170
180

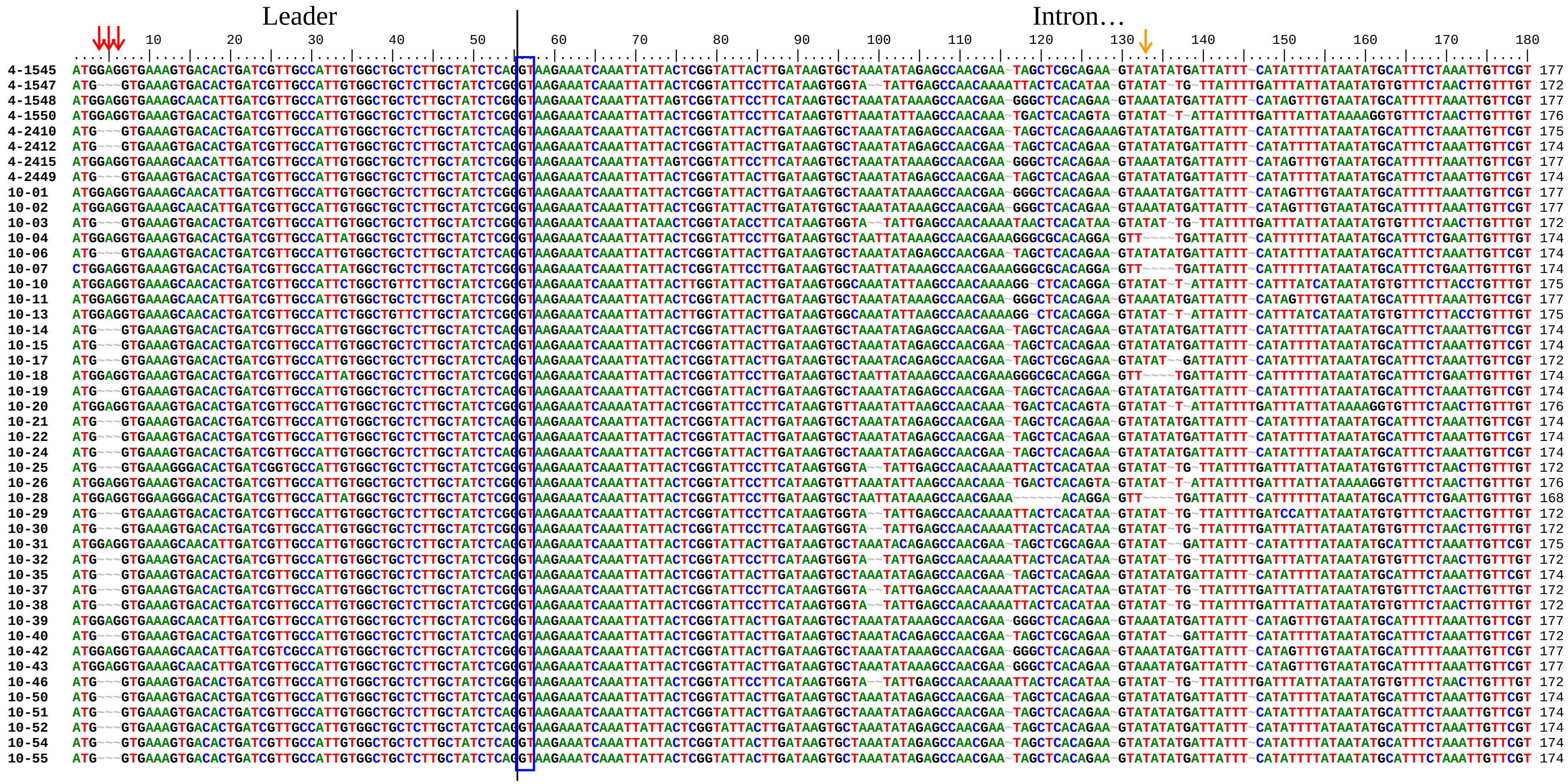
Leader
Intron…
10
20
30
40
50
60
70
80
90
100
110
120
130
140
150
160
170
180
4-1545 177
4-1547 172
4-1548 177
4-1550 176
4-2410 175
4-2412 174
4-2415 177
4-2449 174
10-01 177
10-02 177
10-03 172
10-04 174
10-06 174
10-07 174
10-10 175
10-11 177
10-13 175
10-14 174
10-15 174
10-17 172
10-18 174
10-19 174
10-20 176
10-21 174
10-22 174
10-24 174
10-25 172
10-26 176
10-28 168
10-29 172
10-30 172
10-31 175
10-32 172
10-35 174
10-37 172
10-38 172
10-39 177
10-40 172
10-42 177
10-43 177
10-46 172
10-50 174
10-51 174
10-52 174
10-54 174
10-55 174

…Intron…

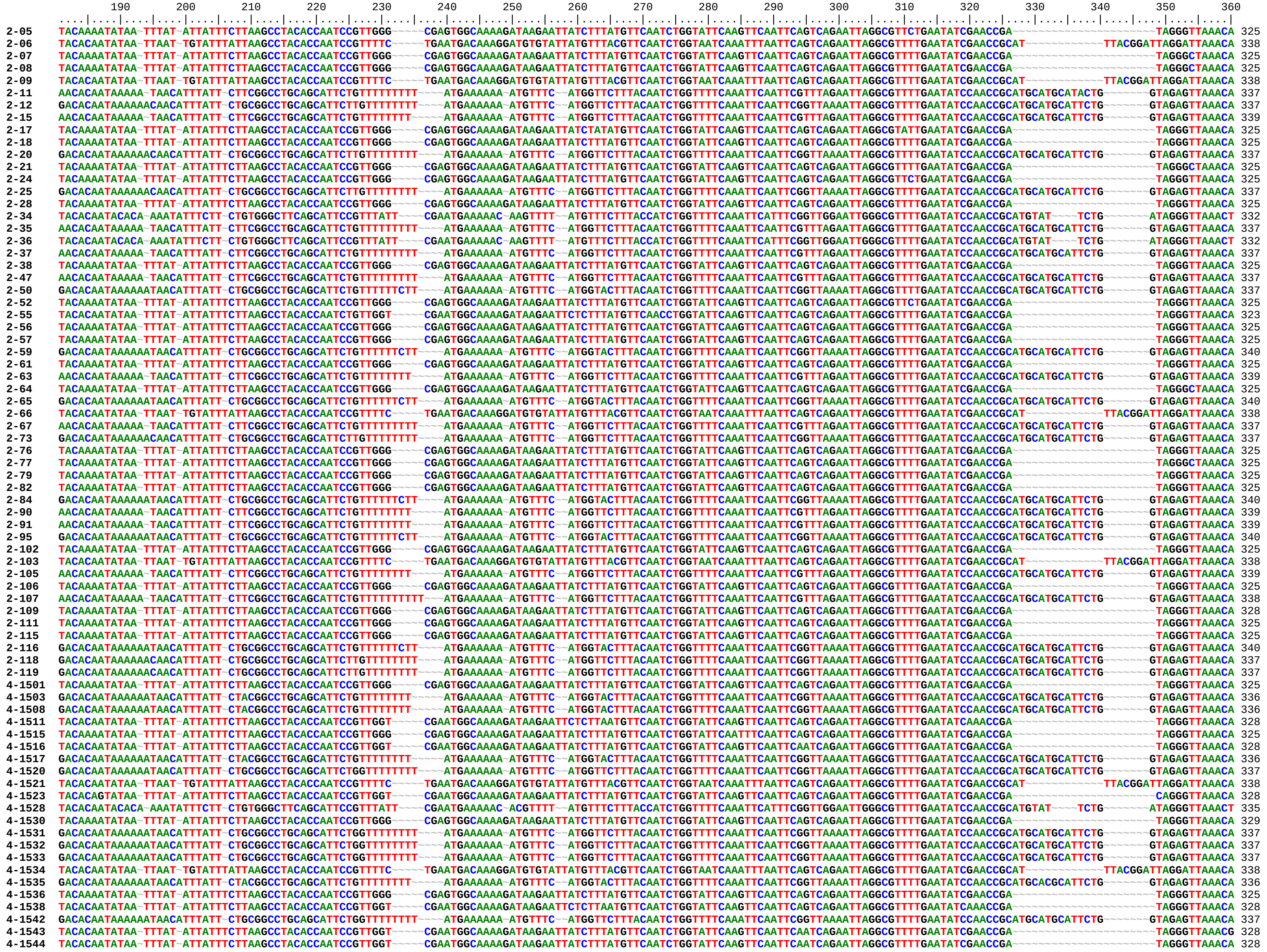

…Intron…

```
                 190       200       210       220       230       240       250       260       270       280       290       300       310       320       330       340       350       360
         ....|....|....|....|....|....|....|....|....|....|....|....|....|....|....|....|....|....|....|....|....|....|....|....|....|....|....|....|....|....|....|....|....|....|....|....|
4-1545   TACACAATATAA~TTTAT~ATTATTTCTTAAGCCTACACCAATCCGTTGGT~~~~~CGAATGGCAAAAGATAAGAATTATCTTTATGTTCAATCTGGTATTCAAGTTCAATTCAATCAGAATTAGGCGTTTTGAATATCGAACCGA~~~~~~~~~~~~~~~~~~~~~~TAGGGTTAAACA 328
4-1547   GACACAATAAAAAATAACATTTATT~CTACGGCCTGCAGCATTCTGTTTTTTTT~~~~~ATGAAAAAA~ATGTTTC~~ATGGTACTTTACAATCTGGTTTTCAAATTCAATTCGGTTAAAATTAGGCGTTTTGAATATCCAACCGCATGCATGCATTCTG~~~~~~~GTAGAGTTAAACA 336
4-1548   TACACAATATAA~TTAAT~TGTATTTATTAAGCCTACACCAATCCGTTTTC~~~~~TGAATGACAAAGGATGTGTATTATGTTTACGTTCAATCTGGTAATCAAATTTAATTCAGTCAGAATTAGGCGTTTTGAATATCGAACCGCAT~~~~~~~~~~~~TTACGGATTAGGATTAAACA 338
4-1550   AACACAATAAAAA~TAACATTTATT~CTTCGGCCTGCAGCATTCTGTTGTTTTTT~~~~ATGAAAAAA~ATGTTTC~~ATGGTTCTTTACAATCTGGTTTTCAAATTGAATTCGTTTAGAATTAGGCGTTTTGAATATCCAACCGCATGCATGCATTCTG~~~~~~~GTAGAGTTAAACA 340
4-2410   TACAAAATATAA~TTTAT~ATTATTTCTTAAGCCTACACCAATCCGTTGGG~~~~~CGAGTGGCAAAAGATAAGAATTATCTTTATGTTCAATCTGGTATTCAAGTTCAATTCAGTCAGAATTAGGCGTTTTGAATATCGAACCGA~~~~~~~~~~~~~~~~~~~~~~TAGGGTTAAACA 326
4-2412   TACAAAATATAA~TTTAT~ATTATTTCTTAAGCCTACACCAATCCGTTGGG~~~~~CGAGTGGCAAAAGATAAGAATTATCTTTATGTTCAATCTGGTATTCAATTTCAATTCAGTCAGAATTAGGCGTTTTGAATATCGAACCGA~~~~~~~~~~~~~~~~~~~~~~TAGGGTTAAACA 325
4-2415   TACACAATATAA~TTAAT~TGTATTTATTAAGCCTACACCAATCCGTTTTC~~~~~TGAATGACAAAGGATGTGTATTATGTTTACGTTCAATCTGGTAATCAAATTTAATTCAGTCAGAATTAGGCGTTTTGAATATCGAACCGCAT~~~~~~~~~~~~TTACGGATTAGGATTAAACA 338
4-2449   TACAAAATATAA~TTTAT~ATTATTTCTTAAGCCTACACCAATCCGTTGGG~~~~~CGAGTGGCAAAAGATAAGAATTATCTTTATGTTCAATCTGGTATTCAATTTCAATTCAGTCAGAATTAGGCGTTTTGAATATCGAACCGA~~~~~~~~~~~~~~~~~~~~~~TAGGGTTAAACA 325
10-01    TACACAATATAA~TTAAT~TGTATTTATTAAGCCTACACCAATCCGTTTTC~~~~~TGAATGACAAAGGATGTGTATTATGTTTACGTTCAATCTGGTAATCAAATTTAATTCAGTCAGAATTAGGCGTTTTGAATATCGAACCGCAT~~~~~~~~~~~~TTACGGATTAGGATTAAACA 338
10-02    TACACAATATAA~TTAAT~TGTATTTATTAAGCCTACACCAATCCGTTTTC~~~~~TGAATGACAAAGGATGTGTATTATGTTTACGTTCAATCTGGTAATCAAATTTAATTCAGTCAGAATTAGGCGTTTTGAATATCGAACCGCAT~~~~~~~~~~~~TTACGGATTAGGATTAAACA 338
10-03    GACACAATAAAAAATAACATTTATT~CTGCGGCCTGCAGCATTCTGGTTTTTTTT~~~~ATGAAAAAA~ATGTTTC~~ATGGTACTTTACAATCTGGTTTTCAAATTCAATTCGGTTAAAATTAGGCGTTTTGAATATCCAACCGCATGCATGCATTCTG~~~~~~~GTAGAGTTAAACA 337
10-04    TACACAATATAA~TTAAC~TTTATTTCTTAAACCTACACCAATCCGTTGGT~~~~~CGAATGACAAAAGATGTGTATTATCTTTATGTTCAATCTGGTATTCAAATTCAATTCAGTCAGAATTAGGCGTTTTGAATATCGAACAACAT~~~~~~~~~~~~TTGAGGAGTAAAGTTAAACA 335
10-06    TACAAAATATAA~TTTAT~ATTATTTCTTAAGCCTACACCAATCCGTTGGG~~~~~CGAGTGGCAAAAGATAAGAATTATCTTTATGTTCAATCTGGTATTCAAGTTCAATTCAGTCAGAATTAGGCGTTTTGAATATCGAACCGA~~~~~~~~~~~~~~~~~~~~~~TAGGGTTAAACA 325
10-07    TACACAATATAA~TTAAC~TTTATTTCTTAAACCTACACCAATCCGTTGGT~~~~~CGAATGACAAAAGATGTGTATTATCTTTATGTTCAATCTGGTATTCAAATTCAATTCAGTCAGAATTAGGCGTTTTGAATATCGAACAACAT~~~~~~~~~~~~TTGAGGAGTAAAGTTAAACA 335
10-10    TACACAATACACA~AAATATTTCTT~CTGTGGGCTTCAGCATTCCGTTTATT~~~~CGAATGAAAAAC~ACGTTTT~~ATGTTTCTTTACCATCTGGTTTTCAAATTCATTTCGGTTGGAATTGGGCGTTTTGAATATCCAACCGCATGTAT~~~~TCTG~~~~~~~ATAGGGTTAAACT 335
10-11    TACACAATATAA~TTAAT~TGTATTTATTAAGCCTACACCAATCCGTTTTC~~~~~TGAATGACAAAGGATGTGTATTATGTTTACGTTCAATCTGGTAATCAAATTTAATTCAGTCAGAATTAGGCGTTTTGAATATCGAACCGCAT~~~~~~~~~~~~TTACGGATTAGGATTAAACA 338
10-13    TACACAATACACA~AAATATTTCTT~CTGTGGGCTTCAGCATTCCGTTTATT~~~~CGAATGAAAAAC~ACGTTTT~~ATGTTTCTTTACCATCTGGTTTTCAAATTCATTTCGGTTGGAATTGGGCGTTTTGAATATCCAACCGCATGTAT~~~~TCTG~~~~~~~ATAGGGTTAAACT 335
10-14    TACAAAATATAA~TTTAT~ATTATTTCTTAAGCCTACACCAATCCGTTGGG~~~~~CGAGTGGCAAAAGATAAGAATTATCTTTATGTTCAATCTGGTATTCAAGTTCAATTCAGTCAGAATTAGGCGTTTTGAATATCGAACCGA~~~~~~~~~~~~~~~~~~~~~~TAGGGTTAAACA 325
10-15    TACAAAATATAA~TTTAT~ATTATTTCTTAAGCCTACACCAATCCGTTGGG~~~~~CGAGTGGCAAAAGATAAGAATTATCTTTATGTTCAATCTGGTATTCAAGTTCAATTCAGTCAGAATTAGGCGTTTTGAATATCGAACCGA~~~~~~~~~~~~~~~~~~~~~~TAGGGTTAA~CA 324
10-17    TACACAATATAA~TTTAT~ATTATTTCTTAAGCCTACACCAATCTGTTGGT~~~~~CGAATGGCAAAAGATAAGAATTCTCTTTATGTTCAACCTGGTATTCAAGTTCAATTCAGTCAGAATTAGGCGTTTTGAATATCGAACCGA~~~~~~~~~~~~~~~~~~~~~~TAGGGTTAAACA 323
10-18    TACACAATATAA~TTAAC~TTTATTTCTTAAACCTACACCAATCCGTTGGT~~~~~CGAATGACAAAAGATGTGTATTATCTTTATGTTCAATCTGGTATTCAAATTCAATTCAGTCAGAATTAGGCGTTTTGAATATCGAACAACAT~~~~~~~~~~~~TTGAGGAGTAAAGTTAAACA 335
10-19    TACAAAATATAA~TTTAT~ATTATTTCTTAAGCCTACACCAATCCGTTGGG~~~~~CGAGTGGCAAAAGATAAGAATTATCTTTATGTTCAATCTGGTATTCAAGTTCAATTCAGTCAGAATTAGGCGTTTTGAATATCGAACCGA~~~~~~~~~~~~~~~~~~~~~~TAGGGTTAA~CA 324
10-20    AACACAATAAAAA~TAACATTTATT~CTTCGGCCTGCAGCATTCTGTTTTTTTTTT~~~ATGAAAAAA~ATGTTTC~~ATGGTTCTTTACAATCTGGTTTTCAAATTCAATTCGTTTAGAATTAGGCGTTTTGAATATCCAACCGCATGCATGCATTCTG~~~~~~~GTAGAGTTAAACA 341
10-21    TACAAAATATAA~TTTAT~ATTATTTCTTAAGCCTACACCAATCCGTTGGG~~~~~CGAGTGGCAAAAGATAAGAATTATCTTTATGTTCAATCTGGTATTCAAGTTCAATTCAGTCAGAATTAGGCGTTTTGAATATCGAACCGA~~~~~~~~~~~~~~~~~~~~~~TAGGGTTAAACA 325
10-22    TACAAAATATAA~TTTAT~ATTATTTCTTAAGCCTACACCAATCCGTTGGG~~~~~CGAGTGGCAAAAGATAAGAATTATCTTTATGTTCAATCTGGTATTCAAGTTCAATTCAGTCAGAATTAGGCGTTTTGAATATCGAACCGA~~~~~~~~~~~~~~~~~~~~~~TAGGGTTAAACA 325
10-24    TACAAAATATAA~TTTAT~ATTATTTCTTAAGCCTACACCAATCCGTTGGG~~~~~CGAGTGGCAAAAGATAAGAATTATCTTTATGTTCAATCTGGTATTCAAGTTCAATTCAGTCAGAATTAGGCGTTTTGAATATCGAACCGA~~~~~~~~~~~~~~~~~~~~~~TAGGGTTAA~CA 324
10-25    GACACAATAAAAAATAACATTTATT~CTGCGGCCTGCAGCATTCTGGTTTTTTTT~~~~ATGAAAAAA~ATGTTTC~~ATGGTACTTTACAATCTGGTTTTCAAATTCAATTCGGTTAAAATTAGGCGTTTTGAATATCCAACCGCATGCATGCATTCTG~~~~~~~GTAGAGTTAAACA 337
10-26    AACACAATAAAAA~TAACATTTATT~CTTCGGCCTGCAGCATTCTGTTTTTTTTTT~~~ATGAAAAAA~ATGTTTC~~ATGGTTCTTTACAATCTGGTTTTCAAATTCAATTCGTTTAGAATTAGGCGTTTTGAATATCCAACCGCATGCATGCATTCTG~~~~~~~GTAGAGTTAAACA 341
10-28    TACACAATATAA~TTAAC~TTTATTTCTTAAACCTACACCAATCCGTTGGT~~~~~CGAATGACAAAAGATGTGTATTATCTTTATGTTCAATCTGGTATTCAAATTCAATTCAGTCAGAATTAGGCGTTTTGAATATCGAACAACAT~~~~~~~~~~~~TTGAGGAGTAAAGTTAAACA 329
10-29    GACACAATAAAAAATAACATTTATT~CTGCGGCCTGCAGCATTCTGGTTTTTTTT~~~~ATGAAAAAA~ATGTTTC~~ATGGTACTTTACAATCTGGTTTTCAAATTCAATTCGGTTAAAATTAGGCGTTTTGAATATCCAACCGCATGCATGCATTCTG~~~~~~~GTAGAGTTAAACA 337
10-30    GACACAATAAAAAATAACATTTATT~CTGCGGCCTGCAGCATTCTGGTTTTTTTT~~~~ATGAAAAAA~ATGTTTC~~ATGGTACTTTACAATCTGGTTTTCAAATTCAATTCGGTTAAAATTAGGCGTTTTGAATATCCAACCGCATGCATGCATTCTG~~~~~~~GTAGAGTTAAACA 337
10-31    TACACAATATAA~TTTAT~ATTATTTCTTAAGCCTACACCAATCTGTTGGT~~~~~CGAATGGCAAAAGATAAGAATTCTCTTTATGTTCAACCTGGTATTCAAGTTCAATTCAGTCAGAATTAGGCGTTTTGAATATCGAACCGA~~~~~~~~~~~~~~~~~~~~~~TAGGGTTAAACA 326
10-32    GACACAATAAAAAATAACATTTATT~CTGCGGCCTGCAGCATTCTGGTTTTTTTT~~~~ATGAAAAAA~ATGTTTC~~ATGGTACTTTACAATCTGGTTTTCAAATTCAATTCGGTTAAAATTAGGCGTTTTGAATATCCAACCGCATGCATGCATTCTG~~~~~~~GTAGAGTTAAACA 337
10-35    TACAAAATATAA~TTTAT~ATTATTTCTTAAGCCTACACCAATCCGTTGGG~~~~~CGAGTGGCAAAAGATAAGAATTATCTTTATGTTCAATCTGGTATTCAAGTTCAATTCAGTCAGAATTAGGCGTTTTGAATATCGAACCGA~~~~~~~~~~~~~~~~~~~~~~TAGGGTTAAACA 325
10-37    GACACAATAAAAAATAACATTTATT~CTGCGGCCTGCAGCATTCTGGTTTTTTTT~~~~ATGAAAAAA~ATGTTTC~~ATGGTACTTTACAATCTGGTTTTCAAATTCAATTCGGTTAAAATTAGGCGTTTTGAATATCCAACCGCATGCATGCATTCTG~~~~~~~GTAGAGTTAAACA 337
10-38    GACACAATAAAAAATAACATTTATT~CTGCGGCCTGCAGCATTCTGGTTTTTTTT~~~~ATGAAAAAA~ATGTTTC~~ATGGTACTTTACAATCTGGTTTTCAAATTCAATTCGGTTAAAATTAGGCGTTTTGAATATCCAACCGCATGCATGCATTCTG~~~~~~~GTAGAGTTAAACA 337
10-39    TACACAATATAA~TTAAT~TGTATTTATTAAGCCTACACCAATCCGTTTTC~~~~~TGAATGACAAAGGATGTGTATTATGTTTACGTTCAATCTGGTAATCAAATTTAATTCAGTCAGAATTAGGCGTTTTGAATATCGAACCGCAT~~~~~~~~~~~~TTACGGATTAGGATTAAACA 338
10-40    TACACAATATAA~TTTAT~ATTATTTCTTAAGCCTACACCAATCTGTTGGT~~~~~CGAATGGCAAAAGATAAGAATTCTCTTTATGTTCAACCTGGTATTCAAGTTCAATTCAGTCAGAATTAGGCGTTTTGAATATCGAACCGA~~~~~~~~~~~~~~~~~~~~~~TAGGGTTAAACA 323
10-42    TACACAATATAA~TTAAT~TGTATTTATTAAGCCTACACCAATCCGTTTTC~~~~~TGAATGACAAAGGATGTGTATTATGTTTACGTTCAATCTGGTAATCAAATTTAATTCAGTCAGAATTAGGCGTTTTGAATATCGAACCGCAT~~~~~~~~~~~~TTACGGATTAGGATTAAACA 338
10-43    TACACAATATAA~TTAAT~TGTATTTATTAAGCCTACACCAATCCGTTTTC~~~~~TGAATGACAAAGGATGTGTATTATGTTTACGTTCAATCTGGTAATCAAATTTAATTCAGTCAGAATTAGGCGTTTTGAATATCGAACCGCAT~~~~~~~~~~~~TTACGGATTAGGATTAAACA 338
10-46    GACACAATAAAAAATAACATTTATT~CTGCGGCCTGCAGCATTCTGGTTTTTTTT~~~~ATGAAAAAA~ATGTTTC~~ATGGTACTTTACAATCTGGTTTTCAAATTCAATTCGGTTAAAATTAGGCGTTTTGAATATCCAACCGCATGCATGCATTCTG~~~~~~~GTAGAGTTAAACA 337
10-50    TACAAAATATAA~TTTAT~ATTATTTCTTAAGCCTACACCAATCCGTTGGG~~~~~CGAGTGGCAAAAGATAAGAATTATCTTTATGTTCAATCTGGTATTCAAGTTCAATTCAGTCAGAATTAGGCGTTTTGAATATCGAACCGA~~~~~~~~~~~~~~~~~~~~~~TAGGGTTAAACA 325
10-51    TACAAAATATAA~TTTAT~ATTATTTCTTAAGCCTACACCAATCCGTTGGG~~~~~CGAGTGGCAAAAGATAAGAATTATCTTTATGTTCAATCTGGTATTCAAGTTCAATTCAGTCAGAATTAGGCGTTTTGAATATCGAACCGA~~~~~~~~~~~~~~~~~~~~~~TAGGGTTAAACA 325
10-52    TACAAAATATAA~TTTAT~ATTATTTCTTAAGCCTACACCAATCCGTTGGG~~~~~CGAGTGGCAAAAGATAAGAATTATCTTTATGTTCAATCTGGTATTCAAGTTCAATTCAGTCAGAATTAGGCGTTTTGAATATCGAACCGA~~~~~~~~~~~~~~~~~~~~~~TAGGGTTAAACA 325
10-54    TACAAAATATAA~TTTAT~ATTATTTCTTAAGCCTACACCAATCCGTTGGG~~~~~CGAGTGGCAAAAGATAAGAATTATCTTTATGTTCAATCTGGTATTCAAGTTCAATTCAGTCAGAATTAGGCGTTTTGAATATCGAACCGA~~~~~~~~~~~~~~~~~~~~~~TAGGGTTAAACA 325
10-55    TACAAAATATAA~TTTAT~ATTATTTCTTAAGCCTACACCAATCCGTTGGG~~~~~CGAGTGGCAAAAGATAAGAATTATCTTTATGTTCAATCTGGTATTCAAGTTCAATTCAGTCAGAATTAGGCGTTTTGAATATCGAACCGA~~~~~~~~~~~~~~~~~~~~~~TAGGGTTAAACA
```

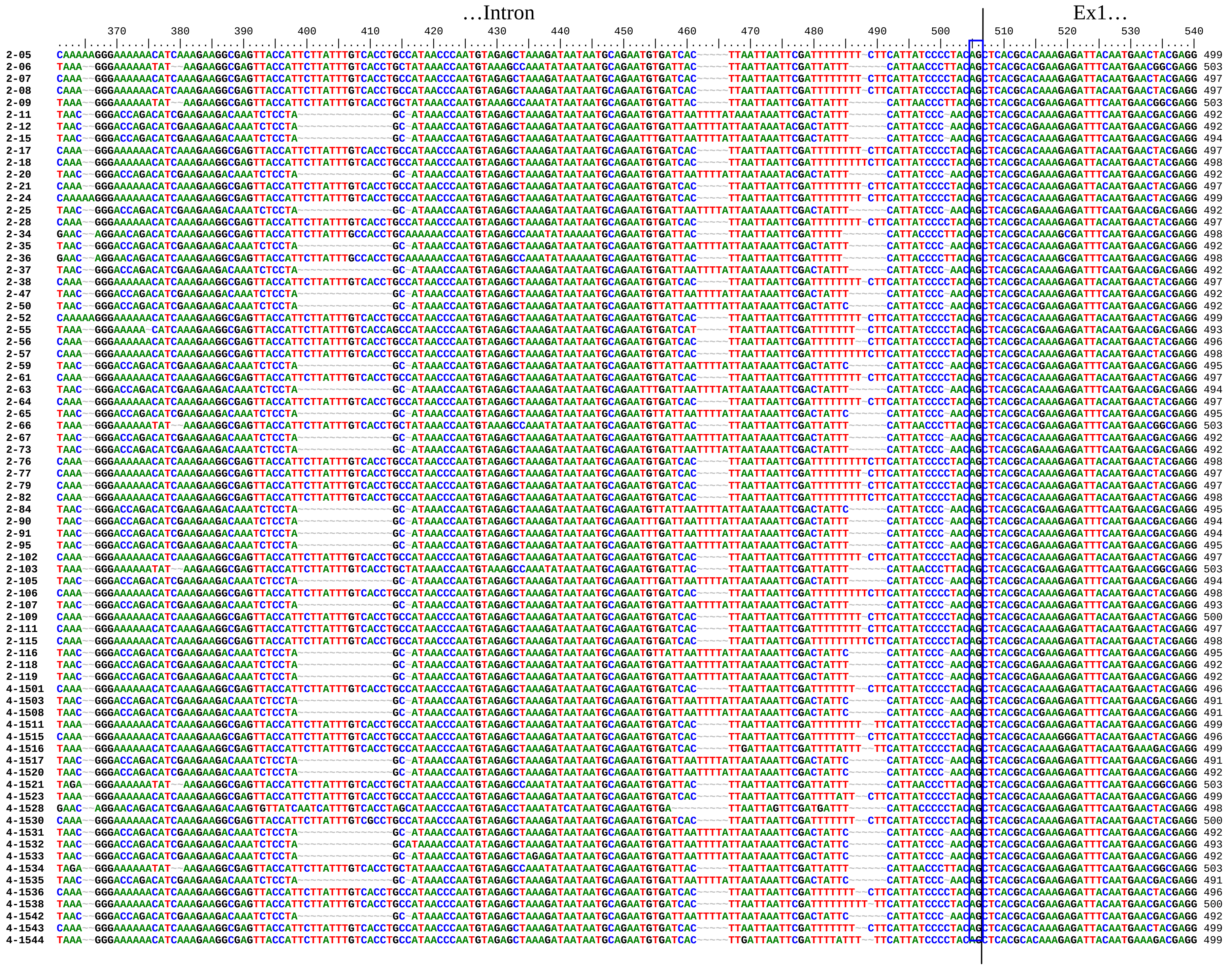
…Intron
Ex1…
370 380 390 400 410 420 430 440 450 460 470 480 490 500 510 520 530 540

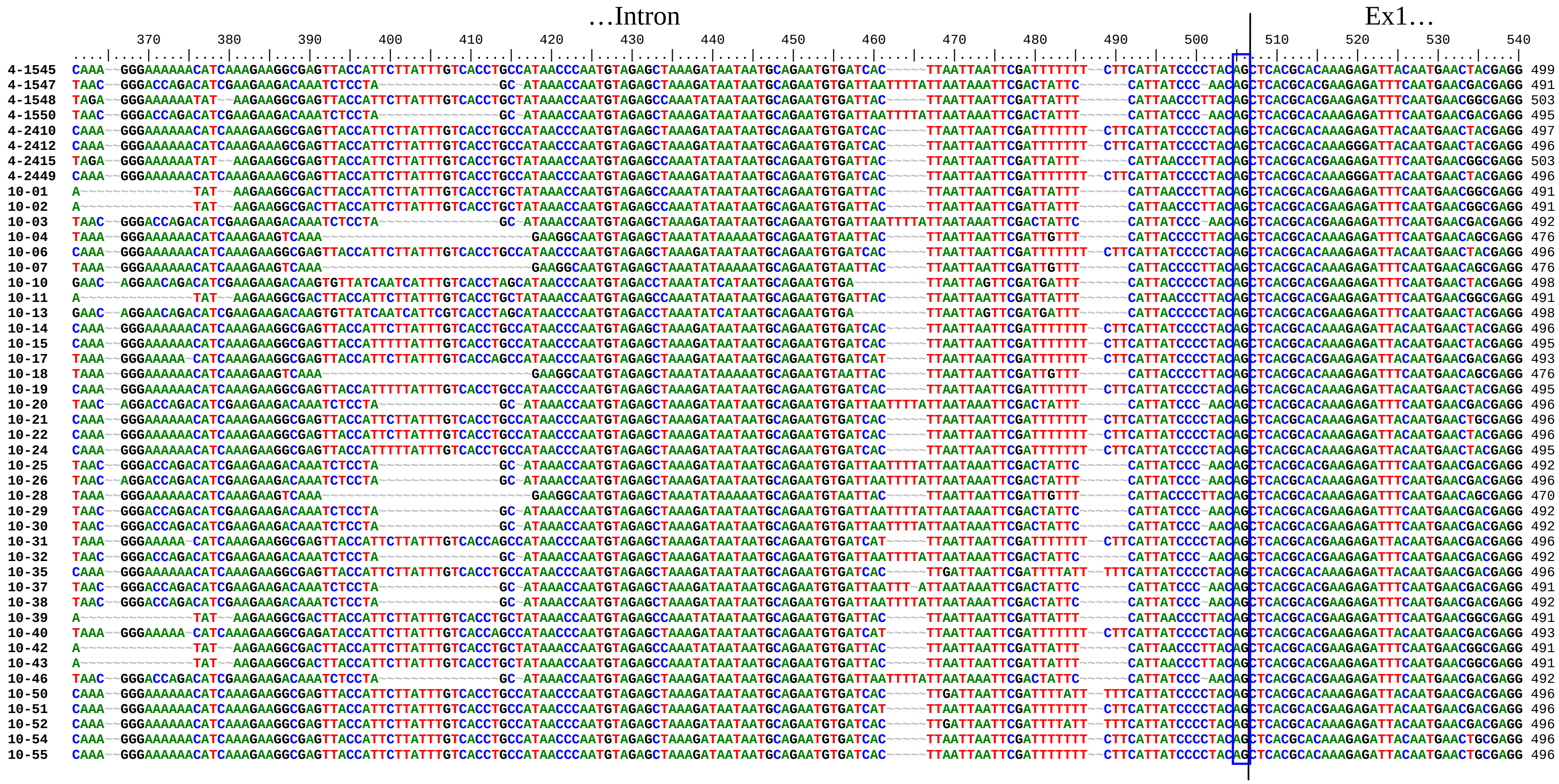
…Intron
Ex1…

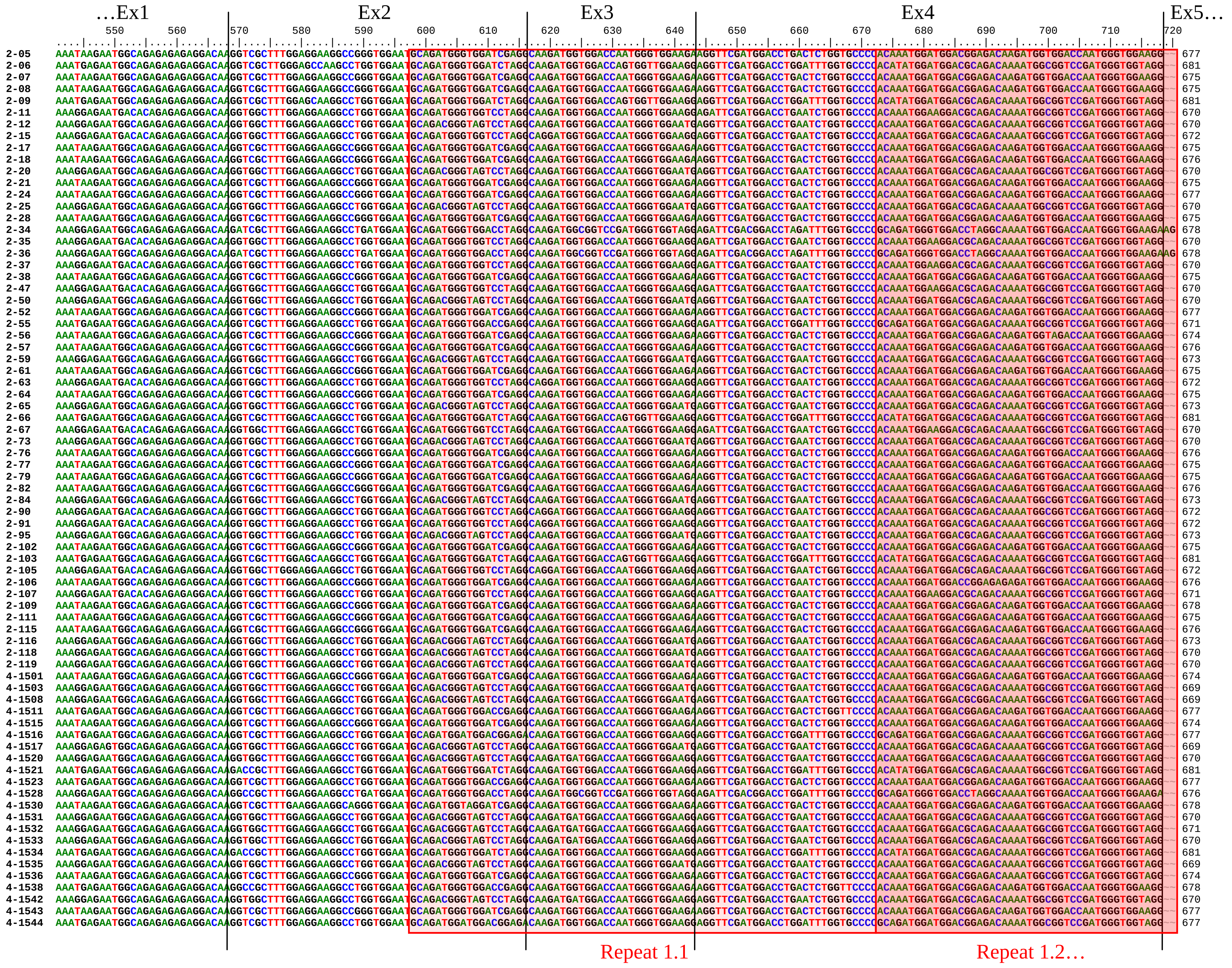
…Ex1
Ex2
Ex3
Ex4
Ex5…
Repeat 1.1
Repeat 1.2…

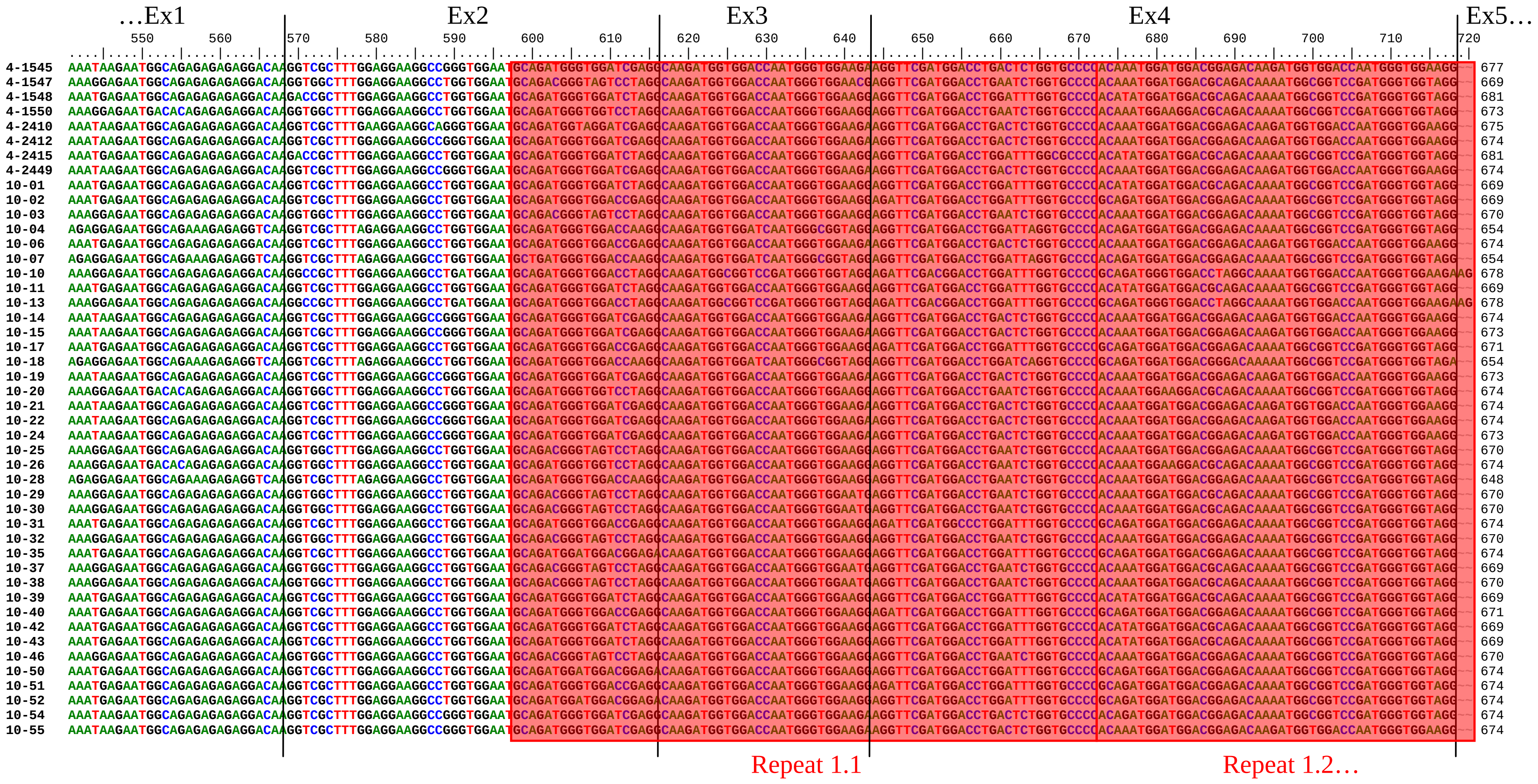

…Ex1
Ex2
Ex3
Ex4
Ex5…
Repeat 1.1
Repeat 1.2…

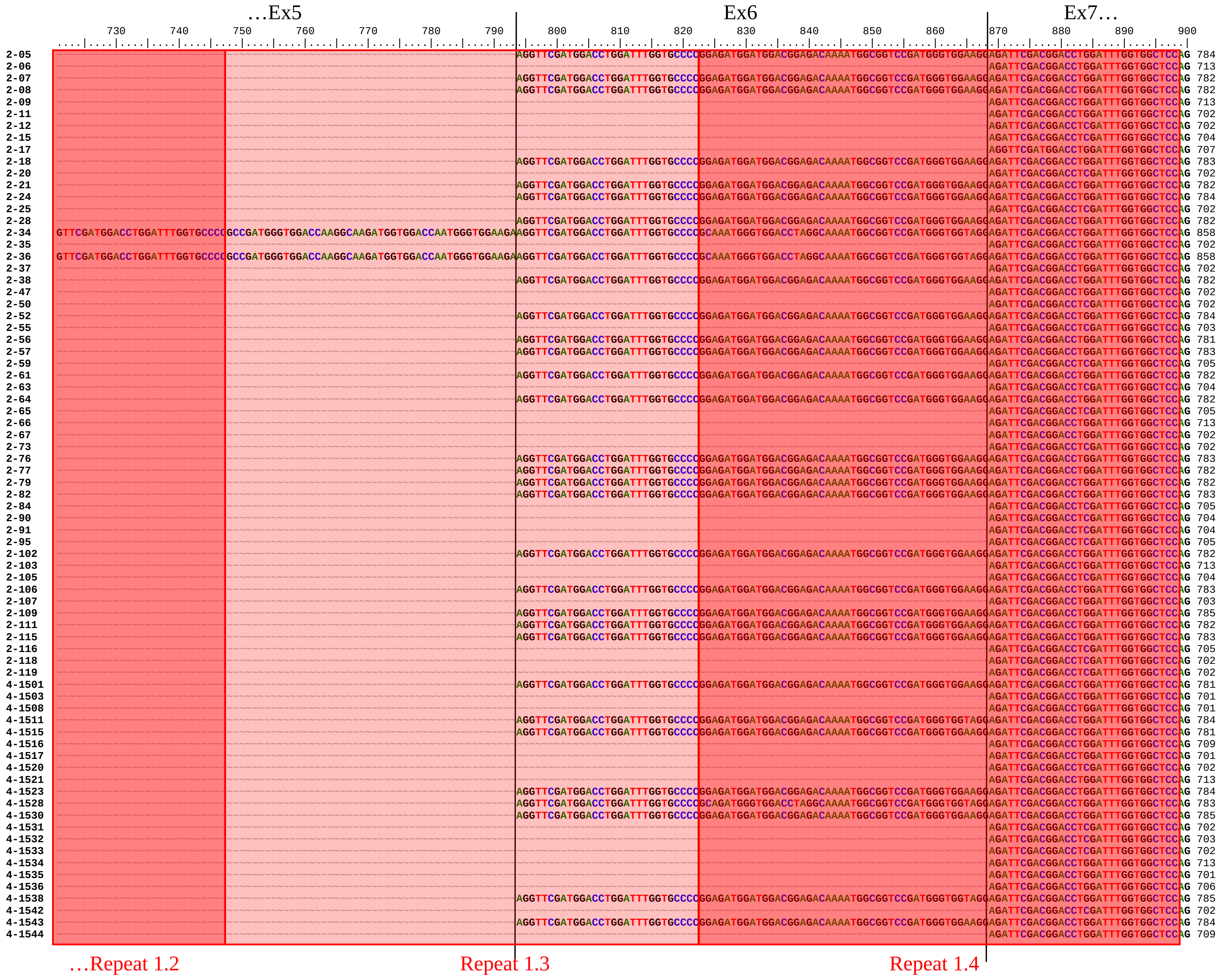
…Ex5
Ex6
Ex7…
…Repeat 1.2
Repeat 1.3
Repeat 1.4

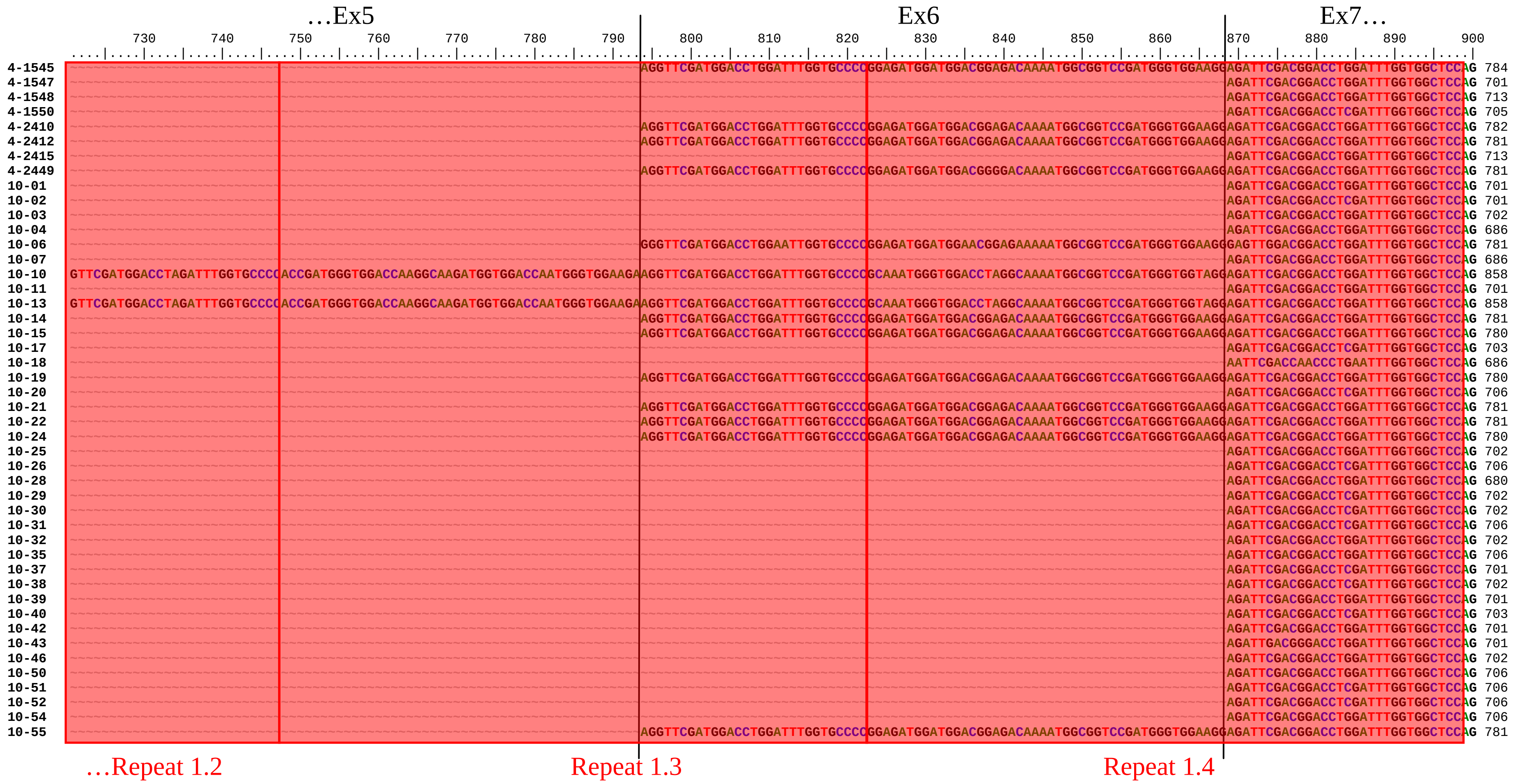

…Ex5
Ex6
Ex7…
…Repeat 1.2
Repeat 1.3
Repeat 1.4

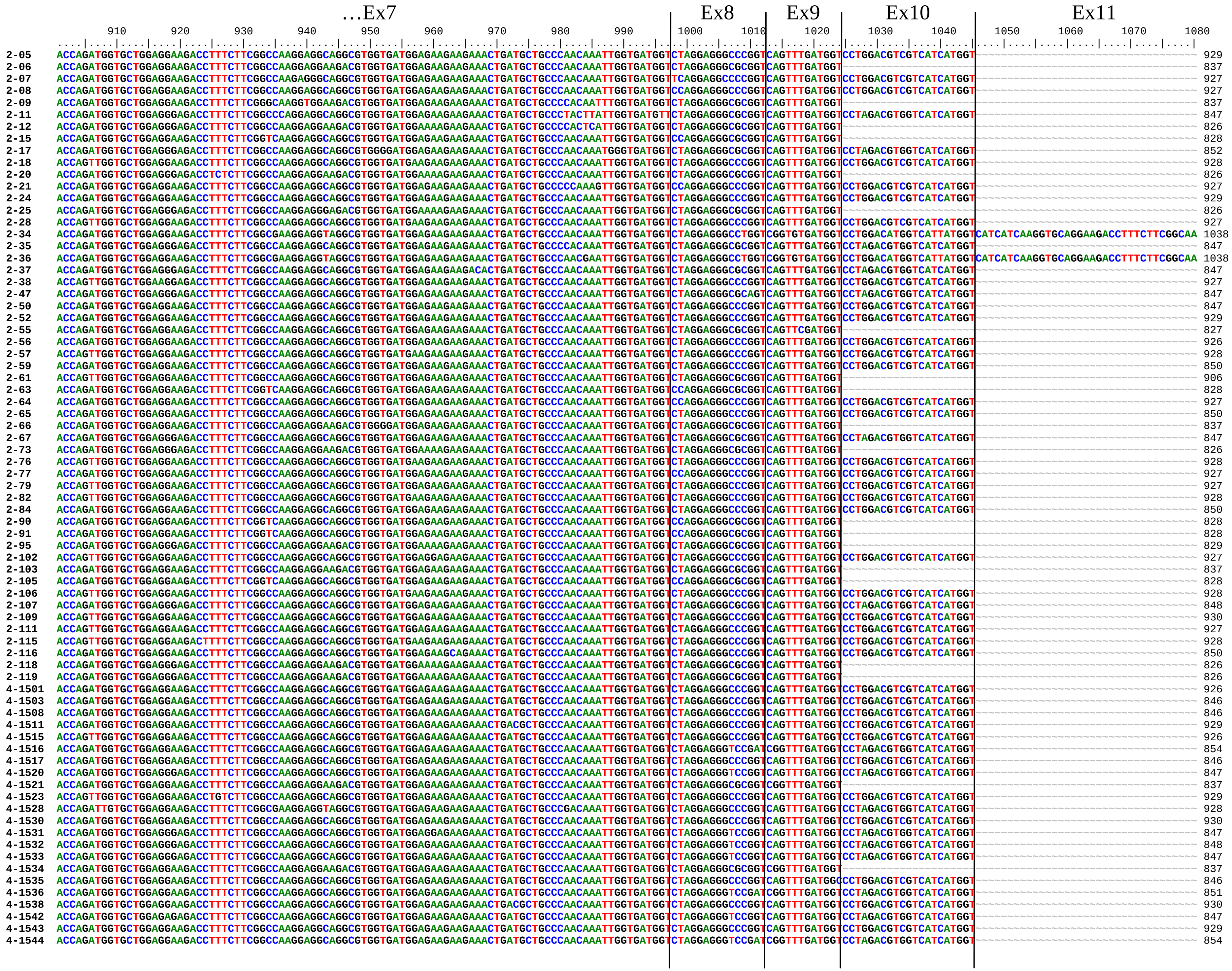
…Ex7
Ex8
Ex9
Ex10
Ex11
910 920 930 940 950 960 970 980 990 1000 1010 1020 1030 1040 1050 1060 1070 1080
2-05 929
2-06 837
2-07 927
2-08 927
2-09 837
2-11 847
2-12 826
2-15 828
2-17 852
2-18 928
2-20 826
2-21 927
2-24 929
2-25 826
2-28 927
2-34 1038
2-35 847
2-36 1038
2-37 847
2-38 927
2-47 847
2-50 847
2-52 929
2-55 827
2-56 926
2-57 928
2-59 850
2-61 906
2-63 828
2-64 927
2-65 850
2-66 837
2-67 847
2-73 826
2-76 928
2-77 927
2-79 927
2-82 928
2-84 850
2-90 828
2-91 828
2-95 829
2-102 927
2-103 837
2-105 828
2-106 928
2-107 848
2-109 930
2-111 927
2-115 928
2-116 850
2-118 826
2-119 826
4-1501 926
4-1503 846
4-1508 846
4-1511 929
4-1515 926
4-1516 854
4-1517 846
4-1520 847
4-1521 837
4-1523 929
4-1528 928
4-1530 930
4-1531 847
4-1532 848
4-1533 847
4-1534 837
4-1535 846
4-1536 851
4-1538 930
4-1542 847
4-1543 929
4-1544 854

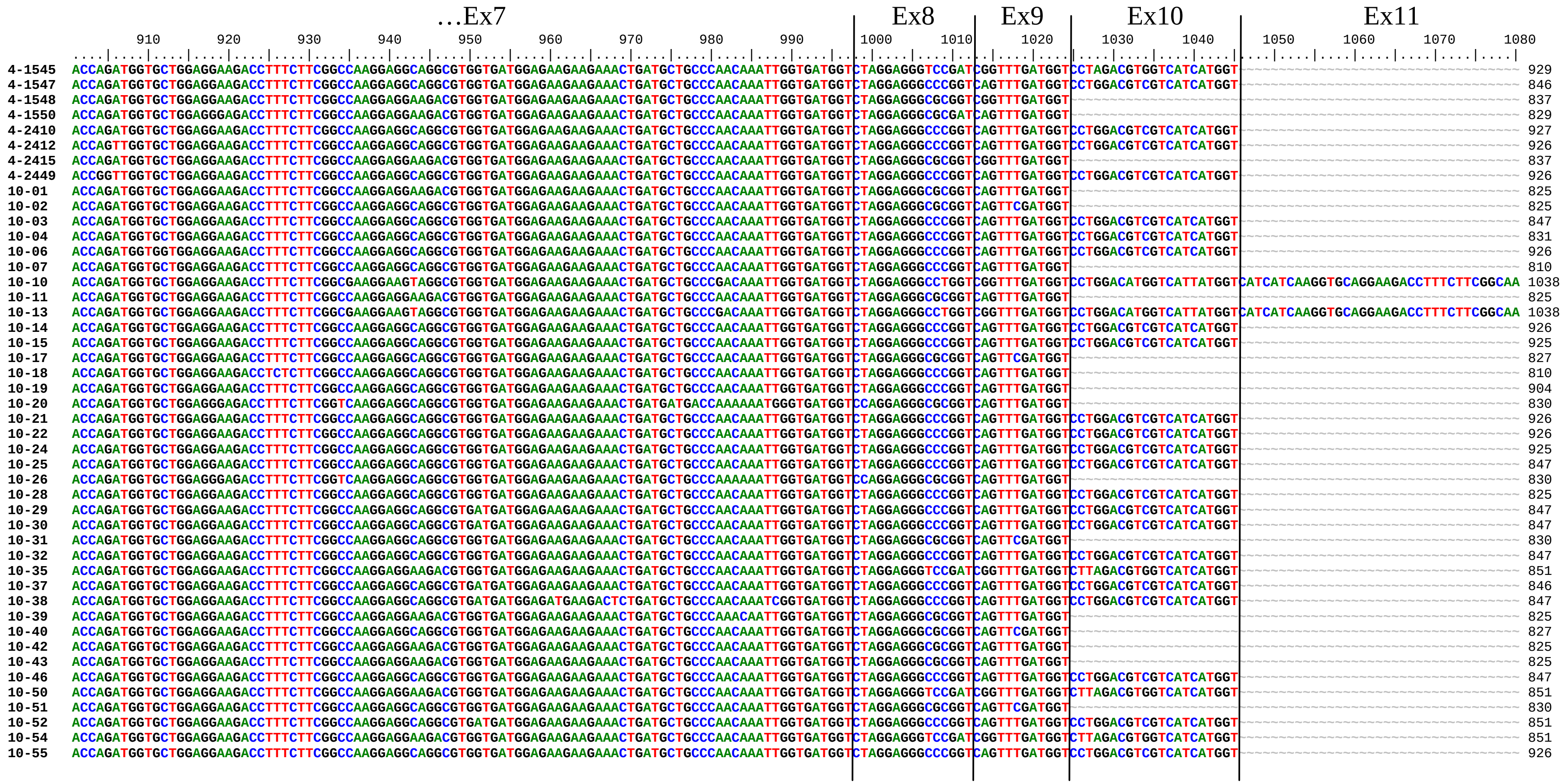

…Ex7
Ex8
Ex9
Ex10
Ex11
910 920 930 940 950 960 970 980 990 1000 1010 1020 1030 1040 1050 1060 1070 1080
4-1545 929
4-1547 846
4-1548 837
4-1550 829
4-2410 927
4-2412 926
4-2415 837
4-2449 926
10-01 825
10-02 825
10-03 847
10-04 831
10-06 926
10-07 810
10-10 1038
10-11 825
10-13 1038
10-14 926
10-15 925
10-17 827
10-18 810
10-19 904
10-20 830
10-21 926
10-22 926
10-24 925
10-25 847
10-26 830
10-28 825
10-29 847
10-30 847
10-31 830
10-32 847
10-35 851
10-37 846
10-38 847
10-39 825
10-40 827
10-42 825
10-43 825
10-46 847
10-50 851
10-51 830
10-52 851
10-54 851
10-55 926

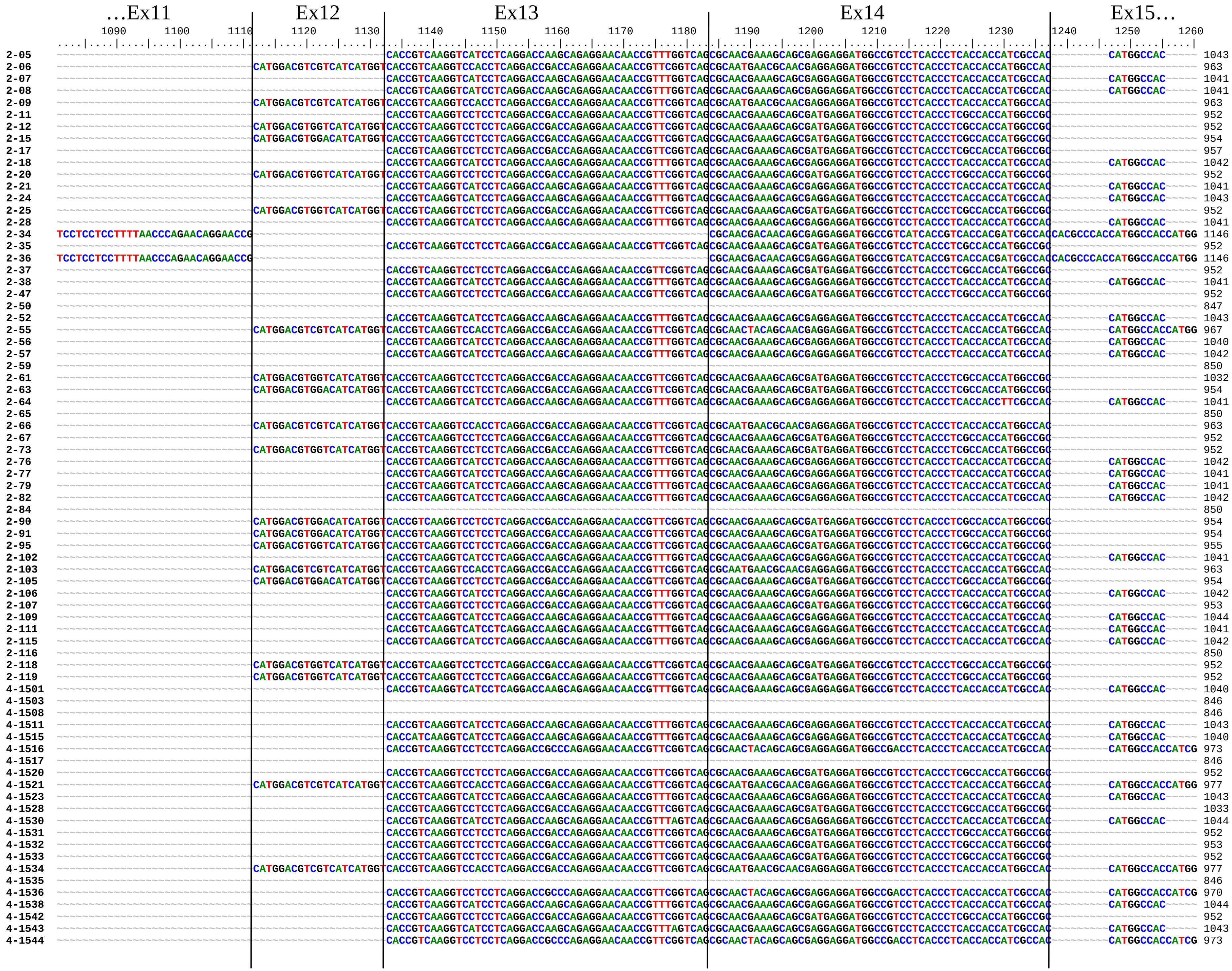
…Ex11
Ex12
Ex13
Ex14
Ex15…
1090
1100
1110
1120
1130
1140
1150
1160
1170
1180
1190
1200
1210
1220
1230
1240
1250
1260

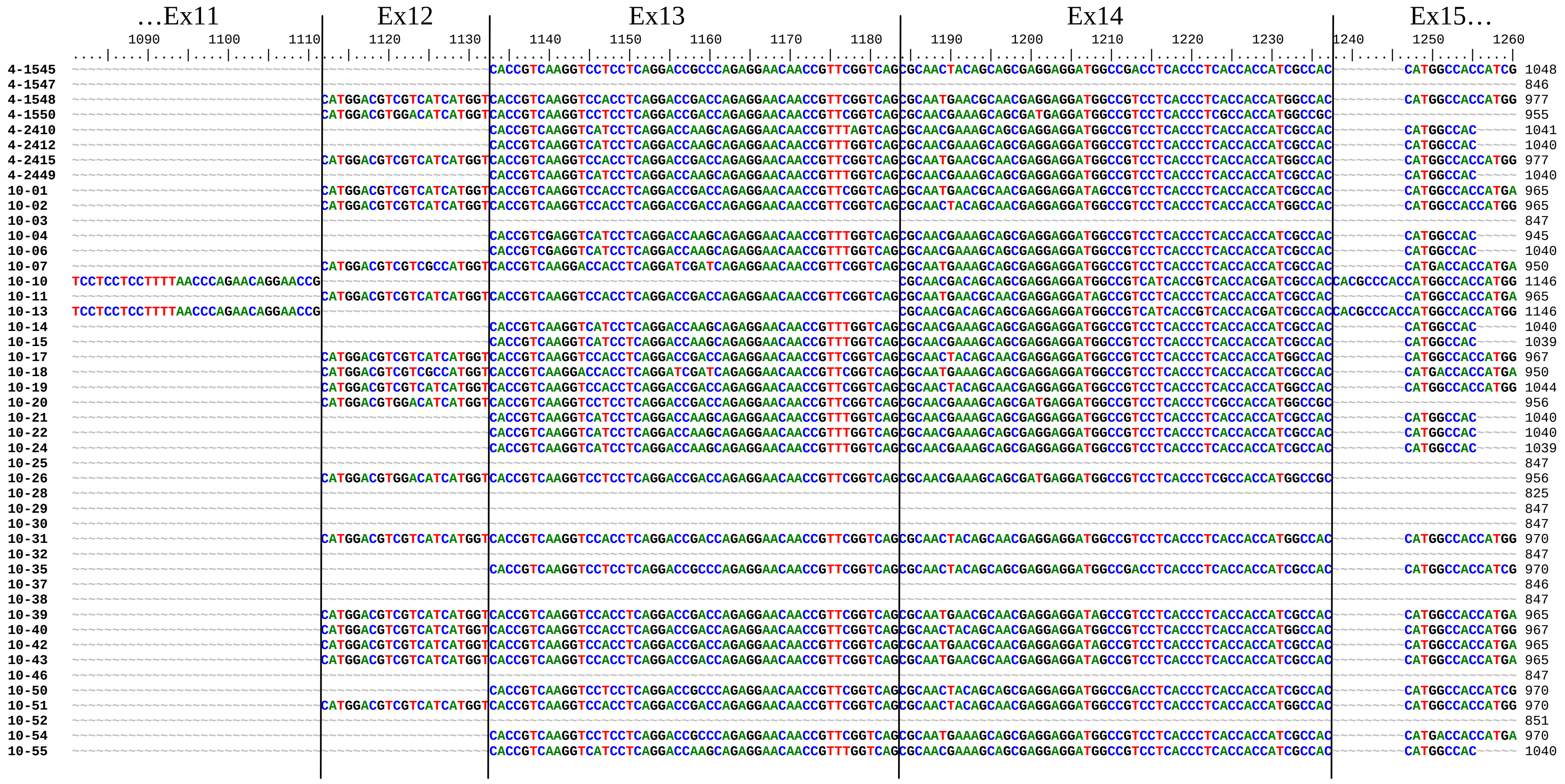
…Ex11
Ex12
Ex13
Ex14
Ex15…

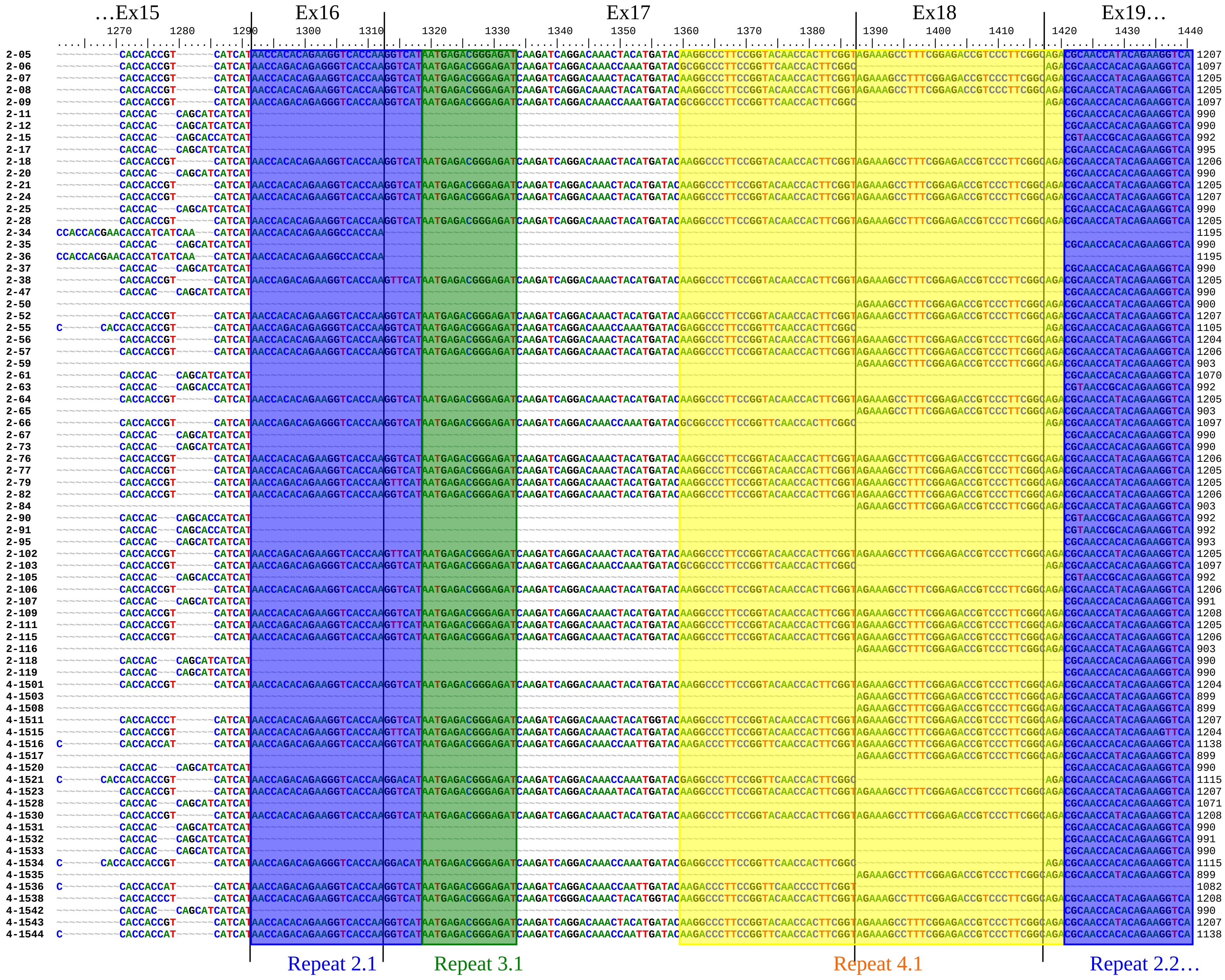

…Ex15
Ex16
Ex17
Ex18
Ex19…
Repeat 2.1
Repeat 3.1
Repeat 4.1
Repeat 2.2…

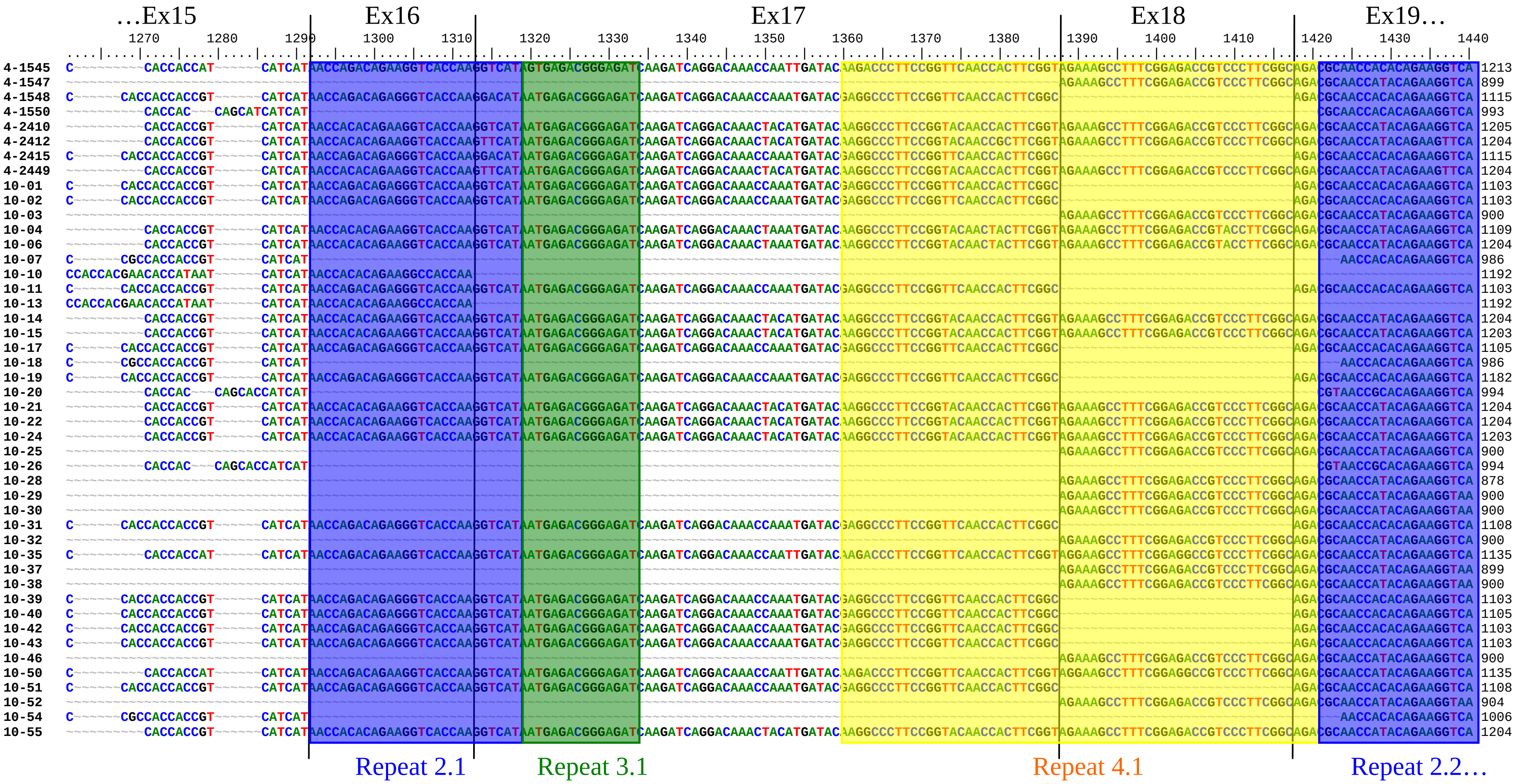

…Ex15
Ex16
Ex17
Ex18
Ex19…
Repeat 2.1
Repeat 3.1
Repeat 4.1
Repeat 2.2…

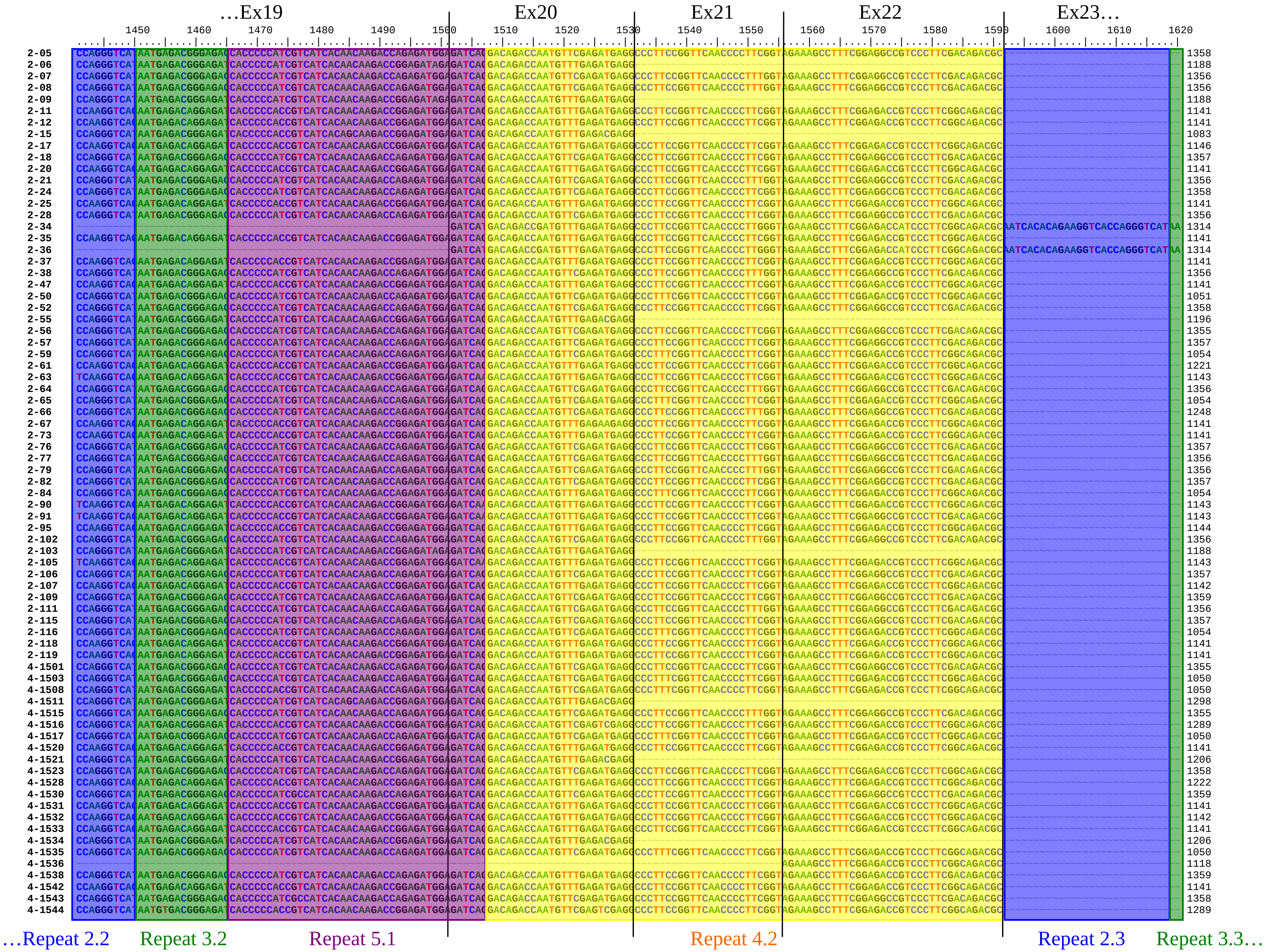

…Ex19
Ex20
Ex21
Ex22
Ex23…
…Repeat 2.2
Repeat 3.2
Repeat 5.1
Repeat 4.2
Repeat 2.3
Repeat 3.3…

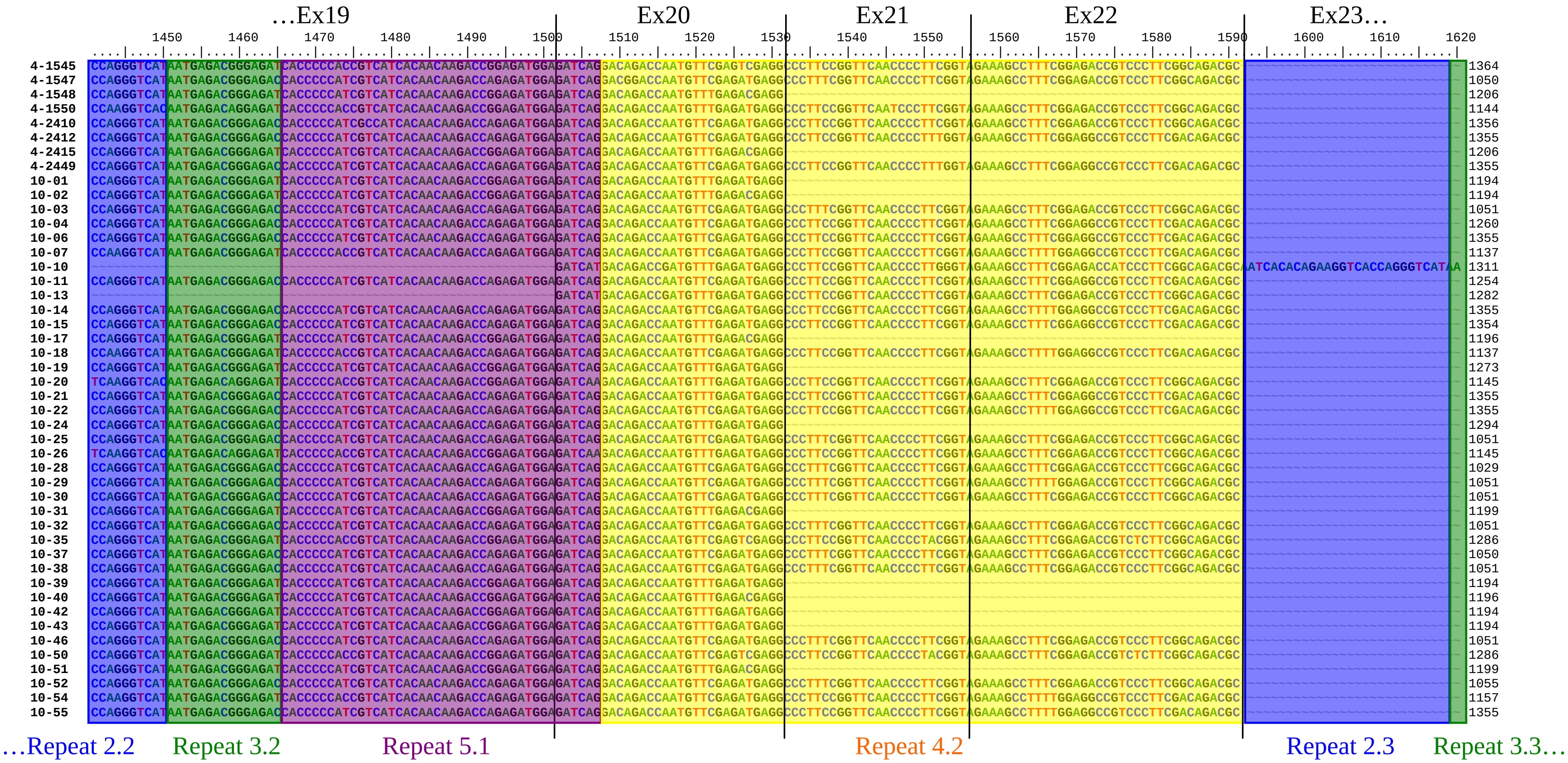
…Ex19
Ex20
Ex21
Ex22
Ex23…
…Repeat 2.2
Repeat 3.2
Repeat 5.1
Repeat 4.2
Repeat 2.3
Repeat 3.3…

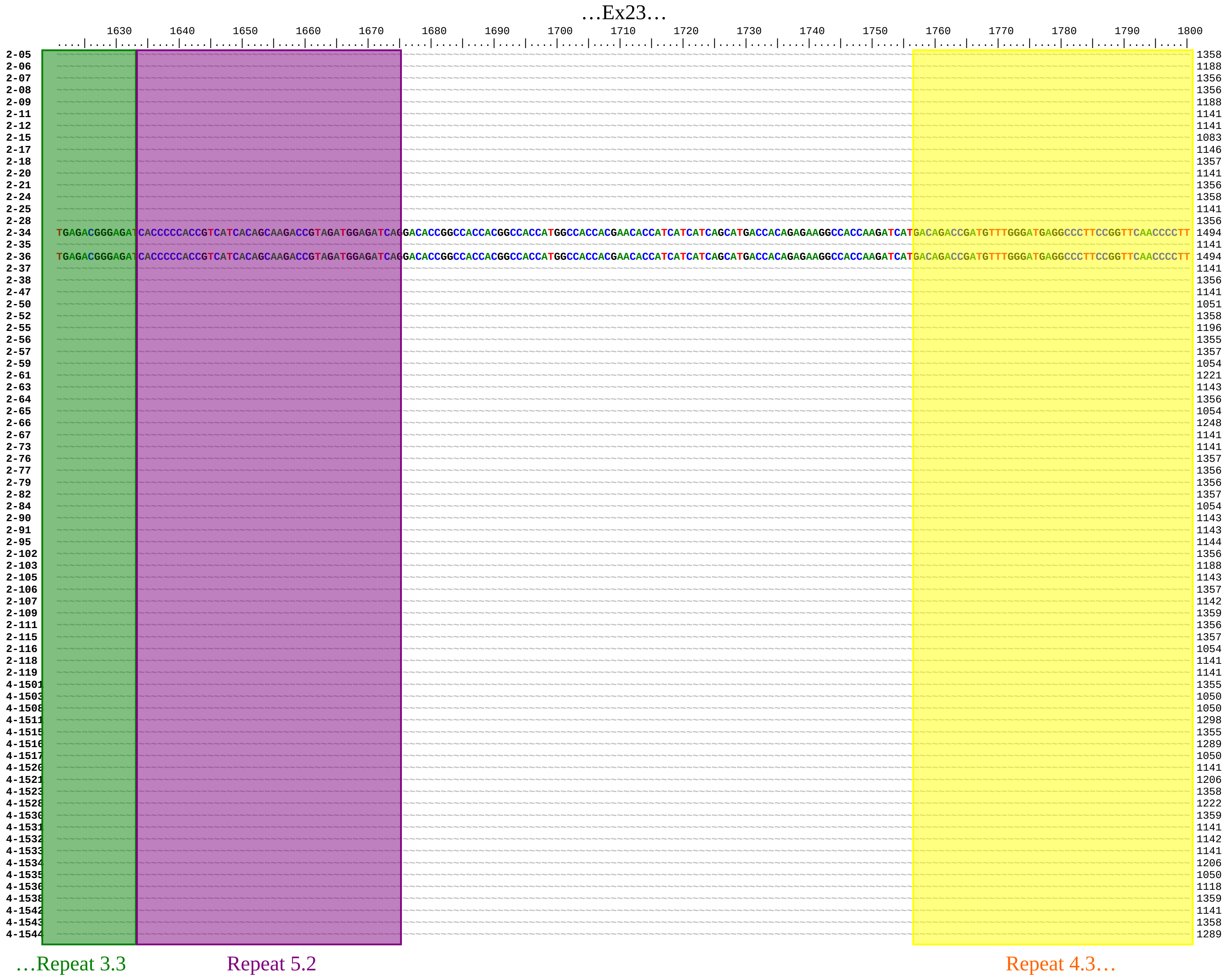
…Ex23…
…Repeat 3.3
Repeat 5.2
Repeat 4.3…

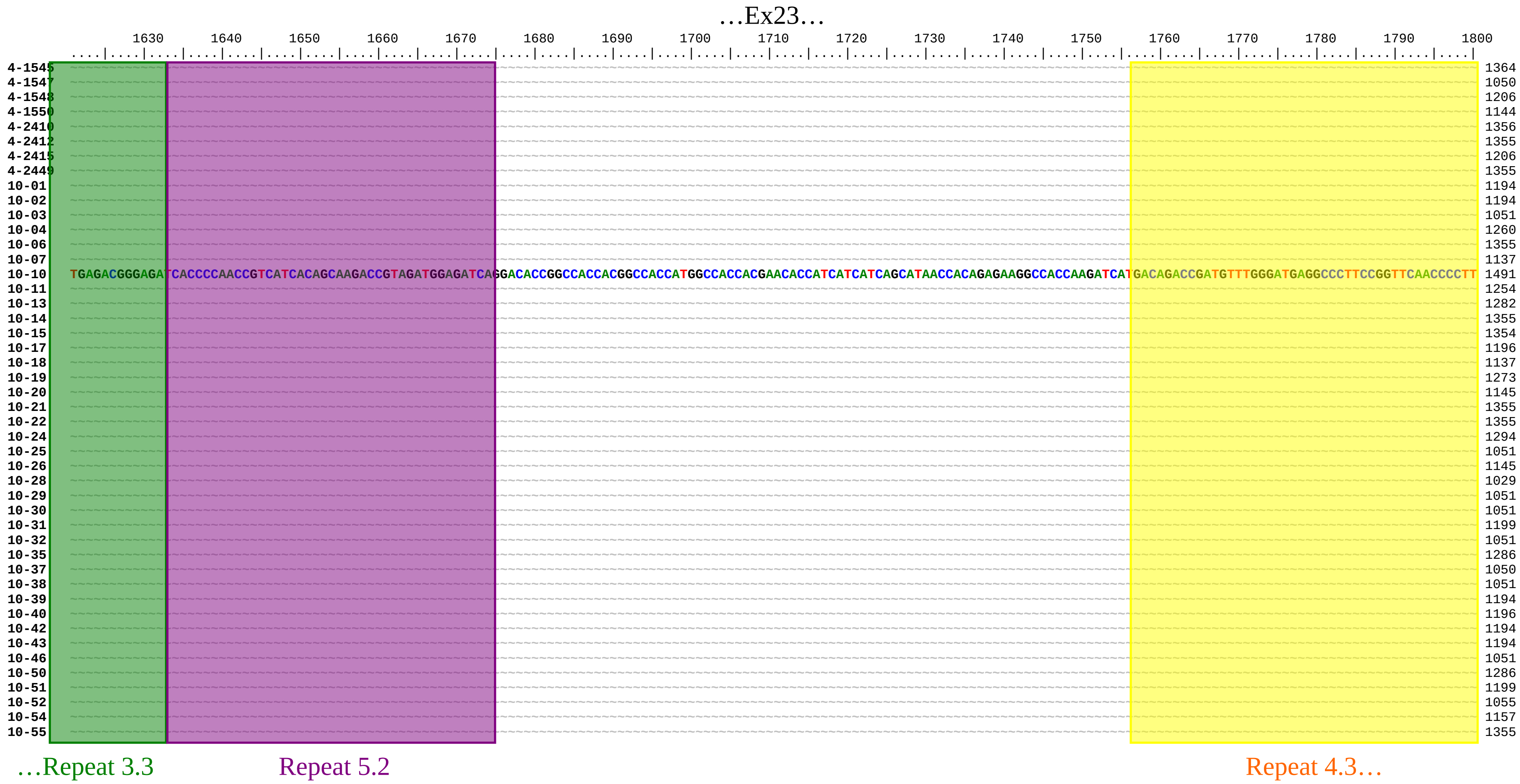

…Ex23…
1630 1640 1650 1660 1670 1680 1690 1700 1710 1720 1730 1740 1750 1760 1770 1780 1790 1800
4-1545 1364
4-1547 1050
4-1548 1206
4-1550 1144
4-2410 1356
4-2412 1355
4-2415 1206
4-2449 1355
10-01 1194
10-02 1194
10-03 1051
10-04 1260
10-06 1355
10-07 1137
10-10 TGAGACGGGAGATCACCCCAACCGTCATCACAGCAAGACCGTAGATGGAGATCAGGACACCGGCCACCACGGCCACCATGGCCACCACGAACACCATCATCATCAGCATAACCACAGAGAAGGCCACCAAGATCATGACAGACCGATGTTTGGGATGAGGCCCTTCCGGTTCAACCCCTT 1491
10-11 1254
10-13 1282
10-14 1355
10-15 1354
10-17 1196
10-18 1137
10-19 1273
10-20 1145
10-21 1355
10-22 1355
10-24 1294
10-25 1051
10-26 1145
10-28 1029
10-29 1051
10-30 1051
10-31 1199
10-32 1051
10-35 1286
10-37 1050
10-38 1051
10-39 1194
10-40 1196
10-42 1194
10-43 1194
10-46 1051
10-50 1286
10-51 1199
10-52 1055
10-54 1157
10-55 1355
…Repeat 3.3
Repeat 5.2
Repeat 4.3…

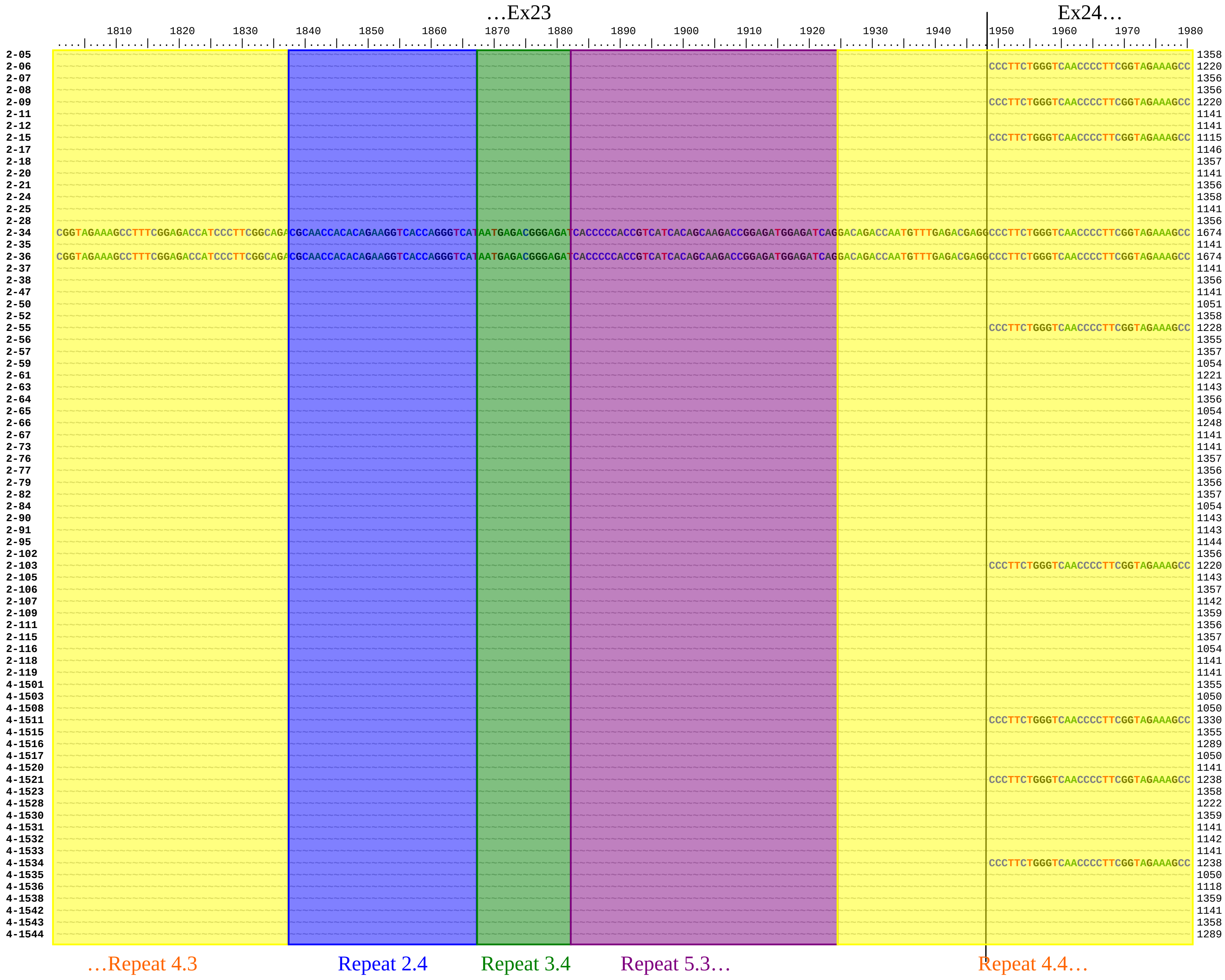

…Ex23
Ex24…
1810 1820 1830 1840 1850 1860 1870 1880 1890 1900 1910 1920 1930 1940 1950 1960 1970 1980
2-05 1358
2-06 CCCTTCTGGGTCAACCCCTTCGGTAGAAAGCC 1220
2-07 1356
2-08 1356
2-09 CCCTTCTGGGTCAACCCCTTCGGTAGAAAGCC 1220
2-11 1141
2-12 1141
2-15 CCCTTCTGGGTCAACCCCTTCGGTAGAAAGCC 1115
2-17 1146
2-18 1357
2-20 1141
2-21 1356
2-24 1358
2-25 1141
2-28 1356
2-34 CGGTAGAAAGCCTTTCGGAGACCATCCCTTCGGCAGACGCAACCACACAGAAGGTCACCAGGGTCATAATGAGACGGGAGATCACCCCCACCGTCATCACAGCAAGACCGGAGATGGAGATCAGGACAGACCAATGTTTGAGACGAGGCCCTTCTGGGTCAACCCCTTCGGTAGAAAGCC 1674
2-35 1141
2-36 CGGTAGAAAGCCTTTCGGAGACCATCCCTTCGGCAGACGCAACCACACAGAAGGTCACCAGGGTCATAATGAGACGGGAGATCACCCCCACCGTCATCACAGCAAGACCGGAGATGGAGATCAGGACAGACCAATGTTTGAGACGAGGCCCTTCTGGGTCAACCCCTTCGGTAGAAAGCC 1674
2-37 1141
2-38 1356
2-47 1141
2-50 1051
2-52 1358
2-55 CCCTTCTGGGTCAACCCCTTCGGTAGAAAGCC 1228
2-56 1355
2-57 1357
2-59 1054
2-61 1221
2-63 1143
2-64 1356
2-65 1054
2-66 1248
2-67 1141
2-73 1141
2-76 1357
2-77 1356
2-79 1356
2-82 1357
2-84 1054
2-90 1143
2-91 1143
2-95 1144
2-102 1356
2-103 CCCTTCTGGGTCAACCCCTTCGGTAGAAAGCC 1220
2-105 1143
2-106 1357
2-107 1142
2-109 1359
2-111 1356
2-115 1357
2-116 1054
2-118 1141
2-119 1141
4-1501 1355
4-1503 1050
4-1508 1050
4-1511 CCCTTCTGGGTCAACCCCTTCGGTAGAAAGCC 1330
4-1515 1355
4-1516 1289
4-1517 1050
4-1520 1141
4-1521 CCCTTCTGGGTCAACCCCTTCGGTAGAAAGCC 1238
4-1523 1358
4-1528 1222
4-1530 1359
4-1531 1141
4-1532 1142
4-1533 1141
4-1534 CCCTTCTGGGTCAACCCCTTCGGTAGAAAGCC 1238
4-1535 1050
4-1536 1118
4-1538 1359
4-1542 1141
4-1543 1358
4-1544 1289
…Repeat 4.3
Repeat 2.4
Repeat 3.4
Repeat 5.3…
Repeat 4.4…

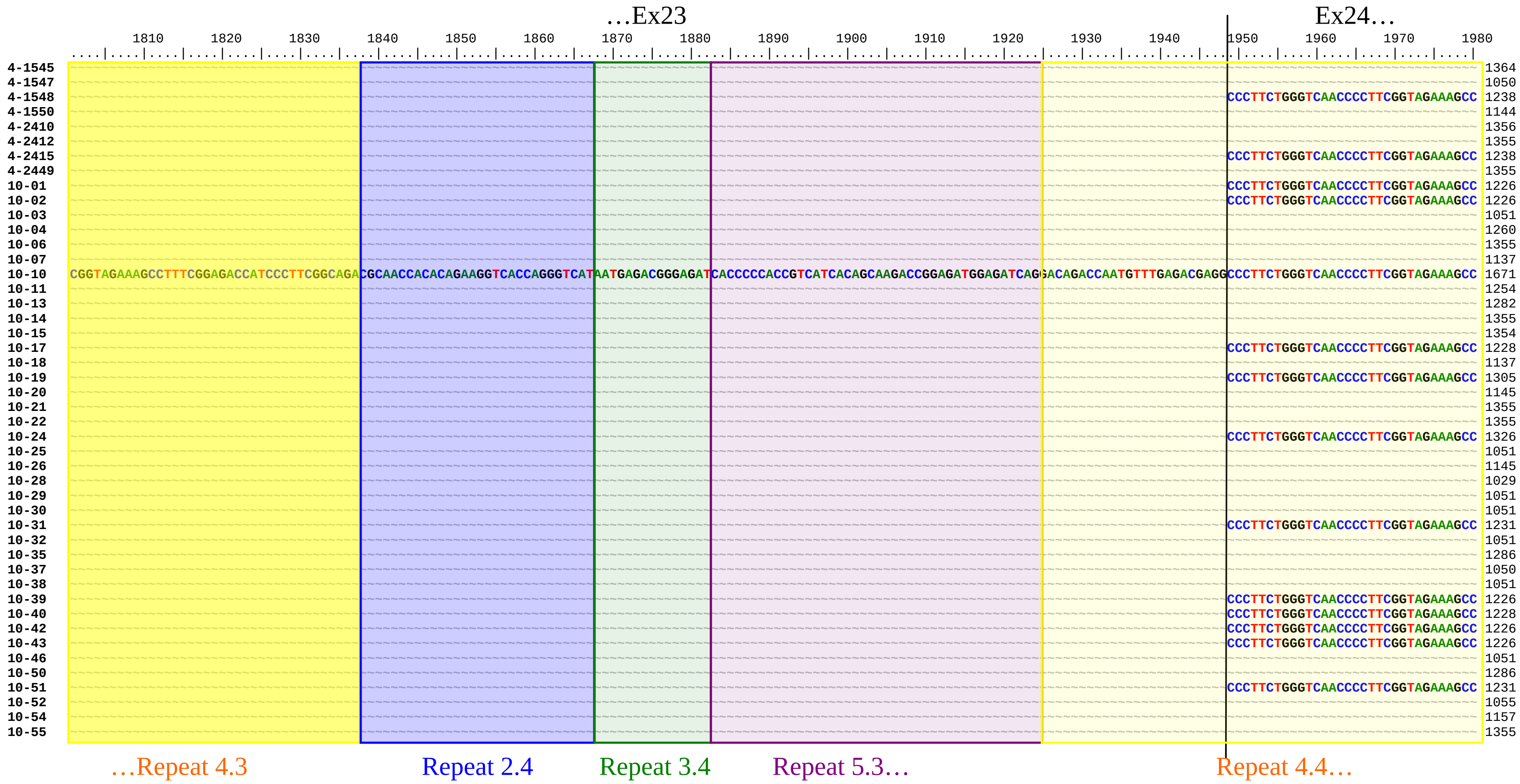

…Ex23
Ex24…
1810 1820 1830 1840 1850 1860 1870 1880 1890 1900 1910 1920 1930 1940 1950 1960 1970 1980
4-1545 1364
4-1547 1050
4-1548 CCCTTCTGGGTCAACCCCTTCGGTAGAAAGCC 1238
4-1550 1144
4-2410 1356
4-2412 1355
4-2415 CCCTTCTGGGTCAACCCCTTCGGTAGAAAGCC 1238
4-2449 1355
10-01 CCCTTCTGGGTCAACCCCTTCGGTAGAAAGCC 1226
10-02 CCCTTCTGGGTCAACCCCTTCGGTAGAAAGCC 1226
10-03 1051
10-04 1260
10-06 1355
10-07 1137
10-10 CGGTAGAAAGCCTTTCGGAGACCATCCCTTCGGCAGACGCAACCACACAGAAGGTCACCAGGGTCATAATGAGACGGGAGATCACCCCCACCGTCATCACAGCAAGACCGGAGATGGAGATCAGGACAGACCAATGTTTGAGACGAGGCCCTTCTGGGTCAACCCCTTCGGTAGAAAGCC 1671
10-11 1254
10-13 1282
10-14 1355
10-15 1354
10-17 CCCTTCTGGGTCAACCCCTTCGGTAGAAAGCC 1228
10-18 1137
10-19 CCCTTCTGGGTCAACCCCTTCGGTAGAAAGCC 1305
10-20 1145
10-21 1355
10-22 1355
10-24 CCCTTCTGGGTCAACCCCTTCGGTAGAAAGCC 1326
10-25 1051
10-26 1145
10-28 1029
10-29 1051
10-30 1051
10-31 CCCTTCTGGGTCAACCCCTTCGGTAGAAAGCC 1231
10-32 1051
10-35 1286
10-37 1050
10-38 1051
10-39 CCCTTCTGGGTCAACCCCTTCGGTAGAAAGCC 1226
10-40 CCCTTCTGGGTCAACCCCTTCGGTAGAAAGCC 1228
10-42 CCCTTCTGGGTCAACCCCTTCGGTAGAAAGCC 1226
10-43 CCCTTCTGGGTCAACCCCTTCGGTAGAAAGCC 1226
10-46 1051
10-50 1286
10-51 CCCTTCTGGGTCAACCCCTTCGGTAGAAAGCC 1231
10-52 1055
10-54 1157
10-55 1355
…Repeat 4.3
Repeat 2.4
Repeat 3.4
Repeat 5.3…
Repeat 4.4…

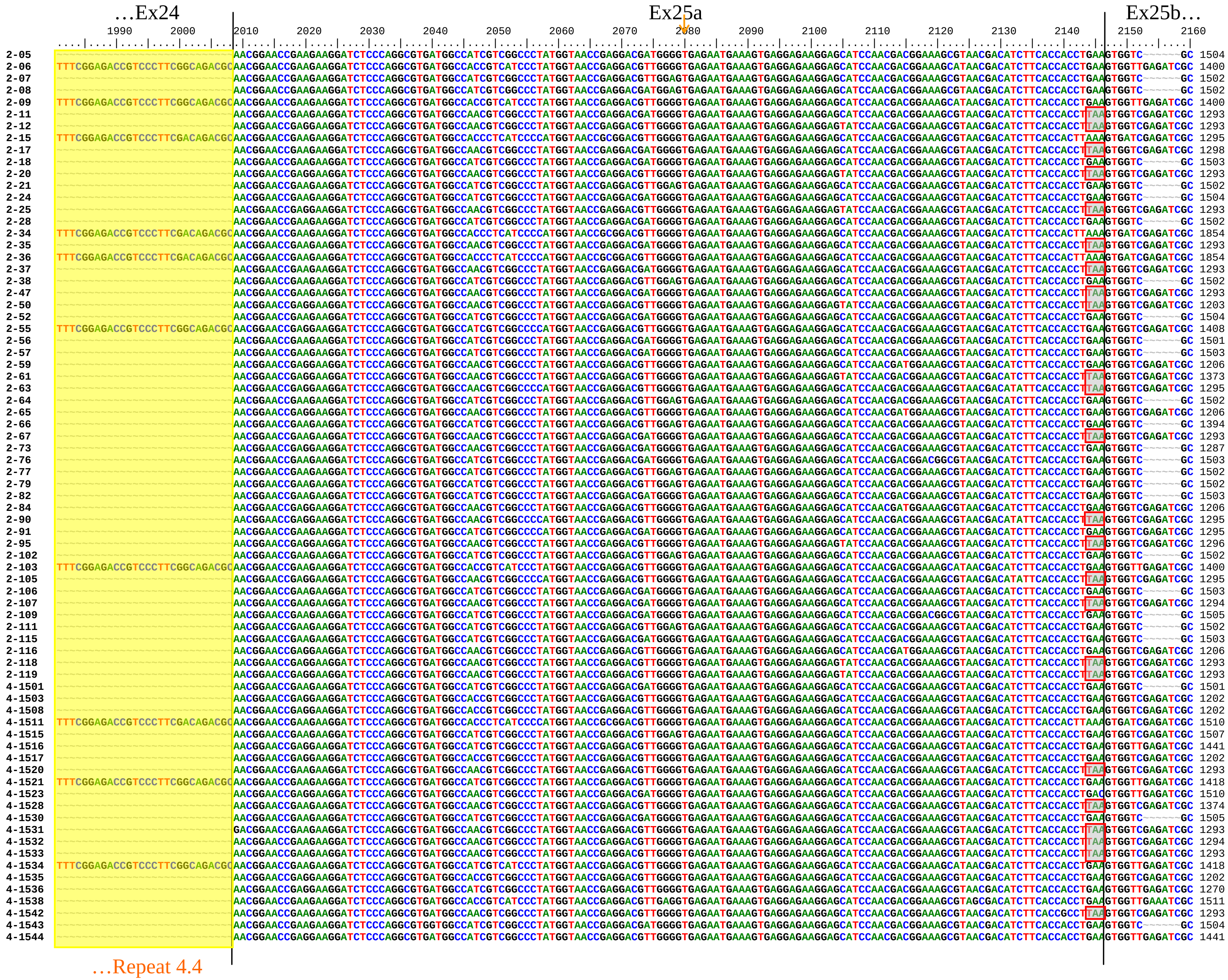

…Ex24
Ex25a
Ex25b…
1990
2000
2010
2020
2030
2040
2050
2060
2070
2080
2090
2100
2110
2120
2130
2140
2150
2160
…Repeat 4.4

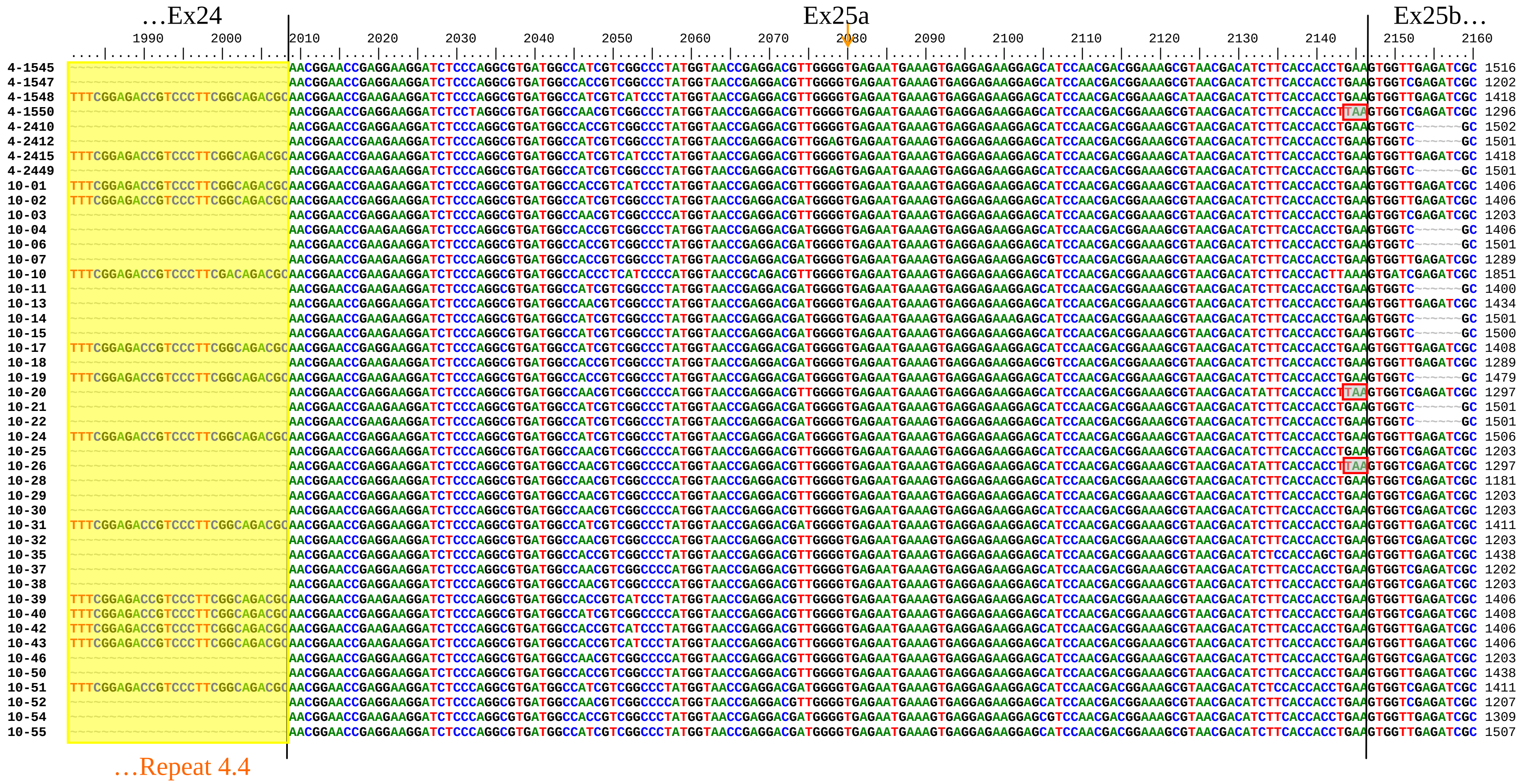
…Ex24
Ex25a
Ex25b…
…Repeat 4.4

...Ex25b

```
                2170      2180      2190      2200
        ....|....|....|....|....|....|....|....|
2-05    AATCAATGAAGAAGACATCAATGTGGTCGCCGAGGTGAAG 1544
2-06    AGTCAATGAAGAAGACGTCAATGTGGTCGCCGAGGTGTAA 1440
2-07    AATCAATGAAGAAGACATCAATGTGGTCGCCGAGGTGTAG 1542
2-08    AATCAATGAAGAAGACATCAATGTGGTCGCCGAGGTGTAG 1542
2-09    AGTCAATGAAGAAGACGTCAATGTGGTCGCCGAGGTGTAG 1440
2-11    AGTCAATGAAGAAGACATCAATGTGGTCGCCGAGGTGTAG 1333
2-12    GATC~~~~~~~~~~~~~~~AATGATGTCGCCGAGGTGTAG 1318
2-15    AATCAATGAAGTAGACACCAATGTGGTCGCCGAGGTGTAG 1335
2-17    AGTCAATGAAGAAGACATCAATGTGGTCGCCGAGGTGTAG 1338
2-18    AATCAATGAAGAAGACATCAATGTGGTCGCCGAGGTGTAA 1543
2-20    GATC~~~~~~~~~~~~~~~AATGATGTCGCCGAGGTGTAG 1318
2-21    AATCAATGAAGAAGACATCAATGTGGTCGCCGAGGTGTAG 1542
2-24    AATCAATGAAGAAGACATCAATGTGGTCGCCGAGGTGTAA 1544
2-25    GATC~~~~~~~~~~~~~~~AATGATGTCGCCGAGGTGTAG 1318
2-28    AATCAATGAAGAAGACATCAATGTGGTCGCCGAGGTGTAG 1542
2-34    AATCAATGAAGTAGACACCAATGTGGTCGCCGAGGTGTAG 1894
2-35    AGTCAATGAAGAAGACATCAATGTGGTCGCCGAGGTGTAG 1333
2-36    AATCAATGAAGTAGACACCAATGTGGTCGCCGAGGTGTAG 1894
2-37    AGTCAATGAAGAAGACATCAATGTGGTCGCCGAGGTGTAG 1333
2-38    AATCAATGAAGAAGACATCAATGTGGTCGCCGAGGTGTAG 1542
2-47    AGTCAATGAAGAAGACATCAATGTGGTCGCCGAGGTGTAG 1333
2-50    GATC~~~~~~~~~~~~~~~AATGATGTCGCCGAGGTGTAG 1228
2-52    AATCAATGAAGAAGACATCAATGTGGTCGCCGAGGTGTAG 1544
2-55    GATC~~~~~~~~~~~~~~~AATGATGTCGCCGAGGTGTAG 1433
2-56    AATCAATGAAGAAGACATCAATGTGGTCGCCGAGGTGTAG 1541
2-57    AATCAATGAAGAAGACATCAATGTGGTCGCCGAGGTGTAG 1543
2-59    GATC~~~~~~~~~~~~~~~AATGATGTCGCCGAGGTGTAG 1231
2-61    GATC~~~~~~~~~~~~~~~AATGATGTCGCCGAGGTGTAG 1398
2-63    AGTCAATGAAGAAGACATCAATGGGGTCGCCGAGGTGTAG 1335
2-64    AATCAATGAAGAAGACATCAATGTGGTCGCCGAGGTGTAG 1542
2-65    GATCAATGAAGTAGACACCAATGGGGTCGCCGAGGTGTAG 1246
2-66    AATCAATGAAGAAGACATCAATGTGGTCGCCGAGGTGTAG 1434
2-67    AGTCAATGAAGAAGACATCAATGTGGTCGCCGAGGTGTAG 1333
2-73    AATCAATGAAGAAGACATCAATGTGGTCGCCGAGGTGTAG 1327
2-76    AATCAATGAAGAAGACATCAATGTGGTCGCCGAGGTGTAG 1543
2-77    AATCAATGAAGAAGACATCAATGTGGTCGCCGAGGTGTAG 1542
2-79    AATCAATGAAGAAGACATCAATGTGGTCGCCGAGGTGTAG 1542
2-82    AATCAATGAAGAAGACATCAATGTGGTCGCCGAGGAGTAG 1543
2-84    GATC~~~~~~~~~~~~~~~AATGATGTCGCCGAGGTGTAG 1231
2-90    AGTCAATGAAGAAGACATCAATGGGGTCGCCGAGGTGTAT 1335
2-91    TGACAAT~~~~~~GACTACGACGTGGTCGCCGAGTCGTAT 1329
2-95    GATC~~~~~~~~~~~~~~~AATGATGTCGCCGAGGTGTAG 1321
2-102   AATCAATGAAGAAGACATCAATGTGGTCGCCGAGGTGTAG 1542
2-103   AGTCAATGAAGAAGACGTCAATGTGGTCGCCGAGGTGTAG 1440
2-105   AGTCAATGAAGAAGACATCAATGGGGTCGCCGAGGTGTAG 1335
2-106   AATCAATGAAGAAGACATCAATGTGGTCGCCGAGGTGTAG 1543
2-107   AGTCAATGAAGAAGACATCAATGTGGTCGCCGAGGTGTAG 1334
2-109   AATCAATGAAGAACACATCAATGTGGTCGCCGAGGTGTAG 1545
2-111   AATCAATGAAGAAGACATCAATGTGGTCGCCGAGGAGTAG 1542
2-115   AATCAATGAAGAAGACATCAATGTGGTCGCCGAGGTGTAG 1543
2-116   GATC~~~~~~~~~~~~~~~AATGATGTCGCCGAGGTGTAG 1231
2-118   GATC~~~~~~~~~~~~~~~AATGATGTCGCCGAGGTGTAG 1318
2-119   GATC~~~~~~~~~~~~~~~AATGATGTCGCCGAGGTGTAG 1318
4-1501  AATCAATGAAGAAGACATCAATGTGGTCGCCGAGGTGTAG 1541
4-1503  GATC~~~~~~~~~~~~~~~AATGATGTCGCCGAGGTGTAG 1227
4-1508  GATC~~~~~~~~~~~~~~~AATGATGTCGCCGAGGTGTAG 1227
4-1511  AATCAATGAAGTAGACACCAATGTGGTCGCCGAGGTGTAG 1550
4-1515  AGTCAATGAAGAAGACATCAATGTGGTCGCCGAGGTGTAG 1547
4-1516  ATTCAATGAAGAAGACGTCAATGTGGTCGCCGAGGTGTAG 1481
4-1517  GATC~~~~~~~~~~~~~~~AATGATGTCGCCGAGGTGTAG 1227
4-1520  AGTCAATGAAGAAGACATCAATGATGTCGCCGAGGTGTAG 1333
4-1521  AGTCAATGAAGAAGACGTCAATGTGGTCGCCGAGGTGTAG 1458
4-1523  AGTCAATGAAGAAGACGTCAATGTGGTCGCCGAGGTGTAG 1550
4-1528  AGTCAATGAAGAAGACATCAATGATGTCGCCGAGGTGTAG 1414
4-1530  AATCAATGAAGAAGACATCAATGTGGTCGCTGAGGTGTAG 1545
4-1531  AGTCAATGAAGAAGACATCAATGATGTCGCCGAGGTGTAG 1333
4-1532  AGTCAATGAAGAAGACATCAATGATGTCGCCGAGGTGTAG 1334
4-1533  AGTCAATGAAGAAGACATCAATGATGTCGCTGAGGTGTAG 1333
4-1534  AGTCAATGAAGAAGACGTCAATATGGTCGCCGAGGTGTAG 1458
4-1535  GATC~~~~~~~~~~~~~~~AATGAGGTCGCCGAGGTGTAG 1227
4-1536  AGTCAATGAAGAAGACGTCAATGTGGTCGCCGAGGTGTAG 1310
4-1538  AGTCAATGAAGAAGACGTCAATGTGGTCGCTGAGGTGTAG 1551
4-1542  AGTCAATGAAGAAGACATCAATGATGTCGCTGAGGTGTAG 1333
4-1543  AATCAATGAAGAAGACATCAATGTGGTCGCCGAGGTGTAG 1544
4-1544  AGTCAATGAAGAAGACGTCAATGTGGTCGCCGAGGTGTAG 1481
```

…Ex25b

```
                 2170      2180      2190      2200
          ....|....|....|....|....|....|....|....|
4-1545    AGTCAATGAAGAAGACGTCAATGTGGTCGCCGAGGTGTAG 1556
4-1547    GATC~~~~~~~~~~~~~~~AATGATGTCGCCGAGGTGTAG 1227
4-1548    AGTCAATGAAGAAGACGTCAATATGGTCGCCGAGGTGTAG 1458
4-1550    AGTCAATGAAGAAGACATCAATGTGGTCGCCGAGGTGTAG 1336
4-2410    AATCAATGAAGAAGACATCAATGTGGTCGCCGAGGTGTAG 1542
4-2412    AATCAATGAAGAAGACATCAATGTGGTCGCCGAGGTGTAG 1541
4-2415    AGTCAATGAAGAAGACGTCAATATGGTCGCCGAGGTGTAA 1458
4-2449    AATCAATGAAGAAGACATCAATGTGGTCGCCGAGGTGTAG 1541
10-01     AGTCAATGAAGAAGACGTCAATGTGGTCGCCGAGGTGTAG 1446
10-02     AGTCAATGAAGAAGACGTCAATGTGGTCGCCGAGGTGTAG 1446
10-03     GATC~~~~~~~~~~~~~~~AATGATGTCGCCGAGGTGTAG 1228
10-04     AATCAATGAAGAAGACATCAATGTGGTCGCCGAGGTGTAG 1446
10-06     AATCAATGAAGAAGACATCAATGTGGTCGCCGAGGTGTAG 1541
10-07     ATTCAATGAAGAAGACGTCCATGTGGTCGCCGAGGTGTAG 1329
10-10     AATCAATGAAGTAGACACCAATGTGGTCGCCGAGGTGTAG 1891
10-11     AATCAATGAAGAAGACATCAATGTGGTCGCCGAGGTGTAG 1440
10-13     AGTCAATGAAGAAGACGTCAATGTGGTCGCCGAGGTGTAG 1474
10-14     AATCAATGAAGAAGACATCAATGTGGTCGCCGAGGTGTAG 1541
10-15     AATCAATGAAGAAGACATCAATGTGGTCGCCGAGGTGTAG 1540
10-17     AGTCAATGAAGAAGACGTCAATGTGGTCGCCGAGGTGTAG 1448
10-18     ATTCAATGAAGAAGACGTCAATGTGGTCGCCGAGGTGTAG 1329
10-19     AATCAATGAAGAAGACATCAATGTGGTCGCCGAGGTGTAG 1519
10-20     AGTCAATGAAGAAGACATCAATGGGGTCGCCGAGGTGTAG 1337
10-21     AATCAATGAAGAAGACATCAATGTGGTCGCCGAGGTGTAG 1541
10-22     AATCAATGAAGAAGACATCAATGTGGTCGCCGAGGTGTAG 1541
10-24     AGTCAATGAAGAAGACGTCAATGTGGTCGCCGAGGTGTAG 1546
10-25     GATC~~~~~~~~~~~~~~~AATGATGTCGCCGAGGTGTAG 1228
10-26     AGTCAATGAAGAAGACATCAATGGGGTCGCCGAGGTGTAG 1337
10-28     GATC~~~~~~~~~~~~~~~AATGATGTCGCCGAGGTGTAG 1206
10-29     GATC~~~~~~~~~~~~~~~AATGATGTCGCCGAGGTGTAG 1228
10-30     GATC~~~~~~~~~~~~~~~AATGATGTCGCCGAGGTGTAG 1228
10-31     AGTCAATGAAGAAGACGTCAATGTGGTCGCCGAGGTGTAG 1451
10-32     GATC~~~~~~~~~~~~~~~AATGATGTCGCCGAGGTGTAG 1228
10-35     ATTCAATGAAGAAGACGTCAATGTGGTCGCCGAGGTGTAG 1478
10-37     GATC~~~~~~~~~~~~~~~AATGATGTCGCCGAGGTGTAG 1227
10-38     GATC~~~~~~~~~~~~~~~AATGATGTCGCCGAGGTGTAG 1228
10-39     AGTCAATGAAGAAGACGTCAATGTGGTCGCCGAGGTGTAG 1446
10-40     GATC~~~~~~~~~~~~~~~AATGATGTCGCCGAGGTGTAG 1433
10-42     AGTCAATGAAGAAGACGTCAATGTGGTCGCCGAGGTGTTA 1446
10-43     AGTCAATGAAGAAGACGTCAATGTGGTCGCCGAGGTGTTA 1446
10-46     GATC~~~~~~~~~~~~~~~AATGATGTGGCCGAGGTGTAG 1228
10-50     ATTCAATGAAGAAGACGTCAATGTGGTCGCCGAGGTGTAG 1478
10-51     AGTCAATGAAGAAGACGTCAATGTGGTCGCCGAGGTGTAG 1451
10-52     GATC~~~~~~~~~~~~~~~AATGATGTCGCCGAGGTGTAG 1232
10-54     ATTCAATGAAGAAGACGTCAATGTGGTCGCCGAGGTGTAG 1349
10-55     AGTCAATGAAGAAGACGTCAATGTGGTCGCCGAGGTGTAG 1547
```
