# Supplementary material for: Extraordinary diversity among members of the large gene family, 185/333, from the purple sea urchin, Strongylocentrotus purpuratus
Source: BMC Mol Biol. 2007 Aug 15;8:68. doi: 10.1186/1471-2199-8-68 (PMC1988830; doi:10.1186/1471-2199-8-68)
Supplement: Additional file 3 — cDNA-based amino acid alignment of the 185/333 genes. The cDNA-based amino acid alignment of the open reading frames of the 121 unique gene sequences showing the element borders and repeats. [file 1471-2199-8-68-S3.pdf]

Additional File 3: Alignment of the encoded amino acid sequences. Vertical black lines indicate the element edges. Elements are labeled above the sequence. Repeats are indicated by colored boxes, which each different color (shades of red = type 1; blue = type 2; green = type 3; yellow = type 4; and purple = type 5).

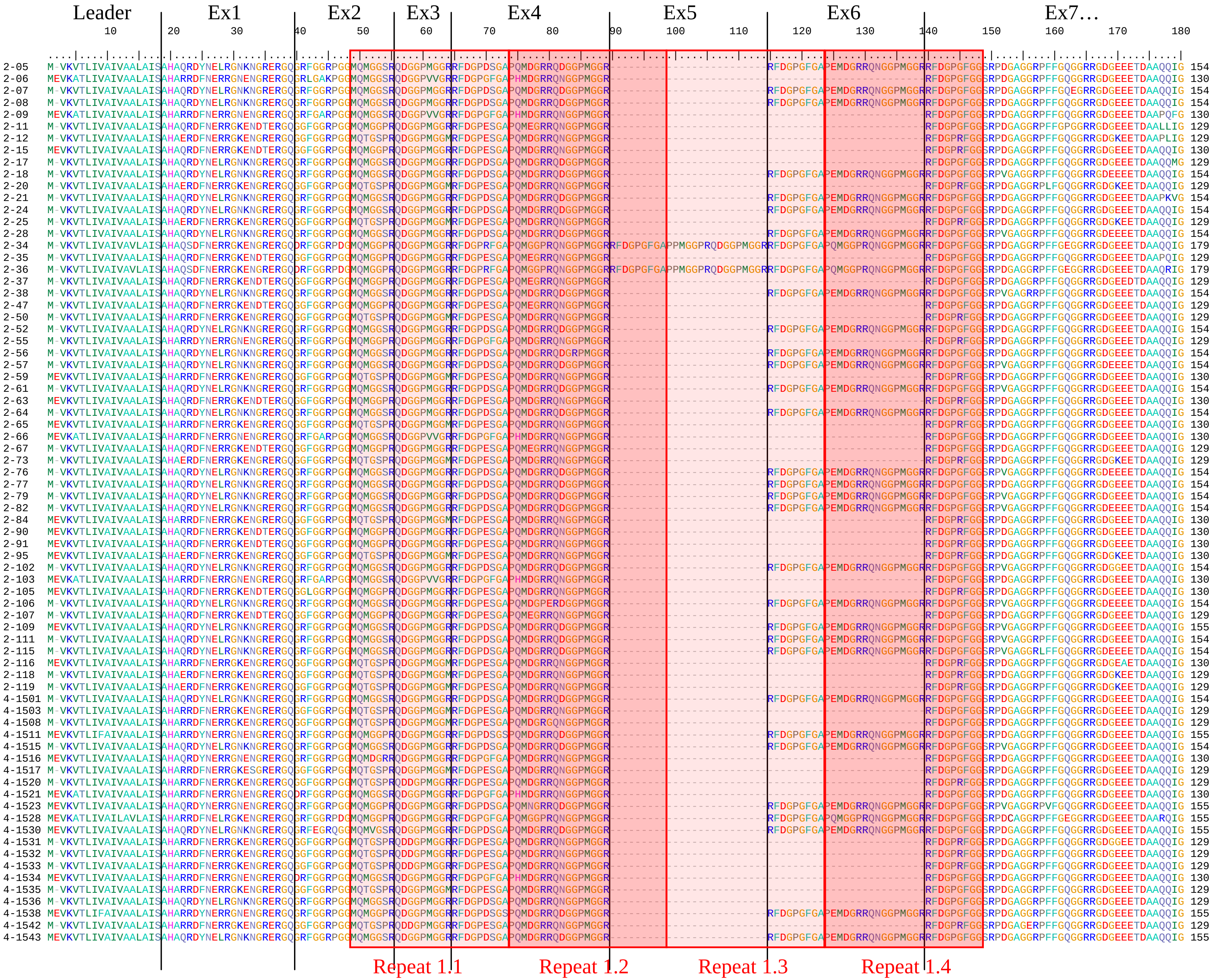
Leader
Ex1
Ex2
Ex3
Ex4
Ex5
Ex6
Ex7…
Repeat 1.1
Repeat 1.2
Repeat 1.3
Repeat 1.4

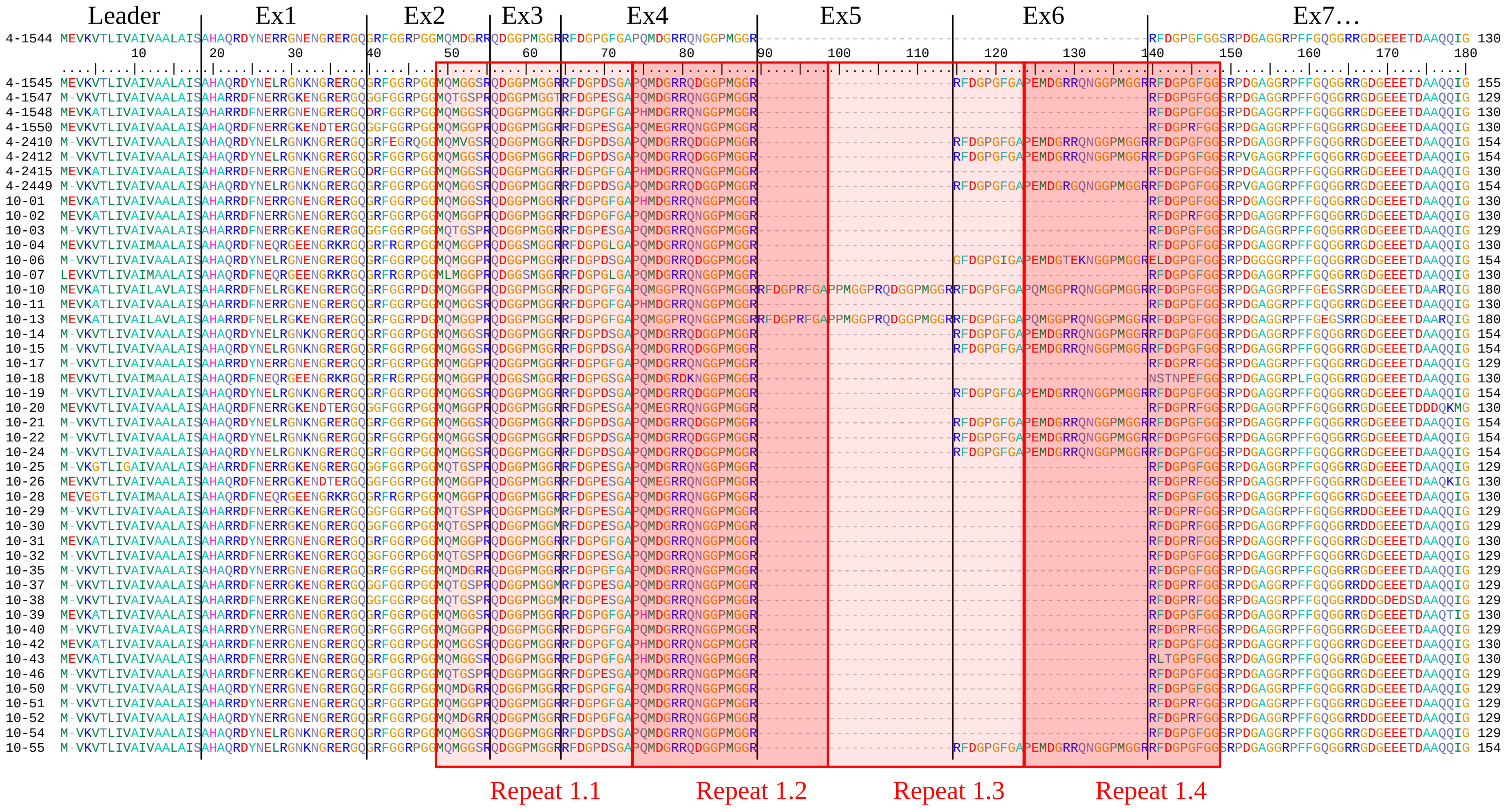

Leader
Ex1
Ex2
Ex3
Ex4
Ex5
Ex6
Ex7…
Repeat 1.1
Repeat 1.2
Repeat 1.3
Repeat 1.4

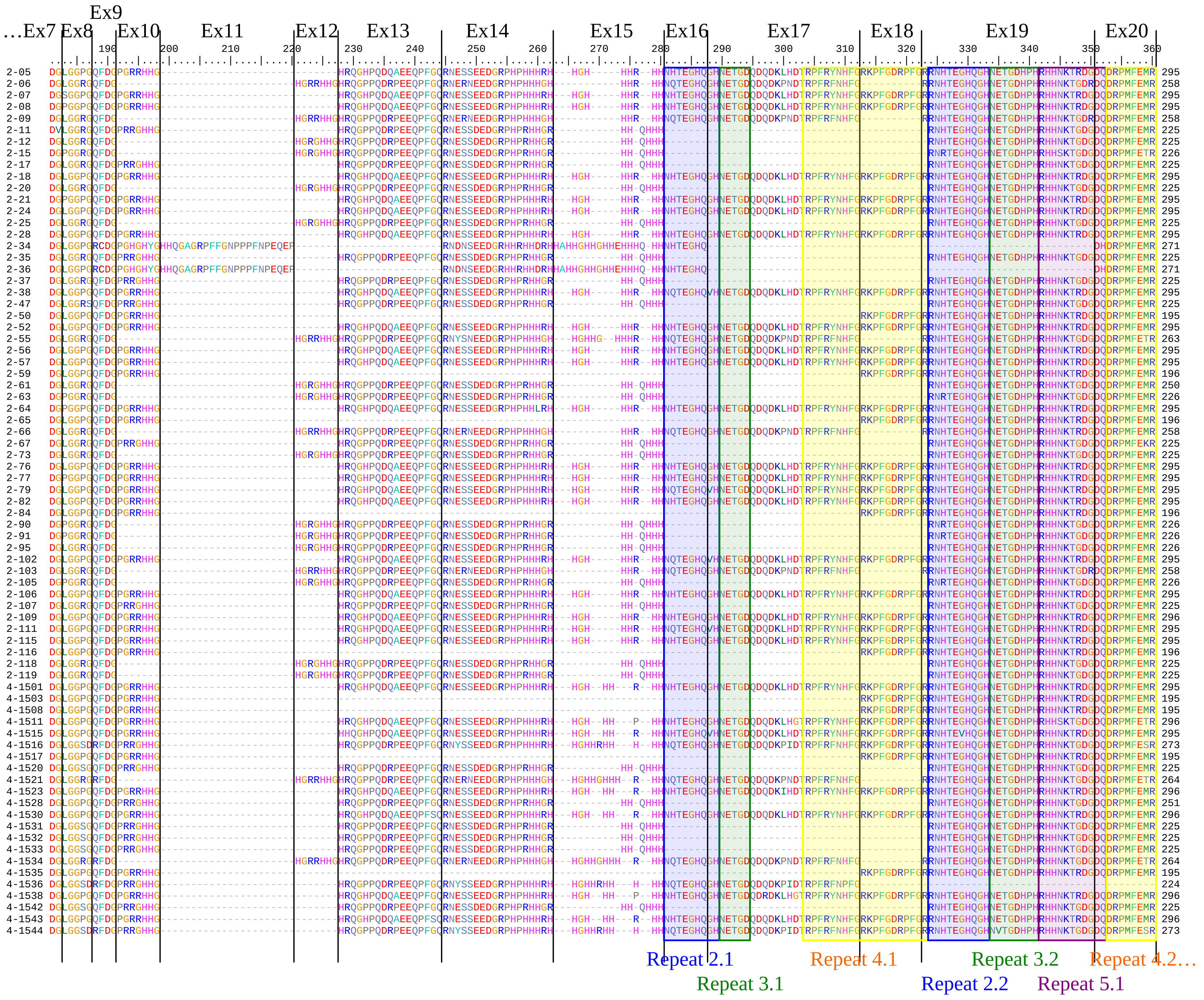

…Ex7 Ex8 Ex9 Ex10 Ex11 Ex12 Ex13 Ex14 Ex15 Ex16 Ex17 Ex18 Ex19 Ex20
Repeat 2.1
Repeat 3.1
Repeat 4.1
Repeat 2.2
Repeat 3.2
Repeat 5.1
Repeat 4.2…

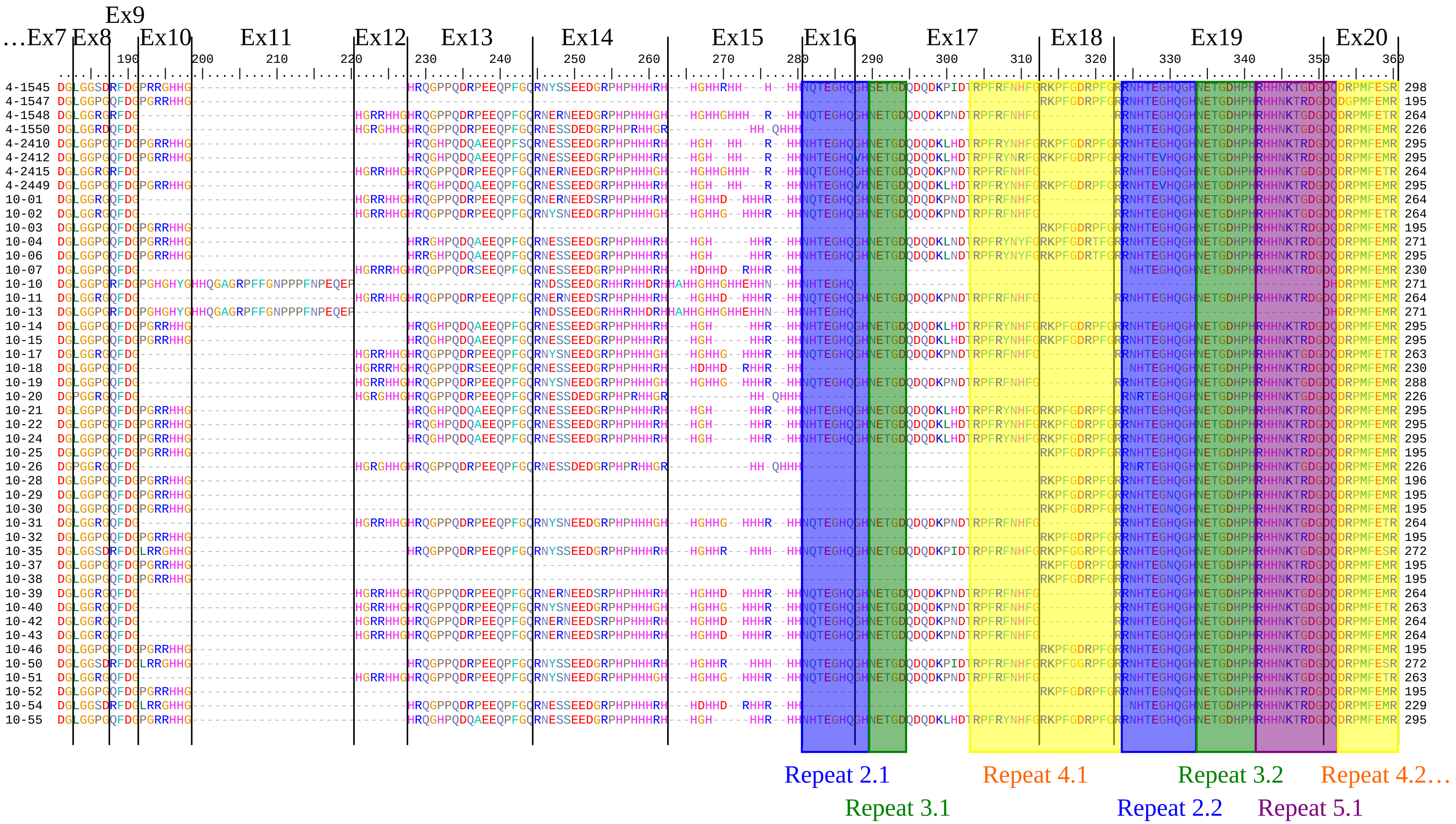
…Ex7
Ex8
Ex9
Ex10
Ex11
Ex12
Ex13
Ex14
Ex15
Ex16
Ex17
Ex18
Ex19
Ex20
Repeat 2.1
Repeat 3.1
Repeat 4.1
Repeat 2.2
Repeat 3.2
Repeat 5.1
Repeat 4.2…

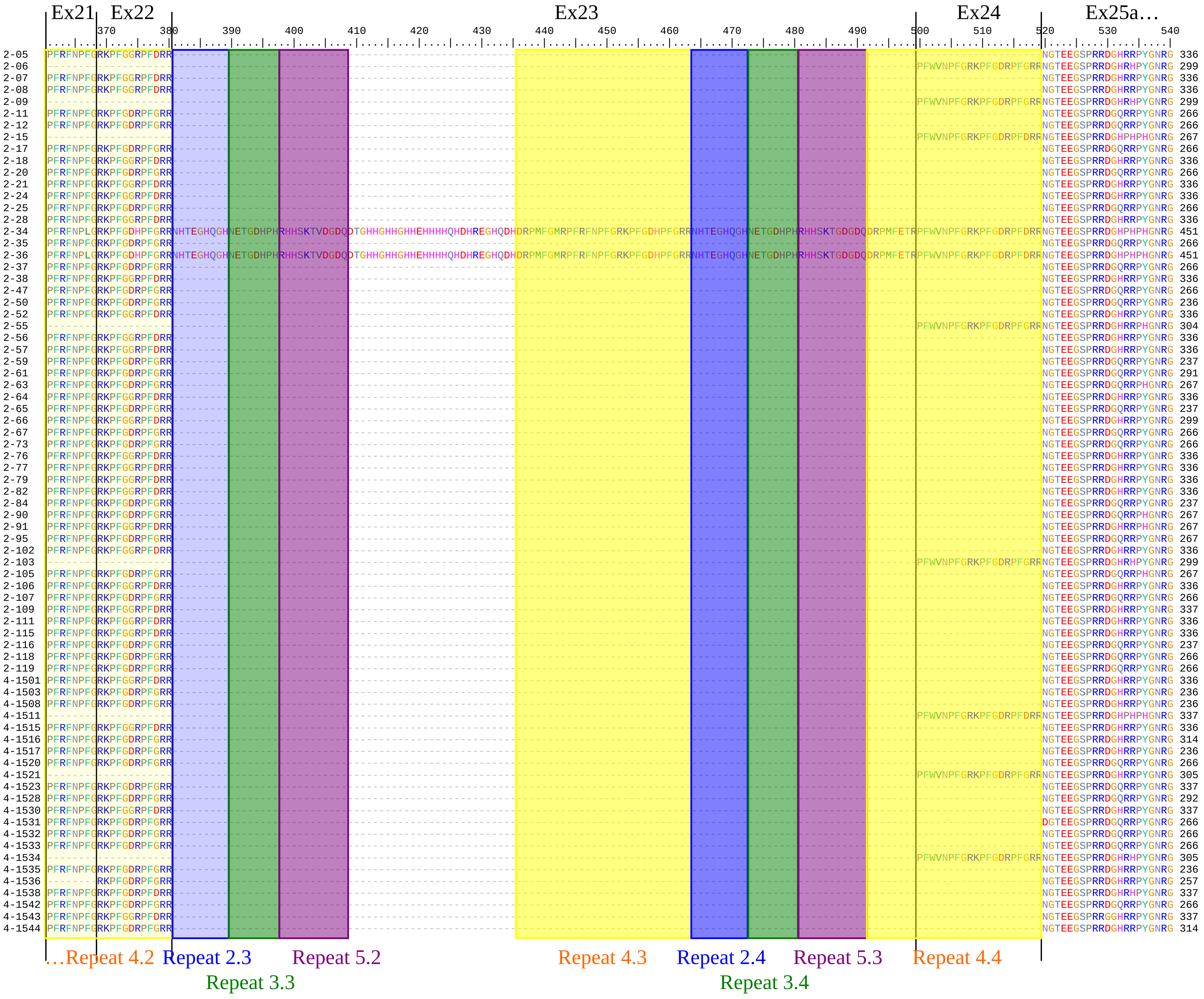

Ex21
Ex22
Ex23
Ex24
Ex25a…
370
380
390
400
410
420
430
440
450
460
470
480
490
500
510
520
530
540
…Repeat 4.2
Repeat 2.3
Repeat 3.3
Repeat 5.2
Repeat 4.3
Repeat 2.4
Repeat 3.4
Repeat 5.3
Repeat 4.4

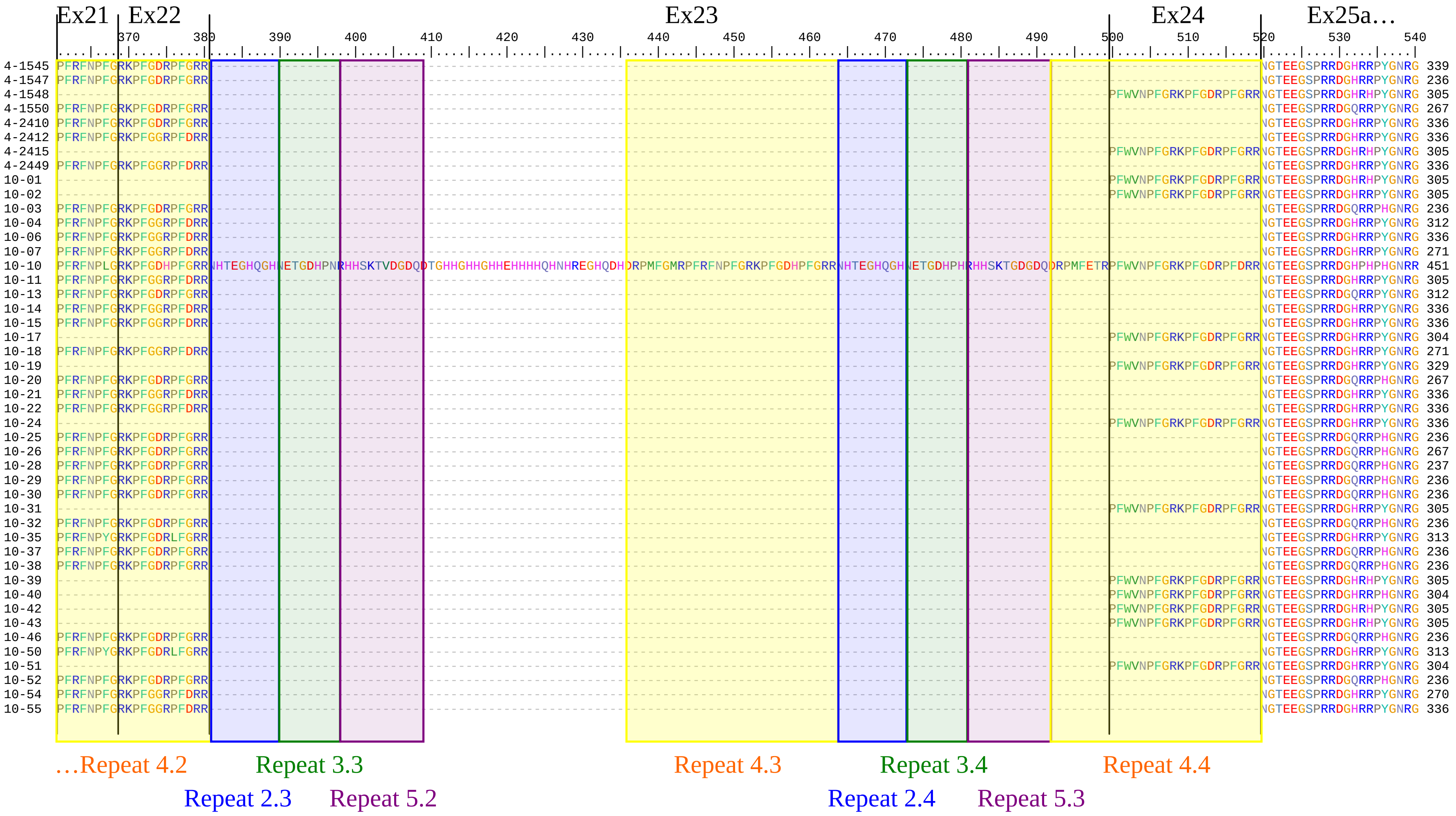
Ex21
Ex22
Ex23
Ex24
Ex25a…
…Repeat 4.2
Repeat 3.3
Repeat 2.3
Repeat 5.2
Repeat 4.3
Repeat 3.4
Repeat 2.4
Repeat 5.3
Repeat 4.4

```
              …Ex25a          Ex25b
                 550       560       570       580
         ....|....|....|....|....|....|....|....|...
2-05     RWGENESEEKEHPTTESVTTSSPPEVV--AINEEDINVVAEVK 377
2-06     RWGENESEEKEHPTTESITTSSPPEVVEIAVNEEDVNVVAEV* 342
2-07     RWSENESEEKEHPTTESVTTSSPPEVV--AINEEDINVVAEV* 377
2-08     RWSENESEEKEHPTTESVTTSSPPEVV--AINEEDINVVAEV* 377
2-09     RWGENESEEKEHPTTESITTSSPPEVVEIAVNEEDVNVVAEV* 342
2-11     RWGENESEEKEHPTTESVTTSSPP*VVEIAVNEEDINVVAEV* 309
2-12     RWGENESEEKEYPTTESVTTSSPP*VVEIAI-----NDVAEV* 304
2-15     RWGENESEEKEHPTTESVTTSSPLKVIEIAINEVDTNVVAEV* 310
2-17     RWGENESEEKEHPTTESVTTSSPP*VVEIAVNEEDINVVAEV* 309
2-18     RWGENESEEKEHPTTESVTTSSPPEVV--AINEEDINVVAEV* 377
2-20     RWGENESEEKEYPTTESVTTSSPP*VVEIAI-----NDVAEV* 304
2-21     RWSENESEEKEHPTTESVTTSSPPEVV--AINEEDINVVAEV* 377
2-24     RWGENESEEKEHPTTESVTTSSPPEVV--AINEEDINVVAEV* 377
2-25     RWGENESEEKEYPTTESVTTSSPP*VVEIAI-----NDVAEV* 304
2-28     RWGENESEEKEHPTTESVTTSSPPEVV--AINEEDINVVAEV* 377
2-34     RWGENESEEKEHPTTESVTTSSPLKVIEIAINEVDTNVVAEV* 494
2-35     RWGENESEEKEHPTTESVTTSSPP*VVEIAVNEEDINVVAEV* 309
2-36     RWGENESEEKEHPTTESVTTSSPLKVIEIAINEVDTNVVAEV* 494
2-37     RWGENESEEKEHPTTESVTTSSPP*VVEIAVNEEDINVVAEV* 309
2-38     RWSENESEEKEHPTTESVTTSSPPEVV--AINEEDINVVAEV* 377
2-47     RWGENESEEKEHPTTESVTTSSPP*VVEIAVNEEDINVVAEV* 309
2-50     RWGENESEEKEYPTTESVTTSSPP*VVEIAI-----NDVAEV* 274
2-52     RWGENESEEKEHPTTESVTTSSPPEVV--AINEEDINVVAEV* 377
2-55     RWGENESEEKEHPTTESVTTSSPPEVVEIAI-----NDVAEV* 342
2-56     RWGENESEEKEHPTTESVTTSSPPEVV--AINEEDINVVAEV* 377
2-57     RWGENESEEKEHPTTESVTTSSPPEVV--AINEEDINVVAEV* 377
2-59     RWGENESEEKEHPTMESVTTSSPPEVVEIAI-----NDVAEV* 275
2-61     RWGENESEEKEYPTTESVTTSSPP*VVEIAI-----NDVAEV* 329
2-63     RWGENESEEKEHPTTESVTTYSPP*VVEIAVNEEDINGVAEV* 310
2-64     RWSENESEEKEHPTTESVTTSSPPEVV--AINEEDINVVAEV* 377
2-65     RWGENESEEKEHPTMESVTTSSPPEVVEIAINEVDTNGVAEV* 280
2-66     RWSENESEEKEHPTTESVTTSSPPEVV--AINEEDINVVAEV* 340
2-67     RWGENESEEKEHPTTESVTTSSPP*VVEIAVNEEDINVVAEV* 309
2-73     RWGENESEEKEHPTTESVTTSSPPEVV--AINEEDINVVAEV* 307
2-76     RWGENESEEKEHPTTDGVTTSSPPEVV--AINEEDINVVAEV* 377
2-77     RWSENESEEKEHPTTESVTTSSPPEVV--AINEEDINVVAEV* 377
2-79     RWSENESEEKEHPTTESVTTSSPPEVV--AINEEDINVVAEV* 377
2-82     RWGENESEEKEHPTTESVTTSSPPEVV--AINEEDINVVAEE* 377
2-84     RWGENESEEKEHPTMESVTTSSPPEVVEIAI-----NDVAEV* 275
2-90     RWGENESEEKEHPTTESVTTYSPP*VVEIAVNEEDINGVAEVY 310
2-91     RWGENESEEKEHPTTESVTTSSPPEVVEIADN--DYDVVAESY 308
2-95     RWGENESEEKEYPTTESVTTSSPP*VVEIAI-----NDVAEV* 305
2-102    RWSENESEEKEHPTTESVTTSSPPEVV--AINEEDINVVAEV* 377
2-103    RWGENESEEKEHPTTESITTSSPPEVVEIAVNEEDVNVVAEV* 342
2-105    RWGENESEEKEHPTTESVTTYSPP*VVEIAVNEEDINGVAEV* 310
2-106    RWGENESEEKEHPTTESVTTSSPPEVV--AINEEDINVVAEV* 377
2-107    RWGENESEEKEHPTTESVTTSSPP*VVEIAVNEEDINVVAEV* 309
2-109    RWGENESEEKEHPTTDGVTTSSPPEVV--AINEEHINVVAEV* 378
2-111    RWSENESEEKEHPTTESVTTSSPPEVV--AINEEDINVVAEE* 377
2-115    RWGENESEEKEHPTTESVTTSSPPEVV--AINEEDINVVAEV* 377
2-116    RWGENESEEKEHPTMESVTTSSPPEVVEIAI-----NDVAEV* 275
2-118    RWGENESEEKEYPTTESVTTSSPP*VVEIAI-----NDVAEV* 304
2-119    RWGENESEEKEYPTTESVTTSSPP*VVEIAI-----NDVAEV* 304
4-1501   RWGENESEEKEHPTTESVTTSSPPEVV--AINEEDINVVAEV* 377
4-1503   RWGENESEEKEHPTTESVTTSSPPEVVEIAI-----NDVAEV* 274
4-1508   RWGENESEEKEHPTTESVTTSSPPEVVEIAI-----NDVAEV* 274
4-1511   RWGENESEEKEHPTTESVTTSSPLKVIEIAINEVDTNVVAEV* 380
4-1515   RWSENESEEKEHPTTESVTTSSPPEVVEIAVNEEDINVVAEV* 379
4-1516   RWGENESEEKEHPTTESVTTSSPPEVVEIAFNEEDVNVVAEV* 357
4-1517   RWGENESEEKEHPTTESVTTSSPPEVVEIAI-----NDVAEV* 274
4-1520   RWGENESEEKEHPTTESVTTSSPP*VVEIAVNEEDINDVAEV* 309
4-1521   RWGENESEEKEHPTTESVTTSSPPEVVEIAVNEEDVNVVAEV* 348
4-1523   RWGENESEEKEHPTTESVTTSSPPDVVEIAVNEEDVNVVAEV* 380
4-1528   RWGENESEEKEHPTTESVTTSSPP*VVEIAVNEEDINDVAEV* 335
4-1530   RWGENESEEKEHPTTESVTTSSPPEVV--AINEEDINVVAEV* 378
4-1531   RWGENESEEKEHPTTESVTTSSPP*VVEIAVNEEDINDVAEV* 309
4-1532   RWGENESEEKEHPTTESVTTSSPP*VVEIAVNEEDINDVAEV* 309
4-1533   RWGENESEEKEHPTTESVTTSSPP*VVEIAVNEEDINDVAEV* 309
4-1534   RWGENESEEKEHPTTESITTSSPPEVVEIAVNEEDVNMVAEV* 348
4-1535   RWGENESEEKEHPTTESVTTSSPPEVVEIAI-----NEVAEV* 274
4-1536   RWGENESEEKEHPTTESVTTSSPPEVVEIAVNEEDVNVVAEV* 300
4-1538   R*GENESEEKEHPTTESVATSSPPEVVEIAVNEEDVNVVAEV* 380
4-1542   RWGENESEEKEHPTTESVTTSSPP*VVEIAVNEEDINDVAEV* 309
4-1543   RWGENESEEKEHPTTESVTTSSPPEVV--AINEEDINVVAEV* 378
4-1544   RWGENESEEKEHPTTESVTTSSPPEVVEIAVNEEDVNVVAEV* 357
```

```
                   ...Ex25a      |    Ex25b
                550       560        570       580
         ....|....|....|....|....|....|....|....|...
4-1545 RWGENESEEKEHPTTESVTTSSPPEVVEIAVNEEDVNVVAEV* 382
4-1547 RWGENESEEKEHPTTESVTTSSPPEVVEIAI-----NDVAEV* 274
4-1548 RWGENESEEKEHPTTESITTSSPPEVVEIAVNEEDVNMVAEV* 348
4-1550 RWGENESEEKEHPTTESVTTSSPP*VVEIAVNEEDINVVAEV* 310
4-2410 RWGENESEEKEHPTTESVTTSSPPEVV--AINEEDINVVAEV* 377
4-2412 RWSENESEEKEHPTTESVTTSSPPEVV--AINEEDINVVAEV* 377
4-2415 RWGENESEEKEHPTTESITTSSPPEVVEIAVNEEDVNMVAEV* 348
4-2449 RWSENESEEKEHPTTESVTTSSPPEVV--AINEEDINVVAEV* 377
10-01  RWGENESEEKEHPTTESVTTSSPPEVVEIAVNEEDVNVVAEV* 348
10-02  RWGENESEEKEHPTTESVTTSSPPEVVEIAVNEEDVNVVAEV* 348
10-03  RWGENESEEKEHPTTESVTTSSPPEVVEIAI-----NDVAEV* 274
10-04  RWGENESEEKEHPTTESVTTSSPPEVV--AINEEDINVVAEV* 353
10-06  RWGENESEEKEHPTTESVTTSSPPEVV--AINEEDINVVAEV* 377
10-07  RWGENESEEKERPTTESVTTSSPPEVVEIAFNEEDVHVVAEV* 314
10-10  RWGENESEEKEHPTTESVTTSSPLKVIEIAINEVDTNVVAEV* 494
10-11  RWGENESEEKEHPTTESVTTSSPPEVV--AINEEDINVVAEV* 346
10-13  RWGENESEEKEHPTTESVTTSSPPEVVEIAVNEEDVNVVAEV* 355
10-14  RWGENESEEKEHPTTESVTTSSPPEVV--AINEEDINVVAEV* 377
10-15  RWGENESEEKEHPTTESVTTSSPPEVV--AINEEDINVVAEV* 377
10-17  RWGENESEEKEHPTTESVTTSSPPEVVEIAVNEEDVNVVAEV* 347
10-18  RWGENESEEKERPTTESVTTSSPPEVVEIAFNEEDVNVVAEV* 314
10-19  RWGENESEEKEHPTTESVTTSSPPEVV--AINEEDINVVAEV* 370
10-20  RWGENESEEKEHPTTESVTTYSPP*VVEIAVNEEDINGVAEV* 310
10-21  RWGENESEEKEHPTTESVTTSSPPEVV--AINEEDINVVAEV* 377
10-22  RWGENESEEKEHPTTESVTTSSPPEVV--AINEEDINVVAEV* 377
10-24  RWGENESEEKEHPTTESVTTSSPPEVVEIAVNEEDVNVVAEV* 379
10-25  RWGENESEEKEHPTTESVTTSSPPEVVEIAI-----NDVAEV* 274
10-26  RWGENESEEKEHPTTESVTTYSPP*VVEIAVNEEDINGVAEV* 310
10-28  RWGENESEEKEHPTTESVTTSSPPEVVEIAI-----NDVAEV* 275
10-29  RWGENESEEKEHPTTESVTTSSPPEVVEIAI-----NDVAEV* 274
10-30  RWGENESEEKEHPTTESVTTSSPPEVVEIAI-----NDVAEV* 274
10-31  RWGENESEEKEHPTTESVTTSSPPEVVEIAVNEEDVNVVAEV* 348
10-32  RWGENESEEKEHPTTESVTTSSPPEVVEIAI-----NDVAEV* 274
10-35  RWGENESEEKEHPTTESVTTSPPAEVVEIAFNEEDVNVVAEV* 356
10-37  RWGENESEEKEHPTTESVTTSSPPEVVEIAI-----NDVAEV* 274
10-38  RWGENESEEKEHPTTESVTTSSPPEVVEIAI-----NDVAEV* 274
10-39  RWGENESEEKEHPTTESVTTSSPPEVVEIAVNEEDVNVVAEV* 348
10-40  RWGENESEEKEHPTTESVTTSSPPEVVEIAI-----NDVAEV* 342
10-42  RWGENESEEKEHPTTESVTTSSPPEVVEIAVNEEDVNVVAEVL 348
10-43  RWGENESEEKEHPTTESVTTSSPPEVVEIAVNEEDVNVVAEVL 348
10-46  RWGENESEEKEHPTTESVTTSSPPEVVEIAI-----NDVAEV* 274
10-50  RWGENESEEKEHPTTESVTTSSPPEVVEIAFNEEDVNVVAEV* 356
10-51  RWGENESEEKEHPTTESVTTSPPPEVVEIAVNEEDVNVVAEV* 347
10-52  RWGENESEEKEHPTTESVTTSSPPEVVEIAI-----NDVAEV* 274
10-54  RWGENESEEKERPTTESVTTSSPPEVVEIAFNEEDVNVVAEV* 313
10-55  RWGENESEEKEHPTTESVTTSSPPEVVEIAVNEEDVNVVAEV* 379
```
