# Supplementary material for: Extraordinary diversity among members of the large gene family, 185/333, from the purple sea urchin, Strongylocentrotus purpuratus
Source: BMC Mol Biol. 2007 Aug 15;8:68. doi: 10.1186/1471-2199-8-68 (PMC1988830; doi:10.1186/1471-2199-8-68)
Supplement: Additional file 4 — Characteristics of the elements. The lengths, frequencies, and diversity characteristics of the elements from both the cDNA-based and repeat-based alignments. [file 1471-2199-8-68-S4.pdf]

Additional File 4: Characteristics of the elements from the cDNA-based and repeat-based alignments.

Element	Length (nt)	#	Diversity		Variable Positions (%)			States per variable position (average)		Unique Sequences		Unshared Polymorphisms	
			Nt	Aa	Ratio	Nt	Aa	Nt	Aa	Nt	Aa	Nt	Aa
**Leader**	55	121	0.0840	0.0949	1.13	25.5	36.8	2.14	2.43	14	10	4	3
**Ex1**	63	121	0.0893	0.2268	2.54	23.8	47.6	2.13	2.3	15	12	1	1
**Ex2**	48	121	0.1364	0.2420	1.77	45.8	81.3	2.09	2.23	16	14	3	2
**Ex3**	27	121	0.1029	0.1193	1.16	44.4	55.6	2.08	2.20	9	6	1	1
**Ex4**	75	121	0.1278	0.1526	1.19	38.7	40.0	2.10	2.80	21	15	6	7
**Ex5**	75	4	0.0185	0.0277	1.50	2.7	4.0	2.00	2.00	2	2	0	0
**Ex6**	75	46	0.0431	0.0663	1.54	17.3	28.0	2.00	2.29	6	4	5	6
**Ex7**	129	121	0.0539	0.0929	1.72	38.8	60.5	2.28	2.58	42	33	37	24
**Ex8**	15	121	0.1216	0.3132	2.58	53.3	60.0	2.00	3.00	10	9	3	2
**Ex9**	12	121	0.0587	0.1147	1.95	33.3	50.0	2.00	2.00	5	3	1	0
**Ex10**	21	83	0.0807	0.2422	3.00	23.8	71.4	2.00	2.00	4	4	0	0
**Ex11**	66	4	0.0000	0.0000	n/a	n/a	n/a	n/a	n/a	1	1	0	0
**Ex12**	21	38	0.0766	0.1267	1.65	19.1	28.6	2.00	2.00	4	3	0	0
**Ex13**	51	97	0.0799	0.1416	1.77	21.6	35.3	2.09	2.17	8	6	1	1
**Ex14**	54	101	0.1058	0.2643	2.50	29.6	61.1	2.00	2.27	10	9	1	1
**Ex15**	54	101	0.4702	0.4977	1.06	88.9	88.9	2.25	2.50	11	12	0	0
**Ex15a**	51	2	0.0000	0.0000	n/a	n/a	n/a	n/a	n/a	1	1	0	0
**Ex15b**	33	19	0.0471	0.1412	3.00	9.1	27.3	2.00	2.00	3	2	0	0
**Ex15c**	30	6	0.0000	0.0000	n/a	n/a	n/a	n/a	n/a	1	1	0	0
**Ex15d**	24	43	0.0078	0.0235	3.00	4.2	12.5	2.00	2.00	2	2	0	0
**Ex15e**	18	32	0.0292	0.0000	0.00	5.6	0.0	2.00	0.00	2	1	0	0
**Ex15f**	15	5	0.0000	0.0000	n/a	n/a	n/a	n/a	n/a	1	1	0	0
**Ex15g**	48	2	0.0000	0.0000	n/a	n/a	n/a	n/a	n/a	1	1	0	0
**Ex16**	21	69	0.0718	0.0978	1.36	14.3	14.3	2.00	2.00	4	2	0	0
**Ex17**	75	65	0.0714	0.1303	1.82	21.3	32.0	2.06	2.50	13	11	5	5
**Ex18**	30	64	0.0139	0.0278	2.00	10.0	20.0	2.00	2.00	3	3	0	0
**Ex19**	84	116	0.0935	0.0779	0.83	25.0	32.1	2.05	2.00	16	11	1	1
**Ex20**	30	120	0.0619	0.0735	1.19	20.0	30.0	2.33	2.67	8	6	2	2

Element	Length (nt)	#	Diversity		Variable Positions (%)			States per variable position (average)		Unique Sequences		Unshared Polymorphisms	
			Nt	Aa	Ratio	Nt	Aa	Nt	Aa	Nt	Aa	Nt	Aa
**Ex21**	24	99	0.0491	0.0292	0.59	16.7	12.5	2.25	3.00	6	3	1	0
**Ex22**	36	100	0.0510	0.1319	2.59	13.9	33.3	2.00	2.00	7	5	0	0
**Ex23**	357	3	0.0036	0.0107	2.97	0.6	1.7	2.00	2.00	2	2	2	2
**Ex24**	60	24	0.0085	0.0256	3.00	1.7	5.0	2.00	2.00	2	2	0	0
**Ex25**	192	121	0.1266	0.1818	1.44	34.9	50.0	2.22	2.56	53	38	23	15
**Ex25a**	138	121	0.0495	0.0949	1.92	21.0	41.3	2.07	2.21	33	21	10	7
**Ex25b**	54	121	0.3237	0.4039	1.25	70.4	72.2	2.34	3.08	26	18	13	8
**Er1**	92	121	0.0888	0.2058	2.32	29.4	58.1	2.07	2.22	21	17	3	2
**Er2**	75	121	0.1272	0.2937	2.31	42.7	76.0	2.09	2.37	24	21	4	2
**Er3**	75	46	0.0514	0.1145	2.23	22.7	40.0	2.00	2.30	7	7	8	6
**Er4**	75	4	0.0092	0.0277	3.01	1.3	4.0	2.00	2.00	2	2	0	0
**Er5**	76	121	0.0886	0.2125	2.40	40.8	65.4	2.16	2.77	19	19	16	17
**Er6**	147	121	0.0715	0.1468	2.05	38.8	65.3	2.23	2.50	50	44	32	21
**Er7**	66	4	0.0000	0.0000	n/a	n/a	n/a	n/a	n/a	1	1	0	0
**Er8**	51	97	0.0799	0.1416	1.77	21.6	35.3	2.09	2.17	8	6	1	1
**Er9**	45	101	0.0935	0.2167	2.32	26.7	53.3	2.00	2.25	9	8	0	0
**Er10**	84	101	0.2554	0.3463	1.36	58.3	64.3	2.23	2.61	12	11	1	1
**Er10a**	60	2	0.0000	0.0000	n/a	n/a	n/a	n/a	n/a	1	1	0	0
**Er10b**	43	19	0.0621	0.1198	1.93	11.6	0.2	2.00	2.00	11	11	0	0
**Er10c**	39	6	0.0000	0.0000	n/a	n/a	n/a	n/a	n/a	1	1	0	0
**Er10d**	33	39	0.0097	0.0292	3.01	6.06	18.2	2.00	2.00	36	36	1	1
**Er10e**	33	39	0.1172	0.1005	0.85	21.2	18.2	2.00	2.00	22	22	0	0
**Er10f**	24	4	0.0000	0.0000	n/a	n/a	n/a	n/a	n/a	1	1	0	0
**Er10g**	57	2	0.0000	0.0000	n/a	n/a	n/a	n/a	n/a	1	1	0	0
**Er11**	21	101	0.0860	0.1192	1.39	28.6	14.3	2.00	3.00	6	3	1	0
**Er12**	88	3	0.0000	0.0000	n/a	n/a	n/a	n/a	n/a	1	1	0	0
**Er13**	23	3	0.0000	0.0000	n/a	n/a	n/a	n/a	n/a	1	1	0	0
**Er14**	23	100	0.0723	0.0387	0.54	21.7	25.0	2.00	2.00	5	3	1	1
**Er15**	33	3	0.0193	0.0579	3.00	3.0	9.1	2.00	2.00	2	1	1	1
**Er16**	11	68	0.0070	0.0000	0	9.1	0.0	2.00	0.00	2	1	1	0

Element	Length (nt)	#	Diversity		Variable Positions (%)			States per variable position (average)		Unique Sequences		Unshared Polymorphisms	
			Nt	Aa	Ratio	Nt	Aa	Nt	Aa	Nt	Aa	Nt	Aa
**Er17**	14	65	0.1785	0.2638	1.48	42.9	80.0	2.00	2.25	6	6	1	1
**Er18**	49	3	0.0000	0.0000	n/a	n/a	n/a	n/a	n/a	1	1	0	0
**Er19**	22	3	0.0289	0.0000	0.00	4.6	0.0	2.00	0.00	2	1	1	0
**Er20**	27	3	0.0000	0.0000	n/a	n/a	n/a	n/a	n/a	1	1	0	0
**Er21**	26	67	0.0602	0.1191	1.98	19.2	22.2	2.20	3.00	7	5	1	1
**Er22**	30	67	0.0398	0.0878	2.21	16.7	30.0	2.00	2.00	5	4	0	0
**Er23**	28	87	0.0346	0.0970	2.80	10.7	30.0	2.00	2.00	4	4	0	0
**Er24**	23	87	0.0300	0.0078	0.26	8.7	12.5	2.00	2.00	3	2	1	1
**Er25**	34	119	0.0513	0.1309	2.56	14.7	33.3	2.00	2.00	8	6	0	0
**Er26**	90	121	0.0728	0.1192	1.64	42.2	50.0	2.21	2.33	22	12	33	12
**Er27**	192	121	0.1277	0.1855	1.45	35.4	50.0	2.28	2.59	56	40	23	14
**Er27a**	138	121	0.0495	0.0949	1.92	21.0	41.3	2.07	2.21	33	21	10	7
**Er27b**	54	121	0.3276	0.4168	1.27	72.2	72.2	2.44	3.15	27	18	13	7
