# Supplementary material for: Extraordinary diversity among members of the large gene family, 185/333, from the purple sea urchin, Strongylocentrotus purpuratus
Source: BMC Mol Biol. 2007 Aug 15;8:68. doi: 10.1186/1471-2199-8-68 (PMC1988830; doi:10.1186/1471-2199-8-68)
Supplement: Additional file 5 — Diversity controls. A description of the control experiments performed to eliminate the possibility that the observed 185/333 gene diversity was the result of either artificial Taq-induced errors or template switching. [file 1471-2199-8-68-S5.pdf]

Extraordinary diversity among members of the large gene family, *185/333*, from the purple sea urchin, *Strongylocentrotus purpuratus*

Additional File 5: Diversity Controls

K.M. Buckley, L.C. Smith

Introduction

The extraordinarily high level of diversity observed among the *185/333* genes led us to investigate whether some of the diversity was the result of artifacts introduced during PCR amplification. There were two possible artificial sources of diversity: (1) *Taq*-induced nucleotide misincorporation during either the PCR amplification and/or cycle sequencing [1], and (2) template switching that occurred during PCR amplification of the *185/333* genes from genomic DNA (gDNA) [2, 3]. *Taq*-induced errors would result in an increased number of random single nucleotide polymorphisms (SNPs) [1], whereas template switching would generate recombinant sequences [2, 3]. Because SNPs were observed and possible recombinant sequences could not be ruled out, it was important to understand the effect that these two processes might have on the observed gene diversity.

Although the published error rates of *Taq* polymerase are low, data indicates that the polymerase occasionally introduces artificial SNPs during PCR amplification [1]. GC-rich stretches or regions of complex secondary structure may decrease the *Taq* fidelity, which means that understanding the error rate for a particular sequence may be important. Due to the high diversity of the *185/333* sequences and the frequency with which SNPs were observed, it was

important to analyze the putative role of introducing errors given the polymerase, amplification conditions, and template sequences used in this study.

Template switching is a phenomenon in which recombinant DNA molecules are generated as artifacts during PCR when multiple homologous templates are present [2, 3]. Because the PCR amplification of genomic DNA used to generate amplicons for cloning would contain multiple homologous templates (i.e., the various members of the *185/333* gene family are $\geq$ 87% identical at the nucleotide level), it was important to determine whether template switching might occur and artificially elevate diversity. This phenomenon was previously analyzed in the genes encoding the fibrinogen-related proteins (FREPs) expressed in freshwater snails infected with trematode worms [4]. The FREP genes are members of a multigene family with at least 13 subfamilies and appear to undergo somatic diversification by mechanisms that appear to include gene conversion [4]. FREP proteins are composed of one or two variable immunoglobulin super family (IgSF) domains linked to a relatively conserved fibrinogen domain. To eliminate template switching artifacts, a mixture of three IgSF domains from the FREP genes was used as the template in a PCR reaction from which the fragments were subsequently cloned and sequenced. From 88 sequenced clones, no evidence of template switching was found [4]. However, because the frequency of template switching may be related to specific sequences, it was important to determine the possible effect that template switching might have during *185/333* gene amplification. To answer this question, a PCR approach was emplyed in which the conditions used during the amplification and cloning of *185/333* genes were mimicked as closely as possible and the rate at which recombinant amplicons were generated was monitored.

Methods

Taq-induced misincorporation

PCR Control: To determine the fidelity of the *Optimase* DNA polymerase (Transgenomic, Inc.), a single *185/333* cDNA (*Sp0228*, GenBank Acc. #DQ183174; [5]) was amplified in a PCR reaction using primers 185-5'UTR and 185-3'UTR (see Table 1) under conditions identical to those used to amplify the genomic DNA from animals 2 and 10 and to clone the *185/333* genes (see Materials & Methods).

Sequencing Control: A single Puc19 control DNA clone (Beckman Coulter) was sequenced in 38 reactions as described above, for a total of 18,911 bases.

Template Switching

To generate a pool of homologous amplicons, PCR amplification was performed using 100 pg of the following clones as templates: 2-013 and 2-103; 2-013 alone; 2-103 alone; 2-013 and actin; 2-103 and actin; and actin alone (see Additional File 1 for *185/333* clone information and accession numbers). The actin clone was generated by amplifying gDNA (isolated as previously described, [5, 6]) for the CyI gene (CyI coding region Genbank acc. # XM_001178271; CyI 3'UTR Genbank acc. #X00319; [6]). The CyF primer (ACG ACG ATG TTG CCC CTC TTG TCA T) annealed in the coding region, and the CyIR primer (CGG TAA AGT GGT CAC CAA TAC TAG CAC) annealed in the CyI 3' UTR. The amplified product was cloned into the pCR4-TOPO vector (Invitrogen) according to the manufacturer's instructions. The two *185/333* clones (Additional File 1), which are patterns D1 and F1, respectively (Figure 2; Additional Files 1, 2, and 3) are 95% similar at the nucleotide level and

are oriented in opposite directions within the pCR4-TOPO vector. The actin sequence was not homologous to the *185/333* sequences.

The templates were initially amplified using the M13 primer pair. The reaction mixtures included 1X company-supplied buffer (Stratagene; La Jolla, CA), 50 μM each dNTP, 175 nM each primer (M13F: GTA AAC GAC GGC CAG T and M13R: CAG AAA CAG CTA TGA C), 200 pg template DNA, and 1 U *Paq5000* DNA Polymerase (Stratagene) and were run in an Eppendorf Mastercycler under the following conditions: 95°C for 3 minutes, followed by 25 cycles of 95°C for 20 seconds, 55°C for 20 seconds and 72°C for 1 minute, and a final extension of 72°C for 1 minute. When two template clones were present in a reaction, 100 pg of each clone was used. The reactions were cleaned using the Wizard SV Gel and PCR Clean-up System (Promega) and DNA concentration was determined on a Beckman DU640 spectrophotometer (Beckman Coulter).

Based on our estimate of 5.75 x 10^{6} templates in the original PCR (starting amount of 100 ng of gDNA, a genome weight of 1.78 pg per diploid genome [7], and our estimate of 100 alleles per genome [5]), 12.6 pg of cleaned amplicons were used as the template in a second round of PCR. This reaction mixture contained 1X company supplied buffer (Takara Mirius Bio), 50 μM each dNTP, 181 nM ^{32}P-dCTP (800 Ci/mmol; Perkin Elmer, Boston MA), 1 μM of either T3L primer (CAA GCT CAG AAT TAA CCC TCA CTA AAG G) or T7L primer (CAA TTC GCC CTA TAG TGA GTC GTA TTA CAA TTC A), and 2.5 U *ExTaq* DNA Polymerase (Takara Mirius Bio). The positive control reaction contained 1 μM each T3L and T7L, and the negative control lacked both primers. Reactions were assembled and run in triplicate. Amplifications were done in an Eppendorf Mastercycler with the following conditions: 95°C for 3 minutes followed by 25 cycles of 95°C for 30 seconds, 68°C for 30 seconds, 72°C for 2

minutes, and a final extension of 72°C for 3 minutes. Unincorporated ^{32}P-dCTP was removed by passing the samples through a G50 Sephadex spin column (Amersham Biosciences, Piscataway, N.J.). The entire flow-through from each column was counted for 30 minutes in a LS6500 liquid scintillation counter (Beckman Coulter) to detect ^{32}P-dCTP incorporation. Duplicate reactions without ^{32}P-dCTP were analyzed on a 1% agarose gel to confirm that the product was the correct size.

Results

Taq-induced misincorporation

Animals 2 and 10: The *185/333* genes from animals 2 and 10 were amplified using *Optimase* DNA polymerase. To determine the fidelity of the *Optimase* DNA polymerase, a single *185/333* clone was amplified by PCR, cloned and sequenced. In total, 20 randomly selected clones were sequenced, providing a total of 28,417 bp. No errors were observed among the 28,417 bp, indicating that the error rate was < 1 in 28,417. The published error rate of *Optimase* is 1 error per 1.3 x 10^6 nt. The cloned genes from animals 2 and 10 (n = 137) totaled 194,597 nt. Therefore, using the published error rate of the polymerase, no errors are expected. However, using the maximum error rate as determined by multiple cloning of *Sp0228,* at most, 6.8 *Taq*-induced errors or 1 error in 20 clones might be expected.

Animal 4: *185/333* genes from animal 4 were amplified using *Ex Taq* DNA polymerase (Takara Mirius Bio). As previously determined [8], the error rate of this polymerase was 1 in 18,228 nt using *185/333* sequence. There were 34 *185/333* genes cloned and sequenced from animal 4, which totaled 42,663 nt. Therefore, 2.3 *Taq*-induced errors, (1 error in 15 clones) were expected.

Template Switching

No directionality was imposed on the inserts when the amplified *185/333* genes were ligated into the pCR4-TOPO vector, and therefore inserts were oriented in both orientations. The cloning site of the pCR4-TOPO vector (Invitrogen) is flanked a pair of primer sites located near the insert (called the “inner” sites to which T3 and T7 anneal) and by a different pair of primer sites that are located farther away from the cloning site (the “outer” sites to which M13F and M13R anneal; Figure 1). To generate a pool of homologous fragments, clones were first amplified using the outer primers. This pool of fragments was then used as the template in a second round of PCR amplification in which only one of the inner primers was used. If template switching occurred, the resulting fragment would have the same inner primer site located on both sides of the insert and would amplify with a single primer (Figure 1). Six different templates were used in the initial PCR reaction, of which one could theoretically promote template switching. Two cloned *185/333* genes and an actin gene were used in various combinations. As a negative control, each of the three clones was used as a single template. Because there was no sequence homology between actin and *185/333* clones, the reactions in which the actin clone was mixed with either of the *185/333* clones also served as putative negative controls. Therefore, the only experimental conditions in which template switching might have occurred were in the template mixtures of the two homologous *185/333* clones.

Preliminary experiments indicated that residual M13 primers from the initial PCR contaminated the second round of amplification and resulted in high levels of ^{32}P incorporation (data not shown). Therefore, in addition to cleaning the initial PCR reactions to eliminate excess reagents, three precautionary steps were taken to ensure that amplification in the second round of

PCR was not a result of residual M13 primers. First, the M13 primer concentration was titrated to the lowest concentration that did not inhibit amplification. Second, because the optimal annealing temperature of the M13 primers was 58°C, the T3 and T7 primers were lengthened (T3L and T7L) to increase their optimal annealing temperature to 68°C, a temperature at which M13 amplification was significantly reduced. Finally, to identify background amplification from residual M13 primers, control reactions that omitted the T3L and T7L primers were run for each template used in the second round of PCR (Supplemental Figure 2, “No primer” samples). Positive controls employed with both T3L and T7L primers (“double primer” samples; Supplemental Figure 2).

For all six templates, the ^{32}P-dCTP incorporation into the products amplified when both T3L and T7L were included in the reaction was significantly higher than in reactions with single primers or when both primers were omitted (Supplemental Figure 2). Specifically, the average counts per minute (cpm) for the positive controls was ~1000 X higher than the single primer reactions, and ~13,000 X higher than the reactions without primers. When unlabeled reactions were analyzed by electrophoresis, bands of the correct size were observed when both primers were included, whereas no bands were observed in reactions with single primers or with no primers (data not shown).

Samples without primers were included to detect residual M13 amplification, and provided background levels of residual unincorporated ^{32}P-dCTP. Therefore, to determine whether template switching occurred, the reactions using single primers were compared to those lacking primers. Results showed that there was no significant difference among any of the six templates between the ^{32}P-dCTP incorporation in the reactions amplified with single primers vs. the reactions without primers (Supplemental Figure 2). Furthermore, there was no significant

difference in single primer amplification between single vs. mixed clone templates. These results are strong evidence that template switching did not occur frequently enough during amplification of genes prior to cloning to generate recombinant amplicons that would artificially elevate the sequence diversity of the *185/333* genes.

Discussion

Artificially introduced SNPs

Based on the error rates of the two *Taq* DNA polymerases characterized in this study, it is expected that there is one artificially introduced error in 2 of the 34 clones from animal 4, and, at most, one error in 7 of the 137 clones from animals 2 and 10. These numbers are likely overestimates, though, due to the fact that no errors were found using *Optimase.* However, of the 34 genes from animal 4, 30 had unique nucleotide sequences and only one pair of sequences differed by a single nucleotide. Furthermore, from the 91 unique genes cloned from animals 2 and 10, 12 pairs of genes differed by a single nucleotide. It is not known whether these SNPs represent real or *Taq*-induced polymorphisms, however, because the diversity scores rely on the frequency of the states at each nucleotide position, variations that occur in a single sequence do not greatly impact the element diversity scores. Furthermore, given that 22% of the nucleotide positions within the second exon (for either alignment) are variable, and that the majority have more than two nucleotides that are different, this extremely low number of putative *Taq*-induced errors does not impact on the variations in diversity scores observed among this set of *185/333* gene sequences.

Artificial gene recombination

Additional File 5: Diversity Control Experiments

Using conditions that closely mimic those originally employed in the amplification of *185/333* genes from gDNA, the results demonstrated that template switching does not occur frequently enough to increase sequence diversity artificially. Furthermore, the reactions in which two homologous *185/333* genes were mixed and amplified with single primers, the ^{32}P-dCTP incorporation was approximately 1100 times less than the reactions in which both primers were present, These results indicate that if recombinant sequences are present, their concentration is too low to detected with the methods employed here. Because less than 100 clones were obtained from each animal, it is highly unlikely that any of the observed diversity is the result of artificial template switching. Although the *185/333* genes are an extraordinarily diverse family, artificially elevated diversity resulting from either increased numbers of SNPs due to *Taq*-induced misincorporation or artificial recombinant templates from template switching can be ruled out.

1. Cline J, Braman JC, Hogrefe HH: **PCR fidelity of pfu DNA polymerase and other thermostable DNA polymerases**. *Nucleic Acids Res* 1996, **24**(18):3546-3551.
2. Odelberg SJ, Weiss RB, Hata A, White R: **Template-switching during DNA synthesis by Thermus aquaticus DNA polymerase I**. *Nucleic Acids Res* 1995, **23**(11):2049-2057.
3. Thompson JR, Marcelino LA, Polz MF: **Heteroduplexes in mixed-template amplifications: formation, consequence and elimination by 'reconditioning PCR'**. *Nucleic Acids Res* 2002, **30**(9):2083-2088.
4. Zhang SM, Adema CM, Kepler TB, Loker ES: **Diversification of Ig superfamily genes in an invertebrate**. *Science* 2004, **305**(5681):251-254.
5. Terwilliger DP, Buckley KM, Mehta D, Moorjani PG, Smith LC: **Unexpected diversity displayed in cDNAs expressed by the immune cells of the purple sea urchin, *Strongylocentrotus purpuratus***. *Physiological Genomics* 2006, **26**(2):134-144.
6. Lee JJ, Shott RJ, Rose SJ, 3rd, Thomas TL, Britten RJ, Davidson EH: **Sea urchin actin gene subtypes. Gene number, linkage and evolution**. *J Mol Biol* 1984, **172**(2):149-176.
7. Coffaro KA, Hinegardner RT: **Immune response in the sea urchin Lytechinus pictus**. *Science* 1977, **197**(4311):1389-1390.
8. Terwilliger DP, Buckley KM, Brockton V, Ritter NJ, Smith LC: **Distinctive expression patterns of 185/333 genes in the purple sea urchin, Strongylocentrotus purpuratus: an unexpectedly diverse family of transcripts in response to LPS, beta-1,3-glucan, and dsRNA**. *BMC Mol Biol* 2007, **8**:16.

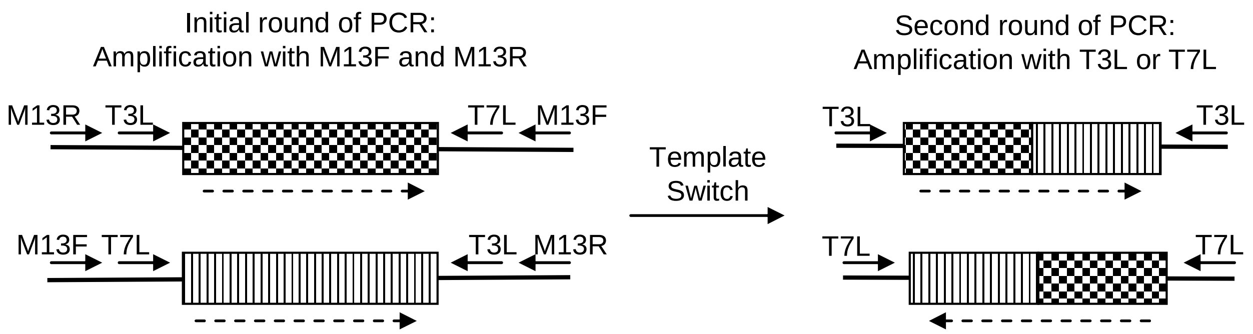

Supplemental Figure 1: Experimental Design for Template Switching. Two genes (checkerboard pattern vs. striped pattern), cloned in opposite orientations into pCR4-TOPO vector are shown. The arrow under each gene indicates the 5' to 3' orientation, and the primer sites in the polylinkers are shown. If template switching occurs, PCR with either T3L primer or T7L primer alone will amplify a heterogeneous product. Templates that do not switch will not amplify with single primers.

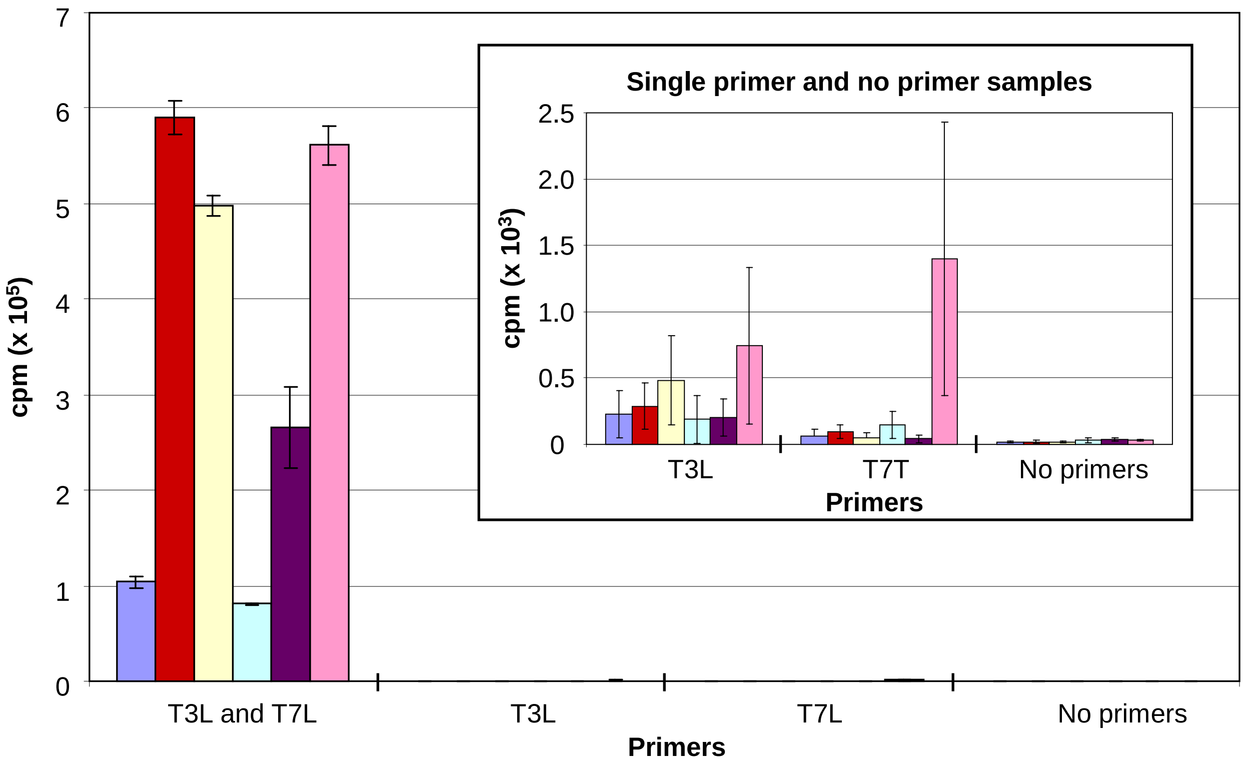

Figure 2: Background-subtracted average counts per minute (cpm) for PCR products amplified using the specified primers. Error bars indicate the standard deviation among three replicates. Each color bar represents one of the six templates used in the initial (M13F/M13R) amplification: blue = 2-013; red = 2-103; yellow = 2-013 and 2-103; light green = actin; purple = actin and 2-013; pink = actin and 2-103. In all six cases, the counts per minute for the double primer samples were statistically different from their respective single or no primer samples ($p < 0.05$). In contrast, none of the single primer samples were statistically different from the respective no primer reactions ($p > 0.05$), indicating that recombinant template amplification did not occur.
