# Supplementary material for: Extraordinary diversity among members of the large gene family, 185/333, from the purple sea urchin, Strongylocentrotus purpuratus
Source: BMC Mol Biol. 2007 Aug 15;8:68. doi: 10.1186/1471-2199-8-68 (PMC1988830; doi:10.1186/1471-2199-8-68)
Supplement: Additional file 6 — Repeat-based nucleotide alignment of the 185/333 genes. The repeat-based alignment of the second exon of the 121 unique gene sequences showing the element borders and repeats. [file 1471-2199-8-68-S6.pdf]

Additional File 6: Repeat-based alignment of the unique gene sequences. Only the second exon is shown, because the leader and intron alignments are identical to those shown in Additional Files 2 and 3. Element borders are indicated by vertical black lines, and are labeled above the sequence. Repeats are indicated by shaded boxes, which each different color (red = type 1; blue = type 2; green = type 3; yellow = type 4; and purple = type 5).

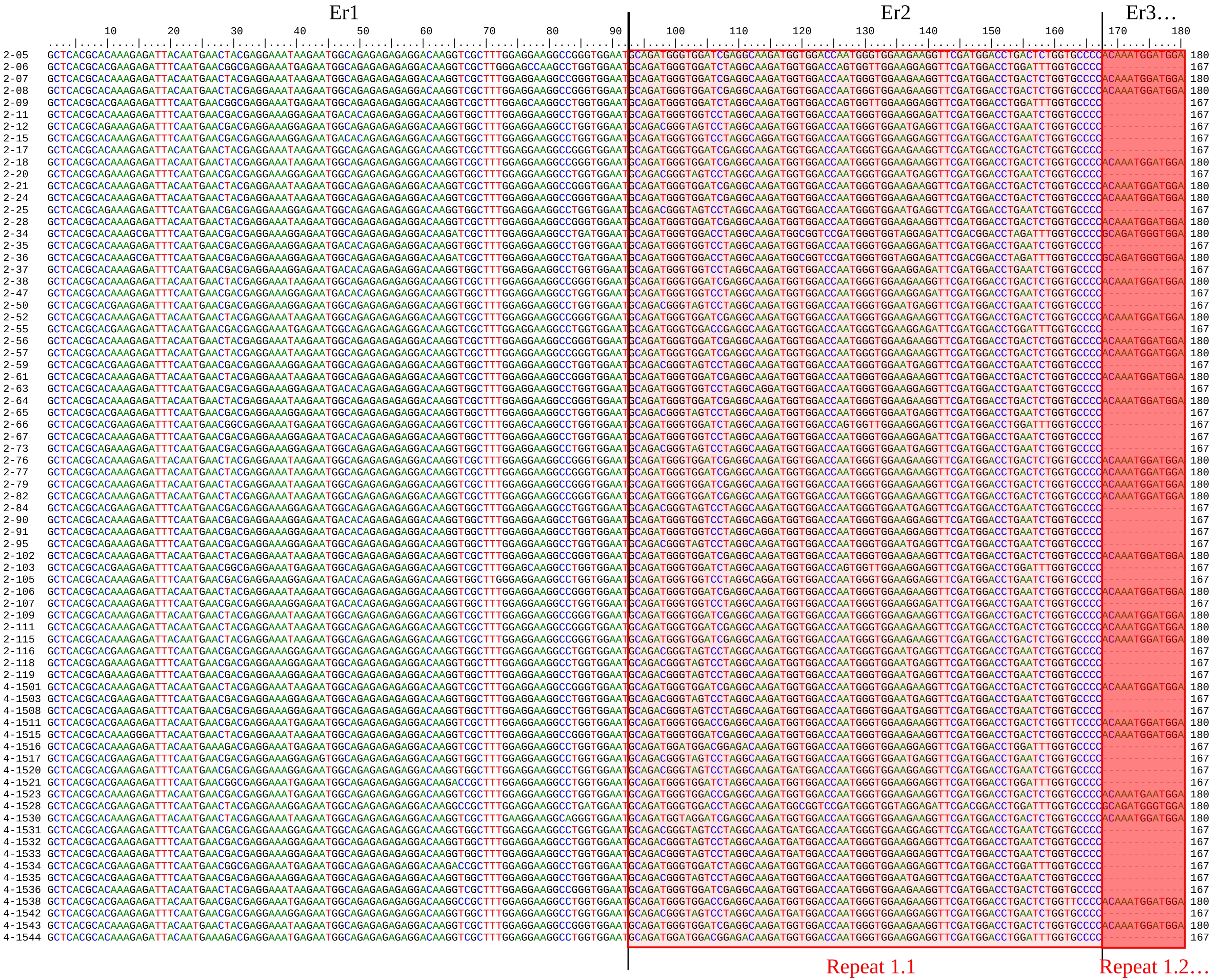
Er1
Er2
Er3…
Repeat 1.1
Repeat 1.2…

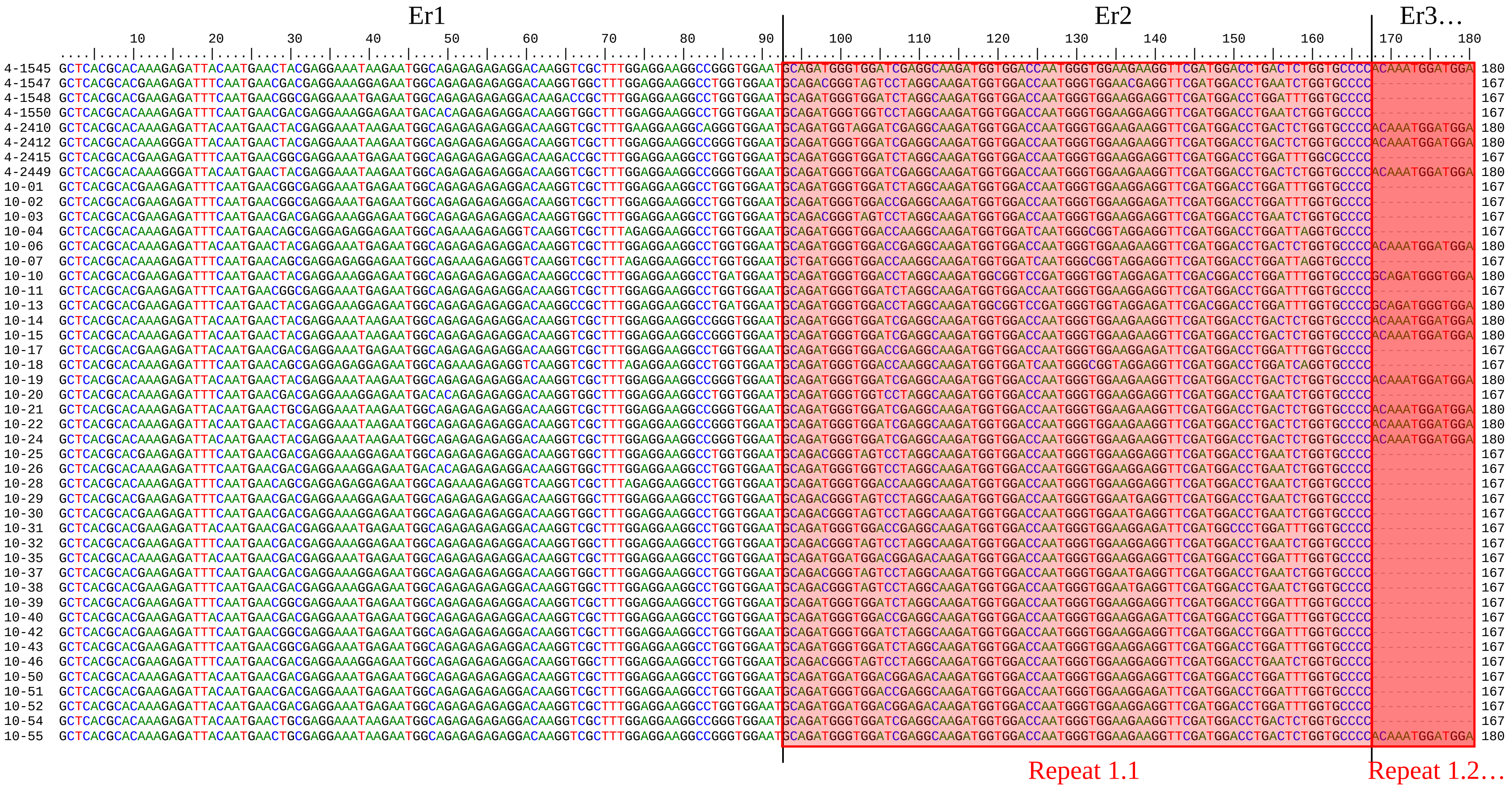
Er1
Er2
Er3…
Repeat 1.1
Repeat 1.2…

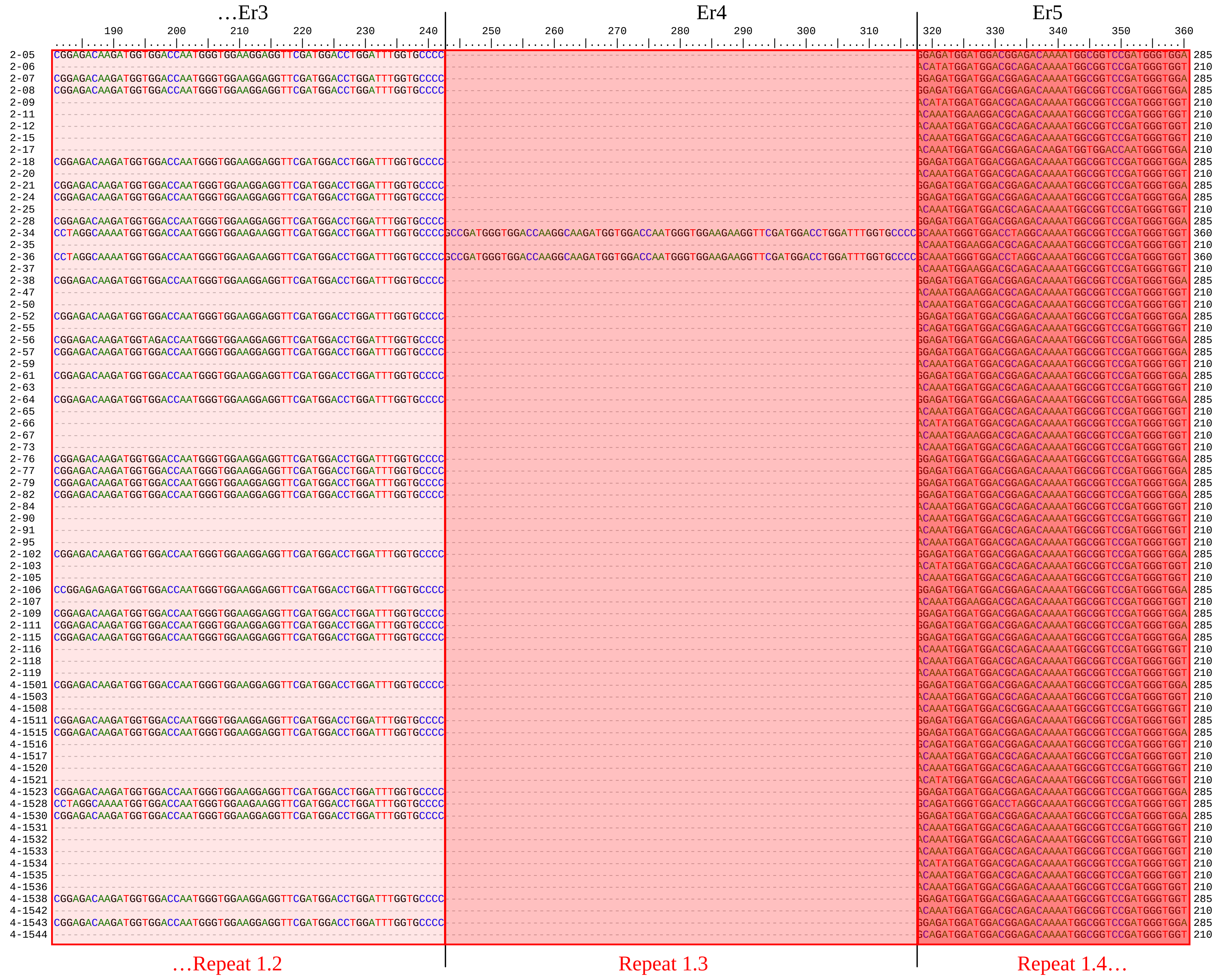
…Er3
Er4
Er5
…Repeat 1.2
Repeat 1.3
Repeat 1.4…

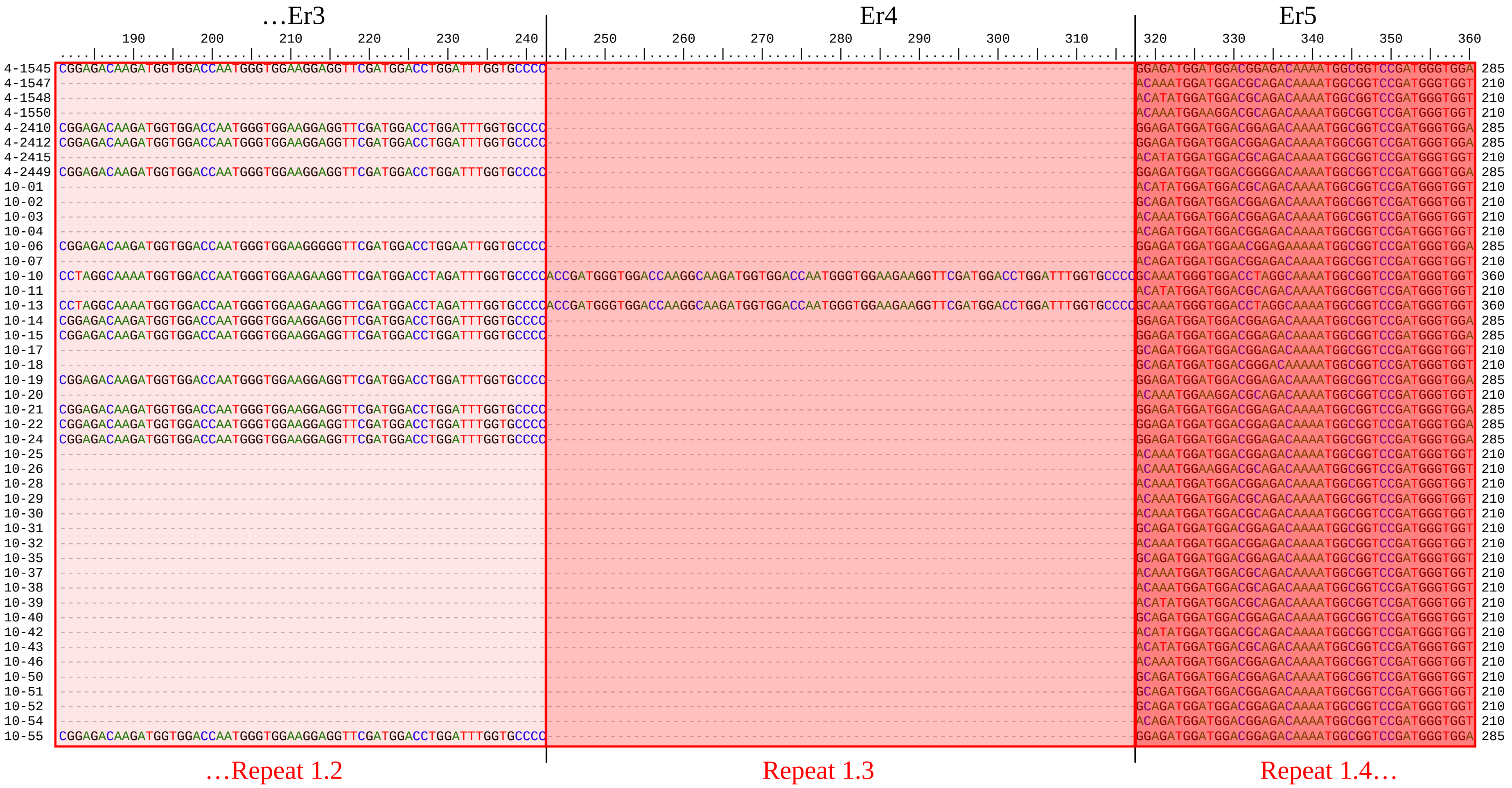
…Er3
Er4
Er5
…Repeat 1.2
Repeat 1.3
Repeat 1.4…

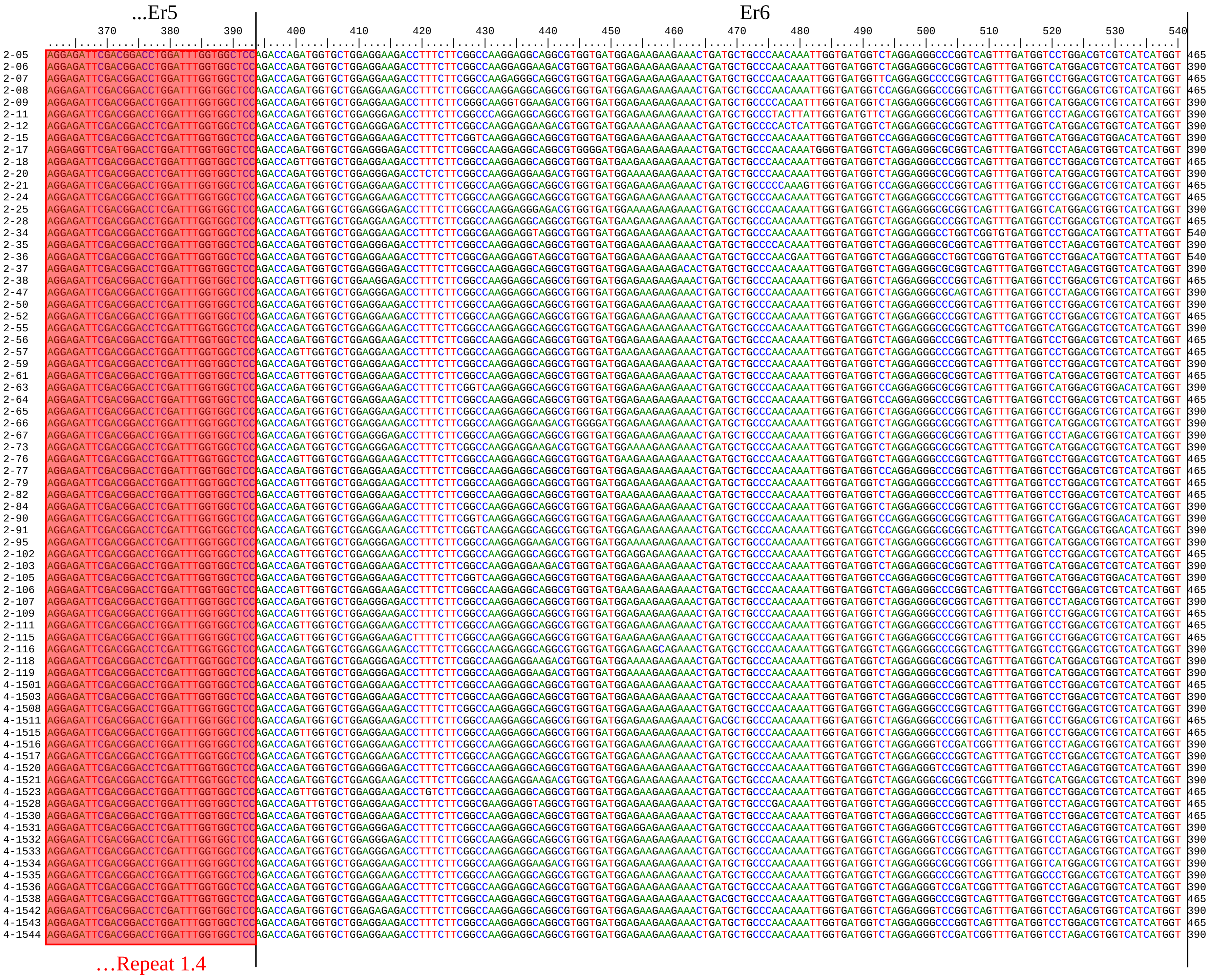

...Er5
Er6
…Repeat 1.4

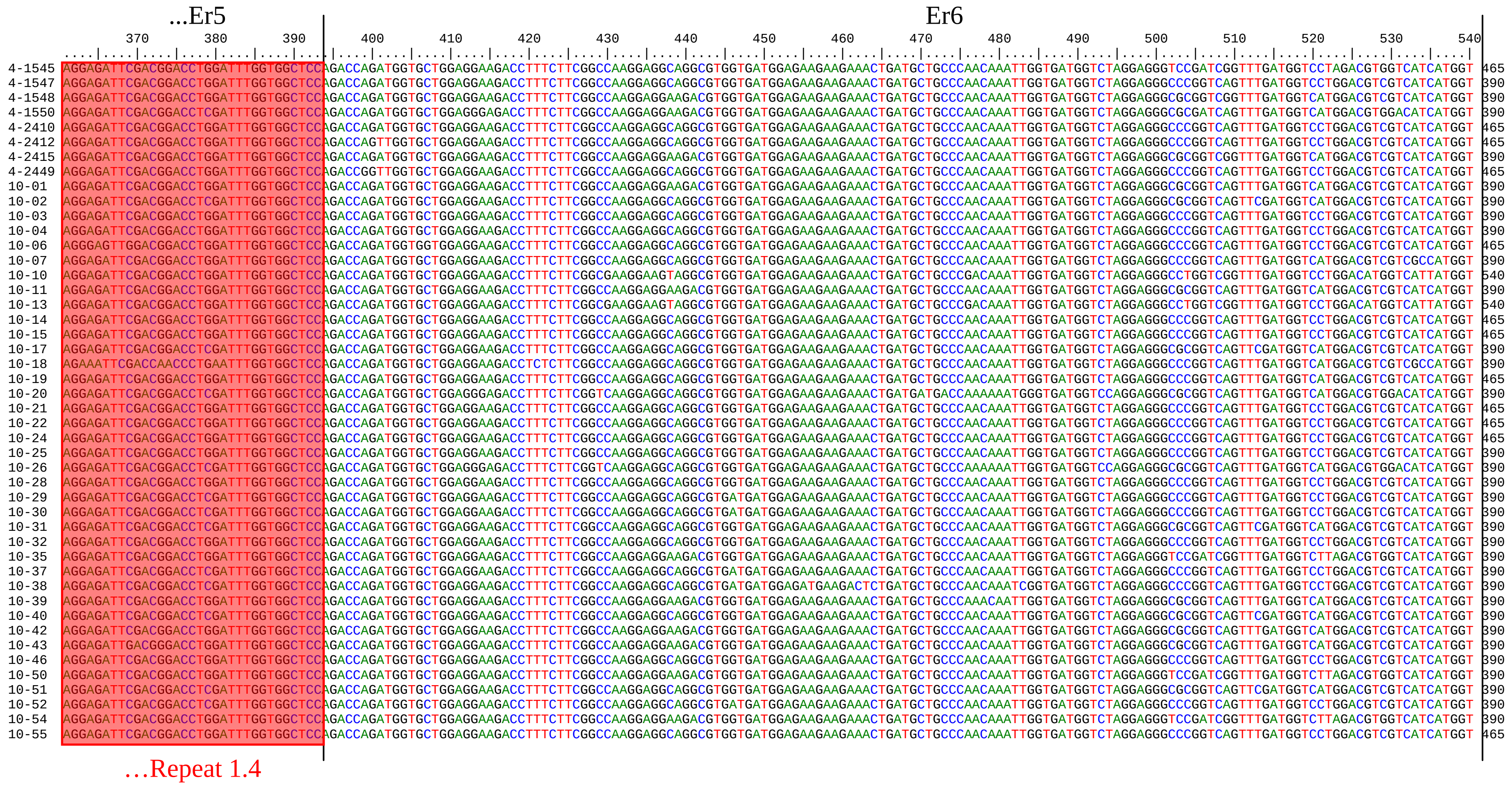
...Er5
Er6
...Repeat 1.4
4-1545
4-1547
4-1548
4-1550
4-2410
4-2412
4-2415
4-2449
10-01
10-02
10-03
10-04
10-06
10-07
10-10
10-11
10-13
10-14
10-15
10-17
10-18
10-19
10-20
10-21
10-22
10-24
10-25
10-26
10-28
10-29
10-30
10-31
10-32
10-35
10-37
10-38
10-39
10-40
10-42
10-43
10-46
10-50
10-51
10-52
10-54
10-55

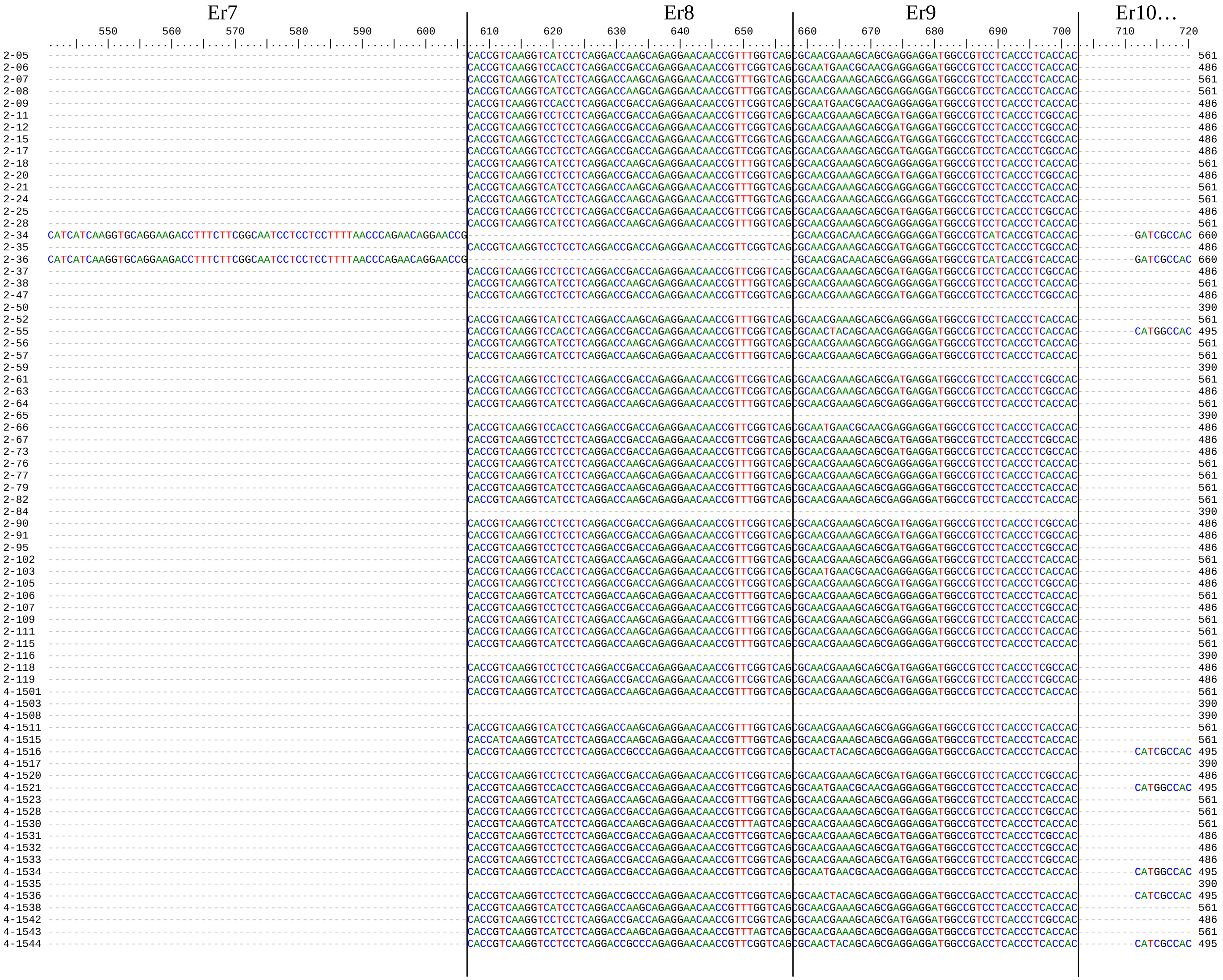
Er7
Er8
Er9
Er10…

	Er7	Er8	Er9	Er10…	
4-1545		CACCGTCAAGGTCCTCCTCAGGACCGCCCAGAGGAACAACCGTTCGGTCAG	CGCAACTACAGCAGCGAGGAGGATGGCCGACCTCACCCTCACCAC	CATCGCCAC	570
4-1547					390
4-1548		CACCGTCAAGGTCCACCTCAGGACCGACCAGAGGAACAACCGTTCGGTCAG	CGCAATGAACGCAACGAGGAGGATGGCCGTCCTCACCCTCACCAC	CATGGCCAC	495
4-1550		CACCGTCAAGGTCCTCCTCAGGACCGACCAGAGGAACAACCGTTCGGTCAG	CGCAACGAAAGCAGCGATGAGGATGGCCGTCCTCACCCTCGCCAC		486
4-2410		CACCGTCAAGGTCATCCTCAGGACCAAGCAGAGGAACAACCGTTTAGTCAG	CGCAACGAAAGCAGCGAGGAGGATGGCCGTCCTCACCCTCACCAC		561
4-2412		CACCGTCAAGGTCATCCTCAGGACCAAGCAGAGGAACAACCGTTTGGTCAG	CGCAACGAAAGCAGCGAGGAGGATGGCCGTCCTCACCCTCACCAC		561
4-2415		CACCGTCAAGGTCCACCTCAGGACCGACCAGAGGAACAACCGTTCGGTCAG	CGCAATGAACGCAACGAGGAGGATGGCCGTCCTCACCCTCACCAC	CATGGCCAC	495
4-2449		CACCGTCAAGGTCATCCTCAGGACCAAGCAGAGGAACAACCGTTTGGTCAG	CGCAACGAAAGCAGCGAGGAGGATGGCCGTCCTCACCCTCACCAC		561
10-01		CACCGTCAAGGTCCACCTCAGGACCGACCAGAGGAACAACCGTTCGGTCAG	CGCAATGAACGCAACGAGGAGGATAGCCGTCCTCACCCTCACCAC	CATCGCCAC	495
10-02		CACCGTCAAGGTCCACCTCAGGACCGACCAGAGGAACAACCGTTCGGTCAG	CGCAACTACAGCAACGAGGAGGATGGCCGTCCTCACCCTCACCAC	CATGGCCAC	495
10-03					390
10-04		CACCGTCGAGGTCATCCTCAGGACCAAGCAGAGGAACAACCGTTTGGTCAG	CGCAACGAAAGCAGCGAGGAGGATGGCCGTCCTCACCCTCACCAC		486
10-06		CACCGTCGAGGTCATCCTCAGGACCAAGCAGAGGAACAACCGTTTGGTCAG	CGCAACGAAAGCAGCGAGGAGGATGGCCGTCCTCACCCTCACCAC		561
10-07		CACCGTCAAGGACCACCTCAGGATCGATCAGAGGAACAACCGTTCGGTCAG	CGCAATGAAAGCAGCGAGGAGGATGGCCGTCCTCACCCTCACCAC	CATCGCCAC	495
10-10	CATCATCAAGGTGCAGGAAGACCTTTCTTCGGCAATCCTCCTCCTTTTAACCCAGAACAGGAACCG		CGCAACGACAGCAGCGAGGAGGATGGCCGTCATCACCGTCACCAC	GATCGCCAC	660
10-11		CACCGTCAAGGTCCACCTCAGGACCGACCAGAGGAACAACCGTTCGGTCAG	CGCAATGAACGCAACGAGGAGGATAGCCGTCCTCACCCTCACCAC	CATCGCCAC	495
10-13	CATCATCAAGGTGCAGGAAGACCTTTCTTCGGCAATCCTCCTCCTTTTAACCCAGAACAGGAACCG		CGCAACGACAGCAGCGAGGAGGATGGCCGTCATCACCGTCACCAC	GATCGCCAC	660
10-14		CACCGTCAAGGTCATCCTCAGGACCAAGCAGAGGAACAACCGTTTGGTCAG	CGCAACGAAAGCAGCGAGGAGGATGGCCGTCCTCACCCTCACCAC		561
10-15		CACCGTCAAGGTCATCCTCAGGACCAAGCAGAGGAACAACCGTTTGGTCAG	CGCAACGAAAGCAGCGAGGAGGATGGCCGTCCTCACCCTCACCAC		561
10-17		CACCGTCAAGGTCCACCTCAGGACCGACCAGAGGAACAACCGTTCGGTCAG	CGCAACTACAGCAACGAGGAGGATGGCCGTCCTCACCCTCACCAC	CATGGCCAC	495
10-18		CACCGTCAAGGACCACCTCAGGATCGATCAGAGGAACAACCGTTCGGTCAG	CGCAATGAAAGCAGCGAGGAGGATGGCCGTCCTCACCCTCACCAC	CATCGCCAC	495
10-19		CACCGTCAAGGTCCACCTCAGGACCGACCAGAGGAACAACCGTTCGGTCAG	CGCAACTACAGCAACGAGGAGGATGGCCGTCCTCACCCTCACCAC	CATGGCCAC	570
10-20		CACCGTCAAGGTCCTCCTCAGGACCGACCAGAGGAACAACCGTTCGGTCAG	CGCAACGAAAGCAGCGATGAGGATGGCCGTCCTCACCCTCGCCAC		486
10-21		CACCGTCAAGGTCATCCTCAGGACCAAGCAGAGGAACAACCGTTTGGTCAG	CGCAACGAAAGCAGCGAGGAGGATGGCCGTCCTCACCCTCACCAC		561
10-22		CACCGTCAAGGTCATCCTCAGGACCAAGCAGAGGAACAACCGTTTGGTCAG	CGCAACGAAAGCAGCGAGGAGGATGGCCGTCCTCACCCTCACCAC		561
10-24		CACCGTCAAGGTCATCCTCAGGACCAAGCAGAGGAACAACCGTTTGGTCAG	CGCAACGAAAGCAGCGAGGAGGATGGCCGTCCTCACCCTCACCAC		561
10-25					390
10-26		CACCGTCAAGGTCCTCCTCAGGACCGACCAGAGGAACAACCGTTCGGTCAG	CGCAACGAAAGCAGCGATGAGGATGGCCGTCCTCACCCTCGCCAC		486
10-28					390
10-29					390
10-30					390
10-31		CACCGTCAAGGTCCACCTCAGGACCGACCAGAGGAACAACCGTTCGGTCAG	CGCAACTACAGCAACGAGGAGGATGGCCGTCCTCACCCTCACCAC	CATGGCCAC	495
10-32					390
10-35		CACCGTCAAGGTCCTCCTCAGGACCGCCCAGAGGAACAACCGTTCGGTCAG	CGCAACTACAGCAGCGAGGAGGATGGCCGACCTCACCCTCACCAC	CATCGCCAC	495
10-37					390
10-38					390
10-39		CACCGTCAAGGTCCACCTCAGGACCGACCAGAGGAACAACCGTTCGGTCAG	CGCAATGAACGCAACGAGGAGGATAGCCGTCCTCACCCTCACCAC	CATCGCCAC	495
10-40		CACCGTCAAGGTCCACCTCAGGACCGACCAGAGGAACAACCGTTCGGTCAG	CGCAACTACAGCAACGAGGAGGATGGCCGTCCTCACCCTCACCAC	CATGGCCAC	495
10-42		CACCGTCAAGGTCCACCTCAGGACCGACCAGAGGAACAACCGTTCGGTCAG	CGCAATGAACGCAACGAGGAGGATAGCCGTCCTCACCCTCACCAC	CATCGCCAC	495
10-43		CACCGTCAAGGTCCACCTCAGGACCGACCAGAGGAACAACCGTTCGGTCAG	CGCAATGAACGCAACGAGGAGGATAGCCGTCCTCACCCTCACCAC	CATCGCCAC	495
10-46					390
10-50		CACCGTCAAGGTCCTCCTCAGGACCGCCCAGAGGAACAACCGTTCGGTCAG	CGCAACTACAGCAGCGAGGAGGATGGCCGACCTCACCCTCACCAC	CATCGCCAC	495
10-51		CACCGTCAAGGTCCACCTCAGGACCGACCAGAGGAACAACCGTTCGGTCAG	CGCAACTACAGCAACGAGGAGGATGGCCGTCCTCACCCTCACCAC	CATGGCCAC	495
10-52					390
10-54		CACCGTCAAGGTCCTCCTCAGGACCGCCCAGAGGAACAACCGTTCGGTCAG	CGCAATGAAAGCAGCGAGGAGGATGGCCGTCCTCACCCTCACCAC	CATCGCCAC	495
10-55		CACCGTCAAGGTCATCCTCAGGACCAAGCAGAGGAACAACCGTTTGGTCAG	CGCAACGAAAGCAGCGAGGAGGATGGCCGTCCTCACCCTCACCAC		561

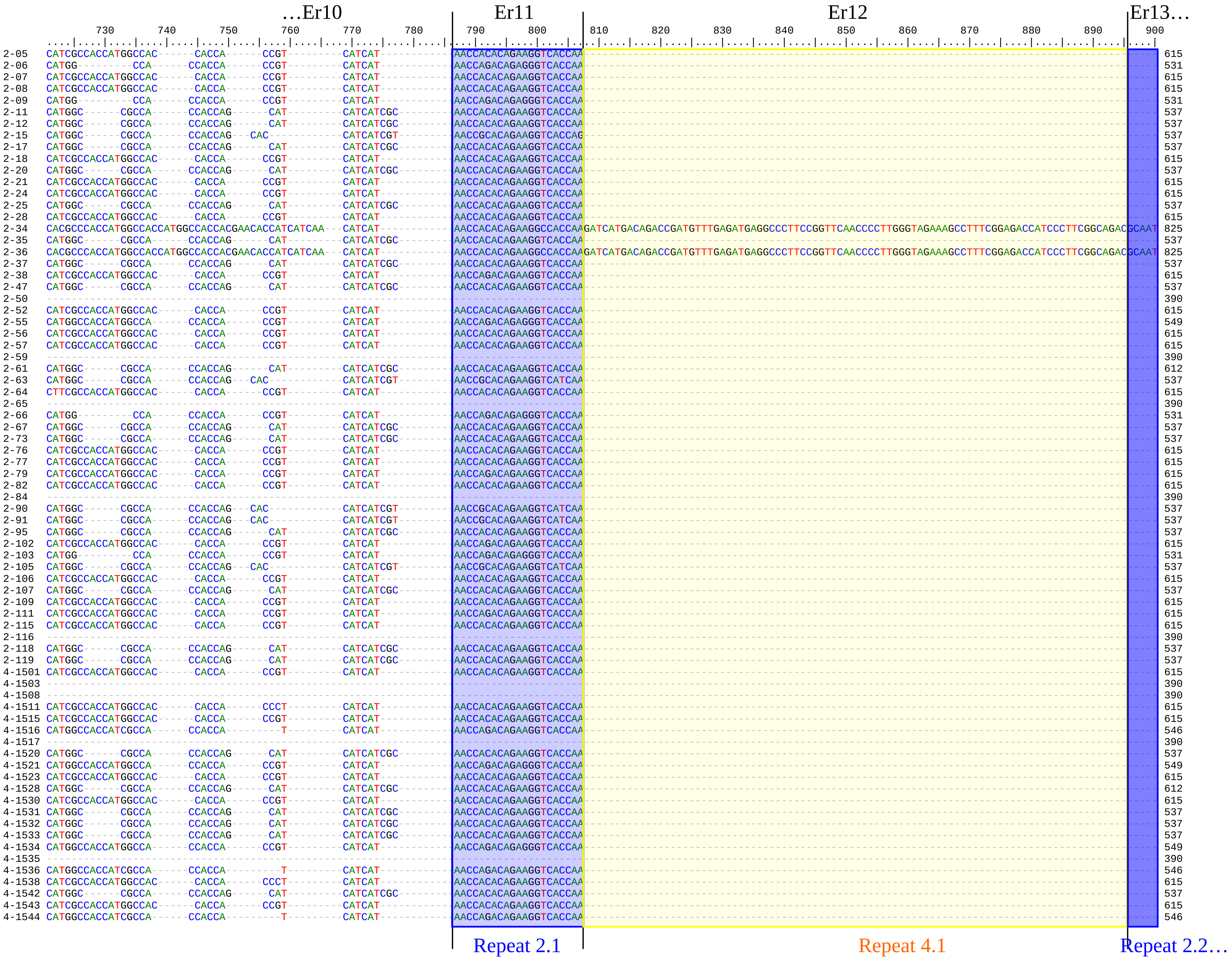

…Er10
Er11
Er12
Er13…
Repeat 2.1
Repeat 4.1
Repeat 2.2…

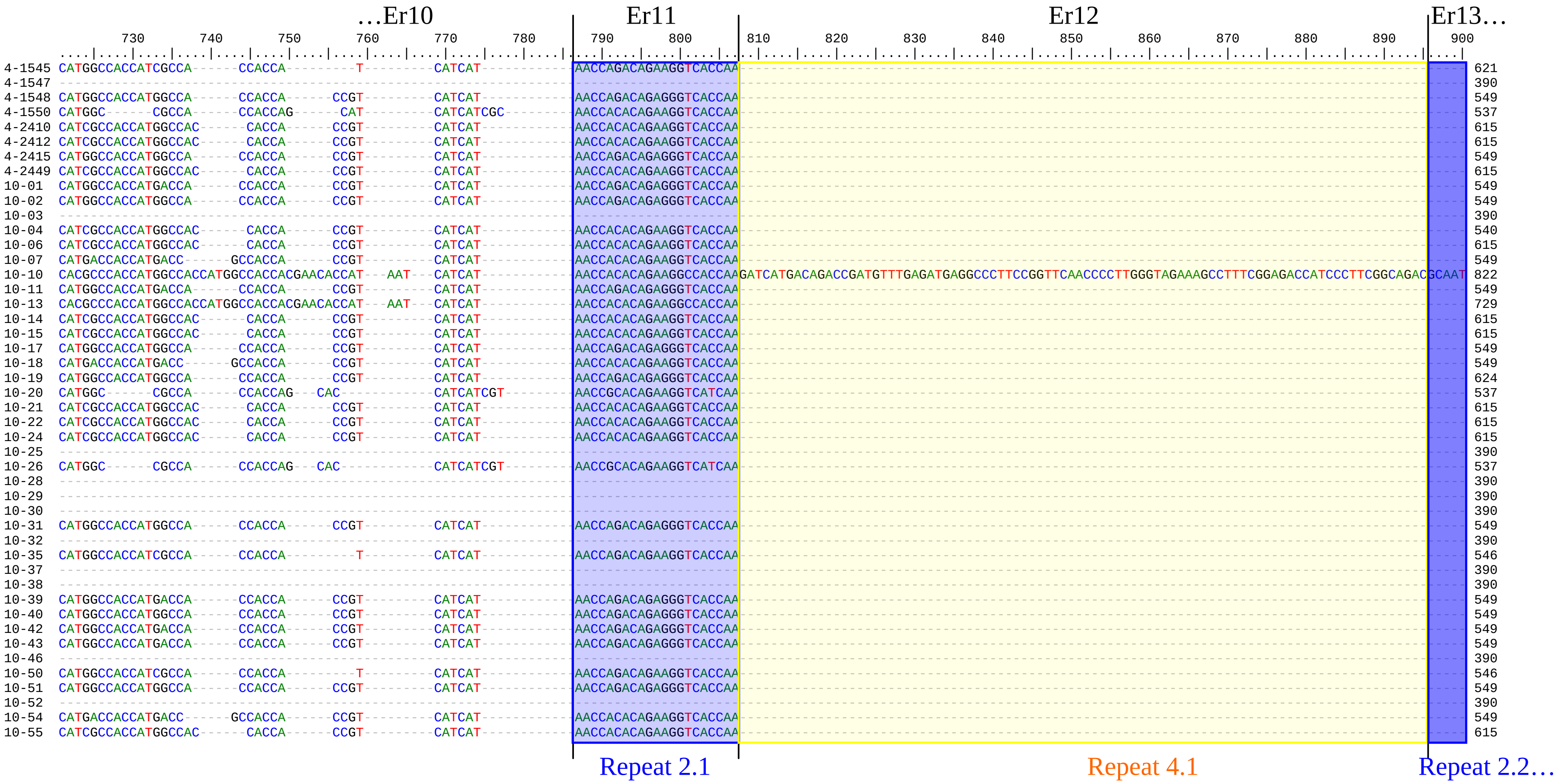
…Er10
Er11
Er12
Er13…
Repeat 2.1
Repeat 4.1
Repeat 2.2…

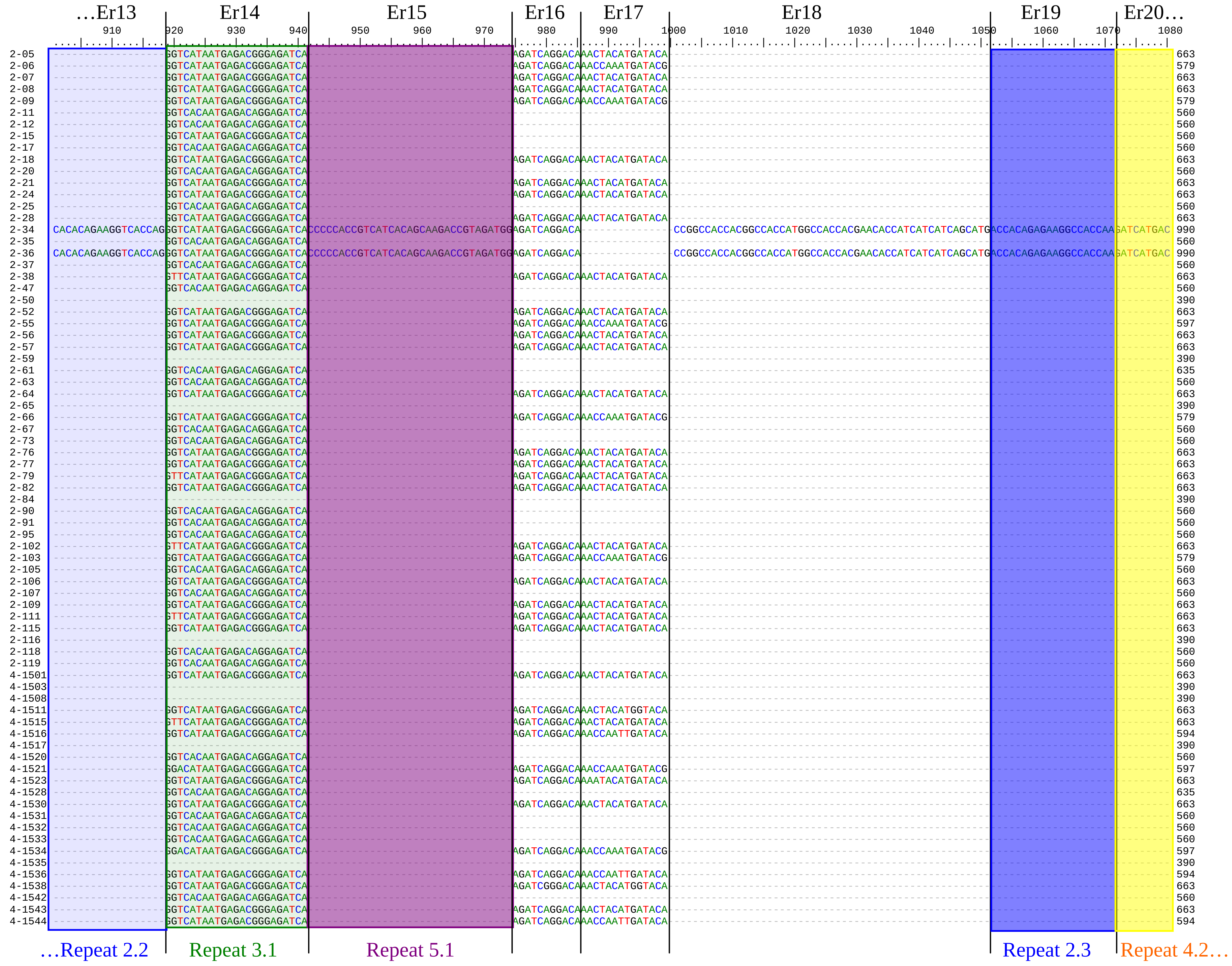
…Er13
Er14
Er15
Er16
Er17
Er18
Er19
Er20…
…Repeat 2.2
Repeat 3.1
Repeat 5.1
Repeat 2.3
Repeat 4.2…

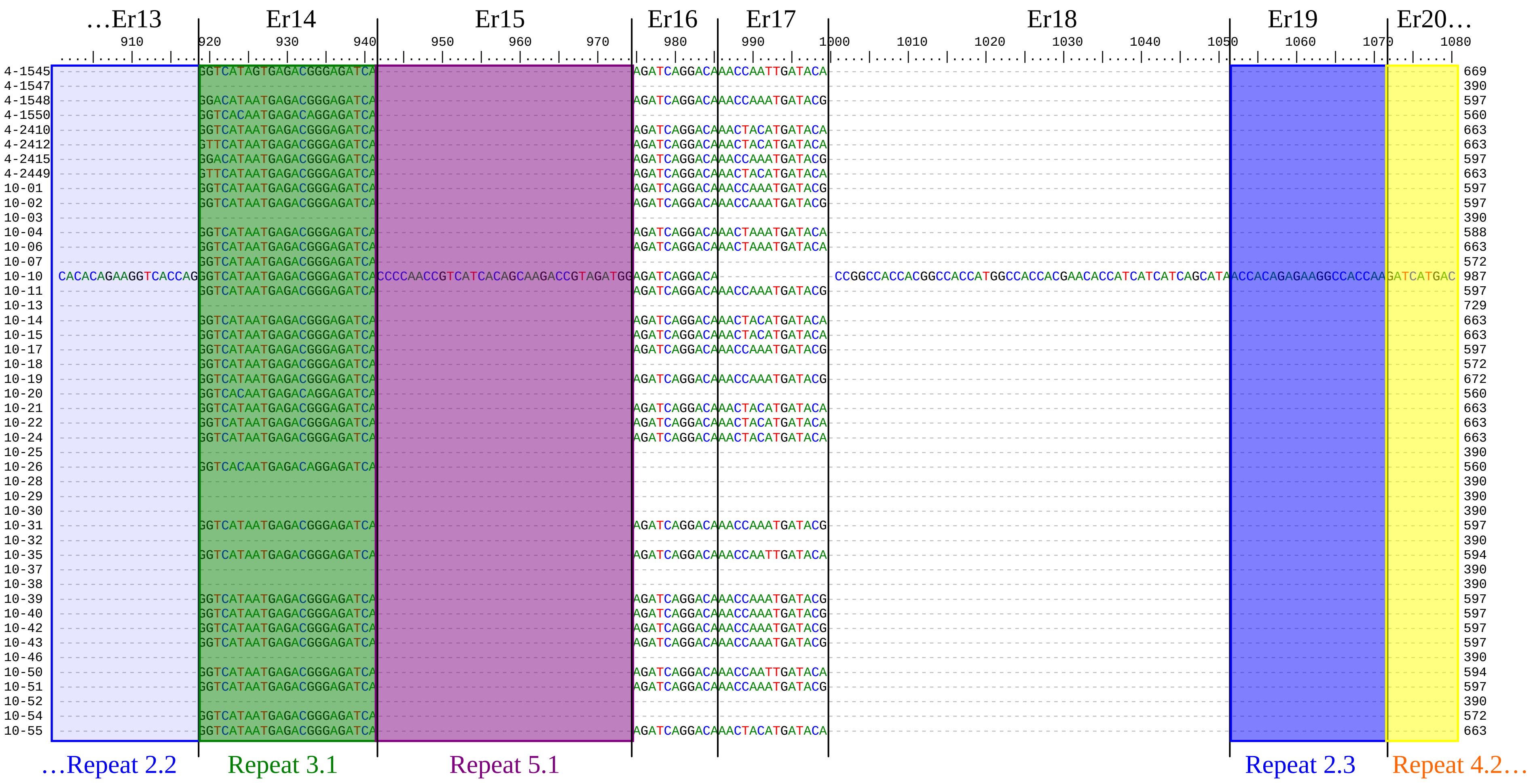
…Er13
Er14
Er15
Er16
Er17
Er18
Er19
Er20…
…Repeat 2.2
Repeat 3.1
Repeat 5.1
Repeat 2.3
Repeat 4.2…

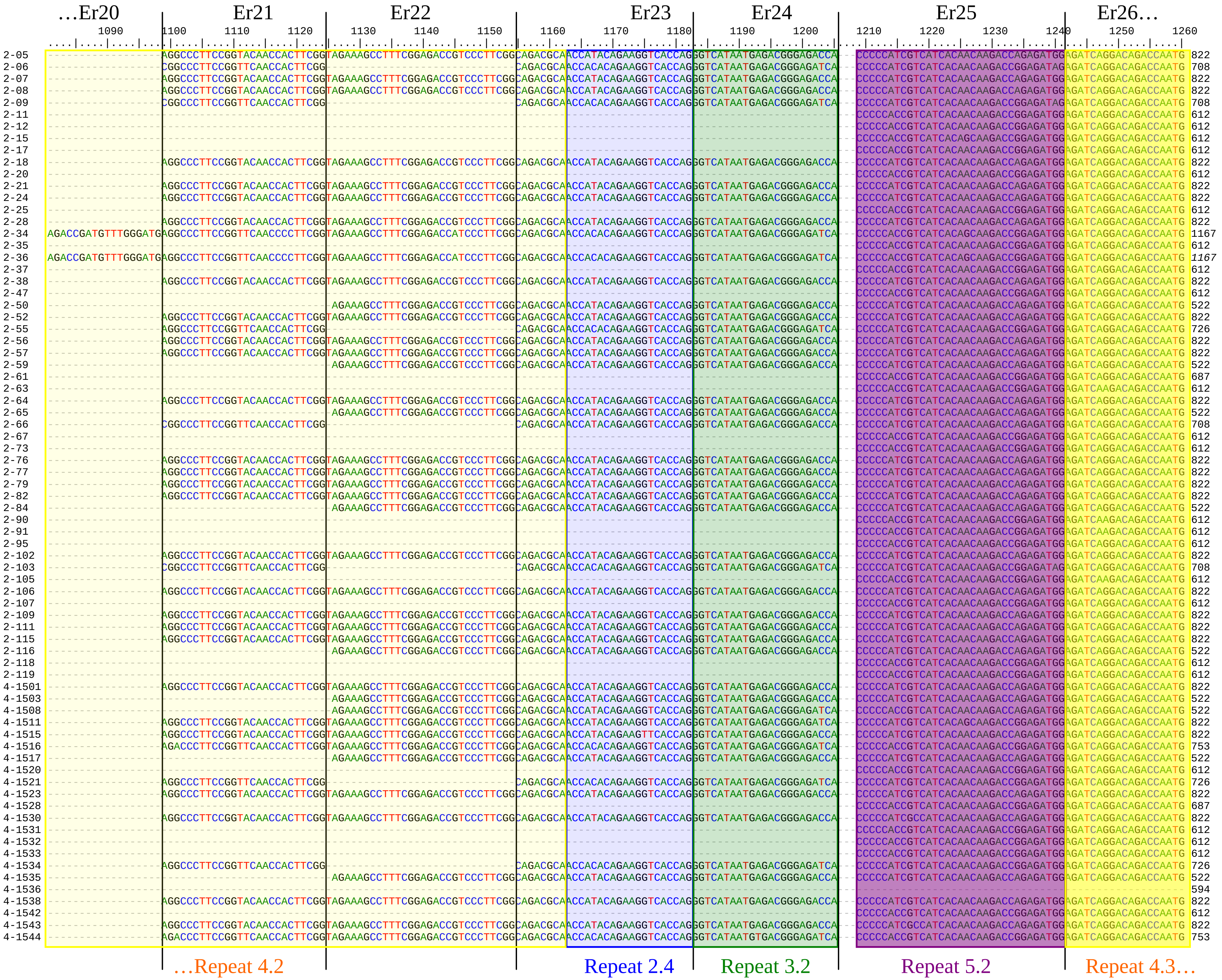
…Er20
Er21
Er22
Er23
Er24
Er25
Er26…
…Repeat 4.2
Repeat 2.4
Repeat 3.2
Repeat 5.2
Repeat 4.3…

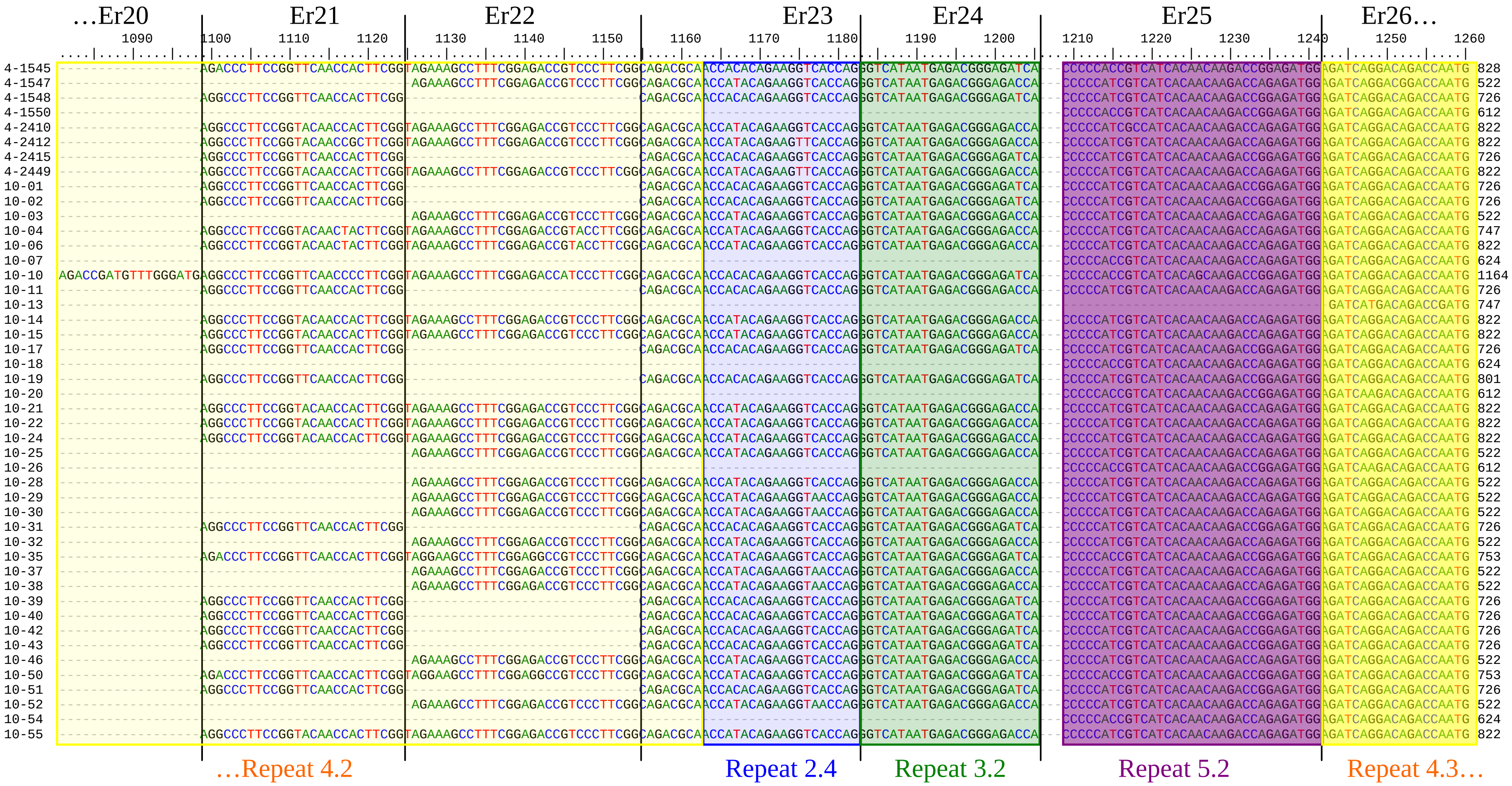

…Er20
Er21
Er22
Er23
Er24
Er25
Er26…
…Repeat 4.2
Repeat 2.4
Repeat 3.2
Repeat 5.2
Repeat 4.3…

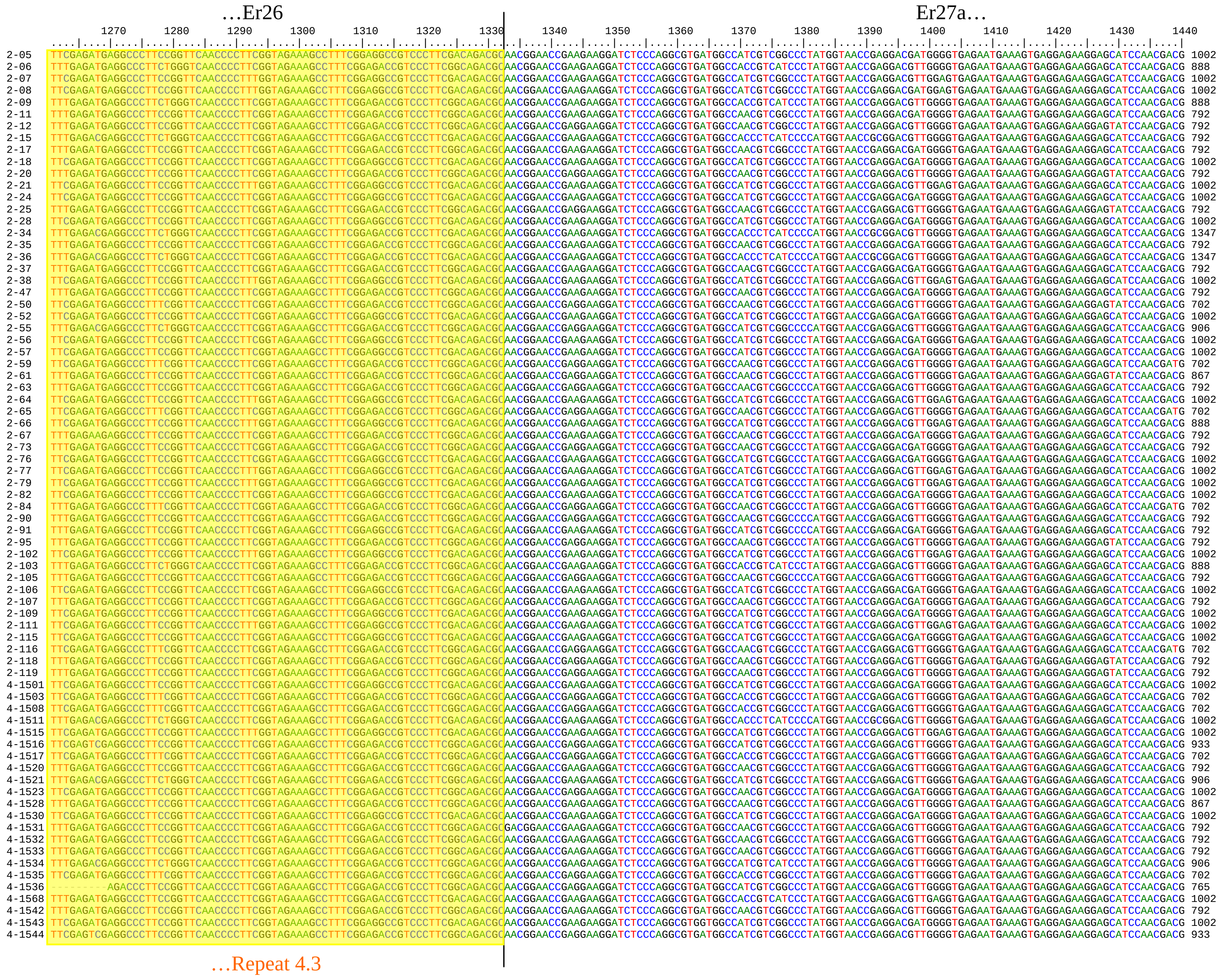
…Er26
Er27a…
…Repeat 4.3

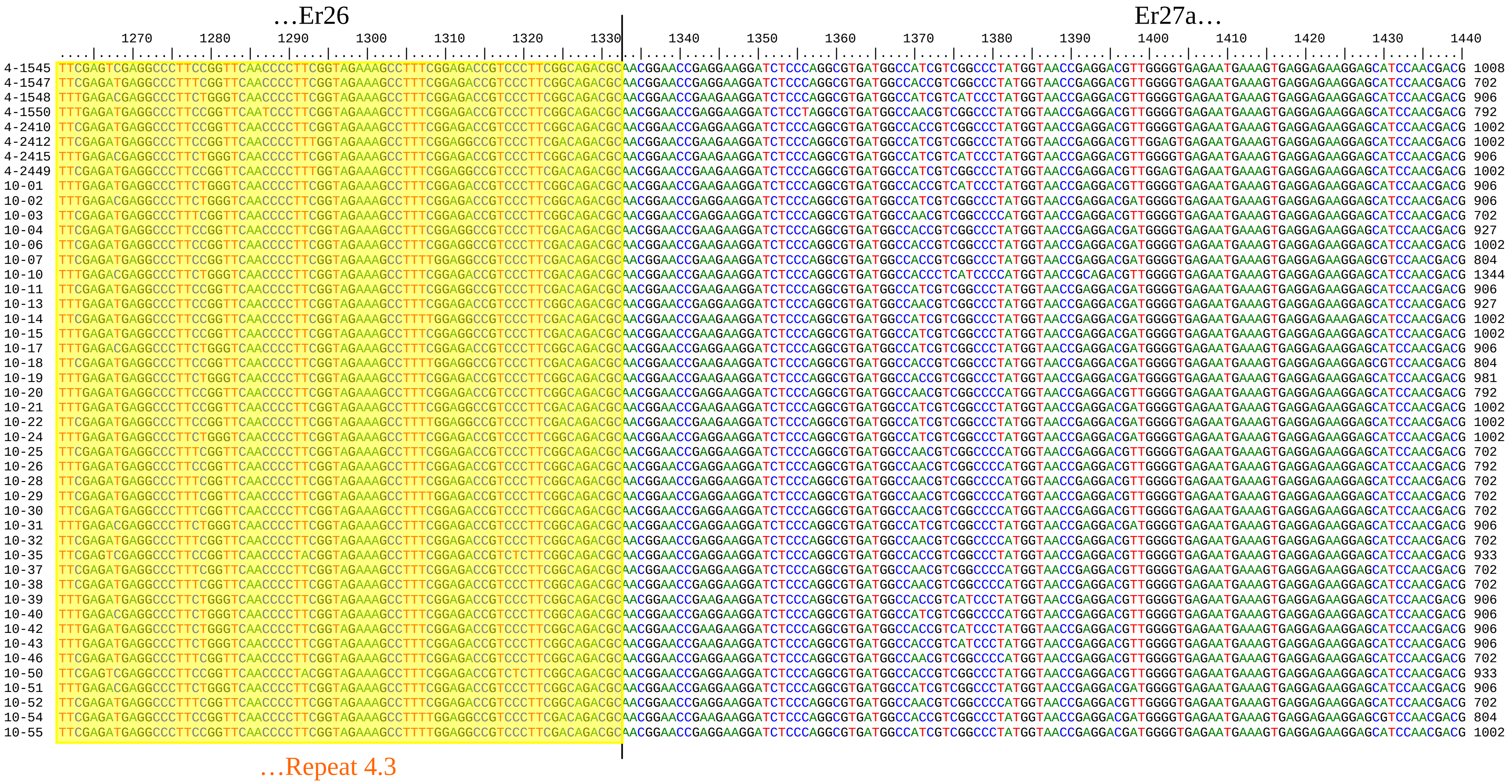
…Er26
Er27a…
…Repeat 4.3

…Er27a | Er27b

```
                1450      1460      1470      1480      1490      1500      1510      1520
        ....|....|....|....|....|....|....|....|....|....|....|....|....|....|....|....|....
2-05    GAAAGCGTAACGACATCTTCACCACCTGAAGTGGTC------GCAATCAATGAAGAAGACATCAATGTGGTCGCCGAGGTGAAG 1080
2-06    GAAAGCATAACGACATCTTCACCACCTGAAGTGGTTGAGATCGCAGTCAATGAAGAAGACGTCAATGTGGTCGCCGAGGTGTAA 972
2-07    GAAAGCGTAACGACATCTTCACCACCTGAAGTGGTC------GCAATCAATGAAGAAGACATCAATGTGGTCGCCGAGGTGTAG 1080
2-08    GAAAGCGTAACGACATCTTCACCACCTGAAGTGGTC------GCAATCAATGAAGAAGACATCAATGTGGTCGCCGAGGTGTAG 1080
2-09    GAAAGCATAACGACATCTTCACCACCTGAAGTGGTTGAGATCGCAGTCAATGAAGAAGACGTCAATGTGGTCGCCGAGGTGTAG 972
2-11    GAAAGCGTAACGACATCTTCACCACCTTAAGTGGTCGAGATCGCAGTCAATGAAGAAGACATCAATGTGGTCGCCGAGGTGTAG 876
2-12    GAAAGCGTAACGACATCTTCACCACCTTAAGTGGTCGAGATCGCGATCAATGA---------------TGTCGCCGAGGTGTAG 861
2-15    GAAAGCGTAACGACATCTTCACCACTTAAAGTGATCGAGATCGCAATCAATGAAGTAGACACCAATGTGGTCGCCGAGGTGTAG 876
2-17    GAAAGCGTAACGACATCTTCACCACCTTAAGTGGTCGAGATCGCAGTCAATGAAGAAGACATCAATGTGGTCGCCGAGGTGTAG 876
2-18    GAAAGCGTAACGACATCTTCACCACCTGAAGTGGTC------GCAATCAATGAAGAAGACATCAATGTGGTCGCCGAGGTGTAA 1080
2-20    GAAAGCGTAACGACATCTTCACCACCTTAAGTGGTCGAGATCGCGATCAATGA---------------TGTCGCCGAGGTGTAG 861
2-21    GAAAGCGTAACGACATCTTCACCACCTGAAGTGGTC------GCAATCAATGAAGAAGACATCAATGTGGTCGCCGAGGTGTAG 1080
2-24    GAAAGCGTAACGACATCTTCACCACCTGAAGTGGTC------GCAATCAATGAAGAAGACATCAATGTGGTCGCCGAGGTGTAA 1080
2-25    GAAAGCGTAACGACATCTTCACCACCTTAAGTGGTCGAGATCGCGATCAATGA---------------TGTCGCCGAGGTGTAG 861
2-28    GAAAGCGTAACGACATCTTCACCACCTGAAGTGGTC------GCAATCAATGAAGAAGACATCAATGTGGTCGCCGAGGTGTAG 1080
2-34    GAAAGCGTAACGACATCTTCACCACTTAAAGTGATCGAGATCGCAATCAATGAAGTAGACACCAATGTGGTCGCCGAGGTGTAG 1431
2-35    GAAAGCGTAACGACATCTTCACCACCTTAAGTGGTCGAGATCGCAGTCAATGAAGAAGACATCAATGTGGTCGCCGAGGTGTAG 876
2-36    GAAAGCGTAACGACATCTTCACCACTTAAAGTGATCGAGATCGCAATCAATGAAGTAGACACCAATGTGGTCGCCGAGGTGTAG 1431
2-37    GAAAGCGTAACGACATCTTCACCACCTTAAGTGGTCGAGATCGCAGTCAATGAAGAAGACATCAATGTGGTCGCCGAGGTGTAG 876
2-38    GAAAGCGTAACGACATCTTCACCACCTGAAGTGGTC------GCAATCAATGAAGAAGACATCAATGTGGTCGCCGAGGTGTAG 1080
2-47    GAAAGCGTAACGACATCTTCACCACCTTAAGTGGTCGAGATCGCAGTCAATGAAGAAGACATCAATGTGGTCGCCGAGGTGTAG 876
2-50    GAAAGCGTAACGACATCTTCACCACCTTAAGTGGTCGAGATCGCGATCAATGA---------------TGTCGCCGAGGTGTAG 771
2-52    GAAAGCGTAACGACATCTTCACCACCTGAAGTGGTC------GCAATCAATGAAGAAGACATCAATGTGGTCGCCGAGGTGTAG 1080
2-55    GAAAGCGTAACGACATCTTCACCACCTGAAGTGGTCGAGATCGCGATC---------------AATGATGTCGCCGAGGTGTAG 975
2-56    GAAAGCGTAACGACATCTTCACCACCTGAAGTGGTC------GCAATCAATGAAGAAGACATCAATGTGGTCGCCGAGGTGTAG 1080
2-57    GAAAGCGTAACGACATCTTCACCACCTGAAGTGGTC------GCAATCAATGAAGAAGACATCAATGTGGTCGCCGAGGTGTAG 1080
2-59    GAAAGCGTAACGACATCTTCACCACCTGAAGTGGTCGAGATCGCGATC---------------AATGATGTCGCCGAGGTGTAG 771
2-61    GAAAGCGTAACGACATCTTCACCACCTTAAGTGGTCGAGATCGCGATCAATGA---------------TGTCGCCGAGGTGTAG 936
2-63    GAAAGCGTAACGACATATTCACCACCTTAAGTGGTCGAGATCGCAGTCAATGAAGAAGACATCAATGGGGTCGCCGAGGTGTAG 876
2-64    GAAAGCGTAACGACATCTTCACCACCTGAAGTGGTC------GCAATCAATGAAGAAGACATCAATGTGGTCGCCGAGGTGTAG 1080
2-65    GAAAGCGTAACGACATCTTCACCACCTGAAGTGGTCGAGATCGCGATCAATGAAGTAGACACCAATGGGGTCGCCGAGGTGTAG 786
2-66    GAAAGCGTAACGACATCTTCACCACCTGAAGTGGTC------GCAATCAATGAAGAAGACATCAATGTGGTCGCCGAGGTGTAG 966
2-67    GAAAGCGTAACGACATCTTCACCACCTTAAGTGGTCGAGATCGCAGTCAATGAAGAAGACATCAATGTGGTCGCCGAGGTGTAG 876
2-73    GAAAGCGTAACGACATCTTCACCACCTGAAGTGGTC------GCAATCAATGAAGAAGACATCAATGTGGTCGCCGAGGTGTAG 870
2-76    GACGGCGTAACGACATCTTCACCACCTGAAGTGGTC------GCAATCAATGAAGAAGACATCAATGTGGTCGCCGAGGTGTAG 1080
2-77    GAAAGCGTAACGACATCTTCACCACCTGAAGTGGTC------GCAATCAATGAAGAAGACATCAATGTGGTCGCCGAGGTGTAG 1080
2-79    GAAAGCGTAACGACATCTTCACCACCTGAAGTGGTC------GCAATCAATGAAGAAGACATCAATGTGGTCGCCGAGGTGTAG 1080
2-82    GAAAGCGTAACGACATCTTCACCACCTGAAGTGGTC------GCAATCAATGAAGAAGACATCAATGTGGTCGCCGAGGAGTAG 1080
2-84    GAAAGCGTAACGACATCTTCACCACCTGAAGTGGTCGAGATCGCGATCAATGA---------------TGTCGCCGAGGTGTAG 771
2-90    GAAAGCGTAACGACATATTCACCACCTTAAGTGGTCGAGATCGCAGTCAATGAAGAAGACATCAATGGGGTCGCCGAGGTGTAT 876
2-91    GAAAGCGTAACGACATCTTCACCACCTGAAGTGGTCGAGATCGCTGACAAT------GACTACGACGTGGTCGCCGAGTCGTAT 870
2-95    GAAAGCGTAACGACATCTTCACCACCTTAAGTGGTCGAGATCGCGATC---------------AATGATGTCGCCGAGGTGTAG 861
2-102   GAAAGCGTAACGACATCTTCACCACCTGAAGTGGTC------GCAATCAATGAAGAAGACATCAATGTGGTCGCCGAGGTGTAG 1080
2-103   GAAAGCATAACGACATCTTCACCACCTGAAGTGGTTGAGATCGCAGTCAATGAAGAAGACGTCAATGTGGTCGCCGAGGTGTAG 972
2-105   GAAAGCGTAACGACATATTCACCACCTTAAGTGGTCGAGATCGCAGTCAATGAAGAAGACATCAATGGGGTCGCCGAGGTGTAG 876
2-106   GAAAGCGTAACGACATCTTCACCACCTGAAGTGGTC------GCAATCAATGAAGAAGACATCAATGTGGTCGCCGAGGTGTAG 1080
2-107   GAAAGCGTAACGACATCTTCACCACCTTAAGTGGTCGAGATCGCAGTCAATGAAGAAGACATCAATGTGGTCGCCGAGGTGTAG 876
2-109   GACGGCGTAACGACATCTTCACCACCTGAAGTGGTC------GCAATCAATGAAGAACACATCAATGTGGTCGCCGAGGTGTAG 1080
2-111   GAAAGCGTAACGACATCTTCACCACCTGAAGTGGTC------GCAATCAATGAAGAAGACATCAATGTGGTCGCCGAGGAGTAG 1080
2-115   GAAAGCGTAACGACATCTTCACCACCTGAAGTGGTC------GCAATCAATGAAGAAGACATCAATGTGGTCGCCGAGGTGTAG 1080
2-116   GAAAGCGTAACGACATCTTCACCACCTGAAGTGGTCGAGATCGCGATCAATGA---------------TGTCGCCGAGGTGTAG 771
2-118   GAAAGCGTAACGACATCTTCACCACCTTAAGTGGTCGAGATCGCGATC---------------AATGATGTCGCCGAGGTGTAG 861
2-119   GAAAGCGTAACGACATCTTCACCACCTTAAGTGGTCGAGATCGCGATCAATGA---------------TGTCGCCGAGGTGTAG 861
4-1501  GAAAGCGTAACGACATCTTCACCACCTGAAGTGGTC------GCAATCAATGAAGAAGACATCAATGTGGTCGCCGAGGTGTAG 1080
4-1503  GAAAGCGTAACGACATCTTCACCACCTGAAGTGGTCGAGATCGCGATCAATGA---------------TGTCGCCGAGGTGTAG 771
4-1508  GAAAGCGTAACGACATCTTCACCACCTGAAGTGGTCGAGATCGCGATCAATGA---------------TGTCGCCGAGGTGTAG 771
4-1511  GAAAGCGTAACGACATCTTCACCACTTAAAGTGATCGAGATCGCAATCAATGAAGTAGACACCAATGTGGTCGCCGAGGTGTAG 1086
4-1515  GAAAGCGTAACGACATCTTCACCACCTGAAGTGGTCGAGATCGCAGTCAATGAAGAAGACATCAATGTGGTCGCCGAGGTGTAG 1086
4-1516  GAAAGCGTAACGACATCTTCACCACCTGAAGTGGTTGAGATCGCATTCAATGAAGAAGACGTCAATGTGGTCGCCGAGGTGTAG 1017
4-1517  GAAAGCGTAACGACATCTTCACCACCTGAAGTGGTCGAGATCGCGATCAATGA---------------TGTCGCCGAGGTGTAG 771
4-1520  GAAAGCGTAACGACATCTTCACCACCTTAAGTGGTCGAGATCGCAGTCAATGAAGAAGACATCAATGATGTCGCCGAGGTGTAG 876
4-1521  GAAAGCGTAACGACATCTTCACCACCTGAAGTGGTTGAGATCGCAGTCAATGAAGAAGACGTCAATGTGGTCGCCGAGGTGTAG 990
4-1523  GAAAGCGTAACGACATCTTCACCACCTGACGTGGTTGAGATCGCAGTCAATGAAGAAGACGTCAATGTGGTCGCCGAGGTGTAG 1086
4-1528  GAAAGCGTAACGACATCTTCACCACCTTAAGTGGTCGAGATCGCAGTCAATGAAGAAGACATCAATGATGTCGCCGAGGTGTAG 951
4-1530  GAAAGCGTAACGACATCTTCACCACCTGAAGTGGTC------GCAATCAATGAAGAAGACATCAATGTGGTCGCTGAGGTGTAG 1080
4-1531  GAAAGCGTAACGACATCTTCACCACCTTAAGTGGTCGAGATCGCAGTCAATGAAGAAGACATCAATGATGTCGCCGAGGTGTAG 876
4-1532  GAAAGCGTAACGACATCTTCACCACCTTAAGTGGTCGAGATCGCAGTCAATGAAGAAGACATCAATGATGTCGCCGAGGTGTAG 876
4-1533  GAAAGCGTAACGACATCTTCACCACCTTAAGTGGTCGAGATCGCAGTCAATGAAGAAGACATCAATGATGTCGCTGAGGTGTAG 876
4-1534  GAAAGCATAACGACATCTTCACCACCTGAAGTGGTTGAGATCGCAGTCAATGAAGAAGACGTCAATATGGTCGCCGAGGTGTAG 990
4-1535  GAAAGCGTAACGACATCTTCACCACCTGAAGTGGTCGAGATCGCGATCAATGA---------------GGTCGCCGAGGTGTAG 771
4-1536  GAAAGCGTAACGACATCTTCACCACCTGAAGTGGTTGAGATCGCAGTCAATGAAGAAGACGTCAATGTGGTCGCCGAGGTGTAG 849
4-1538  GAAAGCGTAGCGACATCTTCACCACCTGAAGTGGTTGAAATCGCAGTCAATGAAGAAGACGTCAATGTGGTCGCTGAGGTGTAG 1086
4-1542  GAAAGCGTAACGACATCTTCACCGCCTTAAGTGGTCGAGATCGCAGTCAATGAAGAAGACATCAATGATGTCGCTGAGGTGTAG 876
4-1543  GAAAGCGTAACGACATCTTCACCACCTGAAGTGGTC------GCAATCAATGAAGAAGACATCAATGTGGTCGCCGAGGTGTAG 1080
4-1544  GAAAGCGTAACGACATCTTCACCACCTGAAGTGGTTGAGATCGCAGTCAATGAAGAAGACGTCAATGTGGTCGCCGAGGTGTAG 1017
```

```
                                …Er27a                           Er27b
                1450      1460      1470      1480      1490      1500      1510      1520
        ....|....|....|....|....|....|....|....|....|....|....|....|....|....|....|....|....
4-1545  GAAAGCGTAACGACATCTTCACCACCTGAAGTGGTTGAGATCGCAGTCAATGAAGAAGACGTCAATGTGGTCGCCGAGGTGTAG 1092
4-1547  GAAAGCGTAACGACATCTTCACCACCTGAAGTGGTCGAGATCGCGATCAATGA---------------TGTCGCCGAGGTGTAG 771
4-1548  GAAAGCATAACGACATCTTCACCACCTGAAGTGGTTGAGATCGCAGTCAATGAAGAAGACGTCAATATGGTCGCCGAGGTGTAG 990
4-1550  GAAAGCGTAACGACATCTTCACCACCTTAAGTGGTCGAGATCGCAGTCAATGAAGAAGACATCAATGTGGTCGCCGAGGTGTAG 876
4-2410  GAAAGCGTAACGACATCTTCACCACCTGAAGTGGTC------GCAATCAATGAAGAAGACATCAATGTGGTCGCCGAGGTGTAG 1080
4-2412  GAAAGCGTAACGACATCTTCACCACCTGAAGTGGTC------GCAATCAATGAAGAAGACATCAATGTGGTCGCCGAGGTGTAG 1080
4-2415  GAAAGCATAACGACATCTTCACCACCTGAAGTGGTTGAGATCGCAGTCAATGAAGAAGACGTCAATATGGTCGCCGAGGTGTAA 990
4-2449  GAAAGCGTAACGACATCTTCACCACCTGAAGTGGTC------GCAATCAATGAAGAAGACATCAATGTGGTCGCCGAGGTGTAG 1080
10-01   GAAAGCGTAACGACATCTTCACCACCTGAAGTGGTTGAGATCGCAGTCAATGAAGAAGACGTCAATGTGGTCGCCGAGGTGTAG 990
10-02   GAAAGCGTAACGACATCTTCACCACCTGAAGTGGTTGAGATCGCAGTCAATGAAGAAGACGTCAATGTGGTCGCCGAGGTGTAG 990
10-03   GAAAGCGTAACGACATCTTCACCACCTGAAGTGGTCGAGATCGCGATCAATGA---------------TGTCGCCGAGGTGTAG 771
10-04   GAAAGCGTAACGACATCTTCACCACCTGAAGTGGTC------GCAATCAATGAAGAAGACATCAATGTGGTCGCCGAGGTGTAG 1005
10-06   GAAAGCGTAACGACATCTTCACCACCTGAAGTGGTC------GCAATCAATGAAGAAGACATCAATGTGGTCGCCGAGGTGTAG 1080
10-07   GAAAGCGTAACGACATCTTCACCACCTGAAGTGGTTGAGATCGCATTCAATGAAGAAGACGTCCATGTGGTCGCCGAGGTGTAG 888
10-10   GAAAGCGTAACGACATCTTCACCACTTAAAGTGATCGAGATCGCAATCAATGAAGTAGACACCAATGTGGTCGCCGAGGTGTAG 1428
10-11   GAAAGCGTAACGACATCTTCACCACCTGAAGTGGTC------GCAATCAATGAAGAAGACATCAATGTGGTCGCCGAGGTGTAG 984
10-13   GAAAGCGTAACGACATCTTCACCACCTGAAGTGGTTGAGATCGCAGTCAATGAAGAAGACGTCAATGTGGTCGCCGAGGTGTAG 1011
10-14   GAAAGCGTAACGACATCTTCACCACCTGAAGTGGTC------GCAATCAATGAAGAAGACATCAATGTGGTCGCCGAGGTGTAG 1080
10-15   GAAAGCGTAACGACATCTTCACCACCTGAAGTGGTC------GCAATCAATGAAGAAGACATCAATGTGGTCGCCGAGGTGTAG 1080
10-17   GAAAGCGTAACGACATCTTCACCACCTGAAGTGGTTGAGATCGCAGTCAATGAAGAAGACGTCAATGTGGTCGCCGAGGTGTAG 990
10-18   GAAAGCGTAACGACATCTTCACCACCTGAAGTGGTTGAGATCGCATTCAATGAAGAAGACGTCAATGTGGTCGCCGAGGTGTAG 888
10-19   GAAAGCGTAACGACATCTTCACCACCTGAAGTGGTC------GCAATCAATGAAGAAGACATCAATGTGGTCGCCGAGGTGTAG 1059
10-20   GAAAGCGTAACGACATATTCACCACCTTAAGTGGTCGAGATCGCAGTCAATGAAGAAGACATCAATGGGGTCGCCGAGGTGTAG 876
10-21   GAAAGCGTAACGACATCTTCACCACCTGAAGTGGTC------GCAATCAATGAAGAAGACATCAATGTGGTCGCCGAGGTGTAG 1080
10-22   GAAAGCGTAACGACATCTTCACCACCTGAAGTGGTC------GCAATCAATGAAGAAGACATCAATGTGGTCGCCGAGGTGTAG 1080
10-24   GAAAGCGTAACGACATCTTCACCACCTGAAGTGGTTGAGATCGCAGTCAATGAAGAAGACGTCAATGTGGTCGCCGAGGTGTAG 1086
10-25   GAAAGCGTAACGACATCTTCACCACCTGAAGTGGTCGAGATCGCGATCAATGA---------------TGTCGCCGAGGTGTAG 771
10-26   GAAAGCGTAACGACATATTCACCACCTTAAGTGGTCGAGATCGCAGTCAATGAAGAAGACATCAATGGGGTCGCCGAGGTGTAG 876
10-28   GAAAGCGTAACGACATCTTCACCACCTGAAGTGGTCGAGATCGCGATCAATGA---------------TGTCGCCGAGGTGTAG 771
10-29   GAAAGCGTAACGACATCTTCACCACCTGAAGTGGTCGAGATCGCGATCAATGA---------------TGTCGCCGAGGTGTAG 771
10-30   GAAAGCGTAACGACATCTTCACCACCTGAAGTGGTCGAGATCGCGATCAATGA---------------TGTCGCCGAGGTGTAG 771
10-31   GAAAGCGTAACGACATCTTCACCACCTGAAGTGGTTGAGATCGCAGTCAATGAAGAAGACGTCAATGTGGTCGCCGAGGTGTAG 990
10-32   GAAAGCGTAACGACATCTTCACCACCTGAAGTGGTCGAGATCGCGATCAATGA---------------TGTCGCCGAGGTGTAG 771
10-35   GAAAGCGTAACGACATCTCCACCAGCTGAAGTGGTTGAGATCGCATTCAATGAAGAAGACGTCAATGTGGTCGCCGAGGTGTAG 1017
10-37   GAAAGCGTAACGACATCTTCACCACCTGAAGTGGTCGAGATCGCGATCAATGA---------------TGTCGCCGAGGTGTAG 771
10-38   GAAAGCGTAACGACATCTTCACCACCTGAAGTGGTCGAGATCGCGATCAATGA---------------TGTCGCCGAGGTGTAG 771
10-39   GAAAGCGTAACGACATCTTCACCACCTGAAGTGGTTGAGATCGCAGTCAATGAAGAAGACGTCAATGTGGTCGCCGAGGTGTAG 990
10-40   GAAAGCGTAACGACATCTTCACCACCTGAAGTGGTCGAGATCGCGATCAATGA---------------TGTCGCCGAGGTGTAG 975
10-42   GAAAGCGTAACGACATCTTCACCACCTGAAGTGGTTGAGATCGCAGTCAATGAAGAAGACGTCAATGTGGTCGCCGAGGTGTTA 990
10-43   GAAAGCGTAACGACATCTTCACCACCTGAAGTGGTTGAGATCGCAGTCAATGAAGAAGACGTCAATGTGGTCGCCGAGGTGTTA 990
10-46   GAAAGCGTAACGACATCTTCACCACCTGAAGTGGTCGAGATCGCGATCAATGA---------------TGTGGCCGAGGTGTAG 771
10-50   GAAAGCGTAACGACATCTTCACCACCTGAAGTGGTTGAGATCGCATTCAATGAAGAAGACGTCAATGTGGTCGCCGAGGTGTAG 1017
10-51   GAAAGCGTAACGACATCTCCACCACCTGAAGTGGTCGAGATCGCAGTCAATGAAGAAGACGTCAATGTGGTCGCCGAGGTGTAG 990
10-52   GAAAGCGTAACGACATCTTCACCACCTGAAGTGGTCGAGATCGCGATCAATGA---------------TGTCGCCGAGGTGTAG 771
10-54   GAAAGCGTAACGACATCTTCACCACCTGAAGTGGTTGAGATCGCATTCAATGAAGAAGACGTCAATGTGGTCGCCGAGGTGTAG 888
10-55   GAAAGCGTAACGACATCTTCACCACCTGAAGTGGTTGAGATCGCAGTCAATGAAGAAGACGTCAATGTGGTCGCCGAGGTGTAG 1086
```
