# Supplementary material for: Extraordinary diversity among members of the large gene family, 185/333, from the purple sea urchin, Strongylocentrotus purpuratus
Source: BMC Mol Biol. 2007 Aug 15;8:68. doi: 10.1186/1471-2199-8-68 (PMC1988830; doi:10.1186/1471-2199-8-68)
Supplement: Additional file 7 — Repeat-based amino acid alignment of the 185/333 genes. The repeat-based alignment of the deduced protein sequence of the second exons of the 121 unique gene sequences showing the element borders and repeats. [file 1471-2199-8-68-S7.pdf]

Additional File 7: Repeat-based alignment of the encoded amino acid sequences. Only the second exon alignment is shown; the leader alignment is identical to that shown in Additional File 3. Element borders are indicated by vertical black lines, and are labeled above the sequence. Repeats are indicated by shaded boxes, which each different color (red = type 1; blue = type 2; green = type 3; yellow = type 4; and purple = type 5).

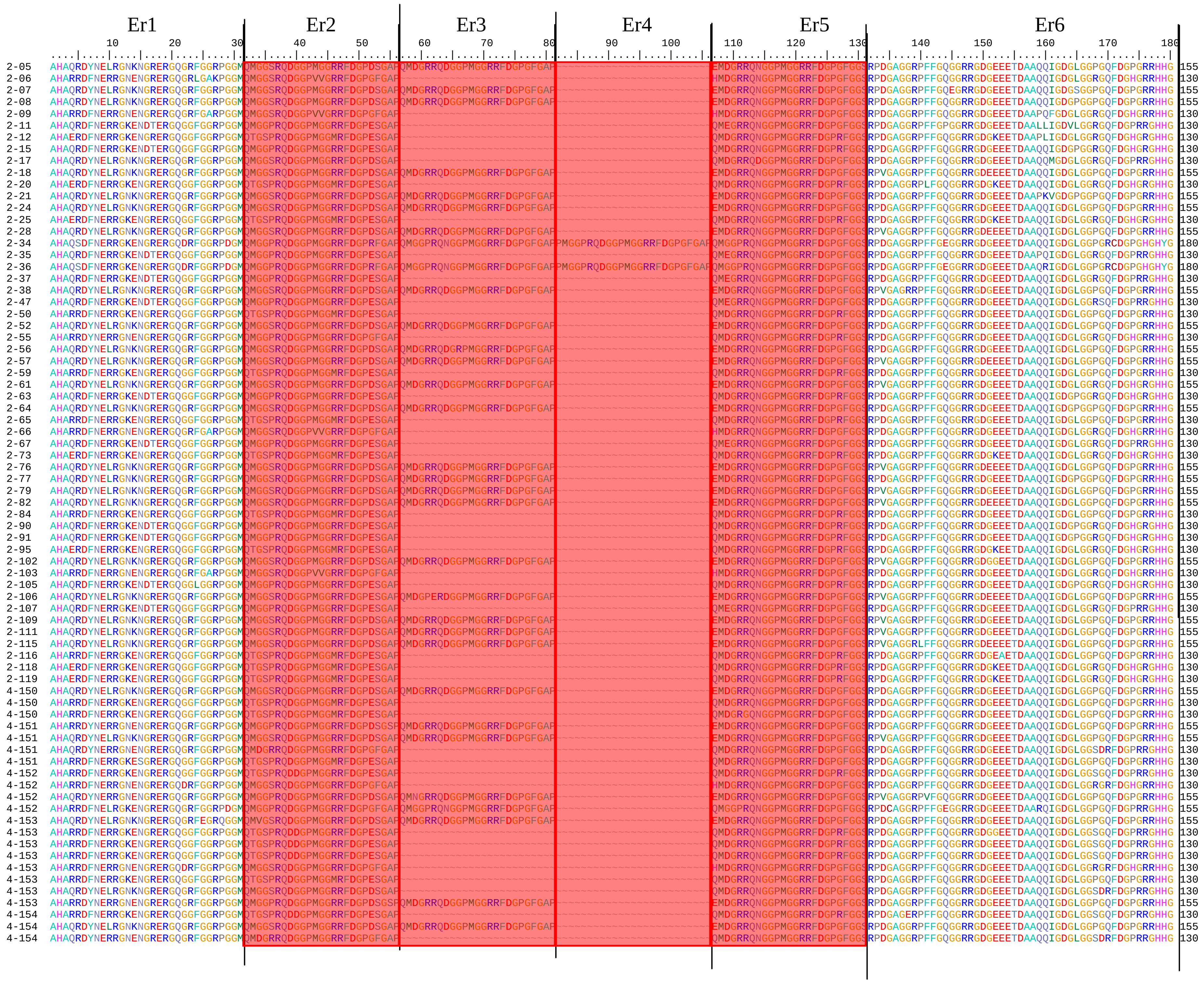

Er1
Er2
Er3
Er4
Er5
Er6

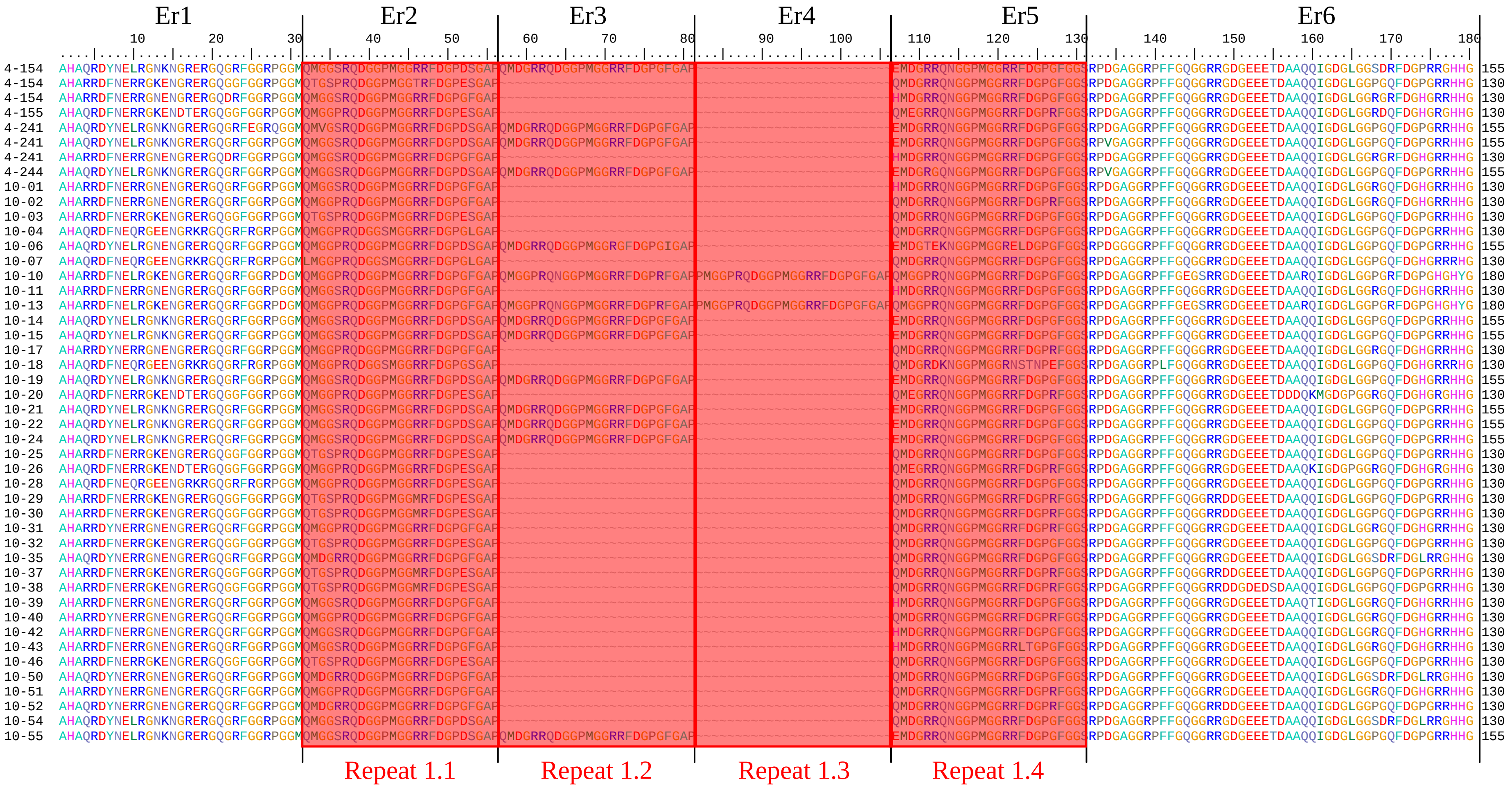
Er1
Er2
Er3
Er4
Er5
Er6
Repeat 1.1
Repeat 1.2
Repeat 1.3
Repeat 1.4

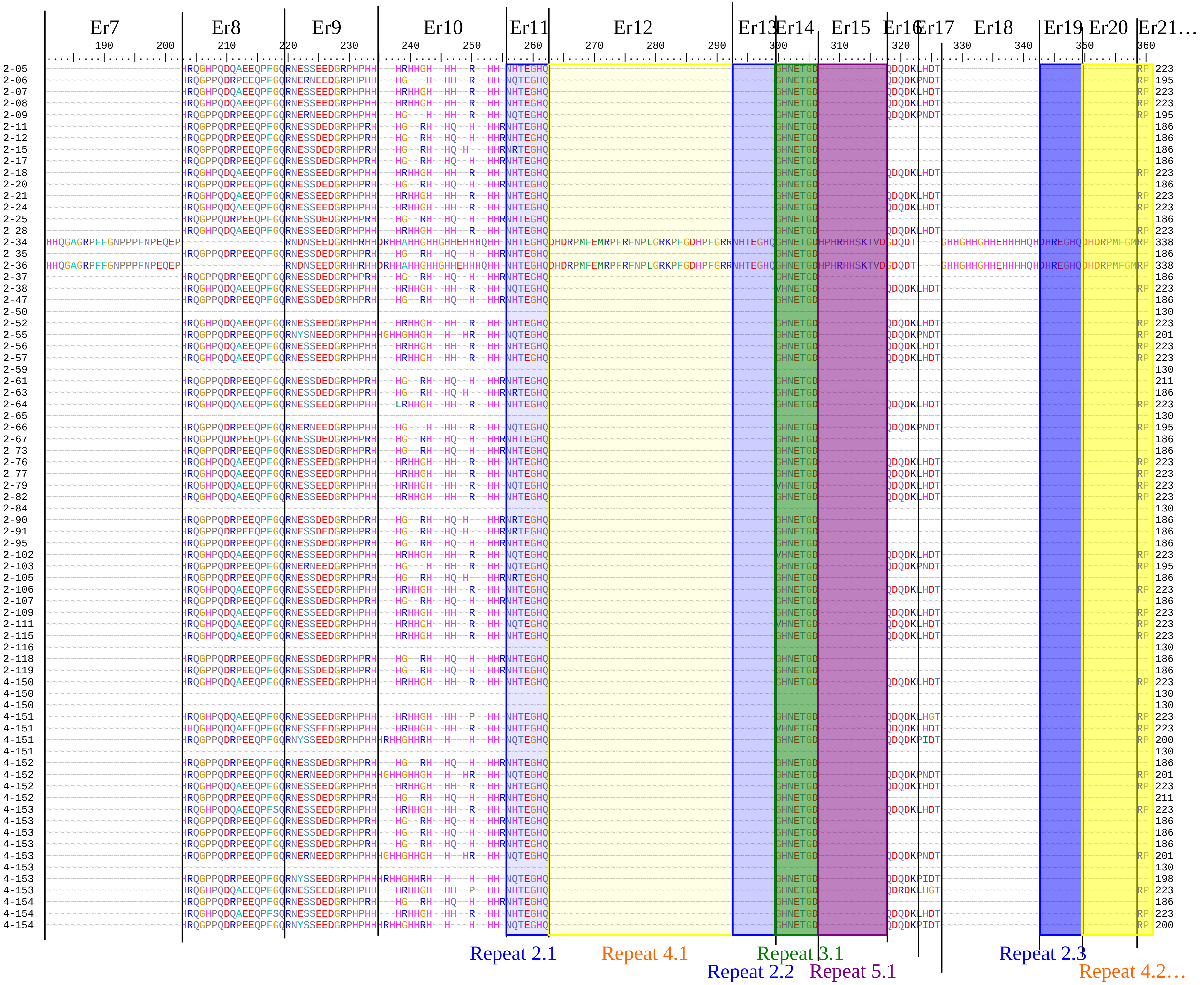
Er7
Er8
Er9
Er10
Er11
Er12
Er13
Er14
Er15
Er16
Er17
Er18
Er19
Er20
Er21…
Repeat 2.1
Repeat 4.1
Repeat 3.1
Repeat 2.2
Repeat 5.1
Repeat 2.3
Repeat 4.2…

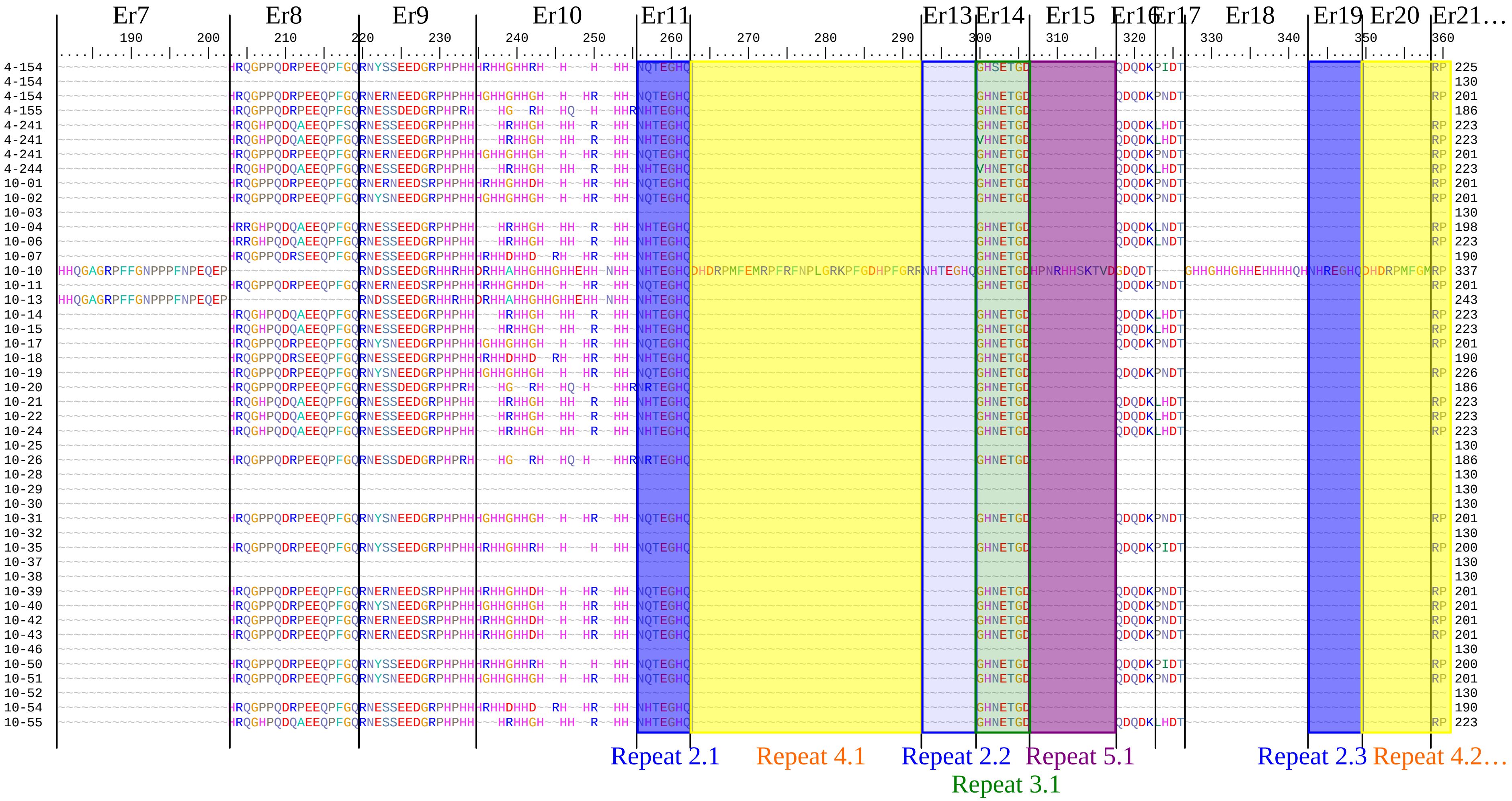
Er7
Er8
Er9
Er10
Er11
Er13
Er14
Er15
Er16
Er17
Er18
Er19
Er20
Er21…
Repeat 2.1
Repeat 4.1
Repeat 2.2
Repeat 5.1
Repeat 3.1
Repeat 2.3
Repeat 4.2…

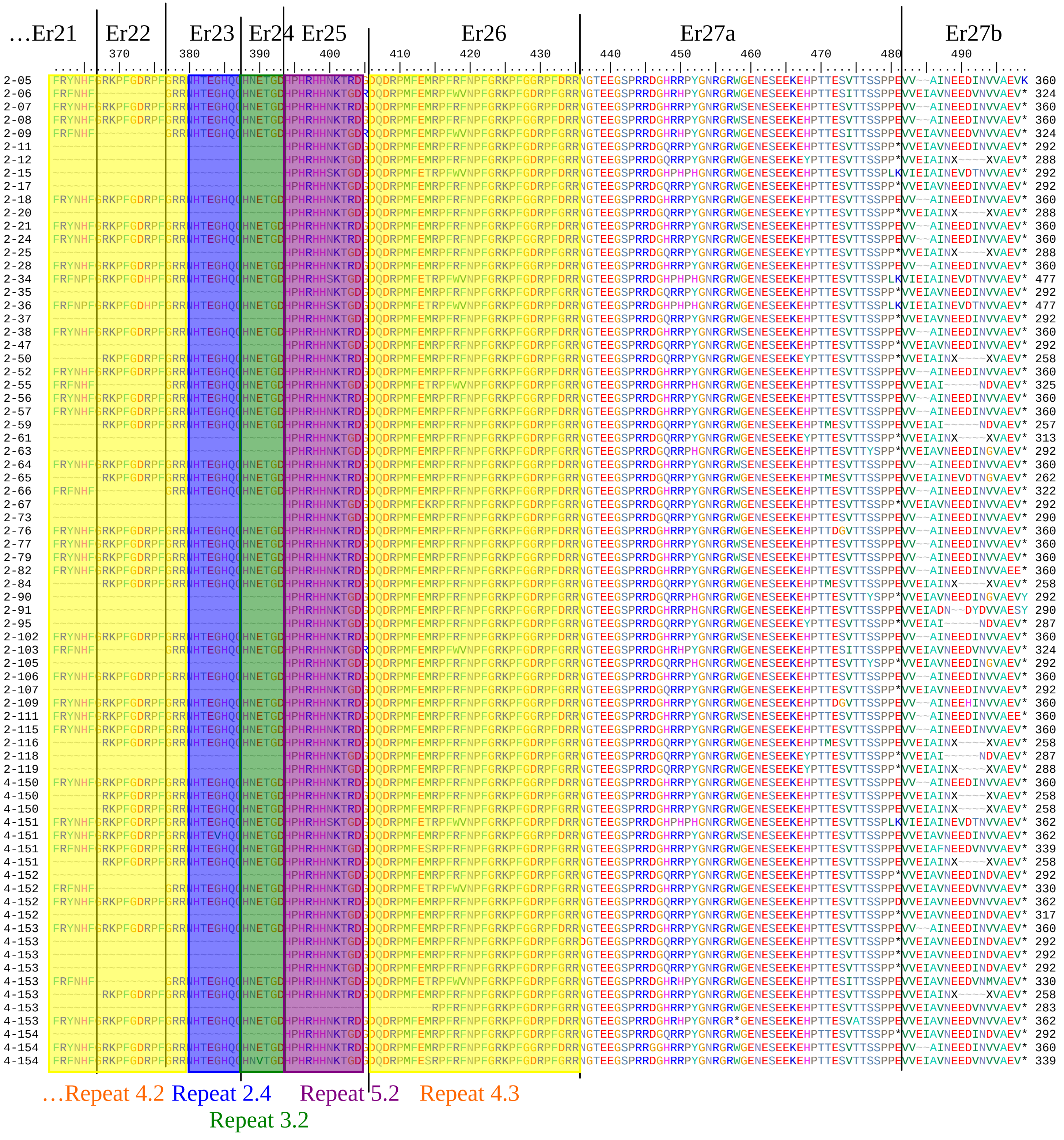
…Er21
Er22
Er23
Er24
Er25
Er26
Er27a
Er27b
…Repeat 4.2
Repeat 2.4
Repeat 3.2
Repeat 5.2
Repeat 4.3

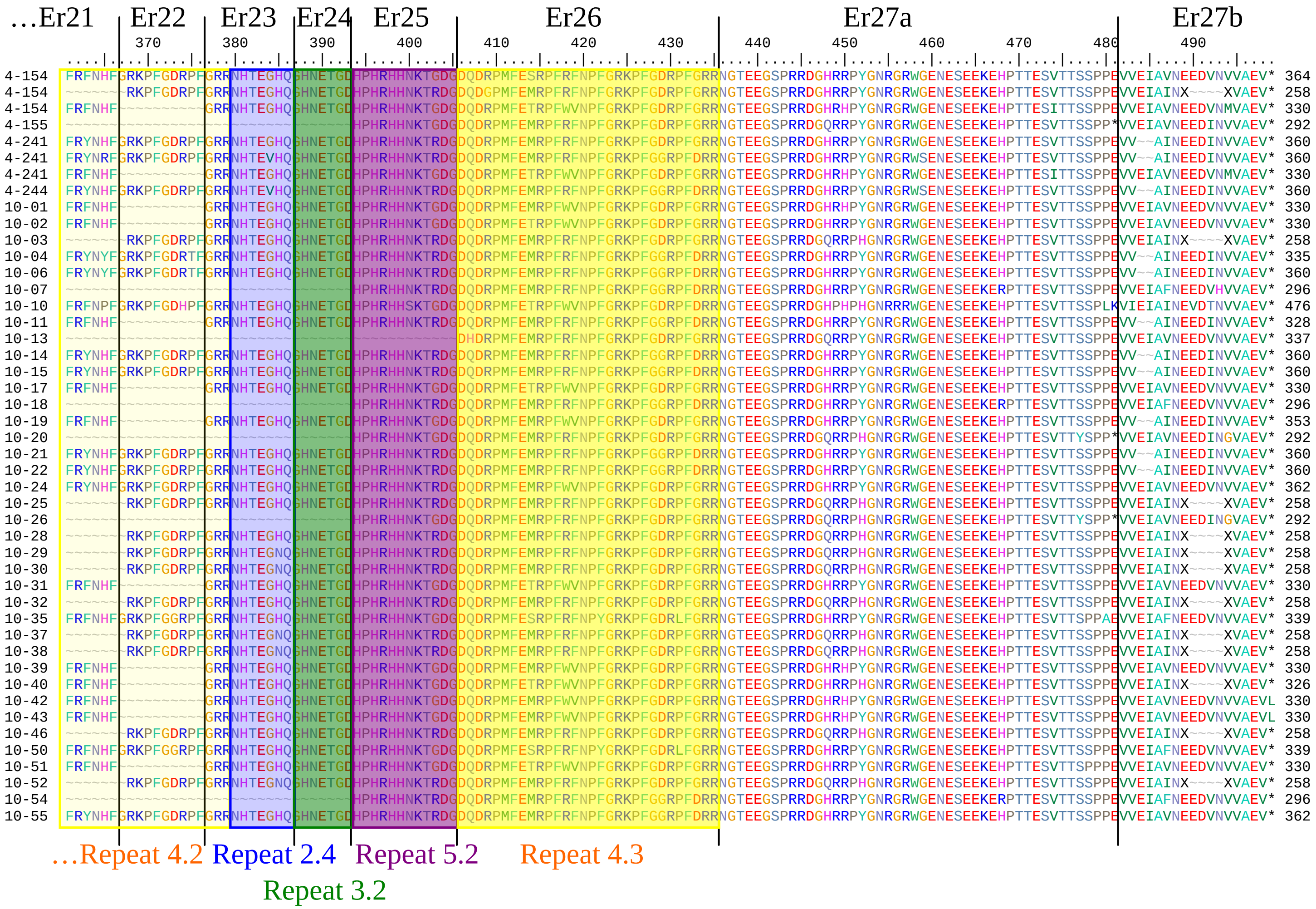
…Er21
Er22
Er23
Er24
Er25
Er26
Er27a
Er27b
…Repeat 4.2
Repeat 2.4
Repeat 5.2
Repeat 4.3
Repeat 3.2
