# Supplementary material for: Extraordinary diversity among members of the large gene family, 185/333, from the purple sea urchin, Strongylocentrotus purpuratus
Source: BMC Mol Biol. 2007 Aug 15;8:68. doi: 10.1186/1471-2199-8-68 (PMC1988830; doi:10.1186/1471-2199-8-68)
Supplement: Additional file 8 — Alignment of the 185/333 genes isolated from two BACs. The nucleotide and amino acid sequence of the five unique 185/333 genes isolated from two separate BACs, aligned with the cDNA-based alignment. [file 1471-2199-8-68-S8.pdf]

Additional File 8: Alignment of the gene sequences isolated from BACs. The aligned nucleotide and amino acid sequences for the five unique genes isolated from two BACs are shown. Clones 091-01, 091-02 and 091-07 were isolated from BAC 091D11. Clones 190-03 and 190-04 were isolated from BAC 190G12.

```
               10        20        30        40        50        60        70        80        90       100       110       120       130       140       150
       ....|....|....|....|....|....|....|....|....|....|....|....|....|....|....|....|....|....|....|....|....|....|....|....|....|....|....|....|....|....|
091-02 ATGGAGGTGAAAGTGACACTGATCGTTGCCATTGTGGCTGCTCTTGCTATCTCGG----------------------------------------------------------------------------------------------- 54
        M  E  V  K  V  T  L  I  V  A  I  V  A  A  L  A  I  S
091-07 ATGGAGGTGAAAGTGACACTGATCGTTGCCATTGTGGCTGCTCTTGCTATCTCGG----------------------------------------------------------------------------------------------- 54
        M  E  V  K  V  T  L  I  V  A  I  V  A  A  L  A  I  S
091-01 ATGGAGGTGAAAGCAACATTGATCGTTGCCATTGTGGCTGCTCTTGCTATCTCGGGTAAGAAATCAAATT-TTACTCGGTATTACTTCATAAGTGCTAAATATAA-AGCCAATGAAAGGGCTCACAGAAGTAAATATGATTATTT-CATA 147
        M  E  V  K  A  T  L  I  V  A  I  V  A  A  L  A  I  S
190-03 CTG---GTGAAAGTGACACTGATCGTTGCCATTGTGGCTGCTCTTGTTATCTCAGGTAAGAAATCAAATTATTACTCGGTATTACTTGATAAGTGCTAAACTATA-GAGCCAACGAATAGCTCACAGAAGTATATATGATTATTT-CATA 145
        L  -  V  K  V  T  L  I  V  A  I  V  A  A  L  V  I  S
190-04 ATGCTGGTGAAAGTGACACTGATCGTTGCCATTGTGGCTGCTTTTGCTATCTCGGGTAAGAAATCAAATTATTACTCGGTATTCTTTCATAAGTGGTA--TATTG--------------------------------------------- 103
        M  L  V  K  V  T  L  I  V  A  I  V  A  A  F  A  I  S

              160       170       180       190       200       210       220       230       240       250       260       270       280       290       300
       ....|....|....|....|....|....|....|....|....|....|....|....|....|....|....|....|....|....|....|....|....|....|....|....|....|....|....|....|....|....|
091-02 ------------------------------------------------------------------------------------------------------------------------------------------------------ 54
091-07 ------------------------------------------------------------------------------------------------------------------------------------------------------ 54
091-01 GTTTGTAATATGCATTTTTAAATTGTTCGTTACACAATATAA---------------------------------------------TTAATTGTATTTATTAAGCCTACACCAATCCGTTTTCTGAATGACAAAGGATGTGTATTATGT 252
190-03 TTTTATAATATGCATTTCTAAATTGTTCGTTACAAAATATAA---------------------------------------------TTTATATTATTTCTTAAGCCTACACCAATCCGTTAGGCGAGTGGCAAAAGATAAGAATTATCT 250
190-04 -------------------AACTTGTTTGTGACACAATAAAA-AATAACATTTATTCTGCGGCCTGCAGCATTCT-GTTTTTTTT------------------------------------------ATGAAAAAA-ATGTTTCA--TGG 187

              310       320       330       340       350       360       370       380       390       400       410       420       430       440       450
       ....|....|....|....|....|....|....|....|....|....|....|....|....|....|....|....|....|....|....|....|....|....|....|....|....|....|....|....|....|....|
091-02 ------------------------------------------------------------------------------------------------------------------------------------------------------ 54
091-07 ------------------------------------------------------------------------------------------------------------------------------------------------------ 54
091-01 TTACGTTCAATCTGGTAATCAAATTTAATTCAGTCAGAATTAGGCGTTTTGAATATCGAACCGC---------------------------------------------------------------ATTTA--CGGATTAGGATTAAAC 337
190-03 TTATGTTCAATCTGGTATTCAAGTTCAATTCAGTCAGAATTAGGCGTTTTGAATATCGAACCGA---------------------------------------------------------------------------TAGGGTTAAAC 325
190-04 TACTTTACAATCTGGTTTTCAAATTCAATTCGGTTAAAATTAGGCGTTTTGAATATCCAACCGCATGCATGCATTCTGGTAGAGTTAAACATAACGGGACCAGACATCGAAGAAGACAAATCTCCTA----------------------- 314

              460       470       480       490       500       510       520       530       540       550       560       570       580       590       600
       ....|....|....|....|....|....|....|....|....|....|....|....|....|....|....|....|....|....|....|....|....|....|....|....|....|....|....|....|....|....|
091-02 ------------------------------------------------------------------------------------------------------------------------------------------------------ 54
091-07 ------------------------------------------------------------------------------------------------------------------------------------------------------ 54
091-01 ATAAA--GGGAAAAATAT---AAGAAGGCGAGTTACCATTCTTATTTGTCACCTGCTATAAACCAATGTAGAGCAAAATATAATAATGCAGAATGTGATTAC-----TTAATTAATTCGATTATTT---------CATTAACCCTTACAG 467
190-03 ACAAA--GGGAAAAAACATCAAAGAAGGCGAGTTACCATTCTTATTTGTCACCTGCCATAACCCAATGTAGAGCTAAAGATAATAATGCAGAATGTGATCAC-----TTAATTAATTCGATTTTTTT-----CTTCATTATCCCCTACAG 462
190-04 -----------------------------------------------------GC-ATAAACCAATGTAGAGCTAAAGATAATAATGCAGAATGTGATTAATTATTTTATTAATAAATTCGACTATT--------CCATTATCCCAACAG 401

              610       620       630       640       650       660       670       680       690       700       710       720       730       740       750
       ....|....|....|....|....|....|....|....|....|....|....|....|....|....|....|....|....|....|....|....|....|....|....|....|....|....|....|....|....|....|
091-02 ----CTCACGCACAAAGAGATTTCAATGAACGACGAGGAAAGGAGAATGACACAGAGAGAGGACAAGGTGGCTTTGGAGGAAGGCCTGGTGGAATGCAGATGGGTGGTCCTAGGCAAGATGGTGGACCAATGGGTGGAAGGAGGTTCGAT 201
           A  H  A  Q  R  D  F  N  E  R  R  G  K  E  N  D  T  E  R  G  Q  G  G  F  G  G  R  P  G  G  M  Q  M  G  G  P  R  Q  D  G  G  P  M  G  G  R  R  F  D
091-07 ----CTCACGCACAAAGAGATTTCAATGAACGACGAGGAAAGGAGAATGACACAGAGAGAGGACAAGGTCGCTTTGGAGGAAGGCCTGGTGGAATGCAGA-------------------------------------------------- 151
           A  H  A  Q  R  D  F  N  E  R  R  G  K  E  N  D  T  E  R  G  Q  G  R  F  G  G  R  P  G  G  M  Q  X  -  -  -  -  -  -  -  -  -  -  -  -  -  -  -  -
091-01 ----CTCACGCACGAAGAGATTTCAATGAACGGCGAGGAAATGAGAATGGCAGAGAGAGAGGACAAGGTCGCTTTGGAGGAAGGCCTGGTGGAATGCAGATGGGTGGATCTAGGCAAGATGGTGGACCAATGGGTGGAAGGAGGTTCGAT 614
           A  H  A  R  R  D  F  N  E  R  R  G  N  E  N  G  R  E  R  G  Q  G  R  F  G  G  R  P  G  G  M  Q  M  G  G  S  R  Q  D  G  G  P  M  G  G  R  R  F  D
190-03 ----CTCACGCACAAAGAGATTACAATGAACTACGAGGAAATAAGAATGGCAGAGAGAGAGGACAAGGTCGCTTTGGAGGAAGGCCGGGTGGAATGCAGATGGGTGGATCGAGGCAAGATGGTGGACCAATGGGTGGGAGAAGGTTCGAT 609
           A  H  A  Q  R  D  Y  N  E  L  R  G  N  K  N  G  R  E  R  G  Q  G  R  F  G  G  R  P  G  G  M  Q  M  G  G  S  R  Q  D  G  G  P  M  G  G  R  R  F  D
190-04 ----CTCACGCACGAAGAGATTTCAATGAACGACGAGGAAAGGAGAATGGCAGAGAGAGAGGACAAGGTGGCTTTGGAGGAAGGCCTGGTGGAATGCAGACGGGTAGTCCTAGGCAAGATGGTGGACCAATGGGTGGAATGAGGTTCGAT 548
           A  H  A  R  R  D  F  N  E  R  R  G  K  E  N  G  R  E  R  G  Q  G  G  F  G  G  R  P  G  G  M  Q  T  G  S  P  R  Q  D  G  G  P  M  G  G  M  R  F  D

              760       770       780       790       800       810       820       830       840       850       860       870       880       890       900
       ....|....|....|....|....|....|....|....|....|....|....|....|....|....|....|....|....|....|....|....|....|....|....|....|....|....|....|....|....|....|
091-02 GGACCTGACTCTGGTGCCCCACAAATGGATGGACGCAGACAAAATGGCGGTCCGATGGGTGGTAGG---------------------------------------------------------------------------AGATTCGAC 276
        G  P  D  S  G  A  P  Q  M  D  G  R  R  Q  N  G  G  P  M  G  G  R  -  -  -  -  -  -  -  -  -  -  -  -  -  -  -  -  -  -  -  -  -  -  -  -  -  R  F  D
091-07 ---------------------------------------CAAAATGGCGGTCCGATGGGTGGTAGG---------------------------------------------------------------------------AGATTCGAC 187
        -  -  -  -  -  -  -  -  -  -  -  -  -  Q  N  G  G  P  M  G  G  R  -  -  -  -  -  -  -  -  -  -  -  -  -  -  -  -  -  -  -  -  -  -  -  -  -  R  F  D
091-01 GGACCTGGATTTGGTGCCCCACATATGGATGGACGCAGACAAAATGGCGGTCCGATGGGTGGTAGG---------------------------------------------------------------------------AGATTCGAC 689
        G  P  G  F  G  A  P  H  M  D  G  R  R  Q  N  G  G  P  M  G  G  R  -  -  -  -  -  -  -  -  -  -  -  -  -  -  -  -  -  -  -  -  -  -  -  -  -  R  F  D
190-03 GGACCTGACTCTGGTGCCCCACAAATGGATGGACGGAGACAAGATGGTGGACCAATGGGTGGAAGGAGGTTCGATGGACCTGGATTTGGTGCCCCGGAGATGGATGGACGTAGACAAAATGGCGGTCCGATGGGTGGAAGGAGATTCGAC 759
        G  P  D  S  G  A  P  Q  M  D  G  R  R  Q  D  G  G  P  M  G  G  R  R  F  D  G  P  G  F  G  A  P  E  M  D  G  R  R  Q  N  G  G  P  M  G  G  R  R  F  D
190-04 GGACCTGAATCTGGTGCCCCACAAATGGATGGACGCAGACAAAATGGCGGTCCGATGGGTGGTAGG---------------------------------------------------------------------------AGATTCGAC 623
        G  P  E  S  G  A  P  Q  M  D  G  R  R  Q  N  G  G  P  M  G  G  R  -  -  -  -  -  -  -  -  -  -  -  -  -  -  -  -  -  -  -  -  -  -  -  -  -  R  F  D
```

```
                910       920       930       940       950       960       970       980       990      1000      1010      1020      1030      1040      1050
       ....|....|....|....|....|....|....|....|....|....|....|....|....|....|....|....|....|....|....|....|....|....|....|....|....|....|....|....|....|....|
091-02 GGACCTGGATTTGGTGGCTCCAGACCAGTTGGTGCTGGAGGGAGACCTTTCTTCGGCCAAGGAGGCAGGCGTGGTGATGGAGAAGAAGAAACTGATGCTGCCCAACAAATTGGTGATGGTCTAGGAGGGCGCGGTCAGTTTGATGGT--- 423
        G  P  G  F  G  G  S  R  P  V  G  A  G  G  R  P  F  F  G  Q  G  G  R  R  G  D  G  E  E  E  T  D  A  A  Q  Q  I  G  D  G  L  G  G  R  G  Q  F  D  G  - 
091-07 GGACCTCGATTTGGTGGCTCCAGACCAGATGGTGCTGGAGGGAGACCTTTCTTCGGCCAAGGAGGCAGGCGTGGTGATGGAGAAGAAGAAACTGATGCTGCCCAACAAATTGGTGATGGTCTAGGAGGGCGCGGTCAGTTCAATGGT--- 334
        G  P  R  F  G  G  S  R  P  D  G  A  G  G  R  P  F  F  G  Q  G  G  R  R  G  D  G  E  E  E  T  D  A  A  Q  Q  I  G  D  G  L  G  G  R  G  Q  F  N  G  - 
091-01 GGACCTGGATTTGGTGGCTCCAGACCAGATGGTGCTGGAGGAAGACCTTTCTTCGGCCAAGGAGGAAGGCGTGGTGATGGAGAAGAAGAAACTGATGCTGCCCAACAAATTGGTGATGGTCTAGGAGGGCGCGGTCAGTTTGATGGT--- 836
        G  P  G  F  G  G  S  R  P  D  G  A  G  G  R  P  F  F  G  Q  G  G  R  R  G  D  G  E  E  E  T  D  A  A  Q  Q  I  G  D  G  L  G  G  R  G  Q  F  D  G  - 
190-03 GGACCTGGATTTGGTGGCTCCAGACCAGATGGTGCTGGAGGAAGACCTTTCTTCGGCCAAGGAGGCAGGCGTGGTGATGGAGAAGAAGAAACTGATGCTGCCCAACAAATTGGTGATGGTCTAGGAGGGCCCGGTCAGTTTGATGGTCCT 909
        G  P  G  F  G  G  S  R  P  D  G  A  G  G  R  P  F  F  G  Q  G  G  R  R  G  D  G  E  E  E  T  D  A  A  Q  Q  I  G  D  G  L  G  G  P  G  Q  F  D  G  P 
190-04 GGACCTGGATTTGGTGGCTCCAGACCAGATGGTGCTGGAGGAAGACCTTTCTTCGGCCAAGGAGGCAGGCGTGGTGATGGAGAAGAAGAAACTGATGCTGCCCAACAAATTGGTGATGGTCTAGGAGGGCCCGGTCAGTTTGATGGTCCT 773
        G  P  G  F  G  G  S  R  P  D  G  A  G  G  R  P  F  F  G  Q  G  G  R  R  G  D  G  E  E  E  T  D  A  A  Q  Q  I  G  D  G  L  G  G  P  G  Q  F  D  G  P 

               1060      1070      1080      1090      1100      1110      1120      1130      1140      1150      1160      1170      1180      1190      1200
       ....|....|....|....|....|....|....|....|....|....|....|....|....|....|....|....|....|....|....|....|....|....|....|....|....|....|....|....|....|....|
091-02 ------------------CATGGACGTGGACATCATGGTCACCGTCAAGGTCCTCCTCAGGACCGACCAGAGGAACAACCGTTCGGTCAGCGCAACGAAAGCAGCGATGAGGGTGGCCGTCCTCACCCTCGCCACCATGGCCGCCACCAC 555
        -  -  -  -  -  -  H  G  R  G  H  H  G  H  R  Q  G  P  P  Q  D  R  P  E  E  Q  P  F  G  Q  R  N  E  S  S  D  E  G  G  R  P  H  P  R  H  H  G  R  H  H 
091-07 ------------------CATGGACGTGGACATCATGGTCACCGTCAAGGTCCTCCTCATGACCGACCAGAGGAACAACCGTTCGGTCAGCGCAACGAAAGCAGCGATGAGGATGGCCGTCCTCACCCTCGCCACCATGGCCGCCACCAC 466
        -  -  -  -  -  -  H  G  R  G  H  H  G  H  R  Q  G  P  P  H  D  R  P  E  E  Q  P  F  G  Q  R  N  E  S  S  D  E  D  G  R  P  H  P  R  H  H  G  R  H  H 
091-01 ------------------CATGGACGTCGTCATCATGGTCACCGTCAAGGTCCACCTCAGGACCGACCAGAGGAACAACCGTTCGGCCAGCGCAATGAACGCAACGAGGAGGATGGCCGTCCTCACCCTCACCACCATGGCCAC------ 962
        -  -  -  -  -  -  H  G  R  R  H  H  G  H  R  Q  G  P  P  Q  D  R  P  E  E  Q  P  F  G  Q  R  N  E  R  N  E  E  D  G  R  P  H  P  H  H  H  G  H  -  - 
190-04 GGACGTCGTCATCATGGT---------------------CACCGTCAAGGTCATCCTCAGGACCAAGCAGAGGAACAACCGTTTGGTCAGCGCAACGAAAGCAGCGAGGAGGATGGCCGTCCTCACCCTCACCACCATCGCCAC------ 1032
        G  R  R  H  H  G  -  -  -  -  -  -  -  H  R  Q  G  H  P  Q  D  Q  A  E  E  Q  P  F  G  Q  R  N  E  S  S  E  E  D  G  R  P  H  P  H  H  H  R  H  -  - 
190-03 GGACGTCGTCATCATGGT------------------------------------------------------------------------------------------------------------------------------------ 791
        G  R  R  H  H  G  -  -  -  -  -  -  -  -  -  -  -  -  -  -  -  -  -  -  -  -  -  -  -  -  -  -  -  -  -  -  -  -  -  -  -  -  -  -  -  -  -  -  -  - 

                1210      1220      1230      1240      1250      1260      1270      1280      1290      1300      1310      1320      1330      1340      1350
       ....|....|....|....|....|....|....|....|....|....|....|....|....|....|....|....|....|....|....|....|....|....|....|....|....|....|....|....|....|....|
091-02 CAGCATCATCAT------------------------------------------------------------------------------------------------------------------------------------------ 567
        Q  H  H  H  -  -  -  -  -  -  -  -  -  -  -  -  -  -  -  -  -  -  -  -  -  -  -  -  -  -  -  -  -  -  -  -  -  -  -  -  -  -  -  -  -  -  -  -  -  - 
091-07 CAGCATCATCAT------------------------------------------------------------------------------------------------------------------------------------------ 478
        Q  H  H  H  -  -  -  -  -  -  -  -  -  -  -  -  -  -  -  -  -  -  -  -  -  -  -  -  -  -  -  -  -  -  -  -  -  -  -  -  -  -  -  -  -  -  -  -  -  - 
091-01 ---CATGGCCACCATGGCCACCACCACCGTCATCATAACCAGACAGAGGGTCACCAAGGACATAATGAGACGGGAGATCAAGATCAGGACAAACCAAATGATACGAGGCCCTTCCGGTTCAACCACTTCGGC------------------ 1091
        -  H  G  H  H  G  H  H  H  R  H  H  N  Q  T  E  G  H  Q  G  H  N  E  T  G  D  Q  D  Q  D  K  P  N  D  T  R  P  F  R  F  N  H  F  G  -  -  -  -  -  - 
190-03 ---CATGGCCGCCACCAC---CGT------CATCATAACCACACAGAAGGTCACCAAGGTCATAATGAGACGGGAGATCAAGATCAGGACAAACTACATGATACAAGGCCCTTCCGGTACAACCACTTCGGTAGAAAGCCTTTCGGAGAC 1170
        -  H  G  R  H  H  -  R  -  -  H  H  N  H  T  E  G  H  Q  G  H  N  E  T  G  D  Q  D  Q  D  K  L  H  D  T  R  P  F  R  Y  N  H  F  G  R  K  P  F  G  D 
190-04 ------------------------------------------------------------------------------------------------------------------------------------AGAAAGCCTTTCGGAGAC 809
        -  -  -  -  -  -  -  -  -  -  -  -  -  -  -  -  -  -  -  -  -  -  -  -  -  -  -  -  -  -  -  -  -  -  -  -  -  -  -  -  -  -  -  -  R  K  P  F  G  D 

                1360      1370      1380      1390      1400      1410      1420      1430      1440      1450      1460      1470      1480      1490      1500
       ....|....|....|....|....|....|....|....|....|....|....|....|....|....|....|....|....|....|....|....|....|....|....|....|....|....|....|....|....|....|
091-02 ---------------CGCAACCACACAGAAGGTCACCAAGGTCACAATGAGACAGGAGATCACCCCCACCGTCATCACAACAAGACCGGAGATGGAGATCAGGACAGACCAATGTTTGAGATGAGGCCCTTCCGGTTCAACCCCTTCGGT 702
        -  -  -  -  -  R  N  H  T  E  G  H  Q  G  H  N  E  T  G  D  H  P  H  R  H  H  N  K  T  G  D  G  D  Q  D  R  P  M  F  E  M  R  P  F  R  F  N  P  F  G 
091-07 ---------------CGCAACCACACAGAAGGTCACCAAGGTCACAATGAGACAGGAGATCACCCCCACCGTCATCACAACAAGACCGGAGATGGAGATCAGGACAGACCAATGTTTGAGATGAGGCCCTTCCGGTTTAACCCCTTCGGT 613
        -  -  -  -  -  R  N  H  T  E  G  H  Q  G  H  N  E  T  G  D  H  P  H  R  H  H  N  K  T  G  D  G  D  Q  D  R  P  M  F  E  M  R  P  F  R  F  N  P  F  G 
091-01 ------------AGACGCAACCACACAGAAGGTCACCAGGGTCATAATGAGACGGGAGATCACCCCCATCGTCATCACAACAAGACCGGAGATGGAGATCAGGACAGACCAATGTTTGAGATGAGG------------------------ 1205
        -  -  -  -  R  R  N  H  T  E  G  H  Q  G  H  N  E  T  G  D  H  P  H  R  H  H  N  K  T  G  D  G  D  Q  D  R  P  M  F  E  M  R  -  -  -  -  -  -  -  - 
190-03 CGTCCCTTCGGCAGACGCAACCATACAGAAGGTCACCAGGGTCATAATGAGACGGGAGACCACCCCCATCGTCATCACAACAAGACCAGAGATGGAGATCAGGACAGACCAATGTTCGAGATGAGGCCTTTCCGGTTCAACCCCTTCGGT 1320
        R  P  F  G  R  R  N  H  T  E  G  H  Q  G  H  N  E  T  G  D  H  P  H  R  H  H  N  K  T  R  D  G  D  Q  D  R  P  M  F  E  M  R  P  F  R  F  N  P  F  G 
190-04 CGTCCCTTCGGCAGACGCAACCATACAGAAGGTCACCAGGGTCATAATGAGACGGGAGACCACCCCCATCGTCATCACAACAAGACCAGAGATGGAGATCAGGACAGACCAATGTTCGAGATGAGGCCCTTTCGGTTCAACCCCTTCGGT 959
        R  P  F  G  R  R  N  H  T  E  G  H  Q  G  H  N  E  T  G  D  H  P  H  R  H  H  N  K  T  R  D  G  D  Q  D  R  P  M  F  E  M  R  P  F  R  F  N  P  F  G 

                1510      1520      1530      1540      1550      1560      1570      1580      1590      1600      1610      1620      1630      1640      1650
       ....|....|....|....|....|....|....|....|....|....|....|....|....|....|....|....|....|....|....|....|....|....|....|....|....|....|....|....|....|....|
091-02 AGAAAGCCTTTCGGAGACCGTCCCTTCGGCAGACGC------------------------------------------------------------AACGGAACCGAGGAAGGATCTCCCAGGCGTGATGGCCAACGTCGGCCCTATGGT 792
        R  K  P  F  G  D  R  P  F  G  R  R  -  -  -  -  -  -  -  -  -  -  -  -  -  -  -  -  -  -  -  -  N  G  T  E  E  G  S  P  R  R  D  G  Q  R  R  P  Y  G 
091-07 AGAAAGCCTTTCGGAGACCGTCCCTTCGGCAGACGC------------------------------------------------------------AACGGAACCGAGGAAGGATCTCCCAGGCGTGATGGCCAACGTCGGCCCTATGGT 703
        R  K  P  F  G  D  R  P  F  G  R  R  -  -  -  -  -  -  -  -  -  -  -  -  -  -  -  -  -  -  -  -  N  G  T  E  E  G  S  P  R  R  D  G  Q  R  R  P  Y  G 
091-01 ------------------------------------CCCTTCTGGGTCAACCCCTTCGGTAGAAAGCCTTTCGGAGACCGTCCCTTCGACAGACGCAACGGAACCGAAGAAGGATCTCCCAGGCGTGATGGCCACCGTCATCCCTATGGT 1319
        -  -  -  -  -  -  -  -  -  -  -  -  P  F  W  V  N  P  F  G  R  K  P  F  G  D  R  P  F  D  R  R  N  G  T  E  E  G  S  P  R  R  D  G  H  R  H  P  Y  G 
190-03 AGAAAGCCTTTCGGAGGCCGTCCCTTCGACAGACGC------------------------------------------------------------AACGGAACCGAAGAAGGATCTCCCAGGCGTGATGGCCATCGTCGGCCCTATGGT 1410
        R  K  P  F  G  G  R  P  F  D  R  R  -  -  -  -  -  -  -  -  -  -  -  -  -  -  -  -  -  -  -  -  N  G  T  E  E  G  S  P  R  R  D  G  H  R  R  P  Y  G 
190-04 AGAAAGCCTTTCGGAGACCGTCCCTTCGGCAGACGC------------------------------------------------------------AACGGAACCGAGGAAGGATCTCCCAGGCGTGATGGCCAACGTCGGCCCCATGGT 1049
        R  K  P  F  G  D  R  P  F  G  R  R  -  -  -  -  -  -  -  -  -  -  -  -  -  -  -  -  -  -  -  -  N  G  T  E  E  G  S  P  R  R  D  G  Q  R  R  P  H  G 
```

```
                1660      1670      1680      1690      1700      1710      1720      1730      1740      1750      1760      1770      1780
       ....|....|....|....|....|....|....|....|....|....|....|....|....|....|....|....|....|....|....|....|....|....|....|....|....|....|....|...
091-02 AACCGAGGACGTTGGGGTGAGAATGAAAGTGAGGAGAAGGAGCATCCAACGACGGAAAGCGTAACGACATCTTCACCACCTTAAGTGGTCGAGATCGCAGTCAATGAAGAAGACATCTATGTGGTCGCCGAGGTGTAG 930
        N  R  G  R  W  G  E  N  E  S  E  E  K  E  H  P  T  T  E  S  V  T  T  S  S  P  P  *  V  V  E  I  A  V  N  E  E  D  I  Y  V  V  A  E  V  * 
091-07 AACCGAGGACGTTGGGGTGAGAATGAAAGTGAGGAGAAGGAGCATCCAACGACGGAAAGCGTAACGACATCTTCACCACCTTAAGTGGTCGAGATCGCAGTCAATGAAGAAGACATCAATGTGGTCGCCGAGGTGTAG 841
        N  R  G  R  W  G  E  N  E  S  E  E  K  E  H  P  T  T  E  S  V  T  T  S  S  P  P  *  V  V  E  I  A  V  N  E  E  D  I  N  V  V  A  E  V  * 
091-01 AACCGAGGACGTTGGGGTGAGAATGAAAGTGAGGAGAAGGAGCATCCAACGACGGAAAGCATAACGACATCTTCACCACCTGAAGTGGTTGAGATCGCAGTCAATGAAGAAGACGTCCAAGTGGTCGCCGAGGTGTAG 1457
        N  R  G  R  W  G  E  N  E  S  E  E  K  E  H  P  T  T  E  S  I  T  T  S  S  P  P  E  V  V  E  I  A  V  N  E  E  D  V  Q  V  V  A  E  V  * 
190-03 AACCGAGGACGTTGGGGTGAGAATGAAAGTGAGGAGAAGGAGCATCCAACGACGGAAAGCGTAACGACATCTTCACCACCG                                                         1491
        N  R  G  R  W  G  E  N  E  S  E  E  K  E  H  P  T  T  E  S  V  T  T  S  S  P  P 
190-04 AACCGAGGACGTTGGGGTGAGAATGAAAGTGAGGAGAAGGAGCATCCAACGACGGAAAGCGTAACGACATCTTCACCACCTGACGTGGTCGAGATCGCGATCAAT---------------GATGTCGCCGAGGTGTAG 1172
        N  R  G  R  W  G  E  N  E  S  E  E  K  E  H  P  T  T  E  S  V  T  T  S  S  P  P  D  V  V  E  I  A  I  N  -  -  -  -  -  D  V  A  E  V  * 
```
